# Nitrogen application and differences in leaf number retained after topping affect the tobacco (*Nicotiana tabacum*) transcriptome and metabolome

**DOI:** 10.1186/s12870-022-03426-x

**Published:** 2022-01-19

**Authors:** Bo Lei, Wei Chang, Huina Zhao, Kai Zhang, Jing Yu, Shizhou Yu, Kai Cai, Jie Zhang, Kun Lu

**Affiliations:** 1 Molecular Genetics Key Laboratory of China Tobacco, China National Tobacco Corporation, Guizhou Academy of Tobacco Science, Guiyang, 550081 China; 2 Upland Flue-Cured Tobacco Quality and Ecology Key Laboratory of China Tobacco, Guizhou Academy of Tobacco Science, Guiyang, 550081 China; 3 College of Agronomy and Biotechnology, Southwest University, Beibei, Chongqing, 400715 China; 4 Academy of Agricultural Sciences, Southwest University, Chongqing, 400715 China; 5 Engineering Research Center of South Upland Agriculture, Ministry of Education, Chongqing, 400715 China

**Keywords:** *Nicotiana tabacum*, Transcriptome, Metabolome, Nitrogen, Leaf number retained

## Abstract

**Background:**

Agronomic treatments such as the application of nitrogen fertilizer and topping (removal of the inflorescence and top leaves) cause substantial changes in plant metabolism. To explore these changes, we conducted comparative transcriptomic and metabolomic analyses of leaves collected from four positions along the stem on plants exposed to two nitrogen doses and with different numbers of leaves retained after topping in tobacco (*Nicotiana tabacum*).

**Results:**

We identified 13,330 unique differentially expressed genes and 32 differentially abundant metabolites. Through RNA-seq and WGCNA analyze, we constructed 2 co-expression networks (green and blue) highly correlation to N application and leaf number retained, predicted a hub gene *NtGER3* may play an important role in N metabolism related to amino acid (cysteine) through CK pathway in tobacco leaves, *NtARFs* may participated in modulating the auxin signal and N in bottom leaves and *NtRAP2.12* as key gene involved in N regulation by ethylene pathway. What’s more, our data prove C/N transformation and balance affect the “source – flow - sink” redistribution and remobilization in tobacco during growth and development process.

**Conclusions:**

Overall, this comparative transcriptomics study provides novel insight into the complex molecular mechanisms underlying plant responses to different levels of nitrogen application and the number of leaves remaining after topping in plants.

**Supplementary Information:**

The online version contains supplementary material available at 10.1186/s12870-022-03426-x.

## Background

Plants as sessile organisms are faced varying nitrogen (N) demand by whole growth, N supply is often the limiting factor and affecting the production of biomass and metabolic processes [1, 2]. Plants rely on N assimilation from nitrate (NO_3_^−^) or ammonium (NH_4_^+^) [1] and under aerobic conditions, the predominant form of N in the soil is nitrate, which is taken up in roots and transported throughout the plant by members of the NITRATE TRANSPORTER 1 (NRT1)/PEPTIDE TRANSPORTER (PTR) Family (NPF), NRT2, CHLORIDE CHANNEL (CLC), and SLOWLY ACTIVATING ANION CHANNEL (SLAC) families of nitrate transporters [3].

NO_3_^−^ also participates in signaling and the transcription factor (TF) NIN-LIKE PROTEIN7 (NLP7) has been proposed as a master regulator in NO_3_^−^ signaling [4]. NO_3_^−^ plays a critical role in regulating embryo [5] and root development, photosynthesis [6], stress tolerance [7], and senescence [8] in *Arabidopsis thaliana* and crops. Not surprisingly, most of these regulatory pathways intersect with phytohormone signaling pathways [9–12]. The more important is that the interaction between N and cytokinins (CKs) are manifested by nitrate-induced CK synthesis in roots and CK-induced expression of N-related genes, *OsIPT4*, *OsIPT5*, *OsIPT7* and *OsIPT8* were up-regulated in response to exogenously applied nitrate and ammonium with accompanying accumulation of CKs in rice [13]. And the exogenous application of CK can repress the expression of Arabidopsis *NRT2* genes [14]. CKs also mainly in the form of *trans*-zeatin-riboside (*tZR*) and *trans*-zeatin (*tZ*) function as the long-distance signals through xylem transport, genes involved in glutamate and glutamine biosynthesis are identified as potential targets of *tZ* regulation, which indicating a possible role of amino acids in the long-distance shoot-to-root N signalling [15, 16].

Another major form of N present in the soil is NH_4_^+^, which is abundant in flooded wetland or acidic soils [2]. NH_4_^+^ is absorbed by plant roots via ammonium transporters (AMTs) [17] and assimilated into amino acids via the glutamine synthetase (GS) /glutamine-2-oxoglutarate aminotransferase (GOGAT) cycle [18].

In most plants, N is primarily assimilated into amino acids in roots or shoots [19]. Within plant leaves, N is stored as amino acids or proteins to sustain plant growth, or is loaded into the phloem to supply N to developing or temporary sink tissues [20–23]. In pea (*Pisum sativum*), overexpressing *AMINO ACID PERMEASE1* (*AAP1*) improved N uptake or utilization efficiency [19]. Amino acids participate in the tricarboxylic acid (TCA) cycle, produce the energy needed by the cell [24], and regulate the carbon and nitrogen (C/N) balance [25].

Topping is an important and traditional agronomic measure that involves removing the flower, often as early as a bud, and chopping off a set number of leaves from the top of the plant, which will affect leaf number retained in the plant. Topping increased the expression of *NtNAC-R1*, encoding a NAM, ATAF1/2 and CUC2 (NAC) domain TF and reduced expression of the microRNA miR164, and resulted in an increase in root indole-3-acetic acid (IAA) content that influences lateral root formation and affects jasmonic acid (JA) signaling, leading to an increase in nicotine content, indicating that topping affect leaf number retained caused crosstalk between JA and auxin signaling [26]. Furthermore, previous transcriptomic data showed that topping affected C and N metabolism, photosynthesis, and secondary metabolism in tobacco, while the amendment of soil with straw-based biochar before topping enhanced amino acid and lipid biosynthesis [27]. Different leaf number retained after topping influences nutrient biosynthesis and distribution during plant growth by profoundly resetting the source–sink relationship throughout the plant body.

Here, we chose tobacco as a model to study the effects of different leaf number retained and nitrogen application. We conducted RNA sequencing (RNA-seq) and metabolite profiling following a split-plot experimental design to assess the effects of two different N concentrations and two distinct numbers of leaves retained, sampled in four sets of leaves positioned along the main stem of tobacco plants. Combining RNA-seq and metabolomic data with weighted gene co-expression network analysis (WGCNA), we identified two networks and several hub genes linked to N applications and the number of leaves retained after topping. These results provide insight about crosstalk between N application and the number of leaves retained and how topping reprograms plant transcriptomes to affect leaf N compounds.

## Results

### Measurement of different metabolites in tobacco leaves

To unravel the relationship between N and amino acid metabolism, we measured the contents of 32 metabolites, such as total N, protein, amino acids and several polyamines (PAs) et al. in tobacco plants (Supplementary Table 2). We used plants that had been treated with two different doses of exogenous N: A1 (pure N 3 kg/667 m^2^) and A2 (pure N 6 kg/667 m^2^), and with 12 (B1) or 16 (B2) leaves remaining on each plant after topping, then we sampled leaves from the bottom, middle, upper, and top parts of the plant.

**Fig. 1:**
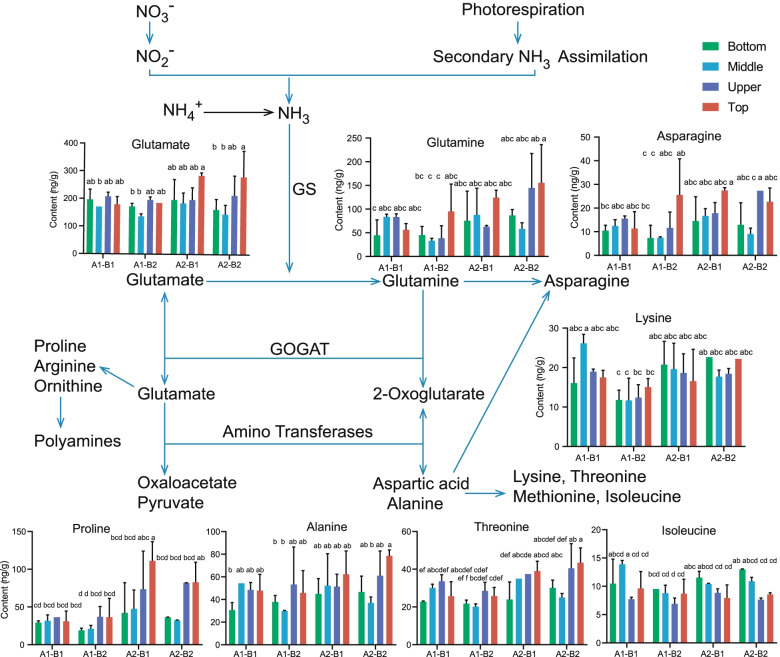
Effects of N nutrition and plant topping on the main nitrate and ammonium assimilation pathways leading to amino acid biosynthesis. Values are shown as means ± standard error (SE) of three biological replicates. Different letters indicate a significant difference (Tukey’s multiple comparison test, *P* < 0.05). GS: Glutamine Synthetase; GOGAT: Glutamine Synthetase

We next calculated correlations between metabolites, revealing a strong positive correlation (*r* = 0.89) between total protein and total N content (Supplementary Fig. 2), which followed the same trend in bottom, middle, and upper leaves (Supplementary Fig. 3). We observed positive correlations between some amino acids, such as leucine, lysine, and tyrosine, which may reflect their shared metabolic pathways (Supplementary Fig. 2). Combining the effects of nitrogen supplementation on amino acid levels (Fig. 1) and the metabolite data (Supplementary Table 2) showed that in the presence of ATP, organic ammonium and glutamate are converted into glutamine by glutamine synthetase (GS), while inorganic nitrogen is converted into organic nitrogen (nitrate) that can then be absorbed and utilized by plants. Proline, phenylalanine, threonine, and asparagine contents were positively correlated with the N dose applied (Fig. 1). In terms of number of leaves retained, our results indicated that leaf contents of aspartic acid, glutamic acid, proline, leucine, isoleucine lysine and tyrosine decreased with an increase in the number of leaves retained (Supplementary Table 2). Our results therefore suggest that the dose of N application and the number of leaves retained affect the content, the types, and metabolic processes of free amino acids in tobacco leaves.

PAs regulate plant growth and responses to stress, PA anabolism is similar to that of arginine and ornithine [28]. We examined the content of several important PAs, the contents and accumulation patterns of putrescine and spermidine were similar, as they both exhibited their highest levels in the A2B2 condition (high N dose and more leaves retained after topping), with an average putrescine content of 10.11 ng/g and an average spermidine content of 9.75 ng/g (Supplementary Fig. 2, Supplementary Table 2).

### Transcriptome analysis

Transcriptome analysis was performed to investigate the changes in tobacco leaves of plants experiencing different doses of N application and different number of leaves retained after topping, we collected samples for deep sequencing of the RNA-seq. After filtering low-quality reads and trimming adapters, we obtained ~ 997 million raw reads, with an average of ~ 31 million reads per sample, corresponding to 127 Gb of sequence in total, with ~ 4 Gb of data per sample on average (Supplementary Table 3). Based on 69,500 annotated tobacco genes and a coding space of only 325.3 Mb (excluding promoters and introns), we estimated the coverage of our RNA-seq data to be about 20X, which should be sufficient to detect genes with low transcript abundance.

Salmon (v0.8.2) was used to map ~ 865 million clean reads to the tobacco reference genome v4.5 (https://solgenomics.net/organism/Nicotiana_tabacum/genome), resulting in an average of ~ 2.9 million mapped reads per sample (80.9 to 85.0% of clean starting reads, Supplementary Table 3). These results indicated that we obtained high-quality sequencing results, and we have enough sequencing data for our subsequent analysis.

### Identification of differentially expressed genes (DEGs) under different treatments

**Fig. 2:**
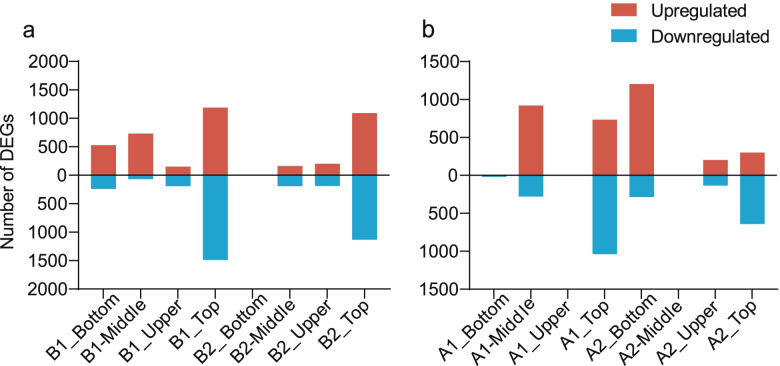
Number of Differentially Expressed Genes (DEGs) identified as a function of N nutrition and plant topping in tobacco

We identified 7560 DEGs in response to N supply across the four collection points for leaves and using the low N dose (A1) as control for the higher N dose treatment (A2), using cutoffs for false discovery rate (FDR) < 0.05 and absolute log_2_(fold change) ≥2 (Fig. 2a, Supplementary Table 3). We detected the highest number of DEGs in the samples collected from the top leaves regardless of the N dose applied. The number of DEGs showed little relationship with the number of leaves retained. When we retained 12 leaves after topping (condition B1), we identified the fewest DEGs in the top leaves, but the lowest leaves showed the fewest DEGs when we left 16 leaves on the plants (condition B2). It is worth noting that when we kept 12 leaves on plants after topping, a majority of DEGs in the bottom and middle leaf positions were up-regulated, indicating that sufficient N supply may modulate the entire plant transcriptional program, improving the ability of plants to perform various biological functions.

We then turned to a comparison of leaf transcriptomes focusing on the effect of the number of leaves retained (12 or 16) for the two N doses applied and the four leaf positions under consideration, which resulted in the identification of 5770 DEGs when12 leaves retained (B1) as the control (Fig. 2b, Supplementary Table 4). With the lower N dose (A1), the top leaves showed the highest number of DEGs, while the bottom leaves were associated with the most DEGs at the higher N dose (A2). The lower N dose (A1) yielded the largest number of up-regulated DEGs in the middle leaves, and the higher N dose (A2) yielded most up-regulated DEGs in the bottom leaves. We saw the highest number of down-regulated genes in the top leaves for both N doses, possibly indicating that newly emerged leaves produce a more-pronounced response to external stimuli.

### GO term enrichment analysis of DEGs

By performing GO functional enrichment analysis, under different N doses, when the number of leaves retained was 12, up-regulated genes were mainly enriched in biological processes such as photosynthesis (GO: 0015979), organo-nitrogen compound metabolic process (GO: 1901564) and down-regulated genes were largely enriched in response to organo-nitrogen compound (GO: 0010243), defense response (GO: 0006952), and hormone-mediated signaling pathway (GO: 0009755) (Supplementary Table 5). These results may reflect the better growth displayed by plants exposed to adequate N fertilization, and how they may be better able to adapt to external biotic and abiotic stress. Under these conditions, the plants may mobilize less resources toward plant resistance through phytohormone responses and other means.

However, the response of bottom and middle leaves to different N doses clearly differed from the response of the top leaves. Genes that respond to external stimuli in the middle leaves showed a general up-regulation, while genes involved in organic N assimilation were mainly up-regulated in the middle leaves (Supplementary Table 5). We hypothesize that bottom and middle leaves may compete for N distribution: although N will preferentially supply the bottom leaves near the root under normal conditions, relative N deficiency will allocate more N to the middle leaves for better photosynthesis.

We detected few DEGs in upper leaves, indicating that these leaves were not directly involved in the changes in N metabolism. GO enrichment analysis indicated that top leaves experienced an inhibition of auxin transport activity and a reduction in photosynthetic capacity related to plant growth and development under N sufficiency (A2), whereas their ability to respond to organic N substances improved, a situation opposite to that of bottom leaves (Supplementary Table 5).

GO enrichment analysis yielded very similar results when the number of leaves retained was 16, with the exception of the top leaves, where up-regulated genes are largely involved in phloem development (GO: 0010088) and terpene biosynthetic process (GO: 0046246), while down-regulated genes are mainly involved in the regulation of phenylpropanoid metabolic process (GO: 2000762) and aminoglycan catabolic process (GO: 0006026). The expression profile changes for top leaves were much more pronounced in response to the two N doses relative to leaves in other positions. Top leaves may regulate their N content by inducing genes related to amino acid biosynthesis or degradation pathways (Supplementary Table 5). Thus, we hypothesize that N distribution in the plant is closely linked to leaf position as well as the number of leaves retained after topping.

At the lower N dose (A1), we detected too few DEGs in bottom and upper leaves for subsequent analysis. By contrast, in middle leaves, up-regulated genes followed clear patterns, with enrichment in xyloglucan metabolic process (GO: 0010411), carbohydrate metabolic process (GO: 0005975), response to amino glycan metabolism, and response to organic N compounds. Down-regulated genes were associated with biological processes such as response to karrikin (GO: 0080167) and flavonoid biosynthetic process (GO: 0009813) (Supplementary Table 5). Up-regulated genes in top leaves were mainly related to various stress responses and down-regulated genes were largely involved in photosynthesis (GO: 0015979), carbohydrate metabolic process (GO: 0005975), and cell wall organization (GO: 0071555) (Supplementary Table 5). The enrichment of these DEGs indicated that, in order to conserve energy for the formation and development of new leaves, the genes related to cell wall biosynthesis and photosynthesis were significantly down-regulated at the lower N dose. At the same time, the entire plant may become more sensitive to external environmental stress, resulting in a more frequent adjustment of transcriptional outputs.

### Construction and analysis of co-expression networks

**Fig. 3:**
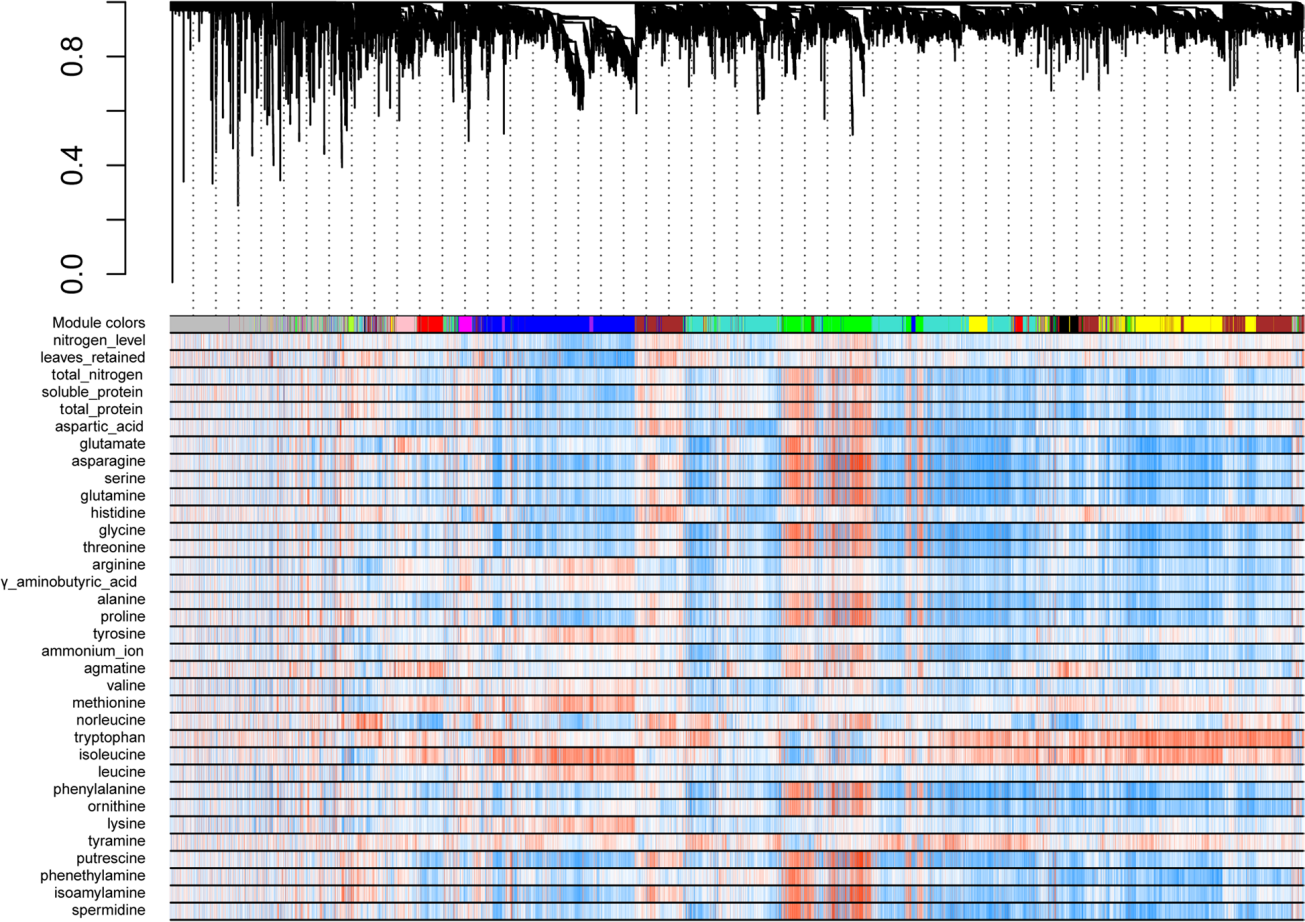
Cluster dendrogram of WGCNA results

In order to further clarify the regulatory mechanism underlying tobacco responses to various N doses and to the number of leaves retained, we integrated the data from 32 metabolites and 13,330 DEGs as above results from RNA-seq and metabolomics into the construction of co-expression networks by using the weighted gene co-expression network analysis (WGCNA) package in R (Fig. 3).

**Fig. 4:**
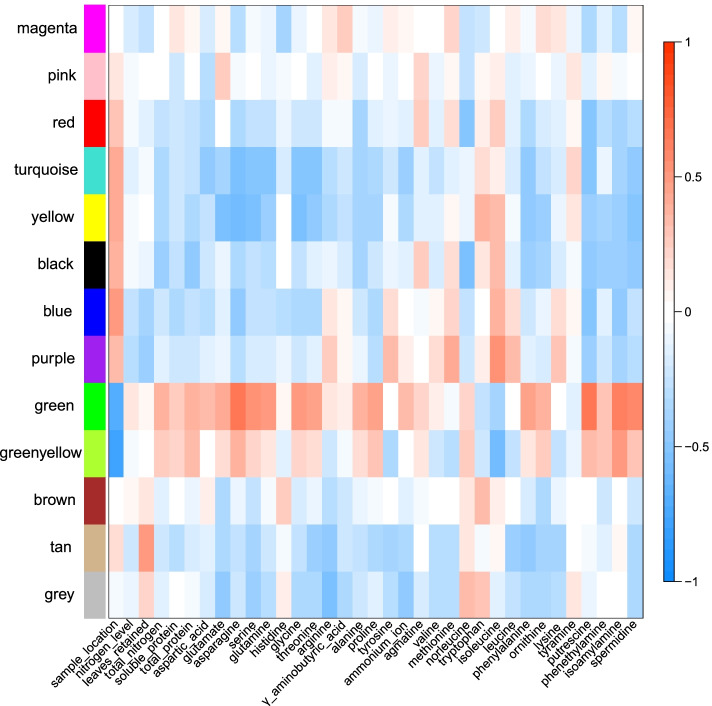
Relationship between 13 co-expression modules and different metabolic traits as determined by WGCNA

Under the given code and running procedures of WGCNA, 13,330 unique DEGs were divided into 13 gene modules with different colors. All trait correlations corresponding to their respective most and least relevant modules are shown in Fig. 4, and Supplementary Tables 6, 7. The numbers of genes in each module were 542, 3332, 3123, 2215, 148, 265, 416, 149, 728, 56, 4281 and 2779 for the black, blue, brown, green, greenyellow, magenta, pink, purple, red, tan, turquoise and yellow colours, respectively. In addition, 3668 genes were not assigned to any modules and were grouped into the grey module.

**Fig. 5:**
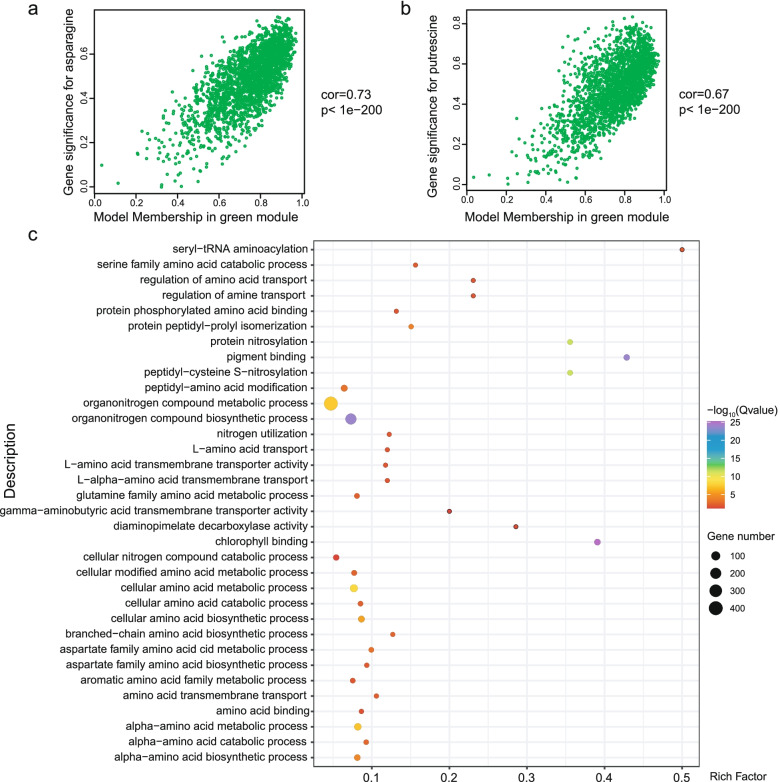
Analysis of the WGCNA green module. **a**. Scatterplot of gene significance for asparagine vs. module membership in the green module. **b**. Scatterplot of gene significance for putrescine vs. module membership in the green module. **c**. GO enrichment analysis results for genes belonging to the green module

The module with the strongest positive correlation to metabolites was the green module (with 2216 genes), such as asparagine (*r* = 0.65, *P* = 5e-05) and putrescine (*r* = 0.64, *P* = 7e-05) (Fig. 5a, b). We turned to GO enrichment analysis to identify processes that were significantly enriched in this module. Genes belonging to the green module were principally involved in amino acid metabolism, such as cellular amino acid metabolic process (GO: 0006520), aspartate family amino acid metabolic process (GO: 0009066), glutamine family amino acid metabolic process (GO: 0009064), and serine family amino acid catabolic process (GO: 0009071) (Fig. 5c, Supplementary Table 8). We also identified *GLUTAMINE SYNTHETASE2* (*Nitab4.5_0000059g0010.1*, *NtGS2*), *ASPARAGINE SYNTHETASE2* (*Nitab4.5_0000102g0010.1*, *NtASN2*), *SERINE/THREONINE SOLUBLE PROTEIN KINASE2* (*Nitab4.5_0000171g0330.1*, *NtS6K2*), *PROLINE-RICH SOLUBLE PROTEIN2* (*Nitab4.5_0000283g0120.1*, *NtPRP2*) and other important amino acid-related genes in the green module. This analysis demonstrated that the difference between N dose and the number of leaves retained affects amino acid biosynthesis in tobacco plants. Notably, we determined that chlorophyll binding (GO: 0016168), photosynthesis, light harvesting (GO: 0009765), photosynthesis (GO: 0015979) and carbon fixation (GO: 0015977), generation of precursor metabolites and energy (GO: 0006091) pathways were also enriched (Supplementary Table 8), suggesting that plant photosynthesis and energy metabolism may play a role in N assimilation into amino acids.

**Fig. 6:**
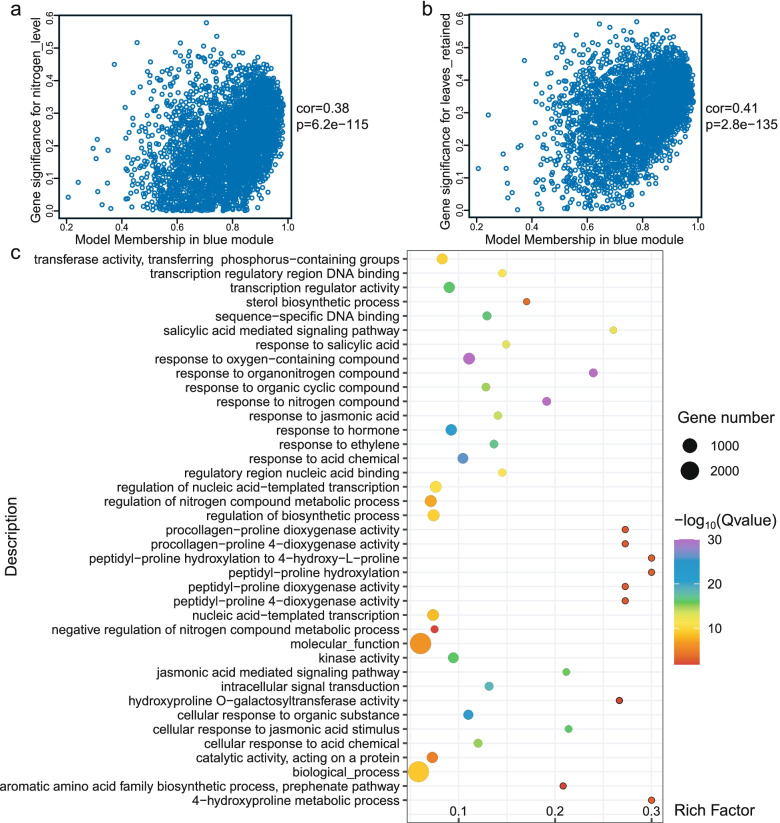
Analysis of the WGCNA blue module. **a**. Scatterplot of gene significance for nitrogen level vs. module membership in the blue module. **b**. Scatterplot of gene significance for leaves retained vs. module membership in the blue module. **c**. GO enrichment analysis results for genes in the blue module

Relationships between module eigengenes revealed that the green module had the highest correlation with the blue modules (*r* = 0.75) (Supplementary Fig. 4), it has a positive correlation with N dose and the number of leaves retained (Fig. 6a, b). By contrast, genes in the blue module (3332 genes) showed a negative correlation with most metabolic substances (Fig. 4, Supplementary Table 6, 7). GO enrichment analysis for the blue module revealed an over-representation of nitrogen compound metabolic process (GO: 0051171), response to hormone (GO: 0009725), especially JA (GO: 0009753), salicylic acid (SA) (GO: 0009751), and ethylene (GO: 0009723), as well as genes involved in aromatic amino acid family biosynthetic process (GO: 0009095) (Fig. 6c, Supplementary Table 8). We identified several classical N marker genes in the blue module, such as nitrate transporter gene *Nitab4.5_0000785g0250.1* (*NtNRT1-2a*) and *Nitab4.5_0004605g0120.1* (*NtNRT1-2b*) [29]. This observation indicates that the blue module is highly related to N metabolism, and that phytohormones may influence N transport and metabolism in tobacco.

### Role of key genes responsive to N application and number of leaves retained

**Fig. 7:**
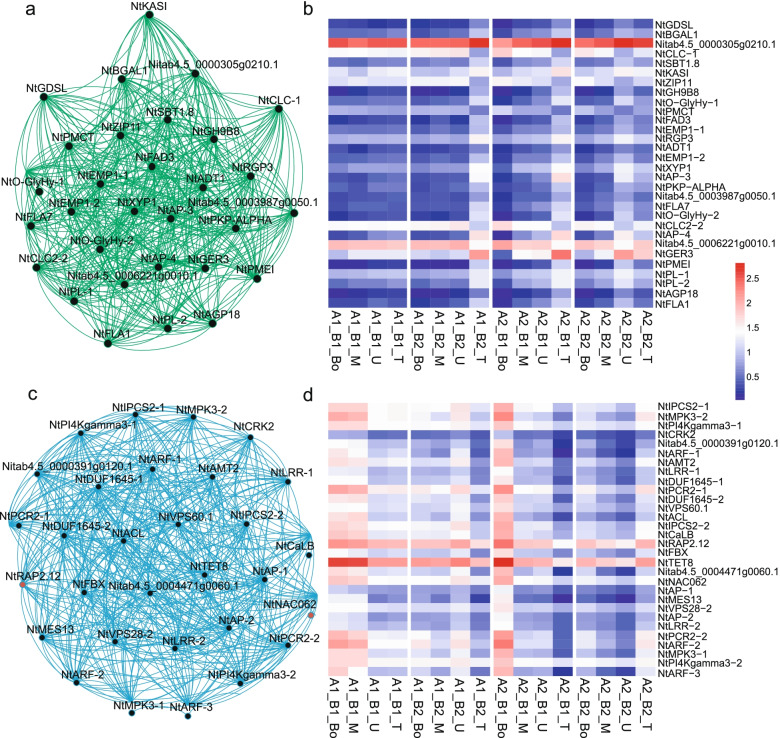
Construction of networks from the green and blue modules. **a**. Green module network. **b**. Heatmap representation of gene expression in the green module network. **c**. Blue module network. Pink circles represent TFs. **d**. Heatmap representation of gene expression in the blue module network. Gene expression values in the heatmaps were normalized to log_2_(FPKM+ 1)

We selected the top 30 significant genes from the blue and green modules (Supplementary Table 9) with a threshold value was less than 0.01 as determined by WGCNA and constructed their corresponding networks, using their FPKM values after log_2_(FPKM + 1) transformation across all samples (Fig. 7).

The green module network consisted of 30 nodes and 476 edges (Fig. 7a). The selected genes were highly expressed in the upper leaves, as shown by the heatmap representation (Fig. 7b). Since our analysis had indicated that metabolites were most highly correlated with the green module (Fig. 4), we selected the *ASPARTYL PROTEASE* genes *Nitab4.5_0003481g0010.1* (*NtAP-3*) and *Nitab4.5_0005808g0080.1* (*NtAP-4*). *NtAP-3* and *NtAP-4* were more highly expressed under high N conditions (A2) independently of the number of leaves retained (Fig. 7b). Within the green module network, we identified a gene with unknown function gene (*Nitab4.5_0000305g0210.1*, whose Arabidopsis ortholog is *At4g01150*) and a high expression level at the higher N dose and with more leaves retained (Fig. 7b). Notably, this gene is associated with the GO terms photosynthetic membrane (GO: 0034357) and chloroplast envelope (GO: 0009941) (Supplementary Table 8), which agrees with our previous results. A thorough characterization of *Nitab4.5_0000305g0210.1* may help us discover new connections between photosynthesis and N metabolism in plants.

We observed high expression for *GERMIN3* gene (*Nitab4.5_0007255g0060.1*, *NtGER3*) under higher N dose and with 16 leaves retained (Fig. 7b), *NtGER3* is mainly enriched in response to cytokinin (CK) stimulus (GO: 0009735), peptidyl-cysteine modification (GO: 0018198), peptidyl-cysteine S-nitrosylation (GO: 0018119) and protein amino acid nitrosylation (GO: 0017014) (Supplementary Table 8), suggesting that *NtGER3* may play an important role in amino acid metabolism through CK pathway. In addition, the carbohydrate and fatty acid metabolism-related genes *Nitab4.5_0000255g0150.1* (*BETA GALACTOSIDASE1*, *NtBGAL1*), *Nitab4.5_0000348g0200.1* (*3-KETOACYL-ACYL CARRIER PROTEIN SYNTHASE1*, *NtKAS1*), *Nitab4.5_0000078g0290.1* (*ESTERASE/LIPASE*, *NtGDSL*) and *Nitab4.5_0001881g0020.1* (*FATTY ACID DESATURASE3*, *NtFAD3*) are up-regulated in response to N dose and leaf number retained in green module, suggesting these genes might be key players in the plant energy regulation and development through carbon/nitrogen (C/N) balance.

In the blue module network, we identified the N marker gene *AMMONIUM TRANSPORTER2* (*NtAMT2*, *Nitab4.5_0000444g0190.1*) and two TFs (belonging to the RAP and NAC families) out of 30 nodes and 476 edges (Fig. 7c). In contrast to the green module network, a heatmap representation of gene expression from genes that belong to the blue module indicated that they were highly expressed in the bottom leaves, especially with the lower number of leaves retained (Fig. 7d). The gene *Nitab4.5_0002716g0070.1* (*RELATED TO AP2 12*, *NtRAP2.12*) belongs to the *ETHYLENE RESPONSE FACTOR (ERF)/APETALA2 (AP2)* TF family and *NtRAP2.12* is involved in ethylene-mediated signaling (GO: 0009873), regulation of nitrogen compound metabolic process (GO: 0051171) and regulation of primary metabolic process (GO: 0080090) (Supplementary Table 8), suggesting *NtRAP2.12* may control both phytohormone and N regulation. Genes within this module showed high expression levels at almost all leaf positions collected and both N doses, especially at high N application and fewer leaves retained (Fig. 7d). Aside from *NtRAP2.12*, we noticed three *AUXIN-RESPONSE FACTOR* (*ARF*) genes in the blue module network: *Nitab4.5_0000441g0240.1* (*NtARF-1*), *Nitab4.5_0011106g0010.1* (*NtARF-2*) and *Nitab4.5_0000019g0400.1* (*NtARF-3*). This result suggests that phytohormones may play an important role in N regulation and that *NtRAP2.12* may be a critical regulatory node. We also determined that *Nitab4.5_0003289g0030.1* (*TETRASPANIN8*, *NtTET8*) shared a highly similar expression pattern with *NtRAP2.12* (Fig. 7d).

The other TF in the blue module network was *Nitab4.5_0004654g0040.1* (*NtNAC062*) and was associated with response to carbohydrate stimulus (GO: 0009743), regulation of biosynthetic process (GO: 0009899) and regulation of N compound metabolic process (GO: 0051171) (Supplementary Table 8). We speculate that *NtNAC062* plays an important role in regulating the C/N balance in tobacco (Fig. 7d).

### qRT-PCR validation

qRT-PCR was used to detect and verify the relative expression levels of ten key DEGs identified by RNA-seq, these two results were calculated the Pearson correlation coefficients and used log_2_ fold changes at all sample stages (Supplementary Table 9). We obtained a correlation coefficient between RNA-seq and qRT-PCR of the ten genes were 0.86 (Supplementary Fig. 5), confirming the reliability and accuracy of the RNA-seq data. Although the results of the two analytical methods are different, it may be related to the detection range and sensitivity of the two methods.

## Discussion

In our study, we determined the contents of 32 metabolites in tobacco leaves collected at four distinct positions, from plants exposed to two N fertilizer doses and two different leaf numbers remaining after topping. We also generated RNA-seq data for the same samples and identified 13,330 DEGs used as input for GO enrichment and WGCNA. We showed that N application and the number of leaves retained after topping affects the development and metabolism of tobacco plants at the molecular level, and identified several candidate genes that may participate in the regulation process.

**Fig. 8:**
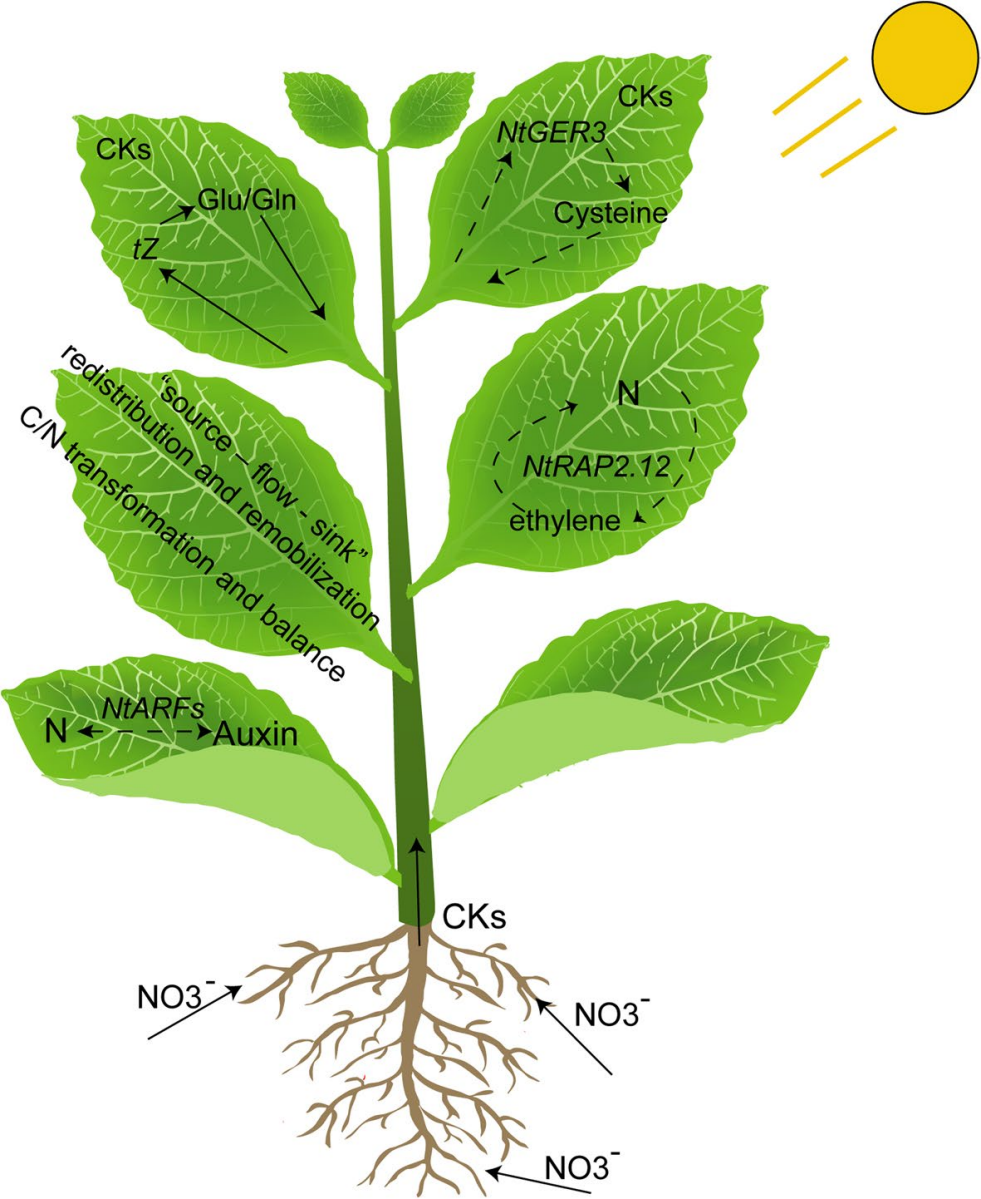
Proposed a regulatory model in tobacco leaves

Phytohormones play an important role in N regulation and leaf number retained. CK metabolism and signaling are closely related to N availability and regulates N uptake [14], the interaction between CKs and N have been studied widely [30–35]. Recently studies show that through shoot transcriptome analysis of the CK biosynthesis or translocation mutants defective under homogenous or heterogeneous nitrate supply, glutamate and glutamine related genes’ biosynthesis are identified as potential targets of *tZ* regulation [16], *AtGER3* highly related to CK in Arabidopsis through proteomic and metabolomic [35]. Across to our WGCNA results, we identified *NtGER3* as a hub gene in green module with high N dose (A2) and 16 leaves retained (B2) is mainly enriched in response to CK stimulus, protein amino acid nitrosylation and peptidyl-cysteine S-nitrosylation by GO analysis, we strongly speculate that *NtGER3* may play an important role in N metabolism related to amino acid (cysteine) through CK pathway in tobacco leaves (Fig. 8).

Our RNA-seq results shows amino acid metabolism is more active in the top and upper part of the plant, different leaf number retained after topping had little effect on the middle and bottom leaf positions, but had strong effects in top and upper leaves. The blue module we identified in WGCNA was positive correlation with N dose and number of leaves retained but negative correlation with metabolic substance. And we identified several *ARFs* in blue module highly expression in bottom leaves. Previous studies NRT1.1/CHL1 is a dual affinity nitrate transporter and nitrate sensor that links nitrate and auxin [11]. The auxin receptor gene *AUXIN SIGNALING F-BOX3* (*AFB3*) is induced by nitrate application, *afb3* mutant shows no change in primary or lateral root growth in response to nitrate, demonstrating that nitrate regulates root architecture through AFB3 by modulating the auxin signal [36], Here we speculate *ARFs* may participated in modulating the auxin signal and N in bottom leaves. Apart to *ARFs*, we also identified the ethylene-related TF gene *NtRAP2.12* in the blue module, which highly expression in low N dose (A1) and less leaf number retained (B1). *AtRAP2.12* connects three gaseous signals (nitric oxide [NO], O_2_, and ethylene), is regulated by the N-degron pathway, and mediates adaptation to flooding-induced hypoxia [37]. In addition, *RAP2.12* stabilization regulates respiration, the TCA cycle, and amino acid metabolism [38]. We speculated *NtRAP2.12* may involve in N regulation through ethylene pathway. Ethylene plays an important regulatory roles in plant responses to mineral nutrients availability, especially N [39]. Plant leaves activate a N-recycling system in which N is recycled from phenylalanine by means of deamination to cinnamic acid under the conditions of N deficiency or starvation and this futile cycle is under the control of ethylene [40]. As stated in our results, genes related to cell wall biosynthesis and photosynthesis were significantly down-regulated at lower N doses, conserving energy for new leaf formation and development.

To summarize our results above, leaves at the bottom and middle positions along the main stem competed for N resources: N preferentially supplied the bottom leaves closer to the root, while middle leaves received more N under lower N application for better photosynthesis. It’s also mentioned enhancing source-to-sink nitrate remobilization represents a new strategy for enhancing NUE and crop production [41], researchers found under N starvation, the *nrt1.7* mutant exhibits growth retardation and *NRT1.7*-mediated source-to-sink remobilization of stored nitrate is important for sustaining growth in plants. We thought N application and leaf number retained may influence “source – flow – sink” redistribution and remobilization in tobacco plants, also involve some energy transformation. Much of the leaf N content is associated with photosynthetic proteins of the Calvin cycle and the structural components of thylakoids, hinting at the influence of N supply on photosynthetic capacity [42]. In the flag leaf of wheat, chlorophyll content and RuBisCO activity were approximately proportional to leaf N content [43]. Currently, substantial numbers of studies have explored the effects of N on plants and the importance of maintaining a C/N balance by combining photorespiration and photosynthesis, the TCA cycle being a critical central hub to their regulation [44–47]. Our results identified several carbohydrate and fatty acid metabolism-related genes are up-regulated in response to N dose and leaf number retained in green module, implied that the C/N transformation and balance affect the “source – flow – sink” redistribution and remobilization in tobacco during growth and development process, which requires us to further investigate.

## Conclusions

In summary, with the help of powerful multi-omics technology, and combined DEGs analysis, GO enrichment analysis and co-expression network constructed results, we identified 13,330 unique differentially expressed genes through RNA-seq and identified 32 metabolites metabolome analysis from four positions under two different nitrogen doses and with different numbers of leaves retained after topping in tobacco. We identified two important regulatory networks involved in nitrogen compounds and nitrogen metabolism, and combined with gene expression data, we predicted hub gene *NtGER3* may play an important role in N metabolism related to amino acid (cysteine) through CK pathway in tobacco leaves, *NtARFs* may participated in modulating the auxin signal and N in bottom leaves and *NtRAP2.12* as key gene involved in N regulation by ethylene pathway. Our data also discussed C/N transformation and balance affect the “source – flow - sink” redistribution and remobilization in tobacco during growth and development process. The results provide a new insight into the complex molecular mechanism of the regulatory network controlling by different nitrogen application and different numbers of leaves retained after topping in tobacco plants.

## Methods

### Plant materials, rowing conditions and treatments

We used the tobacco (*Nicotiana tabacum*) cultivar K326 in this study, which was kindly provided by the Guizhou Tobacco Research Institute, Guiyang, China. In order to analyze the effects of N applications and remaining leaves after topping on the dynamic development of tobacco plants, we applied exogenous N at two doses: A1 (pure N 3 kg/667 m^2^) and A2 (pure N 6 kg/667 m^2^), and it’s applied twice: the first was applied before transplanting and the second was applied 35 days after transplanting. We topped tobacco plants at 55 days after transplanting, sampled the bottom (Bo), middle (M), upper (U), and top (T) for each treated plant (Supplementary Fig. 1). The first sampling is 2 days after the topping, and the second sampling is 17 days after the topping, leaving two distinct numbers of remaining leaves per plant: B1 (12 leaves/plant) and B2 (16 leaves/plant). Plants were subjected to these four factors (A1, A2, B1, B2) in a split-plot experiment design [48], each plot with 60 tobacco plants with a row spacing of 0.6 m and plant spacing of 1.1 m. All tobacco plants were grown in the field (106.42 °E, 26.35 °N) and management measures were carried out according to local tobacco cultivation techniques [49]. Three biological replicates for each of the samples were immediately frozen in liquid nitrogen and stored at − 80 °C until further use.

### Measurement of metabolic compounds

We measured 32 metabolites including total nitrogen, total protein, soluble protein, 21 amino acids, polyamines (PAs), putrescine, spermidine, tyramine, agmatine, phenethylamine, isoamylamine, and ammonium ion, ϒ aminobutyric acid, at the Bo, M, U, and T positions along the main stem in two biological replicates in tobacco plants. We followed the tobacco industry standards of the People’s Republic of China for protein determination by the continuous-flow method (YC/T 249–2008) to extract protein for subsequent content determination and soluble protein content using a commercial assay kit (Coomassie Brilliant Blue G-250, Nanjing Jiancheng Bioengineering Institute, Nanjing, China) according to the manufacturer’s instructions. The 21 free amino acid contents were measured by using on thin layer chromatography plates (TLC) as described previously [50]. We measured PAs content (isoamylamine, spermidine, phenethylamine, and putrescine) in tobacco leaves as described previously [51].

### Total RNA extraction and RNA sequencing

Total RNA was extracted from the leaves using TRIzol reagent (Invitrogen, Carlsbad, CA, USA) and purified using RNeasy Plant Mini Kit (Qiagen, Valencia, CA, USA) according to the manufacturer’s instructions. Using a NanoDrop spectrophotometer (Thermo Fisher Scientific, Inc.) quantified the RNA and the purity of the total RNA was detected by measuring both the A260/280 and A260/230 before use for deep sequencing of the transcriptome (RNA-seq) and reverse transcription followed by quantitative real-time polymerase chain reaction (qRT-PCR) analysis.

Then cDNA libraries were constructed form tobacco leaves for sequencing. In brief, poly-A mRNA was purified from the total RNA using poly-T oligo-attached magnetic beads firstly, then the purified poly-A mRNA was fragmented into smaller fragments and were used as templates for the synthesis of first-strand cDNA with SuperScript II reverse transcriptase (Promega) and hexamer primers. Then DNA polymerase I, RNase H, DNA synthesis buffer and dNTPs with AMPure XP beads were used to synthesize the second-strand cDNA. The cDNA fragments were then purified, end-repaired, A-tailed with the MinElute PCR Purification Kit (Qiagen, Germany). The raw sequencing data are deposited in the BIG Data Center (https://bigd.big.ac.cn/) under BioProject accession number PRJCA003512.

### Bioinformatic analysis

Quality-control analysis of each RNA-seq raw reads was performed using FastQC software (https://www.bioinformatics.babraham.ac.uk/projects/fastqc/). The low-quality reads and trimmed adaptors in the raw reads were removed with NGSQCTookit (v2.3.3) [52]. We mapped all cleaned reads to the *Nicotiana tabacum* reference genome (Nitab v4.5) (https://solgenomics.net/organism/Nicotiana_tabacum/genome) by using Salmon (v0.8.2) with default parameters. We calculated gene expression estimates as fragments per kilobase of exon model per million mapped reads (FPKM) values with the Cufflink software package [52] and used the FeatureCounts [53] option to calculate non-normalized read counts per gene. Differentially expressed genes (DEGs) were identified using raw gene counts as input and calculated using the DESeq2 package [54] with the following requirements: false discovery rate (FDR) < 0.05 and absolute log_2_(fold change) ≥2 [55].

Knowledge about DEGs biological function is crucial for our next step analysis, therefore, we annotated tobacco proteins by performing Basic Local Alignment Sequence Tool for Proteins (BLASTP) searches against Arabidopsis proteins from the Araport11 release with a minimum *e*-value cut-off of 10^− 5^ [56, 57], according to the blast results, the Arabidopsis protein with the highest homology was used as the tobacco corresponding protein for Gene Ontology (GO) pathway annotation. Significant enrichment analysis and statistical tests using the topGO package [58] in R; we visualized GO results using the R package ggplot2.

### Weighted correlation network analysis (WGCNA)

We implemented the R package WGCNA [59, 60] to construct co-expression networks and identify hub genes within important networks, and it’s an open source package at https://horvath.genetics.ucla.edu/html/CoexpressionNetwork/Rpackages/WGCNA/.

Briefly, we used genes expression data based on the log_2_(FPKM+ 1) from RNA-seq as “expression matrix”, and metabolomics data as “phenotypic traits”, the co-expression network is constructed according to the correlation degree of these two sets of data. The soft threshold power was calculated by the pickSoftThreshold function, and our thresholding power is 18. Then, connectivity, module eigengene, intramodular connectivity, topological overlap matrix (TOM), module membership and hub gene were calculated and performed by established code. The co-expression networks were presented using Cytoscape v3.5.1 [61].

### Network analysis of transcription factors (TFs) and genes

To identify transcription regulatory networks in target co-expression modules, we selected the top 30 most significant genes in each module with a threshold value < 0.01. We identified TFs using PlantTFDB (http://planttfdb.gao-lab.org). The resulting co-expression networks were generated and visualized using Cytoscape v3.5.1 [61]. Heatmaps were created using modified expression values by the formula log_2_(FPKM + 1).

### Validation of RNA-seq by qRT-PCR

In order to verify the reliability of RNA-seq results, qRT-PCR was used to detect 10 DEGs identified from our RNA-seq analysis as previously described [25], and *NtEF-1α* as an internal control for data normalization [62]. We designed all 11 gene-specific primers using the qPrimerDB Database (https://biodb.swu.edu.cn/qprimerdb) [63]. Primer sequences are listed in Supplementary Table 1. Relative transcript levels were calculated as 2^*−ΔΔCT*^ [64]. Three independent biological replicates were analyzed per sample.

### Statistical analysis

We performed all statistical analyses in SPSS V16.0 for Windows (SPSS, Chicago, Illinois, USA). We considered a difference to be statistically significant if *P* < 0.05. We performed one-way Analysis of variance (ANOVA) on data from experiment with three replicates [65]. We applied the Turkey test for multiple pairwise tests for significance.

## Supplementary Information

**Additional file 1: Supplementary Figure 1**. Material sampling diagram. **Supplementary Figure 2**. The correlation coefficients for metabolites. **Supplementary Figure 3**. Changes in major nitrogen compounds at four level and four positions. Values are the means ± standard error (SE) of three biological replicates. Different letters indicate a significant difference (Tukey’s multiple comparison test, *P* < 0.05). **Supplementary Figure 4**. Relationship between module eigengenes in WGCNA. **Supplementary Figure 5**. Comparison of gene expression values obtained by qRT-PCR and RNA-seq. Log2(fold change) was calculated for ten genes in different positions of tobacco plant, and r2 = 0.8573 correlation was observed between the results derived from the two methods. **Supplementary Table 1**. Primers used in qRT-PCR. **Supplementary Table 2**. Metabolome data at each sampling stage. **Supplementary Table 3**. RNA-seq read mapping to the *Nicotiana tabacum* reference genome. **Supplementary Table 4**. The number of DEGs in tobacco leaves. **Supplementary Table 5**. GO enrichment analysis of DEGs. **Supplementary Table 6**. Overview of modules and their corresponding traits determined by WGCNA. **Supplementary Table 7**. Overview of module trait *p*-values determined by WGCNA. **Supplementary Table 8**. GO enrichment analysis of WGCNA module genes. **Supplementary Table 9**. FPKM of blue and green network genes.

## Data Availability

All raw reads of transcriptome data have been uploaded to the BioProject PRJCA003512.

## Abbreviations

TF: Transcription factor
NO_3_^−^: nitrate
NH_4_^+^: ammonium
NRT1: NITRATE TRANSPORTER 1
NPF: PEPTIDE TRANSPORTER (PTR) Family
CLC: CHLORIDE CHANNEL
SLAC: SLOWLY ACTIVATING ANION CHANNEL
NLP7: NIN-LIKE PROTEIN7
AP: ASPARTYL PROTEASE
IPT: PHOSPHATE-ISOPENTENYLTRANSFERASE
AMTs: Ammonium transporters
GOGAT: glutamine-2-oxoglutarate aminotransferase
GS: Glutamine synthetase
RuBisCO: RuBP carboxylase/oxygenase
AAP1: AMINO ACID PERMEASE1
NAC: NAM, ATAF1/2 and CUC2
IAA: indole-3-acetic acid
Bo: Bottom
M: Middle
U: Upper
T: Top
CK: Cytokinin
GA: Gibberellin
SA: Salicylic acid
JA: Jasmonic acid
ABA: Abscisic acid
DEGs: Differentially expressed genes
WGCNA: Gene co-expression network analysis
C: Carbon
N: Nitrogen
GC/MS: gas chromatography/mass spectrometry
RNA-seq: RNA sequencing
qRT-PCR: quantitative real-time polymerase chain reaction
FPKM: Fragments per kilobase of exon model per million mapped reads
FDR: False discovery rate
GO: Gene Ontology
TOM: Topological overlap matrix
TCA: Tricarboxylic acid
ANOVA: Analyses of variance
BLASTP: Basic Local Alignment Sequence Tool for Protein
PAs: Polyamines
NO: Nitric oxide
