# Supplementary material for: Nitrogen application and differences in leaf number retained after topping affect the tobacco (Nicotiana tabacum) transcriptome and metabolome
Source: BMC Plant Biol. 2022 Jan 19;22:38. doi: 10.1186/s12870-022-03426-x (PMC8767696; doi:10.1186/s12870-022-03426-x)
Supplement: Supplementary file 1 — Additional file 1: Supplementary Figure 1. Material sampling diagram. Supplementary Figure 2. The correlation coefficients for metabolites. Supplementary Figure 3. Changes in major nitrogen compounds at four level and four positions. Values are the means ± standard error (SE) of three biological replicates. Different letters indicate a significant difference (Tukey’s multiple comparison test, P < 0.05). Supplementary Figure 4. Relationship between module eigengenes in WGCNA. Supplementary Figure 5. Comparison of gene expression values obtained by qRT-PCR and RNA-seq. Log2(fold change) was calculated for ten genes in different positions of tobacco plant, and r2 = 0.8573 correlation was observed between the results derived from the two methods. Supplementary Table 1. Primers used in qRT-PCR. Supplementary Table 2. Metabolome data at each sampling stage. Supplementary Table 3. RNA-seq read mapping to the Nicotiana tabacum reference genome. Supplementary Table 4. The number of DEGs in tobacco leaves. Supplementary Table 5. GO enrichment analysis of DEGs. Supplementary Table 6. Overview of modules and their corresponding traits determined by WGCNA. Supplementary Table 7. Overview of module trait p-values determined by WGCNA. Supplementary Table 8. GO enrichment analysis of WGCNA module genes. Supplementary Table 9. FPKM of blue and green network genes. [file 12870_2022_3426_MOESM1_ESM.pdf]

# Supplementary Material

## Supplementary Figures

Supplementary Figure 1. Material sampling diagram.

Supplementary Figure 2. The correlation coefficients for metabolites.

Supplementary Figure 3. Changes in major nitrogen compounds at four level and four positions. Values are the means ± standard error (SE) of three biological replicates. Different letters indicate a significant difference (Tukey's multiple comparison test, $P < 0.05$).

Supplementary Figure 4. Relationship between module eigengenes in WGCNA.

Supplementary Figure 5. Comparison of gene expression values obtained by qRT-PCR and RNA-seq. $Log_2$(fold change) was calculated for ten genes in different positions of tobacco plant, and $r^2 = 0.8573$ correlation was observed between the results derived from the two methods.

## Supplementary Tables

Supplementary Table 1 Primers used in qRT-PCR.

Supplementary Table 2 Metabolome data at each sampling stage.

Supplementary Table 3 RNA-seq read mapping to the Nicotiana tabacum reference genome.

Supplementary Table 4 The number of DEGs in tobacco leaves.

Supplementary Table 5 GO enrichment analysis of DEGs.

Supplementary Table 6 Overview of modules and their corresponding traits determined by WGCNA.

Supplementary Table 7 Overview of module trait *p*-values determined by WGCNA.

Supplementary Table 8 GO enrichment analysis of WGCNA module genes.

Supplementary Table 9 FPKM of blue and green network genes.

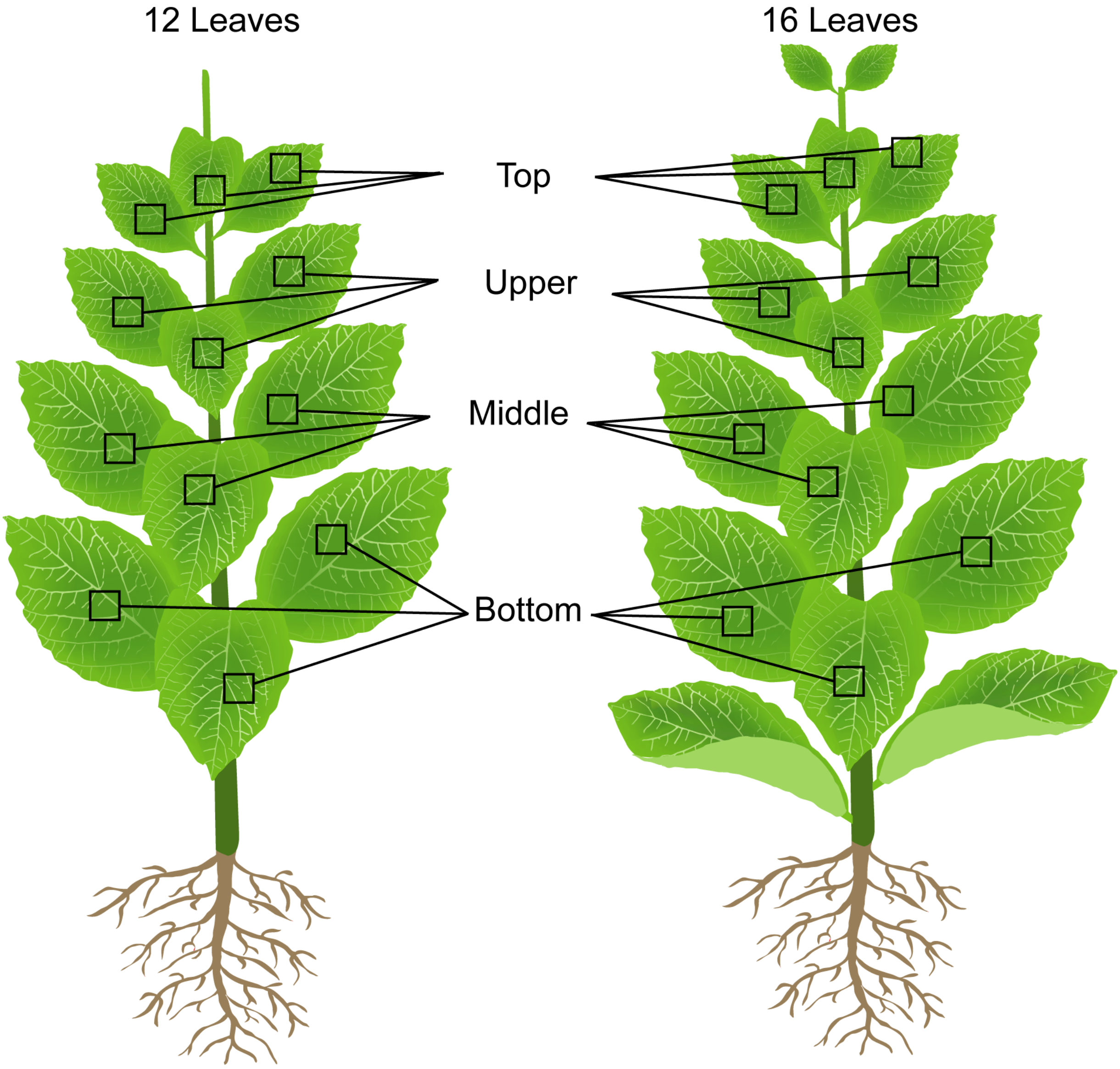


Supplementary Figure 1.

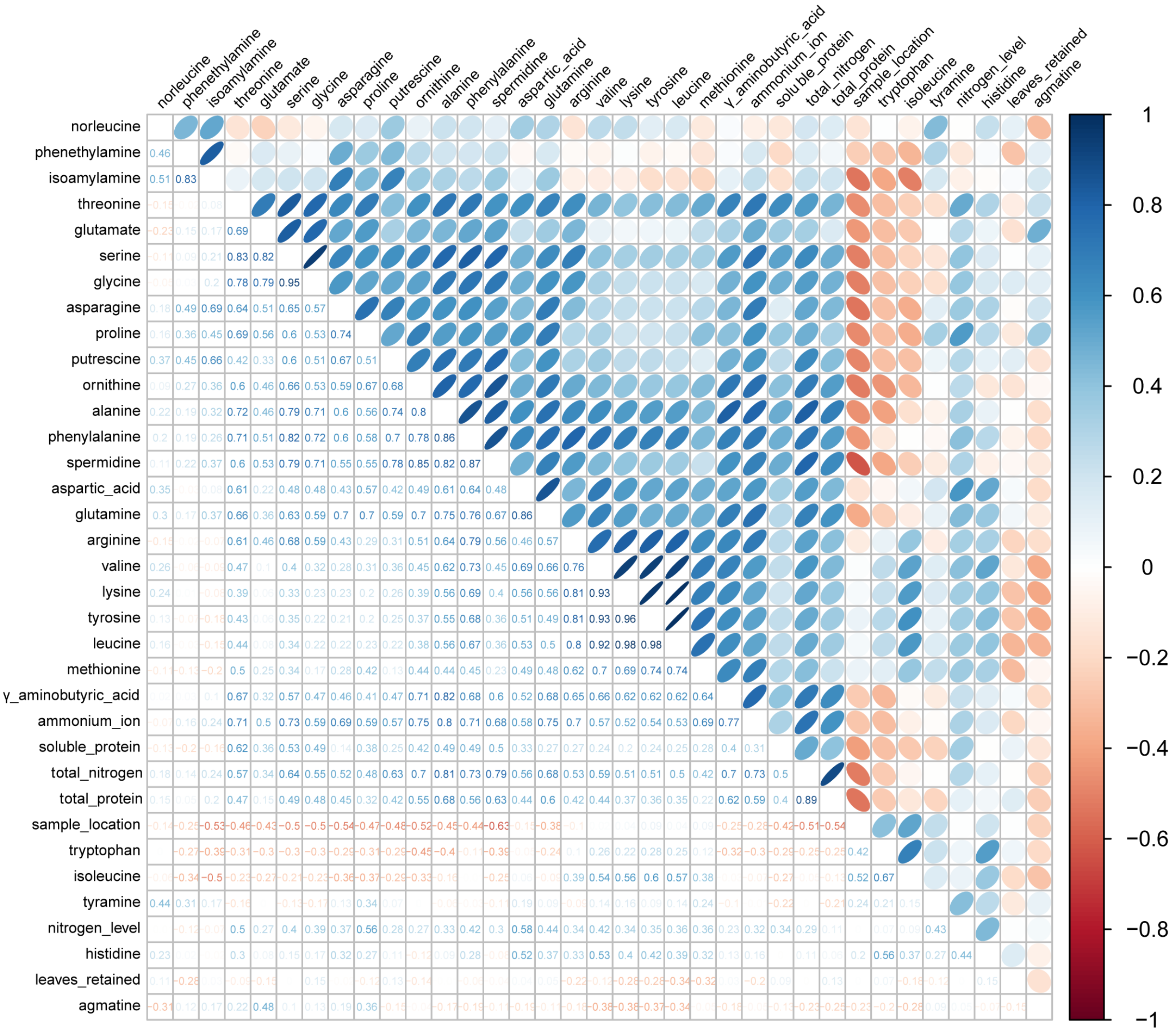


Supplementary Figure 2.

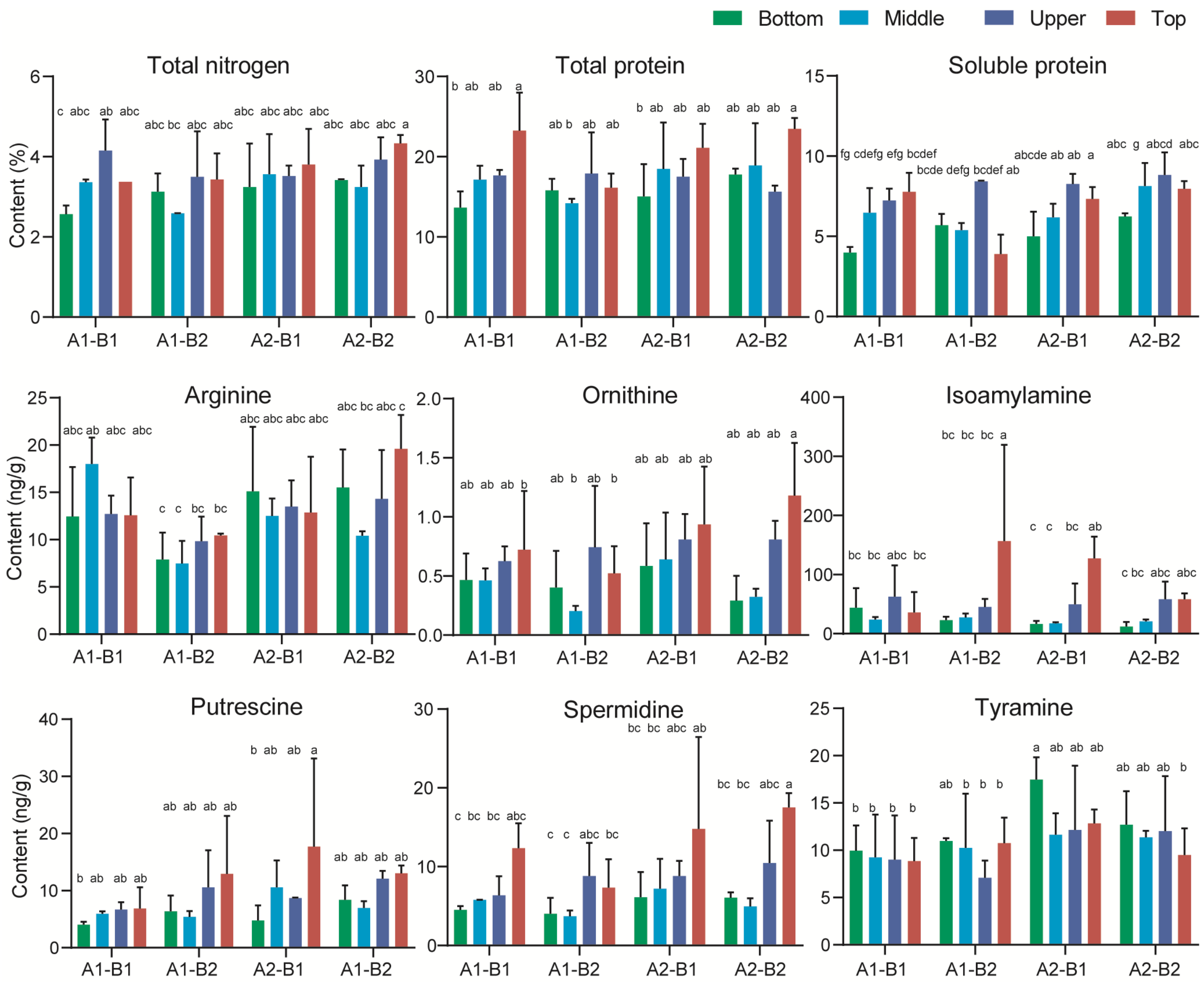


Supplementary Figure 3.

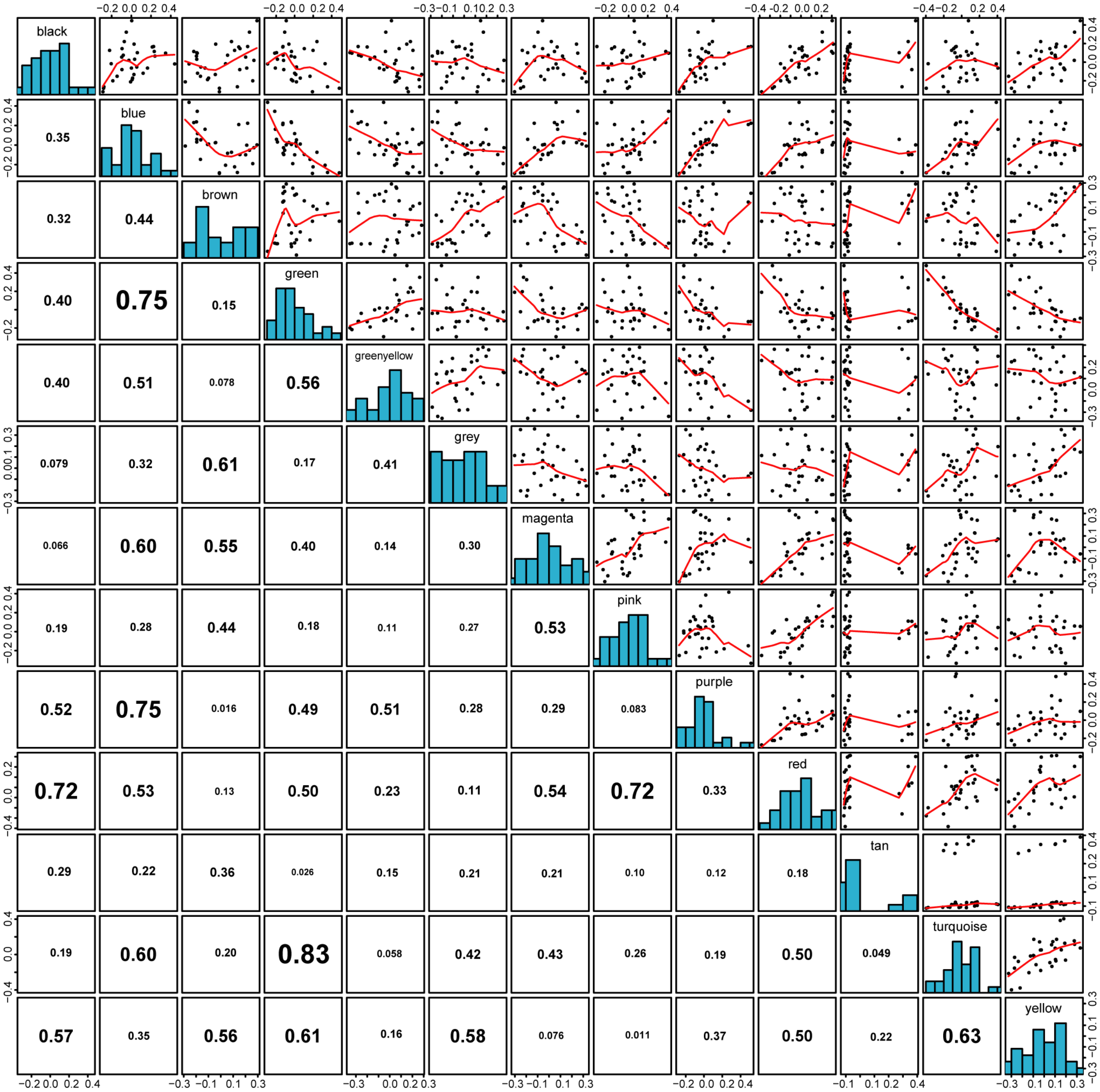


Supplementary Figure 4.

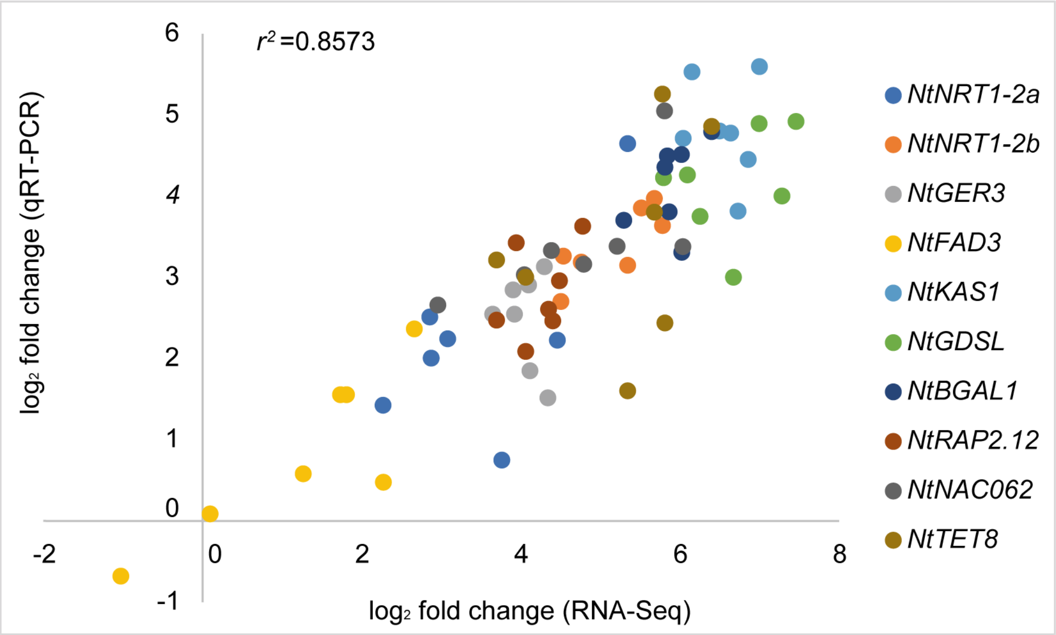


Supplementary Figure 5.

**Supplementary Table 1 Primers used in qRT-PCR**

| Gene name | Gene ID | Forward primer (5'-3') | Reverse primer (5'-3') |
|---|---|---|---|
| *NtNRT1-2a* | Nitab4.5_000 0785g0250.1 | ATAACTGGTAGGTTTCAGCGAA | TGTTGTCATTTTTGCCTCTCTG |
| *NtNRT1-2b* | Nitab4.5_000 4605g0120.1 | TGAATGACCAGAAGTTCATCGA | AAGCAACAGTTCAGCATAACAG |
| *NtGER3* | Nitab4.5_000 7255g0060.1 | GATGCTGGTTACACTTCTGTTG | AGAACACCCTTGAGCTTCTTAA |
| *NtBGAL1* | Nitab4.5_000 0255g0150.1 | CCTTGAATCTTCATTCGCTCAG | GAGCATTGAAAGTGGTCTTGAG |
| *NtKAS1* | Nitab4.5_000 0348g0200.1 | AGCTATTGCAACAGTTAAAGCC | CTGTTTCTTGTTTGCGACAGTA |
| *NtGDSL* | Nitab4.5_000 0078g0290.1 | CCTGGATAATCTACGACGTGAA | GCATGTCCTTGACGATAATGTC |
| *NtFAD3* | Nitab4.5_000 1881g0020.1 | TGAAGACTCTGGACGAAAATCA | GTCGAATTCAAACTCCATCTCG |
| *NtRAP2.12* | Nitab4.5_000 2716g0070.1 | CAAGACGTGTTGTGAAGCTAAA | CTCTTCAACAAAGCTCAACGAA |
| *NtNAC062* | Nitab4.5_000 4654g0040.1 | CAGTGATGAGACACGAGATACA | CTTCAAGGACACATCAACAGTG |
| *NtTET8* | Nitab4.5_000 3289g0030.1 | AAAACCCATAATAGCACTCGGA | TATACCCTTTACCCGAAACCAC |
| *NtEF-1α* | Nitab4.5_000 0022g0070.1 | TGAGATGCACCACGAAGCTC | CCAACATTGTCACCAGGAAGTG |

# Supplementary Table 2 Metabolome data at each sample

| Sample_name | Sample_location | Nitrogen_Application | Remained_Number | Total_nitrogen(%) | Soluble_protein(%) | Total_protein (%) | Aspartic_acid (ng/g) | Glutamate (ng/g) | Asparagine (ng/g) | Serine (ng/g) | Glutamine (ng/g) | Histidine (ng/g) | Glycine (ng/g) | Threonine (ng/g) | Arginine (ng/g) | γ aminobutyric acid (ng/g) | Alanine (ng/g) | Proline (ng/g) | Tyrosine (ng/g) | Ammonium_ion (ng/g) | Agmatine (ng/g) | Valine (ng/g) | Methionine (ng/g) | Norleucine (ng/g) | Tryptophan (ng/g) | Isoleucine (ng/g) | Leucine (ng/g) | Phenylalanine (ng/g) | Ornithine (ng/g) | Lysine (ng/g) | Tyramine (ng/g) | Putrescine (ng/g) | Phenethylamine (ng/g) | Isoamylamine (ng/g) | Spermidine (ng/g) |
|---|---|---|---|---|---|---|---|---|---|---|---|---|---|---|---|---|---|---|---|---|---|---|---|---|---|---|---|---|---|---|---|---|---|---|---|
| A1_B1_Bo_1 | Bottom | A1 | B1 | 2.41 | 4.23 | 12.21 | 48.55 | 222.22 | 8.86 | 21.31 | 21.23 | 4.03 | 6.95 | 22.43 | 8.73 | 72.74 | 25.94 | 30.88 | 7.75 | 7.74 | 6.02 | 10.28 | 4.88 | 0.00 | 7.82 | 7.42 | 20.38 | 18.77 | 0.31 | 11.56 | 11.83 | 3.68 | 2.69 | 67.42 | 4.15 |
| A1_B1_Bo_2 | Bottom | A1 | B1 | 2.72 | 3.73 | 15.07 | 57.62 | 169.91 | 12.08 | 33.01 | 67.62 | 5.47 | 8.67 | 23.05 | 16.15 | 102.26 | 35.29 | 27.47 | 13.72 | 10.38 | 2.57 | 16.63 | 7.26 | 0.00 | 13.53 | 13.52 | 32.52 | 27.88 | 0.62 | 20.57 | 8.05 | 4.40 | 1.62 | 20.45 | 4.85 |
| A1_B1_M_1 | Middle | A1 | B1 | 3.31 | 7.55 | 15.91 | 87.53 | 183.65 | 10.54 | 37.59 | 87.46 | 5.60 | 11.02 | 31.43 | 16.03 | 135.14 | 57.25 | 37.20 | 16.15 | 10.61 | 2.57 | 17.72 | 8.74 | 1.08 | 9.29 | 13.39 | 40.45 | 29.81 | 0.39 | 27.76 | 12.43 | 5.65 | 1.11 | 20.52 | 5.76 |
| A1_B1_M_2 | Middle | A1 | B1 | 3.41 | 5.38 | 18.36 | 99.67 | 157.60 | 14.32 | 33.95 | 80.01 | 5.81 | 8.83 | 28.60 | 19.96 | 113.80 | 51.38 | 26.11 | 15.40 | 14.40 | 2.57 | 17.09 | 7.97 | 0.00 | 12.85 | 14.35 | 37.53 | 31.71 | 0.53 | 24.51 | 6.05 | 6.25 | 1.39 | 26.88 | 5.80 |
| A1_B1_U_1 | Upper | A1 | B1 | 3.37 | 6.72 | 17.19 | 110.63 | 218.50 | 16.34 | 38.46 | 78.72 | 6.31 | 12.10 | 31.09 | 11.31 | 125.19 | 53.07 | 47.00 | 11.00 | 9.06 | 2.57 | 14.80 | 6.55 | 1.43 | 9.04 | 7.97 | 30.50 | 27.99 | 0.54 | 19.42 | 12.30 | 7.60 | 3.80 | 100.09 | 4.66 |
| A1_B1_U_2 | Upper | A1 | B1 | 3.37 | 7.75 | 18.15 | 91.74 | 197.39 | 14.72 | 41.11 | 87.92 | 3.76 | 10.62 | 36.05 | 14.09 | 128.28 | 43.86 | 25.94 | 11.04 | 11.57 | 2.57 | 12.69 | 6.62 | 0.00 | 6.51 | 7.49 | 28.03 | 26.78 | 0.71 | 18.53 | 5.67 | 5.79 | 1.27 | 25.12 | 8.08 |
| A1_B1_T_1 | Top | A1 | B1 | 4.7 | 6.93 | 26.59 | 70.01 | 159.17 | 6.31 | 28.24 | 46.74 | 4.22 | 9.36 | 20.25 | 9.74 | 77.93 | 37.85 | 21.54 | 10.34 | 8.83 | 2.57 | 13.78 | 5.38 | 0.45 | 10.95 | 11.74 | 26.84 | 21.76 | 0.37 | 16.25 | 10.59 | 4.20 | 1.29 | 11.55 | 10.07 |
| A1_B1_T_2 | Top | A1 | B1 | 3.6 | 8.61 | 19.89 | 60.30 | 198.89 | 16.40 | 50.68 | 65.49 | 3.72 | 10.53 | 31.13 | 15.40 | 140.06 | 57.98 | 40.66 | 12.18 | 12.72 | 2.57 | 13.57 | 6.26 | 0.00 | 6.82 | 7.53 | 30.76 | 33.58 | 1.07 | 18.79 | 7.10 | 9.49 | 2.24 | 60.29 | 14.58 |
| A1_B2_Bo_1 | Bottom | A1 | B2 | 2.8 | 6.19 | 14.75 | 83.22 | 179.95 | 3.55 | 15.70 | 31.80 | 5.71 | 8.78 | 20.37 | 5.87 | 77.90 | 33.81 | 17.01 | 7.66 | 6.47 | 2.57 | 11.59 | 4.95 | 0.97 | 10.10 | 9.06 | 18.61 | 17.89 | 0.18 | 10.05 | 11.18 | 4.43 | 1.10 | 18.48 | 2.57 |
| A1_B2_Bo_2 | Bottom | A1 | B2 | 3.45 | 5.20 | 16.82 | 84.17 | 164.35 | 11.16 | 27.67 | 58.06 | 4.73 | 7.87 | 23.07 | 9.91 | 125.04 | 41.87 | 21.04 | 9.60 | 11.04 | 2.57 | 12.91 | 6.11 | 0.00 | 8.39 | 9.99 | 23.46 | 20.16 | 0.62 | 13.54 | 10.77 | 8.34 | 1.44 | 27.13 | 5.44 |
| A1_B2_M_1 | Middle | A1 | B2 | 2.59 | 5.06 | 13.82 | 66.44 | 142.84 | 7.15 | 21.98 | 29.66 | 5.48 | 7.01 | 21.17 | 9.16 | 86.36 | 29.37 | 24.30 | 10.38 | 5.80 | 2.57 | 13.22 | 5.80 | 0.65 | 15.91 | 9.77 | 26.42 | 23.50 | 0.17 | 15.65 | 14.29 | 4.70 | 1.14 | 22.63 | 3.22 |
| A1_B2_M_2 | Middle | A1 | B2 | 2.58 | 5.70 | 14.58 | 65.37 | 129.39 | 7.72 | 19.23 | 36.72 | 3.95 | 7.37 | 19.05 | 5.77 | 72.97 | 30.23 | 17.51 | 6.74 | 7.58 | 2.57 | 9.51 | 5.01 | 0.00 | 7.11 | 7.77 | 15.71 | 16.00 | 0.24 | 7.65 | 6.20 | 6.12 | 0.62 | 32.18 | 4.23 |
| A1_B2_U_1 | Upper | A1 | B2 | 4.3 | 8.46 | 22.57 | 38.71 | 204.01 | 16.36 | 55.00 | 57.12 | 3.44 | 12.08 | 31.73 | 11.67 | 168.90 | 76.61 | 46.69 | 10.68 | 14.40 | 2.57 | 13.34 | 6.95 | 0.00 | 6.29 | 6.20 | 26.13 | 30.47 | 1.11 | 14.70 | 8.37 | 15.16 | 1.58 | 54.97 | 11.79 |
| A1_B2_U_2 | Upper | A1 | B2 | 2.69 | 8.37 | 14.34 | 38.82 | 188.51 | 6.96 | 28.47 | 19.86 | 3.52 | 10.94 | 25.32 | 7.98 | 103.79 | 29.79 | 27.48 | 7.63 | 6.60 | 2.57 | 11.59 | 5.11 | 0.00 | 6.99 | 7.63 | 19.77 | 19.34 | 0.38 | 10.09 | 5.79 | 5.97 | 1.25 | 35.58 | 5.82 |
| A1_B2_T_1 | Top | A1 | B2 | 3.89 | 3.04 | 22.63 | 82.37 | 131.20 | 36.35 | 29.64 | 135.91 | 5.23 | 10.91 | 22.42 | 10.26 | 130.51 | 59.70 | 54.11 | 9.44 | 14.43 | 2.57 | 13.23 | 4.81 | 1.61 | 6.57 | 6.88 | 24.20 | 26.72 | 0.69 | 16.60 | 12.65 | 20.10 | 4.47 | 271.82 | 9.88 |
| A1_B2_T_2 | Top | A1 | B2 | 2.97 | 4.75 | 15.21 | 63.56 | 240.23 | 14.71 | 33.05 | 54.03 | 4.86 | 10.29 | 29.01 | 10.58 | 112.96 | 31.86 | 18.72 | 9.07 | 7.72 | 5.85 | 12.85 | 6.85 | 0.00 | 10.36 | 10.49 | 23.22 | 20.40 | 0.36 | 13.51 | 8.85 | 5.75 | 0.00 | 40.99 | 4.82 |
| A2_B1_Bo_1 | Bottom | A2 | B1 | 2.47 | 3.90 | 12.19 | 66.61 | 144.38 | 7.25 | 19.93 | 31.24 | 3.66 | 6.42 | 17.38 | 10.26 | 76.09 | 35.38 | 13.85 | 9.85 | 5.64 | 2.57 | 12.69 | 4.87 | 0.59 | 7.51 | 10.75 | 26.19 | 19.72 | 0.33 | 16.57 | 15.78 | 2.93 | 1.32 | 12.83 | 3.86 |
| A2_B1_Bo_2 | Bottom | A2 | B1 | 4.01 | 6.08 | 17.89 | 107.83 | 249.39 | 21.75 | 53.42 | 119.50 | 5.15 | 11.72 | 30.51 | 19.93 | 158.83 | 54.48 | 70.46 | 14.66 | 21.73 | 2.57 | 16.72 | 11.27 | 0.00 | 10.15 | 12.31 | 37.57 | 29.18 | 0.84 | 24.93 | 19.14 | 6.64 | 1.70 | 20.08 | 8.37 |
| A2_B1_M_1 | Middle | A2 | B1 | 4.27 | 6.78 | 21.54 | 151.10 | 158.43 | 18.85 | 45.56 | 127.62 | 6.00 | 10.39 | 41.84 | 13.81 | 178.21 | 72.14 | 65.14 | 16.01 | 19.74 | 2.57 | 19.05 | 9.49 | 0.67 | 9.06 | 10.50 | 38.16 | 31.54 | 0.92 | 24.25 | 10.05 | 13.91 | 0.91 | 18.83 | 9.88 |
| A2_B1_M_2 | Middle | A2 | B1 | 2.86 | 5.58 | 14.25 | 81.13 | 211.46 | 14.55 | 29.41 | 48.08 | 6.09 | 10.23 | 28.14 | 11.23 | 91.71 | 32.32 | 29.79 | 9.94 | 8.39 | 2.57 | 12.99 | 6.23 | 0.00 | 12.48 | 10.35 | 25.34 | 21.89 | 0.36 | 14.96 | 13.24 | 7.20 | 0.82 | 15.89 | 4.56 |
| A2_B1_U_1 | Upper | A2 | B1 | 3.33 | 7.82 | 15.89 | 84.09 | 228.79 | 21.00 | 45.98 | 64.72 | 5.64 | 12.30 | 36.86 | 11.53 | 114.29 | 43.64 | 109.19 | 10.33 | 13.56 | 5.63 | 13.39 | 8.69 | 0.00 | 8.88 | 8.31 | 26.25 | 25.58 | 0.96 | 15.21 | 16.93 | 8.61 | 2.46 | 74.36 | 7.43 |
| A2_B1_U_2 | Upper | A2 | B1 | 3.7 | 8.70 | 19.06 | 93.39 | 165.95 | 14.60 | 40.93 | 60.92 | 4.57 | 12.57 | 38.11 | 15.44 | 134.21 | 59.11 | 38.05 | 13.34 | 13.25 | 2.57 | 15.45 | 7.05 | 0.00 | 8.33 | 9.35 | 34.62 | 31.33 | 0.66 | 22.06 | 7.32 | 8.76 | 0.97 | 24.47 | 10.17 |
| A2_B1_T_1 | Top | A2 | B1 | 4.43 | 7.86 | 0.00 | 125.27 | 292.79 | 26.64 | 92.23 | 135.09 | 5.37 | 21.48 | 35.28 | 17.03 | 118.59 | 76.81 | 93.33 | 13.64 | 16.61 | 2.57 | 17.12 | 7.03 | 1.04 | 9.03 | 9.56 | 32.80 | 47.43 | 1.28 | 22.27 | 13.88 | 28.60 | 3.38 | 153.37 | 23.05 |
| A2_B1_T_2 | Top | A2 | B1 | 3.17 | 6.82 | 16.17 | 96.18 | 278.13 | 28.25 | 54.68 | 113.18 | 5.47 | 15.62 | 42.67 | 8.71 | 101.27 | 47.56 | 129.15 | 8.28 | 11.75 | 6.38 | 11.49 | 6.27 | 0.00 | 6.28 | 6.29 | 22.50 | 24.32 | 0.59 | 10.81 | 11.76 | 6.77 | 2.42 | 101.61 | 6.53 |
| A2_B2_Bo_1 | Bottom | A2 | B2 | 3.4 | 6.37 | 17.22 | 125.00 | 136.11 | 6.31 | 23.44 | 95.36 | 5.87 | 9.64 | 27.14 | 12.66 | 132.05 | 56.51 | 36.49 | 13.50 | 7.75 | 2.57 | 17.33 | 6.67 | 0.77 | 10.50 | 13.04 | 33.83 | 27.37 | 0.44 | 22.66 | 15.20 | 10.18 | 1.27 | 17.48 | 6.54 |
| A2_B2_Bo_2 | Bottom | A2 | B2 | 3.43 | 6.09 | 18.30 | 84.65 | 189.07 | 19.50 | 41.31 | 77.71 | 8.57 | 10.71 | 33.02 | 18.35 | 130.92 | 36.71 | 36.00 | 14.28 | 10.24 | 2.57 | 17.00 | 6.72 | 0.00 | 13.75 | 12.90 | 33.07 | 29.94 | 0.14 | 22.66 | 10.21 | 6.60 | 1.49 | 6.73 | 5.58 |
| A2_B2_M_1 | Middle | A2 | B2 | 2.86 | 7.11 | 14.87 | 93.85 | 121.84 | 10.80 | 19.73 | 67.31 | 6.05 | 6.83 | 23.52 | 10.06 | 80.07 | 33.32 | 32.64 | 10.24 | 6.32 | 2.57 | 13.67 | 6.33 | 0.31 | 14.00 | 10.38 | 27.94 | 22.82 | 0.37 | 16.52 | 10.86 | 7.81 | 0.76 | 23.33 | 4.25 |
| A2_B2_M_2 | Middle | A2 | B2 | 3.62 | 9.14 | 17.36 | 85.76 | 169.56 | 7.22 | 30.67 | 48.64 | 5.23 | 9.60 | 26.50 | 10.75 | 110.22 | 40.67 | 31.94 | 12.54 | 6.50 | 2.57 | 14.52 | 6.88 | 0.27 | 10.17 | 11.38 | 30.16 | 25.27 | 0.28 | 18.87 | 11.86 | 6.10 | 1.03 | 18.60 | 5.69 |
| A2_B2_U_1 | Upper | A2 | B2 | 3.53 | 7.81 | 18.98 | 112.89 | 163.30 | 26.66 | 35.15 | 93.51 | 6.10 | 9.44 | 31.18 | 10.66 | 88.53 | 45.49 | 82.20 | 11.86 | 9.42 | 2.57 | 14.82 | 6.40 | 1.37 | 10.49 | 7.41 | 29.04 | 30.80 | 0.70 | 17.45 | 16.13 | 11.11 | 1.81 | 79.42 | 6.64 |
| A2_B2_U_2 | Upper | A2 | B2 | 4.32 | 9.82 | 23.21 | 175.44 | 264.93 | 27.97 | 102.08 | 196.09 | 6.67 | 28.89 | 49.75 | 17.95 | 153.78 | 76.42 | 81.99 | 12.17 | 21.35 | 2.57 | 17.11 | 8.10 | 0.00 | 7.34 | 7.83 | 30.41 | 39.16 | 0.92 | 19.37 | 7.91 | 13.06 | 0.66 | 36.72 | 14.27 |
| A2_B2_T_1 | Top | A2 | B2 | 4.48 | 7.60 | 24.42 | 172.99 | 214.39 | 18.48 | 59.94 | 212.52 | 5.24 | 20.30 | 37.90 | 17.07 | 179.94 | 74.78 | 101.51 | 13.39 | 15.05 | 2.57 | 18.62 | 7.46 | 1.05 | 7.55 | 8.34 | 34.19 | 43.20 | 1.50 | 25.04 | 11.48 | 12.02 | 1.12 | 65.29 | 18.81 |
| A2_B2_T_2 | Top | A2 | B2 | 4.18 | 8.30 | 22.50 | 97.17 | 348.23 | 26.78 | 123.77 | 98.54 | 5.26 | 39.16 | 48.98 | 22.13 | 145.98 | 82.27 | 63.95 | 12.42 | 16.66 | 4.12 | 14.90 | 6.03 | 0.00 | 8.24 | 8.76 | 30.76 | 41.10 | 0.87 | 19.32 | 7.50 | 13.98 | 1.23 | 50.86 | 16.20 |

**Supplementary Table 3 RNA-seq read mapping to reference genome**

| Sample | Row reads number | Clean reads number | Mapped clean reads to genome (%) |
|---|---|---|---|
| A1_B1_Bo_1 | 26,732,516 | 22,216,924 | 83.11% |
| A1_B1_Bo_2 | 34,601,349 | 29,219,871 | 84.45% |
| A1_B1_M_1 | 24,687,607 | 19,982,128 | 80.94% |
| A1_B1_M_2 | 33,211,659 | 27,084,993 | 81.55% |
| A1_B1_U_1 | 30,448,355 | 25,649,565 | 84.24% |
| A1_B1_U_2 | 28,091,039 | 23,556,867 | 83.86% |
| A1_B1_T_1 | 26,098,223 | 21,615,598 | 82.82% |
| A1_B1_T_2 | 31,717,533 | 26,571,039 | 83.77% |
| A1_B2_Bo_1 | 24,092,650 | 19,880,939 | 82.52% |
| A1_B2_Bo_2 | 28,448,699 | 23,472,863 | 82.51% |
| A1_B2_M_1 | 28,102,180 | 22,890,648 | 81.46% |
| A1_B2_M_2 | 34,441,184 | 28,013,487 | 81.34% |
| A1_B2_U_1 | 28,301,946 | 23,593,871 | 83.36% |
| A1_B2_U_2 | 31,373,644 | 26,295,189 | 83.81% |
| A1_B2_T_1 | 23,235,003 | 19,382,921 | 83.42% |
| A1_B2_T_2 | 32,726,789 | 27,375,646 | 83.65% |
| A2_B1_Bo_1 | 26,579,068 | 22,274,216 | 83.80% |
| A2_B1_Bo_2 | 24,677,281 | 20,622,150 | 83.57% |
| A2_B1_M_1 | 38,252,622 | 31,331,767 | 81.91% |
| A2_B1_M_2 | 36,697,622 | 30,251,756 | 82.44% |
| A2_B1_U_1 | 35,799,010 | 29,970,544 | 83.72% |
| A2_B1_U_2 | 31,557,079 | 26,001,485 | 82.40% |
| A2_B1_T_1 | 33,004,129 | 27,527,209 | 83.41% |
| A2_B1_T_2 | 32,305,872 | 26,668,108 | 82.55% |
| A2_B2_Bo_1 | 31,279,615 | 26,070,498 | 83.35% |
| A2_B2_Bo_2 | 35,156,035 | 29,158,797 | 82.94% |
| A2_B2_M_1 | 34,484,256 | 28,216,009 | 81.82% |
| A2_B2_M_2 | 36,102,159 | 29,879,501 | 82.76% |
| A2_B2_U_1 | 32,582,783 | 27,694,686 | 85.00% |
| A2_B2_U_2 | 35,708,823 | 29,681,344 | 83.12% |
| A2_B2_T_1 | 32,308,339 | 26,975,578 | 83.49% |
| A2_B2_T_2 | 34,208,727 | 28,517,855 | 83.36% |
| Overall/Average | 997,013,796 | 827,644,052 | 83.01% |

**Supplementary Table 4 The number of DEGs in tobacco leaves**

| Comparison | Number of up-regulated genes | Number of up-regulated genes | Number of DEGs |
|---|---|---|---|
| B1_Bottom | 528 | 238 | 766 |
| B1-Middle | 732 | 69 | 801 |
| B1_Upper | 152 | 192 | 344 |
| B1_Top | 1187 | 1488 | 2675 |
| B2_Bottom | 2 | 1 | 3 |
| B2-Middle | 161 | 191 | 352 |
| B2_Upper | 203 | 190 | 393 |
| B2_Top | 1093 | 1133 | 2226 |
| A1_Bottom | 2 | 20 | 22 |
| A1-Middle | 922 | 279 | 1201 |
| A1_Upper | 0 | 0 | 0 |
| A1_Top | 734 | 1039 | 1773 |
| A2_Bottom | 1204 | 285 | 1489 |
| A2-Middle | 4 | 3 | 7 |
| A2_Upper | 204 | 133 | 337 |
| A2_Top | 300 | 641 | 941 |

## Supplementary Table 5 GO enrichment analysis of DEGs

| | Group | GO Term | Annotated | Significant | Expected | classicFisher | p-value |
|---|---|---|---|---|---|---|---|
| A1/A2 | B1_Bottom_up-regulated genes | GO:0015979 | photosynthesis | 483 | 132 | 4.65 | < 1e-30 |
| | | GO:0019684 | photosynthesis, light reaction | 266 | 80 | 2.56 | < 1e-30 |
| | | GO:0006091 | generation of precursor metabolites and ... | 728 | 95 | 7.02 | < 1e-30 |
| | | GO:0009765 | photosynthesis, light harvesting | 96 | 35 | 0.93 | < 1e-30 |
| | | GO:0009768 | photosynthesis, light harvesting in phot... | 72 | 31 | 0.69 | < 1e-30 |
| | | GO:0009767 | photosynthetic electron transport chain | 109 | 31 | 1.05 | < 1e-30 |
| | | GO:0022900 | electron transport chain | 214 | 33 | 2.06 | 1.40E-29 |
| | | GO:0008152 | metabolic process | 25748 | 368 | 248.14 | 1.50E-27 |
| | | GO:1901564 | organonitrogen compound metabolic proces... | 4224 | 119 | 40.71 | 9.10E-27 |
| | | GO:0019253 | reductive pentose-phosphate cycle | 49 | 19 | 0.47 | 5.10E-26 |
| | | GO:0019685 | photosynthesis, dark reaction | 51 | 19 | 0.49 | 1.30E-25 |
| | | GO:0015977 | carbon fixation | 55 | 19 | 0.53 | 7.20E-25 |
| | | GO:0044237 | cellular metabolic process | 21131 | 313 | 203.65 | 7.20E-23 |
| | | GO:0044710 | single-organism metabolic process | 8779 | 174 | 84.61 | 2.80E-22 |
| | | GO:0015995 | chlorophyll biosynthetic process | 134 | 23 | 1.29 | 4.20E-22 |
| | | GO:0051186 | cofactor metabolic process | 920 | 49 | 8.87 | 6.20E-22 |
| | | GO:0055114 | oxidation-reduction process | 3234 | 94 | 31.17 | 7.20E-22 |
| | | GO:1901566 | organonitrogen compound biosynthetic pro... | 2940 | 88 | 28.33 | 2.80E-21 |
| | | GO:0006779 | porphyrin-containing compound biosynthet... | 149 | 23 | 1.44 | 5.30E-21 |
| | | GO:0009735 | response to cytokinin | 731 | 43 | 7.04 | 5.50E-21 |
| | | GO:0033014 | tetrapyrrole biosynthetic process | 156 | 23 | 1.5 | 1.50E-20 |
| | | GO:0015994 | chlorophyll metabolic process | 180 | 24 | 1.73 | 2.60E-20 |
| | | GO:0009773 | photosynthetic electron transport in pho... | 34 | 14 | 0.33 | 5.80E-20 |
| | | GO:0006778 | porphyrin-containing compound metabolic ... | 201 | 24 | 1.94 | 3.60E-19 |
| | | GO:0033013 | tetrapyrrole metabolic process | 204 | 24 | 1.97 | 5.10E-19 |
| | | GO:0042549 | photosystem II stabilization | 14 | 10 | 0.13 | 6.10E-18 |
| | | GO:0017014 | protein nitrosylation | 45 | 14 | 0.43 | 6.30E-18 |
| | | GO:0018119 | peptidyl-cysteine S-nitrosylation | 45 | 14 | 0.43 | 6.30E-18 |
| | | GO:0042548 | regulation of photosynthesis, light reac... | 57 | 15 | 0.55 | 7.10E-18 |
| | | GO:0046148 | pigment biosynthetic process | 309 | 27 | 2.98 | 7.30E-18 |
| | | GO:0043467 | regulation of generation of precursor me... | 62 | 15 | 0.6 | 2.90E-17 |
| | | GO:0010109 | regulation of photosynthesis | 80 | 16 | 0.77 | 6.70E-17 |

| | | | | | | |
|---|---|---|---|---|---|---|
| | GO:0009416 | response to light stimulus | 2025 | 64 | 19.52 | 1.10E-16 |
| | GO:0032544 | plastid translation | 24 | 11 | 0.23 | 1.30E-16 |
| | GO:0009314 | response to radiation | 2089 | 64 | 20.13 | 4.80E-16 |
| | GO:0042440 | pigment metabolic process | 377 | 27 | 3.63 | 1.10E-15 |
| | GO:0018198 | peptidyl-cysteine modification | 119 | 17 | 1.15 | 2.70E-15 |
| | GO:0009987 | cellular process | 27246 | 349 | 262.58 | 3.90E-15 |
| | GO:0051188 | cofactor biosynthetic process | 520 | 30 | 5.01 | 8.90E-15 |
| | GO:0009657 | plastid organization | 455 | 28 | 4.38 | 1.40E-14 |
| | GO:0044711 | single-organism biosynthetic process | 3775 | 86 | 36.38 | 9.20E-14 |
| | GO:0042742 | defense response to bacterium | 834 | 36 | 8.04 | 1.20E-13 |
| | GO:0006412 | translation | 1717 | 53 | 16.55 | 1.50E-13 |
| | GO:0043043 | peptide biosynthetic process | 1727 | 53 | 16.64 | 1.80E-13 |
| | GO:0043604 | amide biosynthetic process | 1746 | 53 | 16.83 | 2.80E-13 |
| | GO:0044281 | small molecule metabolic process | 3650 | 82 | 35.18 | 8.00E-13 |
| | GO:0006518 | peptide metabolic process | 1896 | 54 | 18.27 | 1.90E-12 |
| | GO:0043603 | cellular amide metabolic process | 1958 | 54 | 18.87 | 6.60E-12 |
| | GO:0010114 | response to red light | 142 | 15 | 1.37 | 1.00E-11 |
| | GO:0009637 | response to blue light | 168 | 16 | 1.62 | 1.10E-11 |
| | GO:0010218 | response to far red light | 170 | 16 | 1.64 | 1.30E-11 |
| | GO:0006782 | protoporphyrinogen IX biosynthetic proce... | 24 | 8 | 0.23 | 4.50E-11 |
| | GO:0046501 | protoporphyrinogen IX metabolic process | 24 | 8 | 0.23 | 4.50E-11 |
| | GO:0009617 | response to bacterium | 1115 | 37 | 10.75 | 1.10E-10 |
| | GO:0009628 | response to abiotic stimulus | 5496 | 101 | 52.97 | 1.70E-10 |
| | GO:0006783 | heme biosynthetic process | 43 | 9 | 0.41 | 2.80E-10 |
| | GO:0043436 | oxoacid metabolic process | 2318 | 56 | 22.34 | 3.80E-10 |
| | GO:0042168 | heme metabolic process | 47 | 9 | 0.45 | 6.60E-10 |
| | GO:0009658 | chloroplast organization | 331 | 19 | 3.19 | 8.20E-10 |
| | GO:0016051 | carbohydrate biosynthetic process | 678 | 27 | 6.53 | 8.70E-10 |
| | GO:0009409 | response to cold | 1052 | 34 | 10.14 | 1.30E-09 |
| | GO:0008150 | biological_process | 46866 | 489 | 451.66 | 1.50E-09 |
| | GO:0005996 | monosaccharide metabolic process | 206 | 15 | 1.99 | 2.00E-09 |
| | GO:0018193 | peptidyl-amino acid modification | 661 | 26 | 6.37 | 2.30E-09 |
| | GO:1905156 | negative regulation of photosynthesis | 38 | 8 | 0.37 | 2.70E-09 |
| | GO:0019318 | hexose metabolic process | 150 | 13 | 1.45 | 2.90E-09 |

| | | | | | |
|---|---|---|---|---|---|
| GO:0010207 | photosystem II assembly | 40 | 8 | 0.39 | 4.10E-09 |
| GO:0019752 | carboxylic acid metabolic process | 2221 | 52 | 21.4 | 4.90E-09 |
| GO:0006082 | organic acid metabolic process | 2655 | 58 | 25.59 | 7.20E-09 |
| GO:0044723 | single-organism carbohydrate metabolic p... | 1187 | 35 | 11.44 | 7.60E-09 |
| GO:0030388 | fructose 1,6-bisphosphate metabolic proc... | 9 | 5 | 0.09 | 9.90E-09 |
| GO:0006732 | coenzyme metabolic process | 634 | 24 | 6.11 | 2.00E-08 |
| GO:0010205 | photoinhibition | 33 | 7 | 0.32 | 2.50E-08 |
| GO:0043155 | negative regulation of photosynthesis, l... | 33 | 7 | 0.32 | 2.50E-08 |
| GO:0046496 | nicotinamide nucleotide metabolic proces... | 287 | 16 | 2.77 | 2.70E-08 |
| GO:0010196 | nonphotochemical quenching | 20 | 6 | 0.19 | 2.70E-08 |
| GO:1990066 | energy quenching | 20 | 6 | 0.19 | 2.70E-08 |
| GO:0032787 | monocarboxylic acid metabolic process | 1199 | 34 | 11.56 | 3.20E-08 |
| GO:0019362 | pyridine nucleotide metabolic process | 292 | 16 | 2.81 | 3.40E-08 |
| GO:0072524 | pyridine-containing compound metabolic p... | 303 | 16 | 2.92 | 5.60E-08 |
| GO:0006000 | fructose metabolic process | 12 | 5 | 0.12 | 6.10E-08 |
| GO:0006733 | oxidoreduction coenzyme metabolic proces... | 315 | 16 | 3.04 | 9.60E-08 |
| GO:0006546 | glycine catabolic process | 25 | 6 | 0.24 | 1.20E-07 |
| GO:0010275 | NAD(P)H dehydrogenase complex assembly | 6 | 4 | 0.06 | 1.30E-07 |
| GO:0009071 | serine family amino acid catabolic proce... | 27 | 6 | 0.26 | 1.90E-07 |
| GO:0098542 | defense response to other organism | 1745 | 41 | 16.82 | 2.10E-07 |
| GO:0009772 | photosynthetic electron transport in pho... | 16 | 5 | 0.15 | 3.30E-07 |
| GO:0016052 | carbohydrate catabolic process | 690 | 23 | 6.65 | 3.70E-07 |
| GO:0009266 | response to temperature stimulus | 1540 | 36 | 14.84 | 1.30E-06 |
| GO:0005975 | carbohydrate metabolic process | 2386 | 48 | 22.99 | 1.60E-06 |
| GO:0006096 | glycolytic process | 184 | 11 | 1.77 | 2.00E-06 |
| GO:0006757 | ATP generation from ADP | 184 | 11 | 1.77 | 2.00E-06 |
| GO:0009135 | purine nucleoside diphosphate metabolic ... | 184 | 11 | 1.77 | 2.00E-06 |
| GO:0009179 | purine ribonucleoside diphosphate metabo... | 184 | 11 | 1.77 | 2.00E-06 |
| GO:0009185 | ribonucleoside diphosphate metabolic pro... | 184 | 11 | 1.77 | 2.00E-06 |
| GO:0046031 | ADP metabolic process | 184 | 11 | 1.77 | 2.00E-06 |
| GO:0006165 | nucleoside diphosphate phosphorylation | 190 | 11 | 1.83 | 2.80E-06 |
| GO:0006090 | pyruvate metabolic process | 231 | 12 | 2.23 | 3.00E-06 |
| GO:0009132 | nucleoside diphosphate metabolic process | 194 | 11 | 1.87 | 3.40E-06 |
| GO:0046939 | nucleotide phosphorylation | 194 | 11 | 1.87 | 3.40E-06 |

| | | | | | | |
|---|---|---|---|---|---|---|
| B1_Bottom_down-regulated genes | GO:0010200 | response to chitin | 371 | 21 | 1.41 | 1.70E-18 |
| | GO:1901700 | response to oxygen-containing compound | 3985 | 56 | 15.2 | 4.90E-18 |
| | GO:0010243 | response to organonitrogen compound | 439 | 21 | 1.67 | 5.20E-17 |
| | GO:0043207 | response to external biotic stimulus | 2606 | 42 | 9.94 | 1.70E-15 |
| | GO:0051707 | response to other organism | 2606 | 42 | 9.94 | 1.70E-15 |
| | GO:0006952 | defense response | 3448 | 48 | 13.15 | 3.10E-15 |
| | GO:0009607 | response to biotic stimulus | 2657 | 42 | 10.13 | 3.30E-15 |
| | GO:0098542 | defense response to other organism | 1745 | 34 | 6.65 | 5.90E-15 |
| | GO:0042221 | response to chemical | 7052 | 70 | 26.89 | 8.90E-15 |
| | GO:0009719 | response to endogenous stimulus | 4305 | 53 | 16.42 | 1.20E-14 |
| | GO:0006950 | response to stress | 8362 | 77 | 31.89 | 1.20E-14 |
| | GO:0009605 | response to external stimulus | 3371 | 46 | 12.85 | 2.70E-14 |
| | GO:0010033 | response to organic substance | 5028 | 57 | 19.17 | 3.00E-14 |
| | GO:0050896 | response to stimulus | 14686 | 106 | 56 | 8.90E-14 |
| | GO:1901698 | response to nitrogen compound | 711 | 21 | 2.71 | 6.40E-13 |
| | GO:0050832 | defense response to fungus | 662 | 20 | 2.52 | 1.60E-12 |
| | GO:0009620 | response to fungus | 862 | 22 | 3.29 | 3.10E-12 |
| | GO:0009725 | response to hormone | 4064 | 47 | 15.5 | 5.00E-12 |
| | GO:0051704 | multi-organism process | 3460 | 42 | 13.19 | 2.00E-11 |
| | GO:0001101 | response to acid chemical | 2953 | 38 | 11.26 | 4.00E-11 |
| | GO:0002252 | immune effector process | 221 | 11 | 0.84 | 1.10E-09 |
| | GO:0009755 | hormone-mediated signaling pathway | 1777 | 27 | 6.78 | 1.20E-09 |
| | GO:0070887 | cellular response to chemical stimulus | 2616 | 33 | 9.98 | 1.50E-09 |
| | GO:0032870 | cellular response to hormone stimulus | 1898 | 27 | 7.24 | 4.70E-09 |
| | GO:0071495 | cellular response to endogenous stimulus | 1926 | 27 | 7.34 | 6.40E-09 |
| | GO:0071310 | cellular response to organic substance | 2264 | 29 | 8.63 | 1.20E-08 |
| | GO:0009873 | ethylene-activated signaling pathway | 486 | 13 | 1.85 | 5.80E-08 |
| | GO:0001944 | vasculature development | 56 | 6 | 0.21 | 7.90E-08 |
| | GO:0072358 | cardiovascular system development | 56 | 6 | 0.21 | 7.90E-08 |
| | GO:0072359 | circulatory system development | 56 | 6 | 0.21 | 7.90E-08 |
| | GO:0009723 | response to ethylene | 798 | 16 | 3.04 | 9.00E-08 |
| | GO:0071369 | cellular response to ethylene stimulus | 508 | 13 | 1.94 | 9.70E-08 |
| | GO:0009617 | response to bacterium | 1115 | 18 | 4.25 | 3.40E-07 |
| | GO:0000289 | nuclear-transcribed mRNA poly(A) tail sh... | 16 | 4 | 0.06 | 3.60E-07 |

| | | | | | |
|---|---|---|---|---|---|
| GO:0000160 | phosphorelay signal transduction system | 578 | 13 | 2.2 | 4.20E-07 |
| GO:0009759 | indole glucosinolate biosynthetic proces... | 19 | 4 | 0.07 | 7.60E-07 |
| GO:0016144 | S-glycoside biosynthetic process | 19 | 4 | 0.07 | 7.60E-07 |
| GO:0042742 | defense response to bacterium | 834 | 15 | 3.18 | 9.00E-07 |
| GO:0006562 | proline catabolic process | 6 | 3 | 0.02 | 1.10E-06 |
| GO:0010133 | proline catabolic process to glutamate | 6 | 3 | 0.02 | 1.10E-06 |
| GO:0010150 | leaf senescence | 275 | 9 | 1.05 | 1.30E-06 |
| GO:0090693 | plant organ senescence | 275 | 9 | 1.05 | 1.30E-06 |
| GO:0009414 | response to water deprivation | 913 | 15 | 3.48 | 2.70E-06 |
| GO:0016143 | S-glycoside metabolic process | 26 | 4 | 0.1 | 2.90E-06 |
| GO:0042343 | indole glucosinolate metabolic process | 26 | 4 | 0.1 | 2.90E-06 |
| GO:0009415 | response to water | 932 | 15 | 3.55 | 3.50E-06 |
| GO:0006537 | glutamate biosynthetic process | 9 | 3 | 0.03 | 4.50E-06 |
| GO:0002376 | immune system process | 1209 | 17 | 4.61 | 4.60E-06 |
| GO:1900056 | negative regulation of leaf senescence | 31 | 4 | 0.12 | 6.00E-06 |
| GO:0009863 | salicylic acid mediated signaling pathwa... | 119 | 6 | 0.45 | 6.90E-06 |
| GO:0010035 | response to inorganic substance | 2439 | 25 | 9.3 | 7.40E-06 |
| GO:0046395 | carboxylic acid catabolic process | 261 | 8 | 1 | 8.20E-06 |
| GO:0007568 | aging | 448 | 10 | 1.71 | 9.90E-06 |
| GO:0071229 | cellular response to acid chemical | 1033 | 15 | 3.94 | 1.20E-05 |
| GO:0009751 | response to salicylic acid | 469 | 10 | 1.79 | 1.50E-05 |
| GO:0035264 | multicellular organism growth | 13 | 3 | 0.05 | 1.50E-05 |
| GO:0009862 | systemic acquired resistance, salicylic ... | 39 | 4 | 0.15 | 1.50E-05 |
| GO:0010120 | camalexin biosynthetic process | 40 | 4 | 0.15 | 1.70E-05 |
| GO:0052317 | camalexin metabolic process | 40 | 4 | 0.15 | 1.70E-05 |
| GO:1900055 | regulation of leaf senescence | 40 | 4 | 0.15 | 1.70E-05 |
| GO:0071446 | cellular response to salicylic acid stim... | 140 | 6 | 0.53 | 1.80E-05 |
| GO:0009864 | induced systemic resistance, jasmonic ac... | 41 | 4 | 0.16 | 1.90E-05 |
| GO:0009403 | toxin biosynthetic process | 42 | 4 | 0.16 | 2.10E-05 |
| GO:0009700 | indole phytoalexin biosynthetic process | 42 | 4 | 0.16 | 2.10E-05 |
| GO:0046217 | indole phytoalexin metabolic process | 42 | 4 | 0.16 | 2.10E-05 |
| GO:0052314 | phytoalexin metabolic process | 42 | 4 | 0.16 | 2.10E-05 |
| GO:0052315 | phytoalexin biosynthetic process | 42 | 4 | 0.16 | 2.10E-05 |
| GO:0016054 | organic acid catabolic process | 299 | 8 | 1.14 | 2.20E-05 |

| | | | | | | |
|---|---|---|---|---|---|---|
| | GO:0019222 | regulation of metabolic process | 7312 | 50 | 27.88 | 2.20E-05 |
| | GO:0014070 | response to organic cyclic compound | 976 | 14 | 3.72 | 2.60E-05 |
| | GO:0007154 | cell communication | 4063 | 33 | 15.49 | 3.00E-05 |
| | GO:0060255 | regulation of macromolecule metabolic pr... | 6839 | 47 | 26.08 | 3.80E-05 |
| | GO:0051716 | cellular response to stimulus | 5913 | 42 | 22.55 | 5.20E-05 |
| | GO:0031323 | regulation of cellular metabolic process | 6945 | 47 | 26.48 | 5.50E-05 |
| | GO:0009409 | response to cold | 1052 | 14 | 4.01 | 5.90E-05 |
| | GO:0010117 | photoprotection | 20 | 3 | 0.08 | 5.90E-05 |
| | GO:0080090 | regulation of primary metabolic process | 6773 | 46 | 25.83 | 6.20E-05 |
| | GO:0006355 | regulation of transcription, DNA-templat... | 5588 | 40 | 21.31 | 6.80E-05 |
| | GO:0009615 | response to virus | 263 | 7 | 1 | 7.40E-05 |
| | GO:0000288 | nuclear-transcribed mRNA catabolic proce... | 59 | 4 | 0.22 | 7.90E-05 |
| | GO:1903506 | regulation of nucleic acid-templated tra... | 5639 | 40 | 21.5 | 8.30E-05 |
| | GO:2001141 | regulation of RNA biosynthetic process | 5639 | 40 | 21.5 | 8.30E-05 |
| | GO:0033611 | oxalate catabolic process | 4 | 2 | 0.02 | 8.60E-05 |
| | GO:0051252 | regulation of RNA metabolic process | 5698 | 40 | 21.73 | 0.0001 |
| | GO:0097659 | nucleic acid-templated transcription | 5907 | 41 | 22.52 | 0.00011 |
| | GO:1901701 | cellular response to oxygen-containing c... | 1405 | 16 | 5.36 | 0.00011 |
| | GO:0032774 | RNA biosynthetic process | 5919 | 41 | 22.57 | 0.00011 |
| | GO:0035556 | intracellular signal transduction | 988 | 13 | 3.77 | 0.00012 |
| | GO:0009682 | induced systemic resistance | 66 | 4 | 0.25 | 0.00012 |
| | GO:0044282 | small molecule catabolic process | 392 | 8 | 1.49 | 0.00014 |
| | GO:0007165 | signal transduction | 3667 | 29 | 13.98 | 0.00015 |
| | GO:0009894 | regulation of catabolic process | 208 | 6 | 0.79 | 0.00016 |
| | GO:0019219 | regulation of nucleobase-containing comp... | 5809 | 40 | 22.15 | 0.00016 |
| | GO:0016032 | viral process | 398 | 8 | 1.52 | 0.00016 |
| | GO:0044700 | single organism signaling | 3680 | 29 | 14.03 | 0.00016 |
| | GO:0023052 | signaling | 3685 | 29 | 14.05 | 0.00017 |
| | GO:0006351 | transcription, DNA-templated | 5841 | 40 | 22.27 | 0.00018 |
| | GO:0046700 | heterocycle catabolic process | 309 | 7 | 1.18 | 0.0002 |
| | GO:0046345 | abscisic acid catabolic process | 6 | 2 | 0.02 | 0.00021 |
| | GO:0044272 | sulfur compound biosynthetic process | 222 | 6 | 0.85 | 0.00022 |
| B1_Middle_up-regulated genes | GO:0010200 | response to chitin | 371 | 34 | 4.77 | 6.60E-19 |
| | GO:0010243 | response to organonitrogen compound | 439 | 34 | 5.65 | 1.10E-16 |

| | | | | | |
|---|---|---|---|---|---|
| GO:0006952 | defense response | 3448 | 104 | 44.35 | 5.50E-16 |
| GO:0006950 | response to stress | 8362 | 177 | 107.55 | 4.60E-12 |
| GO:1901698 | response to nitrogen compound | 711 | 34 | 9.15 | 9.60E-11 |
| GO:1901700 | response to oxygen-containing compound | 3985 | 96 | 51.26 | 2.30E-09 |
| GO:0010033 | response to organic substance | 5028 | 112 | 64.67 | 7.20E-09 |
| GO:0009719 | response to endogenous stimulus | 4305 | 99 | 55.37 | 1.30E-08 |
| GO:0007165 | signal transduction | 3667 | 87 | 47.17 | 2.90E-08 |
| GO:0006468 | protein phosphorylation | 2011 | 57 | 25.87 | 3.20E-08 |
| GO:0044700 | single organism signaling | 3680 | 87 | 47.33 | 3.40E-08 |
| GO:0023052 | signaling | 3685 | 87 | 47.4 | 3.60E-08 |
| GO:0006464 | cellular protein modification process | 4938 | 107 | 63.51 | 6.80E-08 |
| GO:0036211 | protein modification process | 4938 | 107 | 63.51 | 6.80E-08 |
| GO:0009873 | ethylene-activated signaling pathway | 486 | 23 | 6.25 | 1.20E-07 |
| GO:0050896 | response to stimulus | 14686 | 250 | 188.9 | 1.80E-07 |
| GO:0007154 | cell communication | 4063 | 91 | 52.26 | 1.80E-07 |
| GO:0071369 | cellular response to ethylene stimulus | 508 | 23 | 6.53 | 2.70E-07 |
| GO:0071229 | cellular response to acid chemical | 1033 | 35 | 13.29 | 3.00E-07 |
| GO:0043412 | macromolecule modification | 5407 | 111 | 69.55 | 5.50E-07 |
| GO:0009617 | response to bacterium | 1115 | 36 | 14.34 | 6.30E-07 |
| GO:0009607 | response to biotic stimulus | 2657 | 65 | 34.18 | 6.80E-07 |
| GO:0043207 | response to external biotic stimulus | 2606 | 64 | 33.52 | 7.40E-07 |
| GO:0051707 | response to other organism | 2606 | 64 | 33.52 | 7.40E-07 |
| GO:0001101 | response to acid chemical | 2953 | 70 | 37.98 | 7.70E-07 |
| GO:0071310 | cellular response to organic substance | 2264 | 57 | 29.12 | 1.40E-06 |
| GO:0000160 | phosphorelay signal transduction system | 578 | 23 | 7.43 | 2.40E-06 |
| GO:0051716 | cellular response to stimulus | 5913 | 116 | 76.06 | 2.70E-06 |
| GO:0006904 | vesicle docking involved in exocytosis | 68 | 8 | 0.87 | 2.70E-06 |
| GO:0071495 | cellular response to endogenous stimulus | 1926 | 50 | 24.77 | 2.80E-06 |
| GO:1901701 | cellular response to oxygen-containing c... | 1405 | 40 | 18.07 | 3.40E-06 |
| GO:0009755 | hormone-mediated signaling pathway | 1777 | 47 | 22.86 | 3.50E-06 |
| GO:0032870 | cellular response to hormone stimulus | 1898 | 49 | 24.41 | 4.10E-06 |
| GO:0042742 | defense response to bacterium | 834 | 28 | 10.73 | 5.40E-06 |
| GO:0042221 | response to chemical | 7052 | 131 | 90.71 | 8.60E-06 |
| GO:0070887 | cellular response to chemical stimulus | 2616 | 60 | 33.65 | 1.30E-05 |

| | | | | | | |
|---|---|---|---|---|---|---|
| | GO:0035556 | intracellular signal transduction | 988 | 30 | 12.71 | 1.70E-05 |
| | GO:0016310 | phosphorylation | 2619 | 59 | 33.69 | 2.60E-05 |
| | GO:0048278 | vesicle docking | 93 | 8 | 1.2 | 2.80E-05 |
| | GO:0009611 | response to wounding | 674 | 23 | 8.67 | 2.90E-05 |
| | GO:0010105 | negative regulation of ethylene-activate... | 48 | 6 | 0.62 | 3.40E-05 |
| | GO:0070298 | negative regulation of phosphorelay sign... | 48 | 6 | 0.62 | 3.40E-05 |
| | GO:0009605 | response to external stimulus | 3371 | 71 | 43.36 | 3.50E-05 |
| | GO:0000289 | nuclear-transcribed mRNA poly(A) tail sh... | 16 | 4 | 0.21 | 4.40E-05 |
| | GO:0022406 | membrane docking | 100 | 8 | 1.29 | 4.70E-05 |
| | GO:0051865 | protein autoubiquitination | 51 | 6 | 0.66 | 4.90E-05 |
| | GO:0009723 | response to ethylene | 798 | 25 | 10.26 | 5.20E-05 |
| | GO:1902679 | negative regulation of RNA biosynthetic ... | 565 | 20 | 7.27 | 5.70E-05 |
| | GO:1903507 | negative regulation of nucleic acid-temp... | 565 | 20 | 7.27 | 5.70E-05 |
| | GO:0051253 | negative regulation of RNA metabolic pro... | 567 | 20 | 7.29 | 5.90E-05 |
| | GO:0002237 | response to molecule of bacterial origin | 108 | 8 | 1.39 | 8.20E-05 |
| | GO:0009738 | abscisic acid-activated signaling pathwa... | 405 | 16 | 5.21 | 8.80E-05 |
| | GO:0098542 | defense response to other organism | 1745 | 42 | 22.44 | 9.70E-05 |
| | GO:1902532 | negative regulation of intracellular sig... | 58 | 6 | 0.75 | 0.0001 |
| | GO:0010104 | regulation of ethylene-activated signali... | 59 | 6 | 0.76 | 0.00011 |
| | GO:0070297 | regulation of phosphorelay signal transd... | 59 | 6 | 0.76 | 0.00011 |
| | GO:0006979 | response to oxidative stress | 941 | 27 | 12.1 | 0.00011 |
| | GO:1902531 | regulation of intracellular signal trans... | 86 | 7 | 1.11 | 0.00013 |
| | GO:0071215 | cellular response to abscisic acid stimu... | 472 | 17 | 6.07 | 0.00016 |
| | GO:0097306 | cellular response to alcohol | 472 | 17 | 6.07 | 0.00016 |
| | GO:2000022 | regulation of jasmonic acid mediated sig... | 42 | 5 | 0.54 | 0.0002 |
| | GO:0080142 | regulation of salicylic acid biosyntheti... | 10 | 3 | 0.13 | 0.00024 |
| | GO:0045934 | negative regulation of nucleobase-contai... | 631 | 20 | 8.12 | 0.00024 |
| | GO:0048530 | fruit morphogenesis | 11 | 3 | 0.14 | 0.00032 |
| | GO:0071395 | cellular response to jasmonic acid stimu... | 243 | 11 | 3.13 | 0.00035 |
| | GO:0032940 | secretion by cell | 213 | 10 | 2.74 | 0.00048 |
| | GO:0071446 | cellular response to salicylic acid stim... | 140 | 8 | 1.8 | 0.00049 |
| | GO:0009966 | regulation of signal transduction | 473 | 16 | 6.08 | 0.0005 |
| | GO:0023051 | regulation of signaling | 476 | 16 | 6.12 | 0.00053 |
| | GO:0010646 | regulation of cell communication | 483 | 16 | 6.21 | 0.00062 |

| | | | | | | |
|---|---|---|---|---|---|---|
| | GO:0009409 | response to cold | 1052 | 27 | 13.53 | 0.00065 |
| | GO:0046903 | secretion | 228 | 10 | 2.93 | 0.00081 |
| | GO:0006887 | exocytosis | 152 | 8 | 1.96 | 0.00083 |
| | GO:0009863 | salicylic acid mediated signaling pathwa... | 119 | 7 | 1.53 | 0.00091 |
| | GO:0045087 | innate immune response | 1077 | 27 | 13.85 | 0.00091 |
| | GO:0051704 | multi-organism process | 3460 | 66 | 44.5 | 0.00097 |
| | GO:0006076 | (1->3)-beta-D-glucan catabolic process | 4 | 2 | 0.05 | 0.00097 |
| | GO:0010111 | glyoxysome organization | 4 | 2 | 0.05 | 0.00097 |
| | GO:0010441 | guard cell development | 4 | 2 | 0.05 | 0.00097 |
| | GO:0010442 | guard cell morphogenesis | 4 | 2 | 0.05 | 0.00097 |
| | GO:0009697 | salicylic acid biosynthetic process | 16 | 3 | 0.21 | 0.00105 |
| | GO:0016567 | protein ubiquitination | 1432 | 33 | 18.42 | 0.00109 |
| | GO:0009725 | response to hormone | 4064 | 75 | 52.27 | 0.00109 |
| | GO:0009737 | response to abscisic acid | 1378 | 32 | 17.72 | 0.00114 |
| | GO:0002376 | immune system process | 1209 | 29 | 15.55 | 0.00118 |
| | GO:0009867 | jasmonic acid mediated signaling pathway | 241 | 10 | 3.1 | 0.00124 |
| | GO:0006023 | aminoglycan biosynthetic process | 17 | 3 | 0.22 | 0.00126 |
| | GO:0006024 | glycosaminoglycan biosynthetic process | 17 | 3 | 0.22 | 0.00126 |
| | GO:0030203 | glycosaminoglycan metabolic process | 17 | 3 | 0.22 | 0.00126 |
| | GO:0010337 | regulation of salicylic acid metabolic p... | 37 | 4 | 0.48 | 0.00128 |
| | GO:0031347 | regulation of defense response | 568 | 17 | 7.31 | 0.00129 |
| | GO:0097305 | response to alcohol | 1399 | 32 | 17.99 | 0.00145 |
| | GO:0006955 | immune response | 1112 | 27 | 14.3 | 0.00145 |
| | GO:0009816 | defense response to bacterium, incompati... | 129 | 7 | 1.66 | 0.00146 |
| | GO:0045892 | negative regulation of transcription, DN... | 526 | 16 | 6.77 | 0.0015 |
| | GO:0080164 | regulation of nitric oxide metabolic pro... | 5 | 2 | 0.06 | 0.00161 |
| | GO:0010558 | negative regulation of macromolecule bio... | 741 | 20 | 9.53 | 0.00175 |
| | GO:0016192 | vesicle-mediated transport | 849 | 22 | 10.92 | 0.00175 |
| | GO:0006665 | sphingolipid metabolic process | 100 | 6 | 1.29 | 0.0019 |
| | GO:0048583 | regulation of response to stimulus | 1252 | 29 | 16.1 | 0.00197 |
| B1_Middle_down-regulated genes | GO:0009723 | response to ethylene | 798 | 10 | 0.91 | 2.30E-08 |
| | GO:0009891 | positive regulation of biosynthetic proc... | 753 | 9 | 0.86 | 1.80E-07 |
| | GO:0010105 | negative regulation of ethylene-activate... | 48 | 4 | 0.05 | 2.80E-07 |
| | GO:0070298 | negative regulation of phosphorelay sign... | 48 | 4 | 0.05 | 2.80E-07 |

| | | | | | | |
|---|---|---|---|---|---|---|
| | GO:0009893 | positive regulation of metabolic process | 1104 | 10 | 1.26 | 4.60E-07 |
| | GO:1902532 | negative regulation of intracellular sig... | 58 | 4 | 0.07 | 6.10E-07 |
| | GO:0010104 | regulation of ethylene-activated signali... | 59 | 4 | 0.07 | 6.60E-07 |
| | GO:0070297 | regulation of phosphorelay signal transd... | 59 | 4 | 0.07 | 6.60E-07 |
| | GO:0009873 | ethylene-activated signaling pathway | 486 | 7 | 0.55 | 1.40E-06 |
| | GO:0009725 | response to hormone | 4064 | 17 | 4.63 | 1.80E-06 |
| | GO:0071369 | cellular response to ethylene stimulus | 508 | 7 | 0.58 | 1.80E-06 |
| | GO:0009968 | negative regulation of signal transducti... | 178 | 5 | 0.2 | 1.90E-06 |
| | GO:0010648 | negative regulation of cell communicatio... | 178 | 5 | 0.2 | 1.90E-06 |
| | GO:0023057 | negative regulation of signaling | 178 | 5 | 0.2 | 1.90E-06 |
| | GO:0009658 | chloroplast organization | 331 | 6 | 0.38 | 2.20E-06 |
| | GO:1902531 | regulation of intracellular signal trans... | 86 | 4 | 0.1 | 3.00E-06 |
| | GO:0009719 | response to endogenous stimulus | 4305 | 17 | 4.9 | 3.90E-06 |
| | GO:0000160 | phosphorelay signal transduction system | 578 | 7 | 0.66 | 4.30E-06 |
| | GO:0045893 | positive regulation of transcription, DN... | 613 | 7 | 0.7 | 6.30E-06 |
| | GO:1902680 | positive regulation of RNA biosynthetic ... | 613 | 7 | 0.7 | 6.30E-06 |
| | GO:1903508 | positive regulation of nucleic acid-temp... | 613 | 7 | 0.7 | 6.30E-06 |
| | GO:0010033 | response to organic substance | 5028 | 18 | 5.72 | 7.50E-06 |
| | GO:0051254 | positive regulation of RNA metabolic pro... | 630 | 7 | 0.72 | 7.50E-06 |
| | GO:0010557 | positive regulation of macromolecule bio... | 667 | 7 | 0.76 | 1.10E-05 |
| | GO:0045935 | positive regulation of nucleobase-contai... | 668 | 7 | 0.76 | 1.10E-05 |
| | GO:0010628 | positive regulation of gene expression | 674 | 7 | 0.77 | 1.20E-05 |
| | GO:0048583 | regulation of response to stimulus | 1252 | 9 | 1.43 | 1.20E-05 |
| | GO:0009657 | plastid organization | 455 | 6 | 0.52 | 1.40E-05 |
| | GO:0010604 | positive regulation of macromolecule met... | 984 | 8 | 1.12 | 1.60E-05 |
| | GO:0051173 | positive regulation of nitrogen compound... | 719 | 7 | 0.82 | 1.80E-05 |
| | GO:0031328 | positive regulation of cellular biosynth... | 721 | 7 | 0.82 | 1.80E-05 |
| | GO:0009813 | flavonoid biosynthetic process | 296 | 5 | 0.34 | 2.30E-05 |
| | GO:0031325 | positive regulation of cellular metaboli... | 1041 | 8 | 1.18 | 2.30E-05 |
| | GO:0031146 | SCF-dependent proteasomal ubiquitin-depe... | 161 | 4 | 0.18 | 3.60E-05 |
| | GO:0009812 | flavonoid metabolic process | 341 | 5 | 0.39 | 4.50E-05 |
| | GO:0048518 | positive regulation of biological proces... | 1891 | 10 | 2.15 | 5.10E-05 |
| | GO:0080167 | response to karrikin | 367 | 5 | 0.42 | 6.30E-05 |
| | GO:0035556 | intracellular signal transduction | 988 | 7 | 1.12 | 0.00013 |

| | | | | | |
|---|---|---|---|---|---|
| GO:0009755 | hormone-mediated signaling pathway | 1777 | 9 | 2.02 | 0.00017 |
| GO:0048522 | positive regulation of cellular process | 1414 | 8 | 1.61 | 0.0002 |
| GO:0048585 | negative regulation of response to stimu... | 468 | 5 | 0.53 | 0.0002 |
| GO:0009966 | regulation of signal transduction | 473 | 5 | 0.54 | 0.00021 |
| GO:0023051 | regulation of signaling | 476 | 5 | 0.54 | 0.00021 |
| GO:0010646 | regulation of cell communication | 483 | 5 | 0.55 | 0.00023 |
| GO:0032870 | cellular response to hormone stimulus | 1898 | 9 | 2.16 | 0.00028 |
| GO:0071495 | cellular response to endogenous stimulus | 1926 | 9 | 2.19 | 0.00031 |
| GO:0009698 | phenylpropanoid metabolic process | 532 | 5 | 0.61 | 0.00036 |
| GO:0050896 | response to stimulus | 14686 | 29 | 16.72 | 0.00059 |
| GO:0042221 | response to chemical | 7052 | 18 | 8.03 | 0.00062 |
| GO:0009963 | positive regulation of flavonoid biosynt... | 33 | 2 | 0.04 | 0.00066 |
| GO:0019438 | aromatic compound biosynthetic process | 7157 | 18 | 8.15 | 0.00074 |
| GO:0009733 | response to auxin | 981 | 6 | 1.12 | 0.00087 |
| GO:0050789 | regulation of biological process | 11311 | 24 | 12.87 | 0.00088 |
| GO:0071310 | cellular response to organic substance | 2264 | 9 | 2.58 | 0.001 |
| GO:0065007 | biological regulation | 12478 | 25 | 14.2 | 0.00153 |
| GO:0015843 | methylammonium transport | 2 | 1 | 0 | 0.00228 |
| GO:0019748 | secondary metabolic process | 825 | 5 | 0.94 | 0.00249 |
| GO:0019222 | regulation of metabolic process | 7312 | 17 | 8.32 | 0.00259 |
| GO:0070887 | cellular response to chemical stimulus | 2616 | 9 | 2.98 | 0.00269 |
| GO:1901362 | organic cyclic compound biosynthetic pro... | 7386 | 17 | 8.41 | 0.00288 |
| GO:0050794 | regulation of cellular process | 10208 | 21 | 11.62 | 0.00323 |
| GO:0010498 | proteasomal protein catabolic process | 534 | 4 | 0.61 | 0.00324 |
| GO:0043161 | proteasome-mediated ubiquitin-dependent ... | 534 | 4 | 0.61 | 0.00324 |
| GO:0031323 | regulation of cellular metabolic process | 6945 | 16 | 7.91 | 0.00393 |
| GO:0009962 | regulation of flavonoid biosynthetic pro... | 84 | 2 | 0.1 | 0.00418 |
| GO:0007154 | cell communication | 4063 | 11 | 4.62 | 0.00568 |
| GO:0016567 | protein ubiquitination | 1432 | 6 | 1.63 | 0.00571 |
| GO:0015718 | monocarboxylic acid transport | 99 | 2 | 0.11 | 0.00576 |
| GO:0032446 | protein modification by small protein co... | 1493 | 6 | 1.7 | 0.00696 |
| GO:0008150 | biological_process | 46866 | 59 | 53.35 | 0.00731 |
| GO:0080090 | regulation of primary metabolic process | 6773 | 15 | 7.71 | 0.00773 |
| GO:0009628 | response to abiotic stimulus | 5496 | 13 | 6.26 | 0.00799 |

| | | | | | |
|---|---|---|---|---|---|
| | GO:0010438 | cellular response to sulfur starvation | 8 | 1 | 0.01 | 0.00907 |
| | GO:0015760 | glucose-6-phosphate transport | 8 | 1 | 0.01 | 0.00907 |
| | GO:0019419 | sulfate reduction | 8 | 1 | 0.01 | 0.00907 |
| | GO:0080037 | negative regulation of cytokinin-activat... | 8 | 1 | 0.01 | 0.00907 |
| | GO:0006355 | regulation of transcription, DNA-templat... | 5588 | 13 | 6.36 | 0.00914 |
| | GO:0009889 | regulation of biosynthetic process | 6239 | 14 | 7.1 | 0.00916 |
| | GO:0070647 | protein modification by small protein co... | 1586 | 6 | 1.81 | 0.00923 |
| | GO:0009739 | response to gibberellin | 379 | 3 | 0.43 | 0.00932 |
| | GO:1903506 | regulation of nucleic acid-templated tra... | 5639 | 13 | 6.42 | 0.00983 |
| | GO:2001141 | regulation of RNA biosynthetic process | 5639 | 13 | 6.42 | 0.00983 |
| | GO:0031539 | positive regulation of anthocyanin metab... | 9 | 1 | 0.01 | 0.0102 |
| | GO:0006725 | cellular aromatic compound metabolic pro... | 10524 | 20 | 11.98 | 0.01043 |
| | GO:0051252 | regulation of RNA metabolic process | 5698 | 13 | 6.49 | 0.01069 |
| | GO:0015712 | hexose phosphate transport | 10 | 1 | 0.01 | 0.01133 |
| | GO:0010114 | response to red light | 142 | 2 | 0.16 | 0.01152 |
| | GO:0019219 | regulation of nucleobase-containing comp... | 5809 | 13 | 6.61 | 0.01245 |
| | GO:0006351 | transcription, DNA-templated | 5841 | 13 | 6.65 | 0.013 |
| | GO:0009699 | phenylpropanoid biosynthetic process | 438 | 3 | 0.5 | 0.01374 |
| | GO:0097659 | nucleic acid-templated transcription | 5907 | 13 | 6.72 | 0.01419 |
| | GO:0032774 | RNA biosynthetic process | 5919 | 13 | 6.74 | 0.01442 |
| | GO:0009736 | cytokinin-activated signaling pathway | 163 | 2 | 0.19 | 0.01496 |
| | GO:2000112 | regulation of cellular macromolecule bio... | 5959 | 13 | 6.78 | 0.01519 |
| | GO:0071368 | cellular response to cytokinin stimulus | 165 | 2 | 0.19 | 0.01531 |
| | GO:0009094 | L-phenylalanine biosynthetic process | 14 | 1 | 0.02 | 0.01582 |
| | GO:0015717 | triose phosphate transport | 14 | 1 | 0.02 | 0.01582 |
| | GO:0035436 | triose phosphate transmembrane transport | 14 | 1 | 0.02 | 0.01582 |
| | GO:0010218 | response to far red light | 170 | 2 | 0.19 | 0.01619 |
| | GO:0010556 | regulation of macromolecule biosynthetic... | 6020 | 13 | 6.85 | 0.01643 |
| B1_Top_up-regulated genes | GO:0050896 | response to stimulus | 14686 | 470 | 322.35 | 2.40E-21 |
| | GO:0042221 | response to chemical | 7052 | 271 | 154.79 | 5.70E-21 |
| | GO:0010200 | response to chitin | 371 | 45 | 8.14 | 2.40E-20 |
| | GO:0006950 | response to stress | 8362 | 302 | 183.54 | 1.50E-19 |
| | GO:0010243 | response to organonitrogen compound | 439 | 47 | 9.64 | 5.80E-19 |
| | GO:1901700 | response to oxygen-containing compound | 3985 | 175 | 87.47 | 1.00E-18 |

| | | | | | |
|---|---|---|---|---|---|
| GO:0006952 | defense response | 3448 | 151 | 75.68 | 4.60E-16 |
| GO:0009605 | response to external stimulus | 3371 | 140 | 73.99 | 3.50E-13 |
| GO:0009408 | response to heat | 554 | 44 | 12.16 | 3.70E-13 |
| GO:0009607 | response to biotic stimulus | 2657 | 117 | 58.32 | 9.30E-13 |
| GO:0043207 | response to external biotic stimulus | 2606 | 115 | 57.2 | 1.30E-12 |
| GO:0051707 | response to other organism | 2606 | 115 | 57.2 | 1.30E-12 |
| GO:1901698 | response to nitrogen compound | 711 | 49 | 15.61 | 3.20E-12 |
| GO:0010033 | response to organic substance | 5028 | 184 | 110.36 | 3.70E-12 |
| GO:0001101 | response to acid chemical | 2953 | 122 | 64.82 | 1.90E-11 |
| GO:0009620 | response to fungus | 862 | 52 | 18.92 | 9.40E-11 |
| GO:0009719 | response to endogenous stimulus | 4305 | 157 | 94.49 | 2.40E-10 |
| GO:0010035 | response to inorganic substance | 2439 | 101 | 53.53 | 1.00E-09 |
| GO:0009404 | toxin metabolic process | 137 | 18 | 3.01 | 1.50E-09 |
| GO:0009407 | toxin catabolic process | 95 | 15 | 2.09 | 2.60E-09 |
| GO:0090487 | secondary metabolite catabolic process | 95 | 15 | 2.09 | 2.60E-09 |
| GO:0009628 | response to abiotic stimulus | 5496 | 184 | 120.63 | 4.50E-09 |
| GO:0006979 | response to oxidative stress | 941 | 49 | 20.65 | 3.70E-08 |
| GO:0098542 | defense response to other organism | 1745 | 75 | 38.3 | 3.70E-08 |
| GO:0009751 | response to salicylic acid | 469 | 30 | 10.29 | 2.50E-07 |
| GO:0050832 | defense response to fungus | 662 | 37 | 14.53 | 3.10E-07 |
| GO:0051704 | multi-organism process | 3460 | 121 | 75.95 | 3.20E-07 |
| GO:0045087 | innate immune response | 1077 | 51 | 23.64 | 3.60E-07 |
| GO:0002376 | immune system process | 1209 | 55 | 26.54 | 4.40E-07 |
| GO:0009266 | response to temperature stimulus | 1540 | 65 | 33.8 | 5.70E-07 |
| GO:0019748 | secondary metabolic process | 825 | 42 | 18.11 | 6.10E-07 |
| GO:0006955 | immune response | 1112 | 51 | 24.41 | 9.20E-07 |
| GO:0009617 | response to bacterium | 1115 | 51 | 24.47 | 9.90E-07 |
| GO:1901141 | regulation of lignin biosynthetic proces... | 50 | 9 | 1.1 | 1.30E-06 |
| GO:0009723 | response to ethylene | 798 | 39 | 17.52 | 4.00E-06 |
| GO:0002237 | response to molecule of bacterial origin | 108 | 12 | 2.37 | 4.80E-06 |
| GO:0006749 | glutathione metabolic process | 127 | 13 | 2.79 | 4.90E-06 |
| GO:0042542 | response to hydrogen peroxide | 174 | 15 | 3.82 | 7.90E-06 |
| GO:0007568 | aging | 448 | 26 | 9.83 | 8.90E-06 |
| GO:0009411 | response to UV | 369 | 23 | 8.1 | 9.30E-06 |

| | | | | | | |
|---|---|---|---|---|---|---|
| | GO:0060548 | negative regulation of cell death | 65 | 9 | 1.43 | 1.20E-05 |
| | GO:0009415 | response to water | 932 | 42 | 20.46 | 1.20E-05 |
| | GO:0009644 | response to high light intensity | 204 | 16 | 4.48 | 1.30E-05 |
| | GO:0009873 | ethylene-activated signaling pathway | 486 | 27 | 10.67 | 1.30E-05 |
| | GO:0006468 | protein phosphorylation | 2011 | 74 | 44.14 | 1.30E-05 |
| | GO:0007154 | cell communication | 4063 | 129 | 89.18 | 1.60E-05 |
| | GO:0000160 | phosphorelay signal transduction system | 578 | 30 | 12.69 | 1.70E-05 |
| | GO:0009725 | response to hormone | 4064 | 129 | 89.2 | 1.70E-05 |
| | GO:0009414 | response to water deprivation | 913 | 41 | 20.04 | 1.70E-05 |
| | GO:2000762 | regulation of phenylpropanoid metabolic ... | 86 | 10 | 1.89 | 2.00E-05 |
| | GO:0023052 | signaling | 3685 | 118 | 80.88 | 2.70E-05 |
| | GO:0071369 | cellular response to ethylene stimulus | 508 | 27 | 11.15 | 2.90E-05 |
| | GO:0002682 | regulation of immune system process | 291 | 19 | 6.39 | 2.90E-05 |
| | GO:0009642 | response to light intensity | 344 | 21 | 7.55 | 3.00E-05 |
| | GO:0031347 | regulation of defense response | 568 | 29 | 12.47 | 3.10E-05 |
| | GO:0007165 | signal transduction | 3667 | 117 | 80.49 | 3.40E-05 |
| | GO:0044700 | single organism signaling | 3680 | 117 | 80.77 | 4.00E-05 |
| | GO:0043455 | regulation of secondary metabolic proces... | 133 | 12 | 2.92 | 4.00E-05 |
| | GO:0008219 | cell death | 892 | 39 | 19.58 | 4.80E-05 |
| | GO:0042742 | defense response to bacterium | 834 | 37 | 18.31 | 5.50E-05 |
| | GO:0031349 | positive regulation of defense response | 206 | 15 | 4.52 | 5.70E-05 |
| | GO:0009863 | salicylic acid mediated signaling pathwa... | 119 | 11 | 2.61 | 6.60E-05 |
| | GO:0010325 | raffinose family oligosaccharide biosynt... | 11 | 4 | 0.24 | 6.70E-05 |
| | GO:0000302 | response to reactive oxygen species | 421 | 23 | 9.24 | 7.20E-05 |
| | GO:0009809 | lignin biosynthetic process | 261 | 17 | 5.73 | 7.60E-05 |
| | GO:0080134 | regulation of response to stress | 692 | 32 | 15.19 | 8.10E-05 |
| | GO:1901064 | syringal lignin metabolic process | 34 | 6 | 0.75 | 8.80E-05 |
| | GO:1901066 | syringal lignin biosynthetic process | 34 | 6 | 0.75 | 8.80E-05 |
| | GO:1901428 | regulation of syringal lignin biosynthet... | 34 | 6 | 0.75 | 8.80E-05 |
| | GO:1901430 | positive regulation of syringal lignin b... | 34 | 6 | 0.75 | 8.80E-05 |
| | GO:1900376 | regulation of secondary metabolite biosy... | 83 | 9 | 1.82 | 8.80E-05 |
| | GO:0010941 | regulation of cell death | 267 | 17 | 5.86 | 0.0001 |
| | GO:0045088 | regulation of innate immune response | 245 | 16 | 5.38 | 0.00012 |
| | GO:0010150 | leaf senescence | 275 | 17 | 6.04 | 0.00014 |

| | | | | | | |
|---|---|---|---|---|---|---|
| | GO:0090693 | plant organ senescence | 275 | 17 | 6.04 | 0.00014 |
| | GO:0006575 | cellular modified amino acid metabolic p... | 231 | 15 | 5.07 | 0.0002 |
| | GO:0051716 | cellular response to stimulus | 5913 | 169 | 129.79 | 0.00022 |
| | GO:0001944 | vasculature development | 56 | 7 | 1.23 | 0.00022 |
| | GO:0072358 | cardiovascular system development | 56 | 7 | 1.23 | 0.00022 |
| | GO:0072359 | circulatory system development | 56 | 7 | 1.23 | 0.00022 |
| | GO:1900378 | positive regulation of secondary metabol... | 40 | 6 | 0.88 | 0.00022 |
| | GO:0016310 | phosphorylation | 2619 | 85 | 57.49 | 0.00024 |
| | GO:0009737 | response to abscisic acid | 1378 | 51 | 30.25 | 0.00025 |
| | GO:0071446 | cellular response to salicylic acid stim... | 140 | 11 | 3.07 | 0.00028 |
| | GO:0050776 | regulation of immune response | 265 | 16 | 5.82 | 0.00029 |
| | GO:0009808 | lignin metabolic process | 322 | 18 | 7.07 | 0.00032 |
| | GO:0048584 | positive regulation of response to stimu... | 408 | 21 | 8.96 | 0.00032 |
| | GO:0009939 | positive regulation of gibberellic acid ... | 16 | 4 | 0.35 | 0.00034 |
| | GO:0097305 | response to alcohol | 1399 | 51 | 30.71 | 0.00035 |
| | GO:0012501 | programmed cell death | 785 | 33 | 17.23 | 0.00036 |
| | GO:0045089 | positive regulation of innate immune res... | 168 | 12 | 3.69 | 0.00036 |
| | GO:0070887 | cellular response to chemical stimulus | 2616 | 84 | 57.42 | 0.00037 |
| | GO:0009835 | fruit ripening | 44 | 6 | 0.97 | 0.00038 |
| | GO:0071695 | anatomical structure maturation | 44 | 6 | 0.97 | 0.00038 |
| | GO:0009416 | response to light stimulus | 2025 | 68 | 44.45 | 0.00041 |
| | GO:0002684 | positive regulation of immune system pro... | 175 | 12 | 3.84 | 0.00053 |
| | GO:0050778 | positive regulation of immune response | 175 | 12 | 3.84 | 0.00053 |
| | GO:0009816 | defense response to bacterium, incompati... | 129 | 10 | 2.83 | 0.00058 |
| | GO:0035556 | intracellular signal transduction | 988 | 38 | 21.69 | 0.00073 |
| | GO:0071310 | cellular response to organic substance | 2264 | 73 | 49.69 | 0.00078 |
| B1_Top_down-regulated genes | GO:0015979 | photosynthesis | 483 | 75 | 13.35 | < 1e-30 |
| | GO:0009768 | photosynthesis, light harvesting in phot... | 72 | 33 | 1.99 | < 1e-30 |
| | GO:0009765 | photosynthesis, light harvesting | 96 | 33 | 2.65 | 2.80E-27 |
| | GO:0019684 | photosynthesis, light reaction | 266 | 48 | 7.35 | 4.80E-25 |
| | GO:0007167 | enzyme linked receptor protein signaling... | 456 | 58 | 12.6 | 5.50E-22 |
| | GO:0007169 | transmembrane receptor protein tyrosine ... | 456 | 58 | 12.6 | 5.50E-22 |
| | GO:0007166 | cell surface receptor signaling pathway | 498 | 58 | 13.77 | 4.20E-20 |
| | GO:0005976 | polysaccharide metabolic process | 1023 | 73 | 28.28 | 3.00E-13 |

| | | | | | |
|---|---|---|---|---|---|
| GO:0005975 | carbohydrate metabolic process | 2386 | 128 | 65.95 | 8.90E-13 |
| GO:0006091 | generation of precursor metabolites and ... | 728 | 57 | 20.12 | 3.30E-12 |
| GO:0071554 | cell wall organization or biogenesis | 1682 | 98 | 46.49 | 5.40E-12 |
| GO:0044042 | glucan metabolic process | 457 | 39 | 12.63 | 7.60E-10 |
| GO:0051273 | beta-glucan metabolic process | 188 | 23 | 5.2 | 3.00E-09 |
| GO:0030243 | cellulose metabolic process | 161 | 21 | 4.45 | 4.50E-09 |
| GO:0010583 | response to cyclopentenone | 108 | 17 | 2.99 | 7.50E-09 |
| GO:0006073 | cellular glucan metabolic process | 438 | 36 | 12.11 | 9.00E-09 |
| GO:0000272 | polysaccharide catabolic process | 448 | 36 | 12.38 | 1.60E-08 |
| GO:0071555 | cell wall organization | 1210 | 68 | 33.45 | 4.10E-08 |
| GO:0009637 | response to blue light | 168 | 20 | 4.64 | 5.00E-08 |
| GO:0044264 | cellular polysaccharide metabolic proces... | 581 | 41 | 16.06 | 6.40E-08 |
| GO:0045229 | external encapsulating structure organiz... | 1302 | 69 | 35.99 | 2.90E-07 |
| GO:0044262 | cellular carbohydrate metabolic process | 914 | 53 | 25.26 | 4.90E-07 |
| GO:0006740 | NADPH regeneration | 4 | 4 | 0.11 | 5.80E-07 |
| GO:0009780 | photosynthetic NADP+ reduction | 4 | 4 | 0.11 | 5.80E-07 |
| GO:0009250 | glucan biosynthetic process | 220 | 21 | 6.08 | 1.00E-06 |
| GO:0000271 | polysaccharide biosynthetic process | 423 | 31 | 11.69 | 1.10E-06 |
| GO:0051258 | protein polymerization | 186 | 19 | 5.14 | 1.20E-06 |
| GO:0044700 | single organism signaling | 3680 | 150 | 101.72 | 1.30E-06 |
| GO:0023052 | signaling | 3685 | 150 | 101.86 | 1.40E-06 |
| GO:0009834 | plant-type secondary cell wall biogenesi... | 189 | 19 | 5.22 | 1.50E-06 |
| GO:0007017 | microtubule-based process | 345 | 27 | 9.54 | 1.60E-06 |
| GO:0016052 | carbohydrate catabolic process | 690 | 42 | 19.07 | 2.20E-06 |
| GO:0060918 | auxin transport | 252 | 22 | 6.97 | 2.50E-06 |
| GO:0007165 | signal transduction | 3667 | 148 | 101.36 | 2.60E-06 |
| GO:0042546 | cell wall biogenesis | 492 | 33 | 13.6 | 3.60E-06 |
| GO:0051274 | beta-glucan biosynthetic process | 131 | 15 | 3.62 | 3.70E-06 |
| GO:0009914 | hormone transport | 261 | 22 | 7.21 | 4.40E-06 |
| GO:0009416 | response to light stimulus | 2025 | 91 | 55.97 | 4.90E-06 |
| GO:0007154 | cell communication | 4063 | 159 | 112.3 | 6.10E-06 |
| GO:0010067 | procambium histogenesis | 18 | 6 | 0.5 | 6.20E-06 |
| GO:0034637 | cellular carbohydrate biosynthetic proce... | 438 | 30 | 12.11 | 6.40E-06 |
| GO:0050896 | response to stimulus | 14686 | 481 | 405.93 | 6.80E-06 |

| | | | | | |
|---|---|---|---|---|---|
| GO:0044036 | cell wall macromolecule metabolic proces... | 333 | 25 | 9.2 | 7.90E-06 |
| GO:0030244 | cellulose biosynthetic process | 108 | 13 | 2.99 | 9.40E-06 |
| GO:0071669 | plant-type cell wall organization or bio... | 689 | 40 | 19.04 | 1.20E-05 |
| GO:0010065 | primary meristem tissue development | 20 | 6 | 0.55 | 1.20E-05 |
| GO:0042335 | cuticle development | 81 | 11 | 2.24 | 1.40E-05 |
| GO:0009314 | response to radiation | 2089 | 91 | 57.74 | 1.60E-05 |
| GO:0044763 | single-organism cellular process | 14260 | 465 | 394.16 | 1.70E-05 |
| GO:0016051 | carbohydrate biosynthetic process | 678 | 39 | 18.74 | 1.80E-05 |
| GO:0080165 | callose deposition in phloem sieve plate | 7 | 4 | 0.19 | 1.90E-05 |
| GO:0030245 | cellulose catabolic process | 56 | 9 | 1.55 | 2.20E-05 |
| GO:0010218 | response to far red light | 170 | 16 | 4.7 | 2.20E-05 |
| GO:0033692 | cellular polysaccharide biosynthetic pro... | 339 | 24 | 9.37 | 3.10E-05 |
| GO:0010410 | hemicellulose metabolic process | 216 | 18 | 5.97 | 3.70E-05 |
| GO:0051275 | beta-glucan catabolic process | 60 | 9 | 1.66 | 3.80E-05 |
| GO:0009926 | auxin polar transport | 237 | 19 | 6.55 | 3.80E-05 |
| GO:0010114 | response to red light | 142 | 14 | 3.92 | 4.30E-05 |
| GO:0045493 | xylan catabolic process | 26 | 6 | 0.72 | 6.30E-05 |
| GO:0045488 | pectin metabolic process | 320 | 22 | 8.85 | 9.90E-05 |
| GO:0033609 | oxalate metabolic process | 87 | 10 | 2.4 | 0.00015 |
| GO:0010393 | galacturonan metabolic process | 329 | 22 | 9.09 | 0.00015 |
| GO:0009251 | glucan catabolic process | 122 | 12 | 3.37 | 0.00015 |
| GO:0010196 | nonphotochemical quenching | 20 | 5 | 0.55 | 0.00018 |
| GO:1990066 | energy quenching | 20 | 5 | 0.55 | 0.00018 |
| GO:0016049 | cell growth | 1060 | 50 | 29.3 | 0.00022 |
| GO:0006468 | protein phosphorylation | 2011 | 83 | 55.59 | 0.00022 |
| GO:0009767 | photosynthetic electron transport chain | 109 | 11 | 3.01 | 0.00022 |
| GO:0009832 | plant-type cell wall biogenesis | 387 | 24 | 10.7 | 0.00024 |
| GO:0010383 | cell wall polysaccharide metabolic proce... | 253 | 18 | 6.99 | 0.00027 |
| GO:0048869 | cellular developmental process | 2129 | 86 | 58.85 | 0.00034 |
| GO:0010192 | mucilage biosynthetic process | 35 | 6 | 0.97 | 0.00036 |
| GO:0009629 | response to gravity | 196 | 15 | 5.42 | 0.0004 |
| GO:0040007 | growth | 1552 | 66 | 42.9 | 0.00043 |
| GO:0042550 | photosystem I stabilization | 14 | 4 | 0.39 | 0.00047 |
| GO:0009698 | phenylpropanoid metabolic process | 532 | 29 | 14.7 | 0.00049 |

| | | | | | | |
|---|---|---|---|---|---|---|
| | GO:0044711 | single-organism biosynthetic process | 3775 | 138 | 104.34 | 0.00051 |
| | GO:0044710 | single-organism metabolic process | 8779 | 290 | 242.66 | 0.00054 |
| | GO:0046741 | transport of virus in host, tissue to ti... | 15 | 4 | 0.41 | 0.00062 |
| | GO:0080036 | regulation of cytokinin-activated signal... | 26 | 5 | 0.72 | 0.00065 |
| | GO:0044000 | movement in host | 39 | 6 | 1.08 | 0.00066 |
| | GO:0046739 | transport of virus in multicellular host | 39 | 6 | 1.08 | 0.00066 |
| | GO:0051814 | movement in other organism involved in s... | 39 | 6 | 1.08 | 0.00066 |
| | GO:0052126 | movement in host environment | 39 | 6 | 1.08 | 0.00066 |
| | GO:0052192 | movement in environment of other organis... | 39 | 6 | 1.08 | 0.00066 |
| | GO:0048508 | embryonic meristem development | 71 | 8 | 1.96 | 0.00076 |
| | GO:0010395 | rhamnogalacturonan I metabolic process | 2 | 2 | 0.06 | 0.00076 |
| | GO:0010400 | rhamnogalacturonan I side chain metaboli... | 2 | 2 | 0.06 | 0.00076 |
| | GO:0044275 | cellular carbohydrate catabolic process | 188 | 14 | 5.2 | 0.0008 |
| | GO:0009944 | polarity specification of adaxial/abaxia... | 72 | 8 | 1.99 | 0.00084 |
| | GO:0001944 | vasculature development | 56 | 7 | 1.55 | 0.00086 |
| | GO:0072358 | cardiovascular system development | 56 | 7 | 1.55 | 0.00086 |
| | GO:0072359 | circulatory system development | 56 | 7 | 1.55 | 0.00086 |
| | GO:0006949 | syncytium formation | 41 | 6 | 1.13 | 0.00087 |
| | GO:0044723 | single-organism carbohydrate metabolic p... | 1187 | 52 | 32.81 | 0.00089 |
| | GO:0009630 | gravitropism | 169 | 13 | 4.67 | 0.0009 |
| | GO:0009826 | unidimensional cell growth | 714 | 35 | 19.74 | 0.00093 |
| | GO:0010191 | mucilage metabolic process | 74 | 8 | 2.05 | 0.001 |
| | GO:0065001 | specification of axis polarity | 74 | 8 | 2.05 | 0.001 |
| | GO:0044699 | single-organism process | 20782 | 632 | 574.43 | 0.00101 |
| B2_Bottom_up-regulated genes | GO:0016021 | integral component of membrane | 9224 | 2 | 0.33 | 0.027 |
| | GO:0031224 | intrinsic component of membrane | 9660 | 2 | 0.34 | 0.029 |
| | GO:0009941 | chloroplast envelope | 1633 | 1 | 0.06 | 0.057 |
| | GO:0009526 | plastid envelope | 1693 | 1 | 0.06 | 0.059 |
| | GO:0008150 | biological_process | 46866 | 2 | 1.78 | 7.90E-01 |
| | GO:0000002 | mitochondrial genome maintenance | 13 | 0 | 0 | 1.00E+00 |
| | GO:0000003 | reproduction | 4151 | 0 | 0.16 | 1.00E+00 |
| | GO:0000012 | single strand break repair | 3 | 0 | 0 | 1.00E+00 |
| | GO:0000018 | regulation of DNA recombination | 33 | 0 | 0 | 1.00E+00 |
| | GO:0000019 | regulation of mitotic recombination | 2 | 0 | 0 | 1.00E+00 |

| | | | | | |
|---|---|---|---|---|---|
| GO:0000023 | maltose metabolic process | 13 | 0 | 0 | 1.00E+00 |
| GO:0000024 | maltose biosynthetic process | 6 | 0 | 0 | 1.00E+00 |
| GO:0000025 | maltose catabolic process | 5 | 0 | 0 | 1.00E+00 |
| GO:0000027 | ribosomal large subunit assembly | 77 | 0 | 0 | 1.00E+00 |
| GO:0000028 | ribosomal small subunit assembly | 51 | 0 | 0 | 1.00E+00 |
| GO:0000032 | cell wall mannoprotein biosynthetic proc... | 2 | 0 | 0 | 1.00E+00 |
| GO:0000038 | very long-chain fatty acid metabolic pro... | 59 | 0 | 0 | 1.00E+00 |
| GO:0000041 | transition metal ion transport | 178 | 0 | 0.01 | 1.00E+00 |
| GO:0000045 | autophagosome assembly | 9 | 0 | 0 | 1.00E+00 |
| GO:0000050 | urea cycle | 2 | 0 | 0 | 1.00E+00 |
| GO:0000054 | ribosomal subunit export from nucleus | 10 | 0 | 0 | 1.00E+00 |
| GO:0000055 | ribosomal large subunit export from nucl... | 10 | 0 | 0 | 1.00E+00 |
| GO:0000056 | ribosomal small subunit export from nucl... | 5 | 0 | 0 | 1.00E+00 |
| GO:0000059 | protein import into nucleus, docking | 117 | 0 | 0 | 1.00E+00 |
| GO:0000060 | protein import into nucleus, translocati... | 98 | 0 | 0 | 1.00E+00 |
| GO:0000070 | mitotic sister chromatid segregation | 55 | 0 | 0 | 1.00E+00 |
| GO:0000075 | cell cycle checkpoint | 45 | 0 | 0 | 1.00E+00 |
| GO:0000076 | DNA replication checkpoint | 9 | 0 | 0 | 1.00E+00 |
| GO:0000077 | DNA damage checkpoint | 9 | 0 | 0 | 1.00E+00 |
| GO:0000079 | regulation of cyclin-dependent protein s... | 78 | 0 | 0 | 1.00E+00 |
| GO:0000082 | G1/S transition of mitotic cell cycle | 16 | 0 | 0 | 1.00E+00 |
| GO:0000086 | G2/M transition of mitotic cell cycle | 29 | 0 | 0 | 1.00E+00 |
| GO:0000087 | mitotic M phase | 2 | 0 | 0 | 1.00E+00 |
| GO:0000096 | sulfur amino acid metabolic process | 37 | 0 | 0 | 1.00E+00 |
| GO:0000097 | sulfur amino acid biosynthetic process | 6 | 0 | 0 | 1.00E+00 |
| GO:0000098 | sulfur amino acid catabolic process | 3 | 0 | 0 | 1.00E+00 |
| GO:0000103 | sulfate assimilation | 30 | 0 | 0 | 1.00E+00 |
| GO:0000105 | histidine biosynthetic process | 28 | 0 | 0 | 1.00E+00 |
| GO:0000122 | negative regulation of transcription fro... | 19 | 0 | 0 | 1.00E+00 |
| GO:0000154 | rRNA modification | 34 | 0 | 0 | 1.00E+00 |
| GO:0000160 | phosphorelay signal transduction system | 578 | 0 | 0.02 | 1.00E+00 |
| GO:0000162 | tryptophan biosynthetic process | 36 | 0 | 0 | 1.00E+00 |
| GO:0000165 | MAPK cascade | 129 | 0 | 0 | 1.00E+00 |
| GO:0000169 | activation of MAPK activity involved in ... | 2 | 0 | 0 | 1.00E+00 |

| | | | | | | |
|---|---|---|---|---|---|---|
| | GO:0000183 | chromatin silencing at rDNA | 2 | 0 | 0 | 1.00E+00 |
| | GO:0000184 | nuclear-transcribed mRNA catabolic proce... | 33 | 0 | 0 | 1.00E+00 |
| | GO:0000186 | activation of MAPKK activity | 5 | 0 | 0 | 1.00E+00 |
| | GO:0000187 | activation of MAPK activity | 2 | 0 | 0 | 1.00E+00 |
| | GO:0000209 | protein polyubiquitination | 168 | 0 | 0.01 | 1.00E+00 |
| | GO:0000212 | meiotic spindle organization | 9 | 0 | 0 | 1.00E+00 |
| | GO:0000226 | microtubule cytoskeleton organization | 181 | 0 | 0.01 | 1.00E+00 |
| | GO:0000237 | leptotene | 2 | 0 | 0 | 1.00E+00 |
| | GO:0000238 | zygotene | 2 | 0 | 0 | 1.00E+00 |
| | GO:0000244 | spliceosomal tri-snRNP complex assembly | 9 | 0 | 0 | 1.00E+00 |
| | GO:0000245 | spliceosomal complex assembly | 61 | 0 | 0 | 1.00E+00 |
| | GO:0000255 | allantoin metabolic process | 10 | 0 | 0 | 1.00E+00 |
| | GO:0000256 | allantoin catabolic process | 8 | 0 | 0 | 1.00E+00 |
| | GO:0000266 | mitochondrial fission | 22 | 0 | 0 | 1.00E+00 |
| | GO:0000270 | peptidoglycan metabolic process | 2 | 0 | 0 | 1.00E+00 |
| | GO:0000271 | polysaccharide biosynthetic process | 423 | 0 | 0.02 | 1.00E+00 |
| | GO:0000272 | polysaccharide catabolic process | 448 | 0 | 0.02 | 1.00E+00 |
| | GO:0000278 | mitotic cell cycle | 552 | 0 | 0.02 | 1.00E+00 |
| | GO:0000279 | M phase | 4 | 0 | 0 | 1.00E+00 |
| | GO:0000280 | nuclear division | 463 | 0 | 0.02 | 1.00E+00 |
| | GO:0000281 | mitotic cytokinesis | 116 | 0 | 0 | 1.00E+00 |
| | GO:0000288 | nuclear-transcribed mRNA catabolic proce... | 59 | 0 | 0 | 1.00E+00 |
| | GO:0000289 | nuclear-transcribed mRNA poly(A) tail sh... | 16 | 0 | 0 | 1.00E+00 |
| | GO:0000290 | deadenylation-dependent decapping of nuc... | 8 | 0 | 0 | 1.00E+00 |
| | GO:0000291 | nuclear-transcribed mRNA catabolic proce... | 21 | 0 | 0 | 1.00E+00 |
| | GO:0000301 | retrograde transport, vesicle recycling ... | 4 | 0 | 0 | 1.00E+00 |
| | GO:0000302 | response to reactive oxygen species | 421 | 0 | 0.02 | 1.00E+00 |
| | GO:0000303 | response to superoxide | 37 | 0 | 0 | 1.00E+00 |
| | GO:0000304 | response to singlet oxygen | 22 | 0 | 0 | 1.00E+00 |
| | GO:0000305 | response to oxygen radical | 37 | 0 | 0 | 1.00E+00 |
| | GO:0000338 | protein deneddylation | 23 | 0 | 0 | 1.00E+00 |
| B2_Bottom_down-regulated genes | GO:0071329 | cellular response to sucrose stimulus | 13 | 1 | 0 | 2.50E-04 |
| | GO:0010086 | embryonic root morphogenesis | 14 | 1 | 0 | 2.70E-04 |
| | GO:0007231 | osmosensory signaling pathway | 18 | 1 | 0 | 3.40E-04 |

| | | | | | |
|---|---|---|---|---|---|
| GO:0071324 | cellular response to disaccharide stimul... | 18 | 1 | 0 | 3.40E-04 |
| GO:0018106 | peptidyl-histidine phosphorylation | 44 | 1 | 0 | 8.30E-04 |
| GO:0008272 | sulfate transport | 47 | 1 | 0 | 8.90E-04 |
| GO:0018202 | peptidyl-histidine modification | 49 | 1 | 0 | 9.30E-04 |
| GO:0033500 | carbohydrate homeostasis | 50 | 1 | 0 | 9.50E-04 |
| GO:0072348 | sulfur compound transport | 65 | 1 | 0 | 1.23E-03 |
| GO:0071470 | cellular response to osmotic stress | 97 | 1 | 0 | 1.84E-03 |
| GO:0048598 | embryonic morphogenesis | 110 | 1 | 0 | 2.09E-03 |
| GO:0071322 | cellular response to carbohydrate stimul... | 161 | 1 | 0 | 3.05E-03 |
| GO:0009736 | cytokinin-activated signaling pathway | 163 | 1 | 0 | 3.09E-03 |
| GO:0016036 | cellular response to phosphate starvatio... | 164 | 1 | 0 | 3.11E-03 |
| GO:0071368 | cellular response to cytokinin stimulus | 165 | 1 | 0 | 3.13E-03 |
| GO:0010029 | regulation of seed germination | 177 | 1 | 0 | 3.36E-03 |
| GO:1900140 | regulation of seedling development | 184 | 1 | 0 | 3.49E-03 |
| GO:0009744 | response to sucrose | 206 | 1 | 0 | 3.91E-03 |
| GO:0034285 | response to disaccharide | 216 | 1 | 0 | 4.10E-03 |
| GO:0015698 | inorganic anion transport | 249 | 1 | 0 | 4.72E-03 |
| GO:0048509 | regulation of meristem development | 274 | 1 | 0.01 | 5.20E-03 |
| GO:0009267 | cellular response to starvation | 277 | 1 | 0.01 | 5.25E-03 |
| GO:0042594 | response to starvation | 314 | 1 | 0.01 | 5.96E-03 |
| GO:0031669 | cellular response to nutrient levels | 315 | 1 | 0.01 | 5.98E-03 |
| GO:0031667 | response to nutrient levels | 385 | 1 | 0.01 | 7.30E-03 |
| GO:0031668 | cellular response to extracellular stimu... | 394 | 1 | 0.01 | 7.47E-03 |
| GO:0009743 | response to carbohydrate | 395 | 1 | 0.01 | 7.49E-03 |
| GO:0071496 | cellular response to external stimulus | 417 | 1 | 0.01 | 7.91E-03 |
| GO:0009845 | seed germination | 454 | 1 | 0.01 | 8.61E-03 |
| GO:0071214 | cellular response to abiotic stimulus | 457 | 1 | 0.01 | 8.67E-03 |
| GO:0009991 | response to extracellular stimulus | 468 | 1 | 0.01 | 8.88E-03 |
| GO:0090351 | seedling development | 482 | 1 | 0.01 | 9.14E-03 |
| GO:0048831 | regulation of shoot system development | 523 | 1 | 0.01 | 9.92E-03 |
| GO:0010015 | root morphogenesis | 558 | 1 | 0.01 | 1.06E-02 |
| GO:0000160 | phosphorelay signal transduction system | 578 | 1 | 0.01 | 1.10E-02 |
| GO:0048507 | meristem development | 595 | 1 | 0.01 | 1.13E-02 |
| GO:0018193 | peptidyl-amino acid modification | 661 | 1 | 0.01 | 1.25E-02 |

| | | | | | |
|---|---|---|---|---|---|
| GO:0009735 | response to cytokinin | 731 | 1 | 0.01 | 1.39E-02 |
| GO:0006820 | anion transport | 732 | 1 | 0.01 | 1.39E-02 |
| GO:0042742 | defense response to bacterium | 834 | 1 | 0.02 | 1.58E-02 |
| GO:0048580 | regulation of post-embryonic development | 835 | 1 | 0.02 | 1.58E-02 |
| GO:0048878 | chemical homeostasis | 866 | 1 | 0.02 | 1.64E-02 |
| GO:0009414 | response to water deprivation | 913 | 1 | 0.02 | 1.73E-02 |
| GO:1905392 | plant organ morphogenesis | 928 | 1 | 0.02 | 1.76E-02 |
| GO:0009415 | response to water | 932 | 1 | 0.02 | 1.77E-02 |
| GO:0035556 | intracellular signal transduction | 988 | 1 | 0.02 | 1.87E-02 |
| GO:2000026 | regulation of multicellular organismal d... | 1050 | 1 | 0.02 | 1.99E-02 |
| GO:0042592 | homeostatic process | 1093 | 1 | 0.02 | 2.07E-02 |
| GO:0048364 | root development | 1114 | 1 | 0.02 | 2.11E-02 |
| GO:0009617 | response to bacterium | 1115 | 1 | 0.02 | 2.12E-02 |
| GO:0022622 | root system development | 1118 | 1 | 0.02 | 2.12E-02 |
| GO:0051239 | regulation of multicellular organismal p... | 1161 | 1 | 0.02 | 2.20E-02 |
| GO:1901701 | cellular response to oxygen-containing c... | 1405 | 1 | 0.03 | 2.67E-02 |
| GO:0050793 | regulation of developmental process | 1483 | 1 | 0.03 | 2.81E-02 |
| GO:0009888 | tissue development | 1487 | 1 | 0.03 | 2.82E-02 |
| GO:0009790 | embryo development | 1536 | 1 | 0.03 | 2.91E-02 |
| GO:0006970 | response to osmotic stress | 1738 | 1 | 0.03 | 3.30E-02 |
| GO:0098542 | defense response to other organism | 1745 | 1 | 0.03 | 3.31E-02 |
| GO:0009755 | hormone-mediated signaling pathway | 1777 | 1 | 0.03 | 3.37E-02 |
| GO:0032870 | cellular response to hormone stimulus | 1898 | 1 | 0.04 | 3.60E-02 |
| GO:0071495 | cellular response to endogenous stimulus | 1926 | 1 | 0.04 | 0.03654 |
| GO:0006468 | protein phosphorylation | 2011 | 1 | 0.04 | 0.03815 |
| GO:0006811 | ion transport | 2016 | 1 | 0.04 | 0.03825 |
| GO:0065008 | regulation of biological quality | 2080 | 1 | 0.04 | 0.03946 |
| GO:0033554 | cellular response to stress | 2220 | 1 | 0.04 | 0.04212 |
| GO:0009653 | anatomical structure morphogenesis | 2240 | 1 | 0.04 | 0.0425 |
| GO:0048367 | shoot system development | 2242 | 1 | 0.04 | 0.04253 |
| GO:0071310 | cellular response to organic substance | 2264 | 1 | 0.04 | 0.04295 |
| GO:0099402 | plant organ development | 2328 | 1 | 0.04 | 0.04416 |
| GO:0010035 | response to inorganic substance | 2439 | 1 | 0.05 | 0.04627 |
| GO:0044765 | single-organism transport | 2582 | 1 | 0.05 | 0.04898 |

| | | | | | | |
|---|---|---|---|---|---|---|
| | GO:0043207 | response to external biotic stimulus | 2606 | 1 | 0.05 | 0.04944 |
| | GO:0051707 | response to other organism | 2606 | 1 | 0.05 | 0.04944 |
| | GO:0070887 | cellular response to chemical stimulus | 2616 | 1 | 0.05 | 0.04963 |
| | GO:0016310 | phosphorylation | 2619 | 1 | 0.05 | 0.04969 |
| B2_Middle_up-regulated genes | GO:0009605 | response to external stimulus | 3371 | 43 | 9.21 | 5.50E-18 |
| | GO:0043207 | response to external biotic stimulus | 2606 | 37 | 7.12 | 6.80E-17 |
| | GO:0051707 | response to other organism | 2606 | 37 | 7.12 | 6.80E-17 |
| | GO:0009607 | response to biotic stimulus | 2657 | 37 | 7.26 | 1.30E-16 |
| | GO:0010200 | response to chitin | 371 | 16 | 1.01 | 8.00E-15 |
| | GO:0098542 | defense response to other organism | 1745 | 28 | 4.77 | 3.90E-14 |
| | GO:0051704 | multi-organism process | 3460 | 38 | 9.45 | 8.50E-14 |
| | GO:0010243 | response to organonitrogen compound | 439 | 16 | 1.2 | 1.10E-13 |
| | GO:1901700 | response to oxygen-containing compound | 3985 | 40 | 10.89 | 2.90E-13 |
| | GO:0006950 | response to stress | 8362 | 58 | 22.84 | 1.80E-12 |
| | GO:0031323 | regulation of cellular metabolic process | 6945 | 52 | 18.97 | 2.60E-12 |
| | GO:0006355 | regulation of transcription, DNA-templat... | 5588 | 46 | 15.27 | 3.20E-12 |
| | GO:1903506 | regulation of nucleic acid-templated tra... | 5639 | 46 | 15.4 | 4.40E-12 |
| | GO:2001141 | regulation of RNA biosynthetic process | 5639 | 46 | 15.4 | 4.40E-12 |
| | GO:0019222 | regulation of metabolic process | 7312 | 53 | 19.98 | 5.20E-12 |
| | GO:0060255 | regulation of macromolecule metabolic pr... | 6839 | 51 | 18.68 | 5.50E-12 |
| | GO:0097659 | nucleic acid-templated transcription | 5907 | 47 | 16.14 | 5.70E-12 |
| | GO:0032774 | RNA biosynthetic process | 5919 | 47 | 16.17 | 6.10E-12 |
| | GO:0051252 | regulation of RNA metabolic process | 5698 | 46 | 15.57 | 6.30E-12 |
| | GO:0034654 | nucleobase-containing compound biosynthe... | 6407 | 49 | 17.5 | 7.00E-12 |
| | GO:0019219 | regulation of nucleobase-containing comp... | 5809 | 46 | 15.87 | 1.20E-11 |
| | GO:1901698 | response to nitrogen compound | 711 | 17 | 1.94 | 1.40E-11 |
| | GO:0080090 | regulation of primary metabolic process | 6773 | 50 | 18.5 | 1.40E-11 |
| | GO:0018130 | heterocycle biosynthetic process | 6776 | 50 | 18.51 | 1.40E-11 |
| | GO:0006351 | transcription, DNA-templated | 5841 | 46 | 15.96 | 1.50E-11 |
| | GO:0010468 | regulation of gene expression | 6184 | 47 | 16.89 | 2.80E-11 |
| | GO:2000112 | regulation of cellular macromolecule bio... | 5959 | 46 | 16.28 | 3.00E-11 |
| | GO:0010556 | regulation of macromolecule biosynthetic... | 6020 | 46 | 16.45 | 4.20E-11 |
| | GO:0042221 | response to chemical | 7052 | 50 | 19.26 | 6.20E-11 |
| | GO:0050896 | response to stimulus | 14686 | 77 | 40.12 | 8.50E-11 |

| | | | | | |
|---|---|---|---|---|---|
| GO:0031326 | regulation of cellular biosynthetic proc... | 6183 | 46 | 16.89 | 1.00E-10 |
| GO:0019438 | aromatic compound biosynthetic process | 7157 | 50 | 19.55 | 1.10E-10 |
| GO:0051171 | regulation of nitrogen compound metaboli... | 6202 | 46 | 16.94 | 1.10E-10 |
| GO:0009889 | regulation of biosynthetic process | 6239 | 46 | 17.04 | 1.40E-10 |
| GO:0042742 | defense response to bacterium | 834 | 17 | 2.28 | 1.70E-10 |
| GO:1901362 | organic cyclic compound biosynthetic pro... | 7386 | 50 | 20.18 | 3.20E-10 |
| GO:0010033 | response to organic substance | 5028 | 40 | 13.74 | 3.70E-10 |
| GO:0009617 | response to bacterium | 1115 | 18 | 3.05 | 1.90E-09 |
| GO:0050794 | regulation of cellular process | 10208 | 59 | 27.89 | 2.00E-09 |
| GO:0044271 | cellular nitrogen compound biosynthetic ... | 8405 | 51 | 22.96 | 9.20E-09 |
| GO:0065007 | biological regulation | 12478 | 65 | 34.09 | 1.30E-08 |
| GO:0009719 | response to endogenous stimulus | 4305 | 34 | 11.76 | 1.30E-08 |
| GO:0006952 | defense response | 3448 | 30 | 9.42 | 1.40E-08 |
| GO:0050789 | regulation of biological process | 11311 | 61 | 30.9 | 1.40E-08 |
| GO:1901576 | organic substance biosynthetic process | 12132 | 63 | 33.14 | 3.00E-08 |
| GO:0016070 | RNA metabolic process | 7704 | 47 | 21.05 | 4.00E-08 |
| GO:0050832 | defense response to fungus | 662 | 13 | 1.81 | 4.00E-08 |
| GO:0009058 | biosynthetic process | 12567 | 64 | 34.33 | 4.60E-08 |
| GO:0009611 | response to wounding | 674 | 13 | 1.84 | 5.00E-08 |
| GO:0051091 | positive regulation of sequence-specific... | 4 | 3 | 0.01 | 8.00E-08 |
| GO:0009059 | macromolecule biosynthetic process | 8697 | 50 | 23.76 | 8.10E-08 |
| GO:0034645 | cellular macromolecule biosynthetic proc... | 8498 | 49 | 23.22 | 1.10E-07 |
| GO:0044249 | cellular biosynthetic process | 11973 | 61 | 32.71 | 1.20E-07 |
| GO:0009620 | response to fungus | 862 | 14 | 2.35 | 1.20E-07 |
| GO:0009755 | hormone-mediated signaling pathway | 1777 | 19 | 4.85 | 4.30E-07 |
| GO:0001101 | response to acid chemical | 2953 | 25 | 8.07 | 4.50E-07 |
| GO:0010467 | gene expression | 8864 | 48 | 24.21 | 1.00E-06 |
| GO:0090304 | nucleic acid metabolic process | 8589 | 47 | 23.46 | 1.00E-06 |
| GO:0009814 | defense response, incompatible interacti... | 609 | 11 | 1.66 | 1.00E-06 |
| GO:0046483 | heterocycle metabolic process | 10030 | 52 | 27.4 | 1.10E-06 |
| GO:0015760 | glucose-6-phosphate transport | 8 | 3 | 0.02 | 1.10E-06 |
| GO:0032870 | cellular response to hormone stimulus | 1898 | 19 | 5.19 | 1.10E-06 |
| GO:0071495 | cellular response to endogenous stimulus | 1926 | 19 | 5.26 | 1.40E-06 |
| GO:0009725 | response to hormone | 4064 | 29 | 11.1 | 1.60E-06 |

| | | | | | |
|---|---|---|---|---|---|
| GO:1900056 | negative regulation of leaf senescence | 31 | 4 | 0.08 | 1.60E-06 |
| GO:0015712 | hexose phosphate transport | 10 | 3 | 0.03 | 2.40E-06 |
| GO:0009624 | response to nematode | 216 | 7 | 0.59 | 2.40E-06 |
| GO:0006139 | nucleobase-containing compound metabolic... | 9473 | 49 | 25.88 | 2.90E-06 |
| GO:0006807 | nitrogen compound metabolic process | 12473 | 59 | 34.07 | 3.10E-06 |
| GO:1900055 | regulation of leaf senescence | 40 | 4 | 0.11 | 4.50E-06 |
| GO:0006725 | cellular aromatic compound metabolic pro... | 10524 | 52 | 28.75 | 4.80E-06 |
| GO:0034641 | cellular nitrogen compound metabolic pro... | 11444 | 55 | 31.26 | 5.10E-06 |
| GO:0015717 | triose phosphate transport | 14 | 3 | 0.04 | 7.10E-06 |
| GO:0035436 | triose phosphate transmembrane transport | 14 | 3 | 0.04 | 7.10E-06 |
| GO:0071918 | urea transmembrane transport | 2 | 2 | 0.01 | 7.40E-06 |
| GO:1901360 | organic cyclic compound metabolic proces... | 10760 | 52 | 29.39 | 9.40E-06 |
| GO:0070887 | cellular response to chemical stimulus | 2616 | 21 | 7.15 | 9.40E-06 |
| GO:0071704 | organic substance metabolic process | 22814 | 88 | 62.32 | 1.20E-05 |
| GO:0071310 | cellular response to organic substance | 2264 | 19 | 6.18 | 1.40E-05 |
| GO:0015714 | phosphoenolpyruvate transport | 18 | 3 | 0.05 | 1.60E-05 |
| GO:0001944 | vasculature development | 56 | 4 | 0.15 | 1.80E-05 |
| GO:0072358 | cardiovascular system development | 56 | 4 | 0.15 | 1.80E-05 |
| GO:0072359 | circulatory system development | 56 | 4 | 0.15 | 1.80E-05 |
| GO:0009759 | indole glucosinolate biosynthetic proces... | 19 | 3 | 0.05 | 1.90E-05 |
| GO:0016144 | S-glycoside biosynthetic process | 19 | 3 | 0.05 | 1.90E-05 |
| GO:0015713 | phosphoglycerate transport | 20 | 3 | 0.05 | 2.20E-05 |
| GO:0042873 | aldonate transport | 20 | 3 | 0.05 | 2.20E-05 |
| GO:0034219 | carbohydrate transmembrane transport | 62 | 4 | 0.17 | 2.60E-05 |
| GO:0071229 | cellular response to acid chemical | 1033 | 12 | 2.82 | 2.90E-05 |
| GO:0044237 | cellular metabolic process | 21131 | 82 | 57.73 | 3.20E-05 |
| GO:0015851 | nucleobase transport | 72 | 4 | 0.2 | 4.80E-05 |
| GO:0016143 | S-glycoside metabolic process | 26 | 3 | 0.07 | 5.00E-05 |
| GO:0042343 | indole glucosinolate metabolic process | 26 | 3 | 0.07 | 5.00E-05 |
| GO:0009788 | negative regulation of abscisic acid-act... | 74 | 4 | 0.2 | 5.30E-05 |
| GO:1901420 | negative regulation of response to alcoh... | 74 | 4 | 0.2 | 5.30E-05 |
| GO:0009873 | ethylene-activated signaling pathway | 486 | 8 | 1.33 | 6.20E-05 |
| GO:0080169 | cellular response to boron-containing su... | 5 | 2 | 0.01 | 7.40E-05 |
| GO:0009723 | response to ethylene | 798 | 10 | 2.18 | 7.40E-05 |

| | | | | | | |
|---|---|---|---|---|---|---|
| | GO:0042430 | indole-containing compound metabolic pro... | 157 | 5 | 0.43 | 7.60E-05 |
| | GO:0071369 | cellular response to ethylene stimulus | 508 | 8 | 1.39 | 8.40E-05 |
| B2_Middle_down-regulated genes | GO:0015979 | photosynthesis | 483 | 23 | 1.94 | 5.80E-18 |
| | GO:0019684 | photosynthesis, light reaction | 266 | 12 | 1.07 | 1.10E-09 |
| | GO:0019253 | reductive pentose-phosphate cycle | 49 | 6 | 0.2 | 4.80E-08 |
| | GO:0019685 | photosynthesis, dark reaction | 51 | 6 | 0.21 | 6.10E-08 |
| | GO:0055114 | oxidation-reduction process | 3234 | 35 | 13.01 | 9.00E-08 |
| | GO:0015977 | carbon fixation | 55 | 6 | 0.22 | 9.70E-08 |
| | GO:0042548 | regulation of photosynthesis, light reac... | 57 | 6 | 0.23 | 1.20E-07 |
| | GO:0044710 | single-organism metabolic process | 8779 | 66 | 35.31 | 1.40E-07 |
| | GO:0043467 | regulation of generation of precursor me... | 62 | 6 | 0.25 | 2.00E-07 |
| | GO:0009767 | photosynthetic electron transport chain | 109 | 7 | 0.44 | 3.20E-07 |
| | GO:0010109 | regulation of photosynthesis | 80 | 6 | 0.32 | 9.20E-07 |
| | GO:0009628 | response to abiotic stimulus | 5496 | 46 | 22.1 | 1.20E-06 |
| | GO:0006091 | generation of precursor metabolites and ... | 728 | 14 | 2.93 | 1.80E-06 |
| | GO:0000272 | polysaccharide catabolic process | 448 | 11 | 1.8 | 2.40E-06 |
| | GO:0044711 | single-organism biosynthetic process | 3775 | 35 | 15.18 | 3.20E-06 |
| | GO:0016052 | carbohydrate catabolic process | 690 | 13 | 2.78 | 5.30E-06 |
| | GO:0009416 | response to light stimulus | 2025 | 23 | 8.14 | 8.30E-06 |
| | GO:0010205 | photoinhibition | 33 | 4 | 0.13 | 9.50E-06 |
| | GO:0043155 | negative regulation of photosynthesis, l... | 33 | 4 | 0.13 | 9.50E-06 |
| | GO:0005975 | carbohydrate metabolic process | 2386 | 25 | 9.6 | 1.30E-05 |
| | GO:0009314 | response to radiation | 2089 | 23 | 8.4 | 1.40E-05 |
| | GO:1905156 | negative regulation of photosynthesis | 38 | 4 | 0.15 | 1.70E-05 |
| | GO:0005976 | polysaccharide metabolic process | 1023 | 15 | 4.11 | 2.00E-05 |
| | GO:0010207 | photosystem II assembly | 40 | 4 | 0.16 | 2.10E-05 |
| | GO:0022900 | electron transport chain | 214 | 7 | 0.86 | 2.80E-05 |
| | GO:2000038 | regulation of stomatal complex developme... | 46 | 4 | 0.19 | 3.60E-05 |
| | GO:0015976 | carbon utilization | 19 | 3 | 0.08 | 5.90E-05 |
| | GO:0045493 | xylan catabolic process | 26 | 3 | 0.1 | 1.60E-04 |
| | GO:0043436 | oxoacid metabolic process | 2318 | 22 | 9.32 | 1.80E-04 |
| | GO:0005996 | monosaccharide metabolic process | 206 | 6 | 0.83 | 2.00E-04 |
| | GO:0010037 | response to carbon dioxide | 29 | 3 | 0.12 | 2.20E-04 |
| | GO:2000122 | negative regulation of stomatal complex ... | 29 | 3 | 0.12 | 2.20E-04 |

| | | | | | |
|---|---|---|---|---|---|
| GO:0009620 | response to fungus | 862 | 12 | 3.47 | 2.20E-04 |
| GO:0010410 | hemicellulose metabolic process | 216 | 6 | 0.87 | 2.60E-04 |
| GO:0045490 | pectin catabolic process | 218 | 6 | 0.88 | 2.70E-04 |
| GO:0098542 | defense response to other organism | 1745 | 18 | 7.02 | 2.70E-04 |
| GO:0055129 | L-proline biosynthetic process | 7 | 2 | 0.03 | 3.30E-04 |
| GO:0045488 | pectin metabolic process | 320 | 7 | 1.29 | 3.40E-04 |
| GO:0044281 | small molecule metabolic process | 3650 | 29 | 14.68 | 3.60E-04 |
| GO:0009414 | response to water deprivation | 913 | 12 | 3.67 | 3.60E-04 |
| GO:0043207 | response to external biotic stimulus | 2606 | 23 | 10.48 | 3.70E-04 |
| GO:0051707 | response to other organism | 2606 | 23 | 10.48 | 3.70E-04 |
| GO:0010393 | galacturonan metabolic process | 329 | 7 | 1.32 | 4.00E-04 |
| GO:0009415 | response to water | 932 | 12 | 3.75 | 4.40E-04 |
| GO:0009611 | response to wounding | 674 | 10 | 2.71 | 4.40E-04 |
| GO:0009607 | response to biotic stimulus | 2657 | 23 | 10.69 | 4.80E-04 |
| GO:0010383 | cell wall polysaccharide metabolic proce... | 253 | 6 | 1.02 | 5.90E-04 |
| GO:0009765 | photosynthesis, light harvesting | 96 | 4 | 0.39 | 6.30E-04 |
| GO:0009617 | response to bacterium | 1115 | 13 | 4.48 | 6.40E-04 |
| GO:1901700 | response to oxygen-containing compound | 3985 | 30 | 16.03 | 6.80E-04 |
| GO:0019752 | carboxylic acid metabolic process | 2221 | 20 | 8.93 | 7.00E-04 |
| GO:0031408 | oxylipin biosynthetic process | 101 | 4 | 0.41 | 7.70E-04 |
| GO:0017014 | protein nitrosylation | 45 | 3 | 0.18 | 8.00E-04 |
| GO:0018119 | peptidyl-cysteine S-nitrosylation | 45 | 3 | 0.18 | 8.00E-04 |
| GO:0006636 | unsaturated fatty acid biosynthetic proc... | 46 | 3 | 0.19 | 8.60E-04 |
| GO:0001101 | response to acid chemical | 2953 | 24 | 11.88 | 8.70E-04 |
| GO:0044255 | cellular lipid metabolic process | 1647 | 16 | 6.62 | 1.11E-03 |
| GO:0006082 | organic acid metabolic process | 2655 | 22 | 10.68 | 1.12E-03 |
| GO:0044723 | single-organism carbohydrate metabolic p... | 1187 | 13 | 4.77 | 1.13E-03 |
| GO:0031222 | arabinan catabolic process | 13 | 2 | 0.05 | 1.22E-03 |
| GO:0032787 | monocarboxylic acid metabolic process | 1199 | 13 | 4.82 | 0.00124 |
| GO:0031407 | oxylipin metabolic process | 117 | 4 | 0.47 | 0.00132 |
| GO:0071554 | cell wall organization or biogenesis | 1682 | 16 | 6.76 | 0.00138 |
| GO:0031221 | arabinan metabolic process | 14 | 2 | 0.06 | 0.00142 |
| GO:0042549 | photosystem II stabilization | 14 | 2 | 0.06 | 0.00142 |
| GO:0050832 | defense response to fungus | 662 | 9 | 2.66 | 0.00156 |

| | | | | | |
|---|---|---|---|---|---|
| GO:0033559 | unsaturated fatty acid metabolic process | 61 | 3 | 0.25 | 0.00194 |
| GO:0043094 | cellular metabolic compound salvage | 224 | 5 | 0.9 | 0.00221 |
| GO:0009853 | photorespiration | 137 | 4 | 0.55 | 0.00235 |
| GO:0010374 | stomatal complex development | 137 | 4 | 0.55 | 0.00235 |
| GO:0009605 | response to external stimulus | 3371 | 25 | 13.56 | 0.00239 |
| GO:0044036 | cell wall macromolecule metabolic proces... | 333 | 6 | 1.34 | 0.00241 |
| GO:0010114 | response to red light | 142 | 4 | 0.57 | 0.00268 |
| GO:0034440 | lipid oxidation | 143 | 4 | 0.58 | 0.00275 |
| GO:0009753 | response to jasmonic acid | 597 | 8 | 2.4 | 0.00306 |
| GO:0046394 | carboxylic acid biosynthetic process | 1023 | 11 | 4.11 | 0.00308 |
| GO:0010206 | photosystem II repair | 21 | 2 | 0.08 | 0.00321 |
| GO:0019318 | hexose metabolic process | 150 | 4 | 0.6 | 0.00326 |
| GO:0009831 | plant-type cell wall modification involv... | 76 | 3 | 0.31 | 0.00363 |
| GO:0042547 | cell wall modification involved in multi... | 78 | 3 | 0.31 | 0.00391 |
| GO:0032544 | plastid translation | 24 | 2 | 0.1 | 0.00419 |
| GO:0009809 | lignin biosynthetic process | 261 | 5 | 1.05 | 0.00425 |
| GO:0009694 | jasmonic acid metabolic process | 165 | 4 | 0.66 | 0.00457 |
| GO:0009637 | response to blue light | 168 | 4 | 0.68 | 0.00487 |
| GO:0043692 | monoterpene metabolic process | 26 | 2 | 0.1 | 0.00491 |
| GO:0043693 | monoterpene biosynthetic process | 26 | 2 | 0.1 | 0.00491 |
| GO:0010218 | response to far red light | 170 | 4 | 0.68 | 0.00508 |
| GO:0033609 | oxalate metabolic process | 87 | 3 | 0.35 | 0.0053 |
| GO:0080027 | response to herbivore | 87 | 3 | 0.35 | 0.0053 |
| GO:0008610 | lipid biosynthetic process | 1273 | 12 | 5.12 | 0.00575 |
| GO:0051704 | multi-organism process | 3460 | 24 | 13.92 | 0.00663 |
| GO:0045229 | external encapsulating structure organiz... | 1302 | 12 | 5.24 | 0.00682 |
| GO:0042742 | defense response to bacterium | 834 | 9 | 3.35 | 0.00701 |
| GO:0009828 | plant-type cell wall loosening | 99 | 3 | 0.4 | 0.00758 |
| GO:0006629 | lipid metabolic process | 2395 | 18 | 9.63 | 0.00844 |
| GO:0009644 | response to high light intensity | 204 | 4 | 0.82 | 0.00954 |
| GO:0009695 | jasmonic acid biosynthetic process | 108 | 3 | 0.43 | 0.00961 |
| GO:0006950 | response to stress | 8362 | 47 | 33.63 | 0.00972 |
| GO:0009808 | lignin metabolic process | 322 | 5 | 1.3 | 0.01007 |
| GO:0010411 | xyloglucan metabolic process | 111 | 3 | 0.45 | 0.01035 |

| | | | | | | |
|---|---|---|---|---|---|---|
| B2_Top_up-regulated genes | GO:0080027 | response to herbivore | 87 | 6 | 0.3 | 6.40E-07 |
| | GO:0010088 | phloem development | 22 | 4 | 0.08 | 9.80E-07 |
| | GO:0046246 | terpene biosynthetic process | 116 | 6 | 0.4 | 3.50E-06 |
| | GO:0051761 | sesquiterpene metabolic process | 67 | 5 | 0.23 | 3.90E-06 |
| | GO:0051762 | sesquiterpene biosynthetic process | 67 | 5 | 0.23 | 3.90E-06 |
| | GO:0016106 | sesquiterpenoid biosynthetic process | 78 | 5 | 0.27 | 8.20E-06 |
| | GO:0042214 | terpene metabolic process | 135 | 6 | 0.47 | 8.40E-06 |
| | GO:0006714 | sesquiterpenoid metabolic process | 86 | 5 | 0.3 | 1.30E-05 |
| | GO:0008299 | isoprenoid biosynthetic process | 441 | 9 | 1.53 | 2.70E-05 |
| | GO:0016114 | terpenoid biosynthetic process | 339 | 8 | 1.18 | 2.70E-05 |
| | GO:0010623 | programmed cell death involved in cell d... | 20 | 3 | 0.07 | 4.50E-05 |
| | GO:0051275 | beta-glucan catabolic process | 60 | 4 | 0.21 | 5.90E-05 |
| | GO:0006720 | isoprenoid metabolic process | 499 | 9 | 1.73 | 6.90E-05 |
| | GO:0006721 | terpenoid metabolic process | 393 | 8 | 1.36 | 7.60E-05 |
| | GO:0009607 | response to biotic stimulus | 2657 | 22 | 9.22 | 1.50E-04 |
| | GO:0000272 | polysaccharide catabolic process | 448 | 8 | 1.56 | 1.90E-04 |
| | GO:0045229 | external encapsulating structure organiz... | 1302 | 14 | 4.52 | 2.00E-04 |
| | GO:0019827 | stem cell population maintenance | 159 | 5 | 0.55 | 2.50E-04 |
| | GO:0098727 | maintenance of cell number | 159 | 5 | 0.55 | 2.50E-04 |
| | GO:0010077 | maintenance of inflorescence meristem id... | 38 | 3 | 0.13 | 3.20E-04 |
| | GO:0071555 | cell wall organization | 1210 | 13 | 4.2 | 3.40E-04 |
| | GO:0051094 | positive regulation of developmental pro... | 264 | 6 | 0.92 | 3.40E-04 |
| | GO:0006949 | syncytium formation | 41 | 3 | 0.14 | 4.00E-04 |
| | GO:0009828 | plant-type cell wall loosening | 99 | 4 | 0.34 | 4.10E-04 |
| | GO:0009888 | tissue development | 1487 | 14 | 5.16 | 7.50E-04 |
| | GO:0043207 | response to external biotic stimulus | 2606 | 20 | 9.05 | 7.80E-04 |
| | GO:0051707 | response to other organism | 2606 | 20 | 9.05 | 7.80E-04 |
| | GO:0009251 | glucan catabolic process | 122 | 4 | 0.42 | 9.00E-04 |
| | GO:0051704 | multi-organism process | 3460 | 24 | 12.01 | 9.60E-04 |
| | GO:0030245 | cellulose catabolic process | 56 | 3 | 0.19 | 1.00E-03 |
| | GO:0010074 | maintenance of meristem identity | 130 | 4 | 0.45 | 1.14E-03 |
| | GO:0044247 | cellular polysaccharide catabolic proces... | 131 | 4 | 0.45 | 1.17E-03 |
| | GO:0046741 | transport of virus in host, tissue to ti... | 15 | 2 | 0.05 | 1.22E-03 |
| | GO:0048869 | cellular developmental process | 2129 | 17 | 7.39 | 1.28E-03 |

| | | | | | |
|---|---|---|---|---|---|
| GO:0009911 | positive regulation of flower developmen... | 135 | 4 | 0.47 | 1.31E-03 |
| GO:0051240 | positive regulation of multicellular org... | 234 | 5 | 0.81 | 1.41E-03 |
| GO:0010087 | phloem or xylem histogenesis | 244 | 5 | 0.85 | 1.69E-03 |
| GO:0008610 | lipid biosynthetic process | 1273 | 12 | 4.42 | 1.75E-03 |
| GO:0009827 | plant-type cell wall modification | 147 | 4 | 0.51 | 1.78E-03 |
| GO:0006629 | lipid metabolic process | 2395 | 18 | 8.31 | 1.80E-03 |
| GO:0055114 | oxidation-reduction process | 3234 | 22 | 11.23 | 2.01E-03 |
| GO:0009768 | photosynthesis, light harvesting in phot... | 72 | 3 | 0.25 | 2.06E-03 |
| GO:0050832 | defense response to fungus | 662 | 8 | 2.3 | 2.33E-03 |
| GO:0009627 | systemic acquired resistance | 165 | 4 | 0.57 | 2.71E-03 |
| GO:0007205 | protein kinase C-activating G-protein co... | 23 | 2 | 0.08 | 2.89E-03 |
| GO:0009056 | catabolic process | 3337 | 22 | 11.59 | 2.94E-03 |
| GO:0016052 | carbohydrate catabolic process | 690 | 8 | 2.4 | 3.00E-03 |
| GO:2000243 | positive regulation of reproductive proc... | 179 | 4 | 0.62 | 3.63E-03 |
| GO:0045493 | xylan catabolic process | 26 | 2 | 0.09 | 3.69E-03 |
| GO:0009813 | flavonoid biosynthetic process | 296 | 5 | 1.03 | 3.88E-03 |
| GO:0035336 | long-chain fatty-acyl-CoA metabolic proc... | 27 | 2 | 0.09 | 3.97E-03 |
| GO:0044275 | cellular carbohydrate catabolic process | 188 | 4 | 0.65 | 4.32E-03 |
| GO:0051273 | beta-glucan metabolic process | 188 | 4 | 0.65 | 4.32E-03 |
| GO:0006073 | cellular glucan metabolic process | 438 | 6 | 1.52 | 4.46E-03 |
| GO:0035337 | fatty-acyl-CoA metabolic process | 29 | 2 | 0.1 | 4.58E-03 |
| GO:0009765 | photosynthesis, light harvesting | 96 | 3 | 0.33 | 4.64E-03 |
| GO:0044255 | cellular lipid metabolic process | 1647 | 13 | 5.72 | 5.19E-03 |
| GO:0055062 | phosphate ion homeostasis | 31 | 2 | 0.11 | 5.22E-03 |
| GO:0072506 | trivalent inorganic anion homeostasis | 31 | 2 | 0.11 | 5.22E-03 |
| GO:1901575 | organic substance catabolic process | 3081 | 20 | 10.7 | 5.38E-03 |
| GO:0009057 | macromolecule catabolic process | 1850 | 14 | 6.42 | 5.43E-03 |
| GO:0044042 | glucan metabolic process | 457 | 6 | 1.59 | 5.46E-03 |
| GO:0050896 | response to stimulus | 14686 | 67 | 50.99 | 6.11E-03 |
| GO:0071554 | cell wall organization or biogenesis | 1682 | 13 | 5.84 | 6.15E-03 |
| GO:0044710 | single-organism metabolic process | 8779 | 44 | 30.48 | 6.48E-03 |
| GO:0009605 | response to external stimulus | 3371 | 21 | 11.7 | 6.90E-03 |
| GO:0010063 | positive regulation of trichoblast fate ... | 2 | 1 | 0.01 | 6.93E-03 |
| GO:0030155 | regulation of cell adhesion | 2 | 1 | 0.01 | 6.93E-03 |

| | | | | | | |
|---|---|---|---|---|---|---|
| | GO:0042660 | positive regulation of cell fate specifi... | 2 | 1 | 0.01 | 6.93E-03 |
| | GO:1903890 | positive regulation of plant epidermal c... | 2 | 1 | 0.01 | 6.93E-03 |
| | GO:1905423 | positive regulation of plant organ morph... | 2 | 1 | 0.01 | 6.93E-03 |
| | GO:0009812 | flavonoid metabolic process | 341 | 5 | 1.18 | 6.99E-03 |
| | GO:0048582 | positive regulation of post-embryonic de... | 217 | 4 | 0.75 | 7.12E-03 |
| | GO:0030154 | cell differentiation | 1522 | 12 | 5.28 | 7.20E-03 |
| | GO:0009725 | response to hormone | 4064 | 24 | 14.11 | 7.48E-03 |
| | GO:0046916 | cellular transition metal ion homeostasi... | 223 | 4 | 0.77 | 7.82E-03 |
| | GO:0044000 | movement in host | 39 | 2 | 0.14 | 8.16E-03 |
| | GO:0046739 | transport of virus in multicellular host | 39 | 2 | 0.14 | 8.16E-03 |
| | GO:0051814 | movement in other organism involved in s... | 39 | 2 | 0.14 | 8.16E-03 |
| | GO:0052126 | movement in host environment | 39 | 2 | 0.14 | 8.16E-03 |
| | GO:0052192 | movement in environment of other organis... | 39 | 2 | 0.14 | 8.16E-03 |
| | GO:1902182 | shoot apical meristem development | 40 | 2 | 0.14 | 8.57E-03 |
| | GO:1902183 | regulation of shoot apical meristem deve... | 40 | 2 | 0.14 | 8.57E-03 |
| | GO:0048658 | anther wall tapetum development | 41 | 2 | 0.14 | 8.99E-03 |
| | GO:0046794 | transport of virus | 43 | 2 | 0.15 | 9.86E-03 |
| | GO:0051701 | interaction with host | 129 | 3 | 0.45 | 1.05E-02 |
| | GO:0009620 | response to fungus | 862 | 8 | 2.99 | 1.09E-02 |
| | GO:0009698 | phenylpropanoid metabolic process | 532 | 6 | 1.85 | 1.11E-02 |
| | GO:0009664 | plant-type cell wall organization | 385 | 5 | 1.34 | 1.14E-02 |
| | GO:0035821 | modification of morphology or physiology... | 135 | 3 | 0.47 | 1.18E-02 |
| | GO:0006825 | copper ion transport | 50 | 2 | 0.17 | 1.32E-02 |
| | GO:0044766 | multi-organism transport | 50 | 2 | 0.17 | 1.32E-02 |
| | GO:1902579 | multi-organism localization | 50 | 2 | 0.17 | 1.32E-02 |
| | GO:0006076 | (1->3)-beta-D-glucan catabolic process | 4 | 1 | 0.01 | 1.38E-02 |
| | GO:0010455 | positive regulation of cell fate commitm... | 4 | 1 | 0.01 | 1.38E-02 |
| | GO:0098771 | inorganic ion homeostasis | 560 | 6 | 1.94 | 1.39E-02 |
| | GO:0065008 | regulation of biological quality | 2080 | 14 | 7.22 | 1.42E-02 |
| | GO:0009719 | response to endogenous stimulus | 4305 | 24 | 14.95 | 1.45E-02 |
| | GO:0044699 | single-organism process | 20782 | 87 | 72.15 | 1.54E-02 |
| | GO:0007186 | G-protein coupled receptor signaling pat... | 55 | 2 | 0.19 | 1.58E-02 |
| B2_Top_down-regulated genes | GO:2000762 | regulation of phenylpropanoid metabolic ... | 86 | 6 | 0.29 | 5.00E-07 |
| | GO:1901141 | regulation of lignin biosynthetic proces... | 50 | 5 | 0.17 | 7.60E-07 |

| | | | | | |
|---|---|---|---|---|---|
| GO:0006026 | aminoglycan catabolic process | 57 | 5 | 0.19 | 1.50E-06 |
| GO:0006030 | chitin metabolic process | 57 | 5 | 0.19 | 1.50E-06 |
| GO:0006032 | chitin catabolic process | 57 | 5 | 0.19 | 1.50E-06 |
| GO:0046348 | amino sugar catabolic process | 57 | 5 | 0.19 | 1.50E-06 |
| GO:1901072 | glucosamine-containing compound cataboli... | 57 | 5 | 0.19 | 1.50E-06 |
| GO:1901071 | glucosamine-containing compound metaboli... | 62 | 5 | 0.21 | 2.20E-06 |
| GO:0050832 | defense response to fungus | 662 | 12 | 2.22 | 2.90E-06 |
| GO:0006022 | aminoglycan metabolic process | 74 | 5 | 0.25 | 5.40E-06 |
| GO:0043455 | regulation of secondary metabolic proces... | 133 | 6 | 0.45 | 6.30E-06 |
| GO:0009620 | response to fungus | 862 | 13 | 2.89 | 8.00E-06 |
| GO:1900376 | regulation of secondary metabolite biosy... | 83 | 5 | 0.28 | 9.50E-06 |
| GO:0006950 | response to stress | 8362 | 51 | 28.08 | 9.70E-06 |
| GO:0006040 | amino sugar metabolic process | 87 | 5 | 0.29 | 1.20E-05 |
| GO:0009698 | phenylpropanoid metabolic process | 532 | 10 | 1.79 | 1.40E-05 |
| GO:0009817 | defense response to fungus, incompatible... | 154 | 6 | 0.52 | 1.50E-05 |
| GO:1902475 | L-alpha-amino acid transmembrane transpo... | 44 | 4 | 0.15 | 1.50E-05 |
| GO:0009628 | response to abiotic stimulus | 5496 | 37 | 18.45 | 3.00E-05 |
| GO:0009651 | response to salt stress | 1594 | 17 | 5.35 | 3.00E-05 |
| GO:0009814 | defense response, incompatible interacti... | 609 | 10 | 2.04 | 4.50E-05 |
| GO:0050896 | response to stimulus | 14686 | 74 | 49.31 | 4.50E-05 |
| GO:0009267 | cellular response to starvation | 277 | 7 | 0.93 | 4.60E-05 |
| GO:0003333 | amino acid transmembrane transport | 59 | 4 | 0.2 | 4.80E-05 |
| GO:0006811 | ion transport | 2016 | 19 | 6.77 | 5.30E-05 |
| GO:0031668 | cellular response to extracellular stimu... | 394 | 8 | 1.32 | 6.20E-05 |
| GO:0009605 | response to external stimulus | 3371 | 26 | 11.32 | 6.40E-05 |
| GO:0009816 | defense response to bacterium, incompati... | 129 | 5 | 0.43 | 7.90E-05 |
| GO:0006970 | response to osmotic stress | 1738 | 17 | 5.84 | 8.60E-05 |
| GO:0071496 | cellular response to external stimulus | 417 | 8 | 1.4 | 9.10E-05 |
| GO:1905039 | carboxylic acid transmembrane transport | 70 | 4 | 0.24 | 9.50E-05 |
| GO:0042594 | response to starvation | 314 | 7 | 1.05 | 1.00E-04 |
| GO:0031669 | cellular response to nutrient levels | 315 | 7 | 1.06 | 1.00E-04 |
| GO:1901136 | carbohydrate derivative catabolic proces... | 138 | 5 | 0.46 | 1.10E-04 |
| GO:0009808 | lignin metabolic process | 322 | 7 | 1.08 | 1.20E-04 |
| GO:0019748 | secondary metabolic process | 825 | 11 | 2.77 | 1.20E-04 |

| | | | | | | |
|---|---|---|---|---|---|---|
| | GO:0009699 | phenylpropanoid biosynthetic process | 438 | 8 | 1.47 | 1.30E-04 |
| | GO:0009991 | response to extracellular stimulus | 468 | 8 | 1.57 | 2.00E-04 |
| | GO:1901064 | syringal lignin metabolic process | 34 | 3 | 0.11 | 2.10E-04 |
| | GO:1901066 | syringal lignin biosynthetic process | 34 | 3 | 0.11 | 2.10E-04 |
| | GO:1901428 | regulation of syringal lignin biosynthet... | 34 | 3 | 0.11 | 2.10E-04 |
| | GO:1901430 | positive regulation of syringal lignin b... | 34 | 3 | 0.11 | 2.10E-04 |
| | GO:0034220 | ion transmembrane transport | 1368 | 14 | 4.59 | 2.30E-04 |
| | GO:0016036 | cellular response to phosphate starvatio... | 164 | 5 | 0.55 | 2.40E-04 |
| | GO:0043090 | amino acid import | 37 | 3 | 0.12 | 2.70E-04 |
| | GO:0009809 | lignin biosynthetic process | 261 | 6 | 0.88 | 2.70E-04 |
| | GO:1900378 | positive regulation of secondary metabol... | 40 | 3 | 0.13 | 3.40E-04 |
| | GO:0031667 | response to nutrient levels | 385 | 7 | 1.29 | 3.50E-04 |
| | GO:0015690 | aluminum cation transport | 9 | 2 | 0.03 | 4.00E-04 |
| | GO:0072512 | trivalent inorganic cation transport | 9 | 2 | 0.03 | 4.00E-04 |
| | GO:1903825 | organic acid transmembrane transport | 106 | 4 | 0.36 | 4.70E-04 |
| | GO:0044550 | secondary metabolite biosynthetic proces... | 544 | 8 | 1.83 | 5.40E-04 |
| | GO:0006812 | cation transport | 1316 | 13 | 4.42 | 5.40E-04 |
| | GO:0010411 | xyloglucan metabolic process | 111 | 4 | 0.37 | 5.60E-04 |
| | GO:0006865 | amino acid transport | 205 | 5 | 0.69 | 6.70E-04 |
| | GO:0055085 | transmembrane transport | 2301 | 18 | 7.73 | 7.90E-04 |
| | GO:0015824 | proline transport | 13 | 2 | 0.04 | 8.50E-04 |
| | GO:0044036 | cell wall macromolecule metabolic proces... | 333 | 6 | 1.12 | 9.60E-04 |
| | GO:0001101 | response to acid chemical | 2953 | 21 | 9.92 | 9.80E-04 |
| | GO:0051938 | L-glutamate import | 14 | 2 | 0.05 | 9.90E-04 |
| | GO:0001906 | cell killing | 59 | 3 | 0.2 | 0.00105 |
| | GO:0031640 | killing of cells of other organism | 59 | 3 | 0.2 | 0.00105 |
| | GO:0044364 | disruption of cells of other organism | 59 | 3 | 0.2 | 0.00105 |
| | GO:0042221 | response to chemical | 7052 | 39 | 23.68 | 0.00106 |
| | GO:0010089 | xylem development | 137 | 4 | 0.46 | 0.00122 |
| | GO:0043207 | response to external biotic stimulus | 2606 | 19 | 8.75 | 0.00129 |
| | GO:0051707 | response to other organism | 2606 | 19 | 8.75 | 0.00129 |
| | GO:0055114 | oxidation-reduction process | 3234 | 22 | 10.86 | 0.00131 |
| | GO:0098655 | cation transmembrane transport | 939 | 10 | 3.15 | 0.00137 |
| | GO:0010087 | phloem or xylem histogenesis | 244 | 5 | 0.82 | 0.00146 |

| | | | | | | | |
|---|---|---|---|---|---|---|---|
| | | GO:0009607 | response to biotic stimulus | 2657 | 19 | 8.92 | 0.00161 |
| | | GO:0015849 | organic acid transport | 254 | 5 | 0.85 | 0.00174 |
| | | GO:0043091 | L-arginine import | 19 | 2 | 0.06 | 0.00185 |
| | | GO:0043092 | L-amino acid import | 19 | 2 | 0.06 | 0.00185 |
| | | GO:0090467 | arginine import | 19 | 2 | 0.06 | 0.00185 |
| | | GO:1902023 | L-arginine transport | 19 | 2 | 0.06 | 0.00185 |
| | | GO:0030001 | metal ion transport | 664 | 8 | 2.23 | 0.00193 |
| | | GO:0015804 | neutral amino acid transport | 20 | 2 | 0.07 | 0.00205 |
| | | GO:0098542 | defense response to other organism | 1745 | 14 | 5.86 | 0.00241 |
| | | GO:0006820 | anion transport | 732 | 8 | 2.46 | 0.00349 |
| | | GO:0045087 | innate immune response | 1077 | 10 | 3.62 | 0.00367 |
| | | GO:1901700 | response to oxygen-containing compound | 3985 | 24 | 13.38 | 0.00389 |
| | | GO:0015807 | L-amino acid transport | 28 | 2 | 0.09 | 0.004 |
| | | GO:0015813 | L-glutamate transport | 28 | 2 | 0.09 | 0.004 |
| | | GO:0089711 | L-glutamate transmembrane transport | 28 | 2 | 0.09 | 0.004 |
| | | GO:0006457 | protein folding | 595 | 7 | 2 | 0.00416 |
| | | GO:1902022 | L-lysine transport | 29 | 2 | 0.1 | 0.00429 |
| | | GO:1903401 | L-lysine transmembrane transport | 29 | 2 | 0.1 | 0.00429 |
| | | GO:1903826 | arginine transmembrane transport | 29 | 2 | 0.1 | 0.00429 |
| | | GO:0006955 | immune response | 1112 | 10 | 3.73 | 0.00457 |
| | | GO:0009871 | jasmonic acid and ethylene-dependent sys... | 30 | 2 | 0.1 | 0.00458 |
| | | GO:0033993 | response to lipid | 1877 | 14 | 6.3 | 0.00459 |
| | | GO:0051262 | protein tetramerization | 34 | 2 | 0.11 | 0.00586 |
| | | GO:0046942 | carboxylic acid transport | 213 | 4 | 0.72 | 0.00595 |
| | | GO:0006952 | defense response | 3448 | 21 | 11.58 | 0.00607 |
| | | GO:0010410 | hemicellulose metabolic process | 216 | 4 | 0.73 | 0.00624 |
| | | GO:0051704 | multi-organism process | 3460 | 21 | 11.62 | 0.00631 |
| | | GO:0015711 | organic anion transport | 221 | 4 | 0.74 | 0.00676 |
| | | GO:0002376 | immune system process | 1209 | 10 | 4.06 | 0.00805 |
| | | GO:0010035 | response to inorganic substance | 2439 | 16 | 8.19 | 0.00836 |
| B1/B2 | A1_Middle_up-regulated genes | GO:0009605 | response to external stimulus | 3371 | 111 | 57.24 | 2.20E-11 |
| | | GO:0009607 | response to biotic stimulus | 2657 | 93 | 45.11 | 4.80E-11 |
| | | GO:0043207 | response to external biotic stimulus | 2606 | 91 | 44.25 | 8.90E-11 |
| | | GO:0051707 | response to other organism | 2606 | 91 | 44.25 | 8.90E-11 |

| | | | | | |
|---|---|---|---|---|---|
| GO:0009620 | response to fungus | 862 | 43 | 14.64 | 5.60E-10 |
| GO:0006952 | defense response | 3448 | 107 | 58.54 | 1.40E-09 |
| GO:0006468 | protein phosphorylation | 2011 | 73 | 34.14 | 1.50E-09 |
| GO:0009611 | response to wounding | 674 | 36 | 11.44 | 2.40E-09 |
| GO:0006950 | response to stress | 8362 | 205 | 141.98 | 1.90E-08 |
| GO:0010411 | xyloglucan metabolic process | 111 | 13 | 1.88 | 5.80E-08 |
| GO:0044036 | cell wall macromolecule metabolic proces... | 333 | 22 | 5.65 | 8.50E-08 |
| GO:0005975 | carbohydrate metabolic process | 2386 | 76 | 40.51 | 1.50E-07 |
| GO:0005976 | polysaccharide metabolic process | 1023 | 42 | 17.37 | 2.10E-07 |
| GO:0010410 | hemicellulose metabolic process | 216 | 17 | 3.67 | 2.10E-07 |
| GO:0016310 | phosphorylation | 2619 | 80 | 44.47 | 3.80E-07 |
| GO:0010383 | cell wall polysaccharide metabolic proce... | 253 | 18 | 4.3 | 4.30E-07 |
| GO:0051704 | multi-organism process | 3460 | 97 | 58.75 | 9.90E-07 |
| GO:1901071 | glucosamine-containing compound metaboli... | 62 | 9 | 1.05 | 1.00E-06 |
| GO:0050896 | response to stimulus | 14686 | 311 | 249.35 | 3.30E-06 |
| GO:0042631 | cellular response to water deprivation | 72 | 9 | 1.22 | 3.70E-06 |
| GO:0071462 | cellular response to water stimulus | 72 | 9 | 1.22 | 3.70E-06 |
| GO:0006464 | cellular protein modification process | 4938 | 124 | 83.84 | 7.30E-06 |
| GO:0036211 | protein modification process | 4938 | 124 | 83.84 | 7.30E-06 |
| GO:0010200 | response to chitin | 371 | 20 | 6.3 | 7.30E-06 |
| GO:0098542 | defense response to other organism | 1745 | 55 | 29.63 | 1.10E-05 |
| GO:0006040 | amino sugar metabolic process | 87 | 9 | 1.48 | 1.80E-05 |
| GO:0080027 | response to herbivore | 87 | 9 | 1.48 | 1.80E-05 |
| GO:0010243 | response to organonitrogen compound | 439 | 21 | 7.45 | 2.60E-05 |
| GO:0009694 | jasmonic acid metabolic process | 165 | 12 | 2.8 | 2.80E-05 |
| GO:0050832 | defense response to fungus | 662 | 27 | 11.24 | 3.40E-05 |
| GO:0006796 | phosphate-containing compound metabolic ... | 3784 | 97 | 64.25 | 3.70E-05 |
| GO:0006022 | aminoglycan metabolic process | 74 | 8 | 1.26 | 3.80E-05 |
| GO:0006793 | phosphorus metabolic process | 3809 | 97 | 64.67 | 4.70E-05 |
| GO:0006026 | aminoglycan catabolic process | 57 | 7 | 0.97 | 5.00E-05 |
| GO:0006030 | chitin metabolic process | 57 | 7 | 0.97 | 5.00E-05 |
| GO:0006032 | chitin catabolic process | 57 | 7 | 0.97 | 5.00E-05 |
| GO:0046348 | amino sugar catabolic process | 57 | 7 | 0.97 | 5.00E-05 |
| GO:1901072 | glucosamine-containing compound cataboli... | 57 | 7 | 0.97 | 5.00E-05 |

| | | | | | | |
|---|---|---|---|---|---|---|
| | GO:1901700 | response to oxygen-containing compound | 3985 | 100 | 67.66 | 6.30E-05 |
| | GO:0001906 | cell killing | 59 | 7 | 1 | 6.30E-05 |
| | GO:0031640 | killing of cells of other organism | 59 | 7 | 1 | 6.30E-05 |
| | GO:0044364 | disruption of cells of other organism | 59 | 7 | 1 | 6.30E-05 |
| | GO:0070542 | response to fatty acid | 15 | 4 | 0.25 | 9.70E-05 |
| | GO:0071398 | cellular response to fatty acid | 15 | 4 | 0.25 | 9.70E-05 |
| | GO:0009695 | jasmonic acid biosynthetic process | 108 | 9 | 1.83 | 9.90E-05 |
| | GO:0009617 | response to bacterium | 1115 | 37 | 18.93 | 0.00011 |
| | GO:0009873 | ethylene-activated signaling pathway | 486 | 21 | 8.25 | 0.00011 |
| | GO:0009814 | defense response, incompatible interacti... | 609 | 24 | 10.34 | 0.00015 |
| | GO:0055062 | phosphate ion homeostasis | 31 | 5 | 0.53 | 0.00016 |
| | GO:0072506 | trivalent inorganic anion homeostasis | 31 | 5 | 0.53 | 0.00016 |
| | GO:0031407 | oxylipin metabolic process | 117 | 9 | 1.99 | 0.00018 |
| | GO:0009719 | response to endogenous stimulus | 4305 | 104 | 73.09 | 0.00019 |
| | GO:0071369 | cellular response to ethylene stimulus | 508 | 21 | 8.63 | 0.0002 |
| | GO:0043412 | macromolecule modification | 5407 | 124 | 91.81 | 0.00036 |
| | GO:0015690 | aluminum cation transport | 9 | 3 | 0.15 | 0.00038 |
| | GO:0072512 | trivalent inorganic cation transport | 9 | 3 | 0.15 | 0.00038 |
| | GO:0006955 | immune response | 1112 | 35 | 18.88 | 0.00044 |
| | GO:0010033 | response to organic substance | 5028 | 116 | 85.37 | 0.00046 |
| | GO:0043455 | regulation of secondary metabolic proces... | 133 | 9 | 2.26 | 0.00047 |
| | GO:0046777 | protein autophosphorylation | 323 | 15 | 5.48 | 0.00048 |
| | GO:0045087 | innate immune response | 1077 | 34 | 18.29 | 0.0005 |
| | GO:0019748 | secondary metabolic process | 825 | 28 | 14.01 | 0.00051 |
| | GO:0009809 | lignin biosynthetic process | 261 | 13 | 4.43 | 0.00059 |
| | GO:0070887 | cellular response to chemical stimulus | 2616 | 67 | 44.42 | 0.00062 |
| | GO:0042221 | response to chemical | 7052 | 153 | 119.74 | 0.00083 |
| | GO:0010371 | regulation of gibberellin biosynthetic p... | 12 | 3 | 0.2 | 0.00096 |
| | GO:0002376 | immune system process | 1209 | 36 | 20.53 | 0.00098 |
| | GO:0009804 | coumarin metabolic process | 27 | 4 | 0.46 | 0.00106 |
| | GO:0009805 | coumarin biosynthetic process | 27 | 4 | 0.46 | 0.00106 |
| | GO:0000160 | phosphorelay signal transduction system | 578 | 21 | 9.81 | 0.00107 |
| | GO:0010227 | floral organ abscission | 46 | 5 | 0.78 | 0.00108 |
| | GO:0044262 | cellular carbohydrate metabolic process | 914 | 29 | 15.52 | 0.00115 |

| | | | | | | |
|---|---|---|---|---|---|---|
| | GO:0071310 | cellular response to organic substance | 2264 | 58 | 38.44 | 0.00141 |
| | GO:0006073 | cellular glucan metabolic process | 438 | 17 | 7.44 | 0.00154 |
| | GO:1901141 | regulation of lignin biosynthetic proces... | 50 | 5 | 0.85 | 0.00157 |
| | GO:0009871 | jasmonic acid and ethylene-dependent sys... | 30 | 4 | 0.51 | 0.00159 |
| | GO:0006041 | glucosamine metabolic process | 4 | 2 | 0.07 | 0.00169 |
| | GO:0006042 | glucosamine biosynthetic process | 4 | 2 | 0.07 | 0.00169 |
| | GO:1901073 | glucosamine-containing compound biosynth... | 4 | 2 | 0.07 | 0.00169 |
| | GO:0031408 | oxylipin biosynthetic process | 101 | 7 | 1.71 | 0.00172 |
| | GO:0006979 | response to oxidative stress | 941 | 29 | 15.98 | 0.00178 |
| | GO:0019747 | regulation of isoprenoid metabolic proce... | 31 | 4 | 0.53 | 0.0018 |
| | GO:0071229 | cellular response to acid chemical | 1033 | 31 | 17.54 | 0.00189 |
| | GO:0007154 | cell communication | 4063 | 93 | 68.99 | 0.00215 |
| | GO:0042546 | cell wall biogenesis | 492 | 18 | 8.35 | 0.00217 |
| | GO:0044042 | glucan metabolic process | 457 | 17 | 7.76 | 0.0024 |
| | GO:1901136 | carbohydrate derivative catabolic proces... | 138 | 8 | 2.34 | 0.00257 |
| | GO:0044264 | cellular polysaccharide metabolic proces... | 581 | 20 | 9.86 | 0.00258 |
| | GO:0055081 | anion homeostasis | 56 | 5 | 0.95 | 0.00261 |
| | GO:0072658 | maintenance of protein location in membr... | 5 | 2 | 0.08 | 0.00278 |
| | GO:0072660 | maintenance of protein location in plasm... | 5 | 2 | 0.08 | 0.00278 |
| | GO:1901698 | response to nitrogen compound | 711 | 23 | 12.07 | 0.00281 |
| | GO:0031668 | cellular response to extracellular stimu... | 394 | 15 | 6.69 | 0.00339 |
| | GO:0009838 | abscission | 60 | 5 | 1.02 | 0.00353 |
| | GO:1901701 | cellular response to oxygen-containing c... | 1405 | 38 | 23.86 | 0.00376 |
| | GO:0009699 | phenylpropanoid biosynthetic process | 438 | 16 | 7.44 | 0.00377 |
| | GO:0009808 | lignin metabolic process | 322 | 13 | 5.47 | 0.00378 |
| | GO:0009725 | response to hormone | 4064 | 91 | 69 | 0.00439 |
| | GO:0009268 | response to pH | 20 | 3 | 0.34 | 0.00448 |
| | GO:0000272 | polysaccharide catabolic process | 448 | 16 | 7.61 | 0.00467 |
| A1_Middle_down-regulated genes | GO:0080167 | response to karrikin | 367 | 20 | 1.78 | 2.70E-15 |
| | GO:0042221 | response to chemical | 7052 | 75 | 34.25 | 2.00E-11 |
| | GO:0044710 | single-organism metabolic process | 8779 | 86 | 42.64 | 2.80E-11 |
| | GO:0009813 | flavonoid biosynthetic process | 296 | 14 | 1.44 | 2.80E-10 |
| | GO:0009725 | response to hormone | 4064 | 51 | 19.74 | 3.30E-10 |
| | GO:0009058 | biosynthetic process | 12567 | 106 | 61.03 | 3.70E-10 |

| | | | | | | |
|---|---|---|---|---|---|---|
| | GO:1901576 | organic substance biosynthetic process | 12132 | 103 | 58.92 | 5.60E-10 |
| | GO:0009812 | flavonoid metabolic process | 341 | 14 | 1.66 | 1.80E-09 |
| | GO:0009719 | response to endogenous stimulus | 4305 | 51 | 20.91 | 2.40E-09 |
| | GO:0044711 | single-organism biosynthetic process | 3775 | 46 | 18.33 | 7.20E-09 |
| | GO:0010033 | response to organic substance | 5028 | 55 | 24.42 | 8.10E-09 |
| | GO:0019748 | secondary metabolic process | 825 | 19 | 4.01 | 3.00E-08 |
| | GO:0000160 | phosphorelay signal transduction system | 578 | 16 | 2.81 | 3.20E-08 |
| | GO:0009628 | response to abiotic stimulus | 5496 | 56 | 26.69 | 6.60E-08 |
| | GO:0009698 | phenylpropanoid metabolic process | 532 | 15 | 2.58 | 6.80E-08 |
| | GO:0044699 | single-organism process | 20782 | 143 | 100.93 | 7.40E-08 |
| | GO:0050896 | response to stimulus | 14686 | 110 | 71.32 | 1.50E-07 |
| | GO:0044249 | cellular biosynthetic process | 11973 | 94 | 58.15 | 2.70E-07 |
| | GO:0016053 | organic acid biosynthetic process | 1257 | 22 | 6.1 | 2.90E-07 |
| | GO:0019438 | aromatic compound biosynthetic process | 7157 | 64 | 34.76 | 7.00E-07 |
| | GO:0006082 | organic acid metabolic process | 2655 | 33 | 12.89 | 8.10E-07 |
| | GO:0044283 | small molecule biosynthetic process | 1500 | 23 | 7.28 | 1.50E-06 |
| | GO:0009411 | response to UV | 369 | 11 | 1.79 | 2.30E-06 |
| | GO:0044763 | single-organism cellular process | 14260 | 103 | 69.25 | 3.20E-06 |
| | GO:0044550 | secondary metabolite biosynthetic proces... | 544 | 13 | 2.64 | 3.20E-06 |
| | GO:0046394 | carboxylic acid biosynthetic process | 1023 | 18 | 4.97 | 3.30E-06 |
| | GO:1901362 | organic cyclic compound biosynthetic pro... | 7386 | 63 | 35.87 | 4.30E-06 |
| | GO:0032870 | cellular response to hormone stimulus | 1898 | 25 | 9.22 | 7.30E-06 |
| | GO:0034605 | cellular response to heat | 56 | 5 | 0.27 | 8.10E-06 |
| | GO:0035556 | intracellular signal transduction | 988 | 17 | 4.8 | 8.30E-06 |
| | GO:0009073 | aromatic amino acid family biosynthetic ... | 97 | 6 | 0.47 | 8.40E-06 |
| | GO:0072330 | monocarboxylic acid biosynthetic process | 597 | 13 | 2.9 | 8.70E-06 |
| | GO:0071495 | cellular response to endogenous stimulus | 1926 | 25 | 9.35 | 9.30E-06 |
| | GO:0009723 | response to ethylene | 798 | 15 | 3.88 | 1.00E-05 |
| | GO:0009699 | phenylpropanoid biosynthetic process | 438 | 11 | 2.13 | 1.20E-05 |
| | GO:0001101 | response to acid chemical | 2953 | 32 | 14.34 | 2.00E-05 |
| | GO:0008152 | metabolic process | 25748 | 158 | 125.05 | 2.20E-05 |
| | GO:0009755 | hormone-mediated signaling pathway | 1777 | 23 | 8.63 | 2.30E-05 |
| | GO:0009873 | ethylene-activated signaling pathway | 486 | 11 | 2.36 | 3.00E-05 |
| | GO:0070887 | cellular response to chemical stimulus | 2616 | 29 | 12.7 | 3.30E-05 |

| | | | | | | |
|---|---|---|---|---|---|---|
| | GO:0019752 | carboxylic acid metabolic process | 2221 | 26 | 10.79 | 3.60E-05 |
| | GO:0010025 | wax biosynthetic process | 77 | 5 | 0.37 | 3.90E-05 |
| | GO:0044281 | small molecule metabolic process | 3650 | 36 | 17.73 | 4.20E-05 |
| | GO:0071369 | cellular response to ethylene stimulus | 508 | 11 | 2.47 | 4.50E-05 |
| | GO:1901700 | response to oxygen-containing compound | 3985 | 38 | 19.35 | 5.20E-05 |
| | GO:0010166 | wax metabolic process | 83 | 5 | 0.4 | 5.50E-05 |
| | GO:0009072 | aromatic amino acid family metabolic pro... | 139 | 6 | 0.68 | 6.50E-05 |
| | GO:0043436 | oxoacid metabolic process | 2318 | 26 | 11.26 | 7.20E-05 |
| | GO:0015714 | phosphoenolpyruvate transport | 18 | 3 | 0.09 | 8.80E-05 |
| | GO:0032787 | monocarboxylic acid metabolic process | 1199 | 17 | 5.82 | 9.30E-05 |
| | GO:0071704 | organic substance metabolic process | 22814 | 141 | 110.8 | 9.40E-05 |
| | GO:0015713 | phosphoglycerate transport | 20 | 3 | 0.1 | 0.00012 |
| | GO:0042873 | aldonate transport | 20 | 3 | 0.1 | 0.00012 |
| | GO:0071491 | cellular response to red light | 20 | 3 | 0.1 | 0.00012 |
| | GO:0071310 | cellular response to organic substance | 2264 | 25 | 11 | 0.00013 |
| | GO:0009736 | cytokinin-activated signaling pathway | 163 | 6 | 0.79 | 0.00016 |
| | GO:0009068 | aspartate family amino acid catabolic pr... | 22 | 3 | 0.11 | 0.00016 |
| | GO:0071368 | cellular response to cytokinin stimulus | 165 | 6 | 0.8 | 0.00017 |
| | GO:0009414 | response to water deprivation | 913 | 14 | 4.43 | 0.00017 |
| | GO:0009415 | response to water | 932 | 14 | 4.53 | 0.00022 |
| | GO:0000025 | maltose catabolic process | 5 | 2 | 0.02 | 0.00023 |
| | GO:0034219 | carbohydrate transmembrane transport | 62 | 4 | 0.3 | 0.00024 |
| | GO:0009735 | response to cytokinin | 731 | 12 | 3.55 | 0.00027 |
| | GO:0031323 | regulation of cellular metabolic process | 6945 | 54 | 33.73 | 0.00029 |
| | GO:0006554 | lysine catabolic process | 6 | 2 | 0.03 | 0.00035 |
| | GO:0019477 | L-lysine catabolic process | 6 | 2 | 0.03 | 0.00035 |
| | GO:0046440 | L-lysine metabolic process | 6 | 2 | 0.03 | 0.00035 |
| | GO:0089722 | phosphoenolpyruvate transmembrane transp... | 6 | 2 | 0.03 | 0.00035 |
| | GO:0071214 | cellular response to abiotic stimulus | 457 | 9 | 2.22 | 0.00044 |
| | GO:0009889 | regulation of biosynthetic process | 6239 | 49 | 30.3 | 0.00047 |
| | GO:0070141 | response to UV-A | 7 | 2 | 0.03 | 0.00049 |
| | GO:0071492 | cellular response to UV-A | 7 | 2 | 0.03 | 0.00049 |
| | GO:0044237 | cellular metabolic process | 21131 | 129 | 102.62 | 0.00052 |
| | GO:0019222 | regulation of metabolic process | 7312 | 55 | 35.51 | 0.00057 |

| | | | | | | |
|---|---|---|---|---|---|---|
| | GO:0009739 | response to gibberellin | 379 | 8 | 1.84 | 0.00058 |
| | GO:0006558 | L-phenylalanine metabolic process | 37 | 3 | 0.18 | 0.00078 |
| | GO:0043446 | cellular alkane metabolic process | 37 | 3 | 0.18 | 0.00078 |
| | GO:0043447 | alkane biosynthetic process | 37 | 3 | 0.18 | 0.00078 |
| | GO:0010035 | response to inorganic substance | 2439 | 24 | 11.85 | 0.00089 |
| | GO:0006553 | lysine metabolic process | 39 | 3 | 0.19 | 0.00091 |
| | GO:0006355 | regulation of transcription, DNA-templat... | 5588 | 44 | 27.14 | 0.00091 |
| | GO:0055085 | transmembrane transport | 2301 | 23 | 11.17 | 0.00093 |
| | GO:0015784 | GDP-mannose transport | 10 | 2 | 0.05 | 0.00103 |
| | GO:1990570 | GDP-mannose transmembrane transport | 10 | 2 | 0.05 | 0.00103 |
| | GO:0009808 | lignin metabolic process | 322 | 7 | 1.56 | 0.00107 |
| | GO:1903506 | regulation of nucleic acid-templated tra... | 5639 | 44 | 27.39 | 0.00109 |
| | GO:2001141 | regulation of RNA biosynthetic process | 5639 | 44 | 27.39 | 0.00109 |
| | GO:0046185 | aldehyde catabolic process | 42 | 3 | 0.2 | 0.00113 |
| | GO:0010325 | raffinose family oligosaccharide biosynt... | 11 | 2 | 0.05 | 0.00126 |
| | GO:0051171 | regulation of nitrogen compound metaboli... | 6202 | 47 | 30.12 | 0.00133 |
| | GO:0051252 | regulation of RNA metabolic process | 5698 | 44 | 27.67 | 0.00134 |
| | GO:0008150 | biological_process | 46866 | 242 | 227.61 | 0.00136 |
| | GO:0015718 | monocarboxylic acid transport | 99 | 4 | 0.48 | 0.00143 |
| | GO:0080090 | regulation of primary metabolic process | 6773 | 50 | 32.89 | 0.0016 |
| | GO:0010105 | negative regulation of ethylene-activate... | 48 | 3 | 0.23 | 0.00167 |
| | GO:0070298 | negative regulation of phosphorelay sign... | 48 | 3 | 0.23 | 0.00167 |
| | GO:0000023 | maltose metabolic process | 13 | 2 | 0.06 | 0.00177 |
| | GO:0009809 | lignin biosynthetic process | 261 | 6 | 1.27 | 0.00183 |
| | GO:0019219 | regulation of nucleobase-containing comp... | 5809 | 44 | 28.21 | 0.00195 |
| | GO:0009094 | L-phenylalanine biosynthetic process | 14 | 2 | 0.07 | 0.00206 |
| A1_Top_up-regulated genes | GO:0006950 | response to stress | 8362 | 191 | 111.2 | 9.00E-15 |
| | GO:0050896 | response to stimulus | 14686 | 282 | 195.3 | 8.40E-13 |
| | GO:0042221 | response to chemical | 7052 | 161 | 93.78 | 2.70E-12 |
| | GO:1901700 | response to oxygen-containing compound | 3985 | 105 | 53 | 1.60E-11 |
| | GO:0010035 | response to inorganic substance | 2439 | 75 | 32.44 | 2.10E-11 |
| | GO:0009620 | response to fungus | 862 | 39 | 11.46 | 5.50E-11 |
| | GO:0009628 | response to abiotic stimulus | 5496 | 128 | 73.09 | 2.50E-10 |
| | GO:0009408 | response to heat | 554 | 29 | 7.37 | 6.30E-10 |

| | | | | | |
|---|---|---|---|---|---|
| GO:0009605 | response to external stimulus | 3371 | 88 | 44.83 | 1.40E-09 |
| GO:0009607 | response to biotic stimulus | 2657 | 73 | 35.33 | 5.10E-09 |
| GO:0043207 | response to external biotic stimulus | 2606 | 72 | 34.66 | 5.20E-09 |
| GO:0051707 | response to other organism | 2606 | 72 | 34.66 | 5.20E-09 |
| GO:0006979 | response to oxidative stress | 941 | 37 | 12.51 | 7.50E-09 |
| GO:0050832 | defense response to fungus | 662 | 30 | 8.8 | 8.90E-09 |
| GO:0042542 | response to hydrogen peroxide | 174 | 15 | 2.31 | 1.50E-08 |
| GO:0009644 | response to high light intensity | 204 | 16 | 2.71 | 1.90E-08 |
| GO:0009414 | response to water deprivation | 913 | 34 | 12.14 | 1.10E-07 |
| GO:0080169 | cellular response to boron-containing su... | 5 | 4 | 0.07 | 1.50E-07 |
| GO:0009415 | response to water | 932 | 34 | 12.39 | 1.80E-07 |
| GO:0098542 | defense response to other organism | 1745 | 51 | 23.21 | 1.90E-07 |
| GO:0009642 | response to light intensity | 344 | 19 | 4.57 | 2.50E-07 |
| GO:0006952 | defense response | 3448 | 82 | 45.85 | 2.80E-07 |
| GO:0009266 | response to temperature stimulus | 1540 | 46 | 20.48 | 4.20E-07 |
| GO:0001101 | response to acid chemical | 2953 | 71 | 39.27 | 1.30E-06 |
| GO:0009617 | response to bacterium | 1115 | 36 | 14.83 | 1.40E-06 |
| GO:0042742 | defense response to bacterium | 834 | 29 | 11.09 | 3.60E-06 |
| GO:1901141 | regulation of lignin biosynthetic proces... | 50 | 7 | 0.66 | 4.30E-06 |
| GO:0046459 | short-chain fatty acid metabolic process | 10 | 4 | 0.13 | 6.10E-06 |
| GO:0051704 | multi-organism process | 3460 | 77 | 46.01 | 7.60E-06 |
| GO:0009817 | defense response to fungus, incompatible... | 154 | 11 | 2.05 | 7.70E-06 |
| GO:2000762 | regulation of phenylpropanoid metabolic ... | 86 | 8 | 1.14 | 2.00E-05 |
| GO:0010033 | response to organic substance | 5028 | 101 | 66.87 | 2.00E-05 |
| GO:0006970 | response to osmotic stress | 1738 | 45 | 23.11 | 2.20E-05 |
| GO:0031668 | cellular response to extracellular stimu... | 394 | 17 | 5.24 | 2.70E-05 |
| GO:0009814 | defense response, incompatible interacti... | 609 | 22 | 8.1 | 3.00E-05 |
| GO:0010243 | response to organonitrogen compound | 439 | 18 | 5.84 | 3.10E-05 |
| GO:0080029 | cellular response to boron-containing su... | 15 | 4 | 0.2 | 3.80E-05 |
| GO:0009719 | response to endogenous stimulus | 4305 | 88 | 57.25 | 4.00E-05 |
| GO:0010200 | response to chitin | 371 | 16 | 4.93 | 4.60E-05 |
| GO:0009611 | response to wounding | 674 | 23 | 8.96 | 4.70E-05 |
| GO:0010036 | response to boron-containing substance | 16 | 4 | 0.21 | 5.00E-05 |
| GO:0071496 | cellular response to external stimulus | 417 | 17 | 5.55 | 5.50E-05 |

| | | | | | |
|---|---|---|---|---|---|
| GO:0009816 | defense response to bacterium, incompati... | 129 | 9 | 1.72 | 6.10E-05 |
| GO:0000302 | response to reactive oxygen species | 421 | 17 | 5.6 | 6.10E-05 |
| GO:0009991 | response to extracellular stimulus | 468 | 18 | 6.22 | 7.00E-05 |
| GO:0006883 | cellular sodium ion homeostasis | 7 | 3 | 0.09 | 7.90E-05 |
| GO:0006468 | protein phosphorylation | 2011 | 48 | 26.74 | 8.50E-05 |
| GO:0009404 | toxin metabolic process | 137 | 9 | 1.82 | 9.80E-05 |
| GO:0010942 | positive regulation of cell death | 56 | 6 | 0.74 | 1.00E-04 |
| GO:0009725 | response to hormone | 4064 | 82 | 54.05 | 0.00012 |
| GO:0009651 | response to salt stress | 1594 | 40 | 21.2 | 0.00012 |
| GO:1900376 | regulation of secondary metabolite biosy... | 83 | 7 | 1.1 | 0.00012 |
| GO:0001906 | cell killing | 59 | 6 | 0.78 | 0.00013 |
| GO:0031640 | killing of cells of other organism | 59 | 6 | 0.78 | 0.00013 |
| GO:0044364 | disruption of cells of other organism | 59 | 6 | 0.78 | 0.00013 |
| GO:0046686 | response to cadmium ion | 919 | 27 | 12.22 | 0.00013 |
| GO:0019748 | secondary metabolic process | 825 | 25 | 10.97 | 0.00015 |
| GO:1902348 | cellular response to strigolactone | 2 | 2 | 0.03 | 0.00018 |
| GO:0015690 | aluminum cation transport | 9 | 3 | 0.12 | 0.00019 |
| GO:0072512 | trivalent inorganic cation transport | 9 | 3 | 0.12 | 0.00019 |
| GO:0009416 | response to light stimulus | 2025 | 47 | 26.93 | 0.00019 |
| GO:0009407 | toxin catabolic process | 95 | 7 | 1.26 | 0.00029 |
| GO:0090487 | secondary metabolite catabolic process | 95 | 7 | 1.26 | 0.00029 |
| GO:0045087 | innate immune response | 1077 | 29 | 14.32 | 0.00033 |
| GO:0031669 | cellular response to nutrient levels | 315 | 13 | 4.19 | 0.00035 |
| GO:0010227 | floral organ abscission | 46 | 5 | 0.61 | 0.00036 |
| GO:0009314 | response to radiation | 2089 | 47 | 27.78 | 0.00038 |
| GO:0043455 | regulation of secondary metabolic proces... | 133 | 8 | 1.77 | 0.00043 |
| GO:0006812 | cation transport | 1316 | 33 | 17.5 | 0.00046 |
| GO:0010236 | plastoquinone biosynthetic process | 12 | 3 | 0.16 | 0.00047 |
| GO:0060860 | regulation of floral organ abscission | 12 | 3 | 0.16 | 0.00047 |
| GO:0060862 | negative regulation of floral organ absc... | 12 | 3 | 0.16 | 0.00047 |
| GO:0009753 | response to jasmonic acid | 597 | 19 | 7.94 | 0.00049 |
| GO:0006955 | immune response | 1112 | 29 | 14.79 | 0.00055 |
| GO:0006811 | ion transport | 2016 | 45 | 26.81 | 0.00059 |
| GO:0031667 | response to nutrient levels | 385 | 14 | 5.12 | 0.00075 |

| | | | | | | |
|---|---|---|---|---|---|---|
| | GO:0018108 | peptidyl-tyrosine phosphorylation | 15 | 3 | 0.2 | 0.00095 |
| | GO:0018212 | peptidyl-tyrosine modification | 15 | 3 | 0.2 | 0.00095 |
| | GO:0019357 | nicotinate nucleotide biosynthetic proce... | 4 | 2 | 0.05 | 0.00104 |
| | GO:0019358 | nicotinate nucleotide salvage | 4 | 2 | 0.05 | 0.00104 |
| | GO:0045792 | negative regulation of cell size | 4 | 2 | 0.05 | 0.00104 |
| | GO:0046497 | nicotinate nucleotide metabolic process | 4 | 2 | 0.05 | 0.00104 |
| | GO:1902039 | negative regulation of seed dormancy pro... | 4 | 2 | 0.05 | 0.00104 |
| | GO:1901064 | syringal lignin metabolic process | 34 | 4 | 0.45 | 0.00105 |
| | GO:1901066 | syringal lignin biosynthetic process | 34 | 4 | 0.45 | 0.00105 |
| | GO:1901428 | regulation of syringal lignin biosynthet... | 34 | 4 | 0.45 | 0.00105 |
| | GO:1901430 | positive regulation of syringal lignin b... | 34 | 4 | 0.45 | 0.00105 |
| | GO:0080027 | response to herbivore | 87 | 6 | 1.16 | 0.0011 |
| | GO:0007568 | aging | 448 | 15 | 5.96 | 0.00113 |
| | GO:0009939 | positive regulation of gibberellic acid ... | 16 | 3 | 0.21 | 0.00115 |
| | GO:0009838 | abscission | 60 | 5 | 0.8 | 0.00123 |
| | GO:0009267 | cellular response to starvation | 277 | 11 | 3.68 | 0.00134 |
| | GO:1901701 | cellular response to oxygen-containing c... | 1405 | 33 | 18.68 | 0.00137 |
| | GO:0043090 | amino acid import | 37 | 4 | 0.49 | 0.00145 |
| | GO:0006749 | glutathione metabolic process | 127 | 7 | 1.69 | 0.00161 |
| | GO:0006875 | cellular metal ion homeostasis | 327 | 12 | 4.35 | 0.00161 |
| | GO:0016036 | cellular response to phosphate starvatio... | 164 | 8 | 2.18 | 0.00167 |
| | GO:1901334 | lactone metabolic process | 5 | 2 | 0.07 | 0.00172 |
| | GO:1901336 | lactone biosynthetic process | 5 | 2 | 0.07 | 0.00172 |
| | GO:1901600 | strigolactone metabolic process | 5 | 2 | 0.07 | 0.00172 |
| A1_Top_down-regulated genes | GO:0009768 | photosynthesis, light harvesting in phot... | 72 | 18 | 1.37 | 1.50E-15 |
| | GO:0007167 | enzyme linked receptor protein signaling... | 456 | 39 | 8.7 | 1.00E-14 |
| | GO:0007169 | transmembrane receptor protein tyrosine ... | 456 | 39 | 8.7 | 1.00E-14 |
| | GO:0015979 | photosynthesis | 483 | 39 | 9.22 | 6.60E-14 |
| | GO:0007166 | cell surface receptor signaling pathway | 498 | 39 | 9.5 | 1.70E-13 |
| | GO:0009765 | photosynthesis, light harvesting | 96 | 18 | 1.83 | 3.30E-13 |
| | GO:0005976 | polysaccharide metabolic process | 1023 | 56 | 19.52 | 3.40E-12 |
| | GO:0000272 | polysaccharide catabolic process | 448 | 34 | 8.55 | 1.50E-11 |
| | GO:0051275 | beta-glucan catabolic process | 60 | 13 | 1.15 | 9.30E-11 |
| | GO:0005975 | carbohydrate metabolic process | 2386 | 92 | 45.54 | 1.90E-10 |

| | | | | | | |
|---|---|---|---|---|---|---|
| | GO:0019684 | photosynthesis, light reaction | 266 | 23 | 5.08 | 2.40E-09 |
| | GO:0016052 | carbohydrate catabolic process | 690 | 39 | 13.17 | 2.80E-09 |
| | GO:0030245 | cellulose catabolic process | 56 | 11 | 1.07 | 7.90E-09 |
| | GO:0009251 | glucan catabolic process | 122 | 15 | 2.33 | 1.40E-08 |
| | GO:0051273 | beta-glucan metabolic process | 188 | 18 | 3.59 | 2.70E-08 |
| | GO:0007017 | microtubule-based process | 345 | 24 | 6.58 | 7.40E-08 |
| | GO:0030243 | cellulose metabolic process | 161 | 16 | 3.07 | 9.40E-08 |
| | GO:0010583 | response to cyclopentenone | 108 | 13 | 2.06 | 1.60E-07 |
| | GO:0071555 | cell wall organization | 1210 | 51 | 23.09 | 1.80E-07 |
| | GO:0044247 | cellular polysaccharide catabolic proces... | 131 | 14 | 2.5 | 2.40E-07 |
| | GO:0071554 | cell wall organization or biogenesis | 1682 | 63 | 32.1 | 4.20E-07 |
| | GO:0045229 | external encapsulating structure organiz... | 1302 | 52 | 24.85 | 6.80E-07 |
| | GO:0044275 | cellular carbohydrate catabolic process | 188 | 16 | 3.59 | 7.80E-07 |
| | GO:0044042 | glucan metabolic process | 457 | 26 | 8.72 | 1.10E-06 |
| | GO:0006073 | cellular glucan metabolic process | 438 | 24 | 8.36 | 5.10E-06 |
| | GO:0044264 | cellular polysaccharide metabolic proces... | 581 | 28 | 11.09 | 9.80E-06 |
| | GO:0001944 | vasculature development | 56 | 8 | 1.07 | 1.10E-05 |
| | GO:0072358 | cardiovascular system development | 56 | 8 | 1.07 | 1.10E-05 |
| | GO:0072359 | circulatory system development | 56 | 8 | 1.07 | 1.10E-05 |
| | GO:0051258 | protein polymerization | 186 | 14 | 3.55 | 1.50E-05 |
| | GO:0010067 | procambium histogenesis | 18 | 5 | 0.34 | 1.70E-05 |
| | GO:0010065 | primary meristem tissue development | 20 | 5 | 0.38 | 3.10E-05 |
| | GO:0060918 | auxin transport | 252 | 16 | 4.81 | 3.30E-05 |
| | GO:0080027 | response to herbivore | 87 | 9 | 1.66 | 4.40E-05 |
| | GO:0044262 | cellular carbohydrate metabolic process | 914 | 36 | 17.44 | 4.60E-05 |
| | GO:0009914 | hormone transport | 261 | 16 | 4.98 | 5.00E-05 |
| | GO:0045488 | pectin metabolic process | 320 | 18 | 6.11 | 5.30E-05 |
| | GO:0010393 | galacturonan metabolic process | 329 | 18 | 6.28 | 7.60E-05 |
| | GO:0009554 | megasporogenesis | 24 | 5 | 0.46 | 7.90E-05 |
| | GO:0045490 | pectin catabolic process | 218 | 14 | 4.16 | 8.80E-05 |
| | GO:0044000 | movement in host | 39 | 6 | 0.74 | 9.10E-05 |
| | GO:0046739 | transport of virus in multicellular host | 39 | 6 | 0.74 | 9.10E-05 |
| | GO:0051814 | movement in other organism involved in s... | 39 | 6 | 0.74 | 9.10E-05 |
| | GO:0052126 | movement in host environment | 39 | 6 | 0.74 | 9.10E-05 |

| | | | | | |
|---|---|---|---|---|---|
| GO:0052192 | movement in environment of other organis... | 39 | 6 | 0.74 | 9.10E-05 |
| GO:0034605 | cellular response to heat | 56 | 7 | 1.07 | 9.30E-05 |
| GO:0048869 | cellular developmental process | 2129 | 66 | 40.63 | 9.50E-05 |
| GO:0040011 | locomotion | 98 | 9 | 1.87 | 1.10E-04 |
| GO:0010016 | shoot system morphogenesis | 466 | 22 | 8.89 | 1.10E-04 |
| GO:0080036 | regulation of cytokinin-activated signal... | 26 | 5 | 0.5 | 1.20E-04 |
| GO:0006949 | syncytium formation | 41 | 6 | 0.78 | 1.20E-04 |
| GO:0007018 | microtubule-based movement | 101 | 9 | 1.93 | 0.00014 |
| GO:0044763 | single-organism cellular process | 14260 | 324 | 272.15 | 0.00015 |
| GO:0046741 | transport of virus in host, tissue to ti... | 15 | 4 | 0.29 | 0.00015 |
| GO:0046794 | transport of virus | 43 | 6 | 0.82 | 0.00016 |
| GO:0071669 | plant-type cell wall organization or bio... | 689 | 28 | 13.15 | 0.00019 |
| GO:0017014 | protein nitrosylation | 45 | 6 | 0.86 | 0.00021 |
| GO:0018119 | peptidyl-cysteine S-nitrosylation | 45 | 6 | 0.86 | 0.00021 |
| GO:0010087 | phloem or xylem histogenesis | 244 | 14 | 4.66 | 0.00028 |
| GO:0050896 | response to stimulus | 14686 | 330 | 280.28 | 0.00029 |
| GO:0007155 | cell adhesion | 48 | 6 | 0.92 | 0.00029 |
| GO:0022610 | biological adhesion | 48 | 6 | 0.92 | 0.00029 |
| GO:0015714 | phosphoenolpyruvate transport | 18 | 4 | 0.34 | 0.00033 |
| GO:0080037 | negative regulation of cytokinin-activat... | 8 | 3 | 0.15 | 0.00036 |
| GO:0030155 | regulation of cell adhesion | 2 | 2 | 0.04 | 0.00036 |
| GO:0044766 | multi-organism transport | 50 | 6 | 0.95 | 0.00037 |
| GO:1902579 | multi-organism localization | 50 | 6 | 0.95 | 0.00037 |
| GO:0009653 | anatomical structure morphogenesis | 2240 | 66 | 42.75 | 0.00038 |
| GO:0044699 | single-organism process | 20782 | 449 | 396.62 | 0.00039 |
| GO:0048508 | embryonic meristem development | 71 | 7 | 1.36 | 0.00042 |
| GO:0006091 | generation of precursor metabolites and ... | 728 | 28 | 13.89 | 0.00045 |
| GO:0015713 | phosphoglycerate transport | 20 | 4 | 0.38 | 0.0005 |
| GO:0042873 | aldonate transport | 20 | 4 | 0.38 | 0.0005 |
| GO:0042545 | cell wall modification | 386 | 18 | 7.37 | 0.00053 |
| GO:0033240 | positive regulation of cellular amine me... | 9 | 3 | 0.17 | 0.00053 |
| GO:0090358 | positive regulation of tryptophan metabo... | 9 | 3 | 0.17 | 0.00053 |
| GO:0009828 | plant-type cell wall loosening | 99 | 8 | 1.89 | 0.00064 |
| GO:0009826 | unidimensional cell growth | 714 | 27 | 13.63 | 0.00072 |

| | | | | | | |
|---|---|---|---|---|---|---|
| | GO:0010088 | phloem development | 22 | 4 | 0.42 | 0.00073 |
| | GO:0016049 | cell growth | 1060 | 36 | 20.23 | 0.00076 |
| | GO:0009416 | response to light stimulus | 2025 | 59 | 38.65 | 0.001 |
| | GO:0010410 | hemicellulose metabolic process | 216 | 12 | 4.12 | 0.001 |
| | GO:0007154 | cell communication | 4063 | 105 | 77.54 | 0.00101 |
| | GO:0090357 | regulation of tryptophan metabolic proce... | 11 | 3 | 0.21 | 0.00102 |
| | GO:0006928 | movement of cell or subcellular componen... | 134 | 9 | 2.56 | 0.00113 |
| | GO:0044700 | single organism signaling | 3680 | 96 | 70.23 | 0.00125 |
| | GO:0010817 | regulation of hormone levels | 630 | 24 | 12.02 | 0.00125 |
| | GO:0023052 | signaling | 3685 | 96 | 70.33 | 0.0013 |
| | GO:0006629 | lipid metabolic process | 2395 | 67 | 45.71 | 0.00131 |
| | GO:0010089 | xylem development | 137 | 9 | 2.61 | 0.00132 |
| | GO:0009664 | plant-type cell wall organization | 385 | 17 | 7.35 | 0.00135 |
| | GO:0045493 | xylan catabolic process | 26 | 4 | 0.5 | 0.00141 |
| | GO:0009628 | response to abiotic stimulus | 5496 | 135 | 104.89 | 0.00142 |
| | GO:0042546 | cell wall biogenesis | 492 | 20 | 9.39 | 0.00145 |
| | GO:0009637 | response to blue light | 168 | 10 | 3.21 | 0.00154 |
| | GO:0016051 | carbohydrate biosynthetic process | 678 | 25 | 12.94 | 0.00157 |
| | GO:0051761 | sesquiterpene metabolic process | 67 | 6 | 1.28 | 0.00177 |
| | GO:0051762 | sesquiterpene biosynthetic process | 67 | 6 | 1.28 | 0.00177 |
| | GO:0090354 | regulation of auxin metabolic process | 28 | 4 | 0.53 | 0.00187 |
| | GO:0009314 | response to radiation | 2089 | 59 | 39.87 | 0.00202 |
| A2_Bottom_up-regulated genes | GO:0010200 | response to chitin | 371 | 58 | 7.69 | < 1e-30 |
| | GO:0010243 | response to organonitrogen compound | 439 | 59 | 9.1 | 5.90E-30 |
| | GO:0006952 | defense response | 3448 | 176 | 71.5 | 1.20E-28 |
| | GO:0006950 | response to stress | 8362 | 311 | 173.39 | 1.70E-26 |
| | GO:1901700 | response to oxygen-containing compound | 3985 | 184 | 82.63 | 7.50E-25 |
| | GO:0043207 | response to external biotic stimulus | 2606 | 131 | 54.04 | 1.00E-20 |
| | GO:0051707 | response to other organism | 2606 | 131 | 54.04 | 1.00E-20 |
| | GO:0009607 | response to biotic stimulus | 2657 | 131 | 55.09 | 5.30E-20 |
| | GO:1901698 | response to nitrogen compound | 711 | 60 | 14.74 | 7.00E-20 |
| | GO:0050896 | response to stimulus | 14686 | 439 | 304.52 | 5.70E-19 |
| | GO:0009605 | response to external stimulus | 3371 | 145 | 69.9 | 7.00E-17 |
| | GO:0001101 | response to acid chemical | 2953 | 131 | 61.23 | 2.60E-16 |

| | | | | | |
|---|---|---|---|---|---|
| GO:0098542 | defense response to other organism | 1745 | 91 | 36.18 | 1.50E-15 |
| GO:0009620 | response to fungus | 862 | 59 | 17.87 | 2.20E-15 |
| GO:0042221 | response to chemical | 7052 | 238 | 146.23 | 1.00E-14 |
| GO:0010033 | response to organic substance | 5028 | 184 | 104.26 | 2.00E-14 |
| GO:0009719 | response to endogenous stimulus | 4305 | 164 | 89.27 | 2.60E-14 |
| GO:0051704 | multi-organism process | 3460 | 140 | 71.74 | 3.10E-14 |
| GO:0006464 | cellular protein modification process | 4938 | 180 | 102.39 | 6.00E-14 |
| GO:0036211 | protein modification process | 4938 | 180 | 102.39 | 6.00E-14 |
| GO:0006468 | protein phosphorylation | 2011 | 95 | 41.7 | 1.30E-13 |
| GO:0071229 | cellular response to acid chemical | 1033 | 61 | 21.42 | 4.90E-13 |
| GO:0050832 | defense response to fungus | 662 | 45 | 13.73 | 6.00E-12 |
| GO:0007154 | cell communication | 4063 | 149 | 84.25 | 8.30E-12 |
| GO:0007165 | signal transduction | 3667 | 137 | 76.04 | 1.80E-11 |
| GO:0043412 | macromolecule modification | 5407 | 183 | 112.12 | 2.20E-11 |
| GO:0044700 | single organism signaling | 3680 | 137 | 76.31 | 2.40E-11 |
| GO:0023052 | signaling | 3685 | 137 | 76.41 | 2.60E-11 |
| GO:0002376 | immune system process | 1209 | 63 | 25.07 | 4.10E-11 |
| GO:0071395 | cellular response to jasmonic acid stimu... | 243 | 25 | 5.04 | 6.80E-11 |
| GO:0016310 | phosphorylation | 2619 | 105 | 54.31 | 1.20E-10 |
| GO:0071495 | cellular response to endogenous stimulus | 1926 | 84 | 39.94 | 1.90E-10 |
| GO:0071310 | cellular response to organic substance | 2264 | 94 | 46.94 | 2.00E-10 |
| GO:0006796 | phosphate-containing compound metabolic ... | 3784 | 136 | 78.46 | 2.90E-10 |
| GO:0009867 | jasmonic acid mediated signaling pathway | 241 | 24 | 5 | 3.20E-10 |
| GO:0006793 | phosphorus metabolic process | 3809 | 136 | 78.98 | 4.50E-10 |
| GO:0032870 | cellular response to hormone stimulus | 1898 | 82 | 39.36 | 5.00E-10 |
| GO:0045087 | innate immune response | 1077 | 56 | 22.33 | 5.40E-10 |
| GO:0071446 | cellular response to salicylic acid stim... | 140 | 18 | 2.9 | 8.70E-10 |
| GO:0070887 | cellular response to chemical stimulus | 2616 | 102 | 54.24 | 9.90E-10 |
| GO:1901701 | cellular response to oxygen-containing c... | 1405 | 66 | 29.13 | 1.00E-09 |
| GO:0006955 | immune response | 1112 | 56 | 23.06 | 1.70E-09 |
| GO:0009617 | response to bacterium | 1115 | 56 | 23.12 | 1.90E-09 |
| GO:0009863 | salicylic acid mediated signaling pathwa... | 119 | 16 | 2.47 | 3.80E-09 |
| GO:0009753 | response to jasmonic acid | 597 | 37 | 12.38 | 5.50E-09 |
| GO:0009755 | hormone-mediated signaling pathway | 1777 | 75 | 36.85 | 7.50E-09 |

| | | | | | | |
|---|---|---|---|---|---|---|
| | GO:0035556 | intracellular signal transduction | 988 | 50 | 20.49 | 1.10E-08 |
| | GO:0009873 | ethylene-activated signaling pathway | 486 | 32 | 10.08 | 1.40E-08 |
| | GO:0051716 | cellular response to stimulus | 5913 | 183 | 122.61 | 2.20E-08 |
| | GO:0071369 | cellular response to ethylene stimulus | 508 | 32 | 10.53 | 4.00E-08 |
| | GO:0009611 | response to wounding | 674 | 38 | 13.98 | 4.20E-08 |
| | GO:0010941 | regulation of cell death | 267 | 22 | 5.54 | 5.50E-08 |
| | GO:0042742 | defense response to bacterium | 834 | 43 | 17.29 | 6.90E-08 |
| | GO:0009751 | response to salicylic acid | 469 | 30 | 9.72 | 7.50E-08 |
| | GO:0046777 | protein autophosphorylation | 323 | 24 | 6.7 | 9.90E-08 |
| | GO:0009723 | response to ethylene | 798 | 41 | 16.55 | 1.50E-07 |
| | GO:0031347 | regulation of defense response | 568 | 33 | 11.78 | 1.60E-07 |
| | GO:0048583 | regulation of response to stimulus | 1252 | 55 | 25.96 | 2.20E-07 |
| | GO:0006470 | protein dephosphorylation | 324 | 23 | 6.72 | 4.10E-07 |
| | GO:0008219 | cell death | 892 | 43 | 18.5 | 4.30E-07 |
| | GO:0080134 | regulation of response to stress | 692 | 36 | 14.35 | 6.40E-07 |
| | GO:0000160 | phosphorelay signal transduction system | 578 | 32 | 11.99 | 7.10E-07 |
| | GO:0060548 | negative regulation of cell death | 65 | 10 | 1.35 | 9.00E-07 |
| | GO:0072658 | maintenance of protein location in membr... | 5 | 4 | 0.1 | 9.00E-07 |
| | GO:0072660 | maintenance of protein location in plasm... | 5 | 4 | 0.1 | 9.00E-07 |
| | GO:0009725 | response to hormone | 4064 | 128 | 84.27 | 1.60E-06 |
| | GO:0009737 | response to abscisic acid | 1378 | 56 | 28.57 | 1.90E-06 |
| | GO:0097305 | response to alcohol | 1399 | 56 | 29.01 | 3.10E-06 |
| | GO:0002682 | regulation of immune system process | 291 | 20 | 6.03 | 3.70E-06 |
| | GO:0010185 | regulation of cellular defense response | 13 | 5 | 0.27 | 4.30E-06 |
| | GO:0002252 | immune effector process | 221 | 17 | 4.58 | 4.40E-06 |
| | GO:0072659 | protein localization to plasma membrane | 7 | 4 | 0.15 | 6.10E-06 |
| | GO:0072661 | protein targeting to plasma membrane | 7 | 4 | 0.15 | 6.10E-06 |
| | GO:0090002 | establishment of protein localization to... | 7 | 4 | 0.15 | 6.10E-06 |
| | GO:1990778 | protein localization to cell periphery | 7 | 4 | 0.15 | 6.10E-06 |
| | GO:0006968 | cellular defense response | 14 | 5 | 0.29 | 6.50E-06 |
| | GO:0006979 | response to oxidative stress | 941 | 41 | 19.51 | 9.20E-06 |
| | GO:0002237 | response to molecule of bacterial origin | 108 | 11 | 2.24 | 1.60E-05 |
| | GO:0051245 | negative regulation of cellular defense ... | 9 | 4 | 0.19 | 2.10E-05 |
| | GO:0071215 | cellular response to abscisic acid stimu... | 472 | 25 | 9.79 | 2.40E-05 |

| | | | | | | |
|---|---|---|---|---|---|---|
| | GO:0097306 | cellular response to alcohol | 472 | 25 | 9.79 | 2.40E-05 |
| | GO:0012501 | programmed cell death | 785 | 35 | 16.28 | 2.50E-05 |
| | GO:0043067 | regulation of programmed cell death | 229 | 16 | 4.75 | 2.80E-05 |
| | GO:0016311 | dephosphorylation | 425 | 23 | 8.81 | 3.50E-05 |
| | GO:0010227 | floral organ abscission | 46 | 7 | 0.95 | 4.30E-05 |
| | GO:0070676 | intralumenal vesicle formation | 20 | 5 | 0.41 | 4.50E-05 |
| | GO:0006474 | N-terminal protein amino acid acetylatio... | 21 | 5 | 0.44 | 5.90E-05 |
| | GO:0009626 | plant-type hypersensitive response | 470 | 24 | 9.75 | 6.00E-05 |
| | GO:0034050 | host programmed cell death induced by sy... | 470 | 24 | 9.75 | 6.00E-05 |
| | GO:0000165 | MAPK cascade | 129 | 11 | 2.67 | 8.30E-05 |
| | GO:0000186 | activation of MAPKK activity | 5 | 3 | 0.1 | 8.60E-05 |
| | GO:0033993 | response to lipid | 1877 | 64 | 38.92 | 8.70E-05 |
| | GO:0009814 | defense response, incompatible interacti... | 609 | 28 | 12.63 | 9.50E-05 |
| | GO:0014070 | response to organic cyclic compound | 976 | 39 | 20.24 | 9.70E-05 |
| | GO:0050794 | regulation of cellular process | 10208 | 261 | 211.67 | 0.00011 |
| | GO:0044267 | cellular protein metabolic process | 7153 | 191 | 148.32 | 0.00013 |
| | GO:0023014 | signal transduction by protein phosphory... | 137 | 11 | 2.84 | 0.00014 |
| | GO:0010193 | response to ozone | 94 | 9 | 1.95 | 0.00015 |
| | GO:0050776 | regulation of immune response | 265 | 16 | 5.49 | 0.00015 |
| | GO:0009864 | induced systemic resistance, jasmonic ac... | 41 | 6 | 0.85 | 0.00019 |
| A2_Bottom_down-regulated genes | GO:0009637 | response to blue light | 168 | 13 | 0.83 | 3.40E-12 |
| | GO:0015979 | photosynthesis | 483 | 19 | 2.39 | 6.00E-12 |
| | GO:0019253 | reductive pentose-phosphate cycle | 49 | 8 | 0.24 | 1.20E-10 |
| | GO:0019685 | photosynthesis, dark reaction | 51 | 8 | 0.25 | 1.70E-10 |
| | GO:0015977 | carbon fixation | 55 | 8 | 0.27 | 3.20E-10 |
| | GO:0009416 | response to light stimulus | 2025 | 34 | 10.03 | 6.30E-10 |
| | GO:0009314 | response to radiation | 2089 | 34 | 10.34 | 1.40E-09 |
| | GO:0009628 | response to abiotic stimulus | 5496 | 57 | 27.21 | 5.40E-08 |
| | GO:0010218 | response to far red light | 170 | 9 | 0.84 | 2.10E-07 |
| | GO:0050896 | response to stimulus | 14686 | 106 | 72.72 | 5.90E-06 |
| | GO:0009639 | response to red or far red light | 656 | 14 | 3.25 | 6.10E-06 |
| | GO:0007154 | cell communication | 4063 | 41 | 20.12 | 1.10E-05 |
| | GO:0042221 | response to chemical | 7052 | 60 | 34.92 | 1.60E-05 |
| | GO:0010033 | response to organic substance | 5028 | 47 | 24.9 | 1.60E-05 |

| | | | | | | |
|---|---|---|---|---|---|---|
| | GO:0015843 | methylammonium transport | 2 | 2 | 0.01 | 2.40E-05 |
| | GO:0006833 | water transport | 69 | 5 | 0.34 | 2.50E-05 |
| | GO:0042044 | fluid transport | 69 | 5 | 0.34 | 2.50E-05 |
| | GO:0015696 | ammonium transport | 36 | 4 | 0.18 | 3.10E-05 |
| | GO:0072488 | ammonium transmembrane transport | 36 | 4 | 0.18 | 3.10E-05 |
| | GO:0010114 | response to red light | 142 | 6 | 0.7 | 8.10E-05 |
| | GO:0015840 | urea transport | 19 | 3 | 0.09 | 1.10E-04 |
| | GO:0030308 | negative regulation of cell growth | 20 | 3 | 0.1 | 1.30E-04 |
| | GO:0018149 | peptide cross-linking | 4 | 2 | 0.02 | 1.50E-04 |
| | GO:0018316 | peptide cross-linking via L-cystine | 4 | 2 | 0.02 | 1.50E-04 |
| | GO:0019755 | one-carbon compound transport | 22 | 3 | 0.11 | 1.70E-04 |
| | GO:0051716 | cellular response to stimulus | 5913 | 49 | 29.28 | 2.20E-04 |
| | GO:0010110 | regulation of photosynthesis, dark react... | 5 | 2 | 0.02 | 2.40E-04 |
| | GO:0071454 | cellular response to anoxia | 5 | 2 | 0.02 | 2.40E-04 |
| | GO:0080152 | regulation of reductive pentose-phosphat... | 5 | 2 | 0.02 | 2.40E-04 |
| | GO:0080153 | negative regulation of reductive pentose... | 5 | 2 | 0.02 | 2.40E-04 |
| | GO:0045893 | positive regulation of transcription, DN... | 613 | 11 | 3.04 | 2.70E-04 |
| | GO:1902680 | positive regulation of RNA biosynthetic ... | 613 | 11 | 3.04 | 2.70E-04 |
| | GO:1903508 | positive regulation of nucleic acid-temp... | 613 | 11 | 3.04 | 2.70E-04 |
| | GO:0050794 | regulation of cellular process | 10208 | 74 | 50.54 | 2.80E-04 |
| | GO:0051254 | positive regulation of RNA metabolic pro... | 630 | 11 | 3.12 | 3.50E-04 |
| | GO:0042126 | nitrate metabolic process | 68 | 4 | 0.34 | 3.70E-04 |
| | GO:0042128 | nitrate assimilation | 68 | 4 | 0.34 | 3.70E-04 |
| | GO:0019684 | photosynthesis, light reaction | 266 | 7 | 1.32 | 3.90E-04 |
| | GO:0009889 | regulation of biosynthetic process | 6239 | 50 | 30.89 | 4.10E-04 |
| | GO:0010017 | red or far-red light signaling pathway | 192 | 6 | 0.95 | 4.20E-04 |
| | GO:0009891 | positive regulation of biosynthetic proc... | 753 | 12 | 3.73 | 4.30E-04 |
| | GO:0071489 | cellular response to red or far red ligh... | 196 | 6 | 0.97 | 4.60E-04 |
| | GO:0009725 | response to hormone | 4064 | 36 | 20.12 | 5.10E-04 |
| | GO:0050789 | regulation of biological process | 11311 | 79 | 56.01 | 5.30E-04 |
| | GO:0010557 | positive regulation of macromolecule bio... | 667 | 11 | 3.3 | 5.50E-04 |
| | GO:0045935 | positive regulation of nucleobase-contai... | 668 | 11 | 3.31 | 5.60E-04 |
| | GO:0009740 | gibberellic acid mediated signaling path... | 206 | 6 | 1.02 | 6.00E-04 |
| | GO:0009744 | response to sucrose | 206 | 6 | 1.02 | 6.00E-04 |

| | | | | | | |
|---|---|---|---|---|---|---|
| | GO:0010628 | positive regulation of gene expression | 674 | 11 | 3.34 | 6.00E-04 |
| | GO:0009853 | photorespiration | 137 | 5 | 0.68 | 6.30E-04 |
| | GO:0070887 | cellular response to chemical stimulus | 2616 | 26 | 12.95 | 6.30E-04 |
| | GO:0010476 | gibberellin mediated signaling pathway | 210 | 6 | 1.04 | 0.00066 |
| | GO:0007165 | signal transduction | 3667 | 33 | 18.16 | 0.00067 |
| | GO:0044700 | single organism signaling | 3680 | 33 | 18.22 | 0.00072 |
| | GO:0023052 | signaling | 3685 | 33 | 18.25 | 0.00073 |
| | GO:0071370 | cellular response to gibberellin stimulu... | 214 | 6 | 1.06 | 0.00073 |
| | GO:0009719 | response to endogenous stimulus | 4305 | 37 | 21.32 | 0.00074 |
| | GO:0048506 | regulation of timing of meristematic pha... | 82 | 4 | 0.41 | 0.00076 |
| | GO:0048510 | regulation of timing of transition from ... | 82 | 4 | 0.41 | 0.00076 |
| | GO:0034285 | response to disaccharide | 216 | 6 | 1.07 | 0.00077 |
| | GO:0042886 | amide transport | 218 | 6 | 1.08 | 0.00081 |
| | GO:0048519 | negative regulation of biological proces... | 1954 | 21 | 9.68 | 0.00081 |
| | GO:0001558 | regulation of cell growth | 145 | 5 | 0.72 | 0.00081 |
| | GO:0031668 | cellular response to extracellular stimu... | 394 | 8 | 1.95 | 0.00085 |
| | GO:0048367 | shoot system development | 2242 | 23 | 11.1 | 0.00086 |
| | GO:2001057 | reactive nitrogen species metabolic proc... | 85 | 4 | 0.42 | 0.00087 |
| | GO:0043094 | cellular metabolic compound salvage | 224 | 6 | 1.11 | 0.00093 |
| | GO:0051173 | positive regulation of nitrogen compound... | 719 | 11 | 3.56 | 0.00102 |
| | GO:0031328 | positive regulation of cellular biosynth... | 721 | 11 | 3.57 | 0.00104 |
| | GO:0065007 | biological regulation | 12478 | 84 | 61.78 | 0.00106 |
| | GO:0045912 | negative regulation of carbohydrate meta... | 10 | 2 | 0.05 | 0.00107 |
| | GO:0071941 | nitrogen cycle metabolic process | 90 | 4 | 0.45 | 0.00107 |
| | GO:0071496 | cellular response to external stimulus | 417 | 8 | 2.06 | 0.00121 |
| | GO:0032055 | negative regulation of translation in re... | 11 | 2 | 0.05 | 0.0013 |
| | GO:0080149 | sucrose induced translational repression | 11 | 2 | 0.05 | 0.0013 |
| | GO:0032870 | cellular response to hormone stimulus | 1898 | 20 | 9.4 | 0.00135 |
| | GO:0006355 | regulation of transcription, DNA-templat... | 5588 | 44 | 27.67 | 0.00136 |
| | GO:0080090 | regulation of primary metabolic process | 6773 | 51 | 33.54 | 0.00144 |
| | GO:0071495 | cellular response to endogenous stimulus | 1926 | 20 | 9.54 | 0.00161 |
| | GO:1903506 | regulation of nucleic acid-templated tra... | 5639 | 44 | 27.92 | 0.00162 |
| | GO:2001141 | regulation of RNA biosynthetic process | 5639 | 44 | 27.92 | 0.00162 |
| | GO:0019740 | nitrogen utilization | 49 | 3 | 0.24 | 0.00187 |

| | | | | | | |
|---|---|---|---|---|---|---|
| | GO:0045926 | negative regulation of growth | 49 | 3 | 0.24 | 0.00187 |
| | GO:0031326 | regulation of cellular biosynthetic proc... | 6183 | 47 | 30.61 | 0.00188 |
| | GO:0051252 | regulation of RNA metabolic process | 5698 | 44 | 28.21 | 0.00198 |
| | GO:0034059 | response to anoxia | 14 | 2 | 0.07 | 0.00214 |
| | GO:0071214 | cellular response to abiotic stimulus | 457 | 8 | 2.26 | 0.00215 |
| | GO:0009767 | photosynthetic electron transport chain | 109 | 4 | 0.54 | 0.00218 |
| | GO:0048523 | negative regulation of cellular process | 1306 | 15 | 6.47 | 0.00239 |
| | GO:0010016 | shoot system morphogenesis | 466 | 8 | 2.31 | 0.00242 |
| | GO:0009409 | response to cold | 1052 | 13 | 5.21 | 0.00248 |
| | GO:0009991 | response to extracellular stimulus | 468 | 8 | 2.32 | 0.00249 |
| | GO:2000112 | regulation of cellular macromolecule bio... | 5959 | 45 | 29.51 | 0.00269 |
| | GO:0040034 | regulation of development, heterochronic | 116 | 4 | 0.57 | 0.00273 |
| | GO:0009643 | photosynthetic acclimation | 16 | 2 | 0.08 | 0.0028 |
| | GO:0019219 | regulation of nucleobase-containing comp... | 5809 | 44 | 28.76 | 0.00285 |
| | GO:0007602 | phototransduction | 120 | 4 | 0.59 | 0.00308 |
| | GO:0009585 | red, far-red light phototransduction | 120 | 4 | 0.59 | 0.00308 |
| | GO:0006351 | transcription, DNA-templated | 5841 | 44 | 28.92 | 0.00316 |
| | GO:0050793 | regulation of developmental process | 1483 | 16 | 7.34 | 0.0032 |
| A2_Upper_up-regulated genes | GO:0043207 | response to external biotic stimulus | 2606 | 37 | 9.69 | 2.10E-12 |
| | GO:0051707 | response to other organism | 2606 | 37 | 9.69 | 2.10E-12 |
| | GO:0009607 | response to biotic stimulus | 2657 | 37 | 9.88 | 3.70E-12 |
| | GO:0006979 | response to oxidative stress | 941 | 22 | 3.5 | 1.00E-11 |
| | GO:0009605 | response to external stimulus | 3371 | 40 | 12.53 | 5.70E-11 |
| | GO:0098542 | defense response to other organism | 1745 | 28 | 6.49 | 9.10E-11 |
| | GO:0009620 | response to fungus | 862 | 20 | 3.21 | 1.10E-10 |
| | GO:0050832 | defense response to fungus | 662 | 17 | 2.46 | 6.30E-10 |
| | GO:0009814 | defense response, incompatible interacti... | 609 | 16 | 2.26 | 1.50E-09 |
| | GO:0006950 | response to stress | 8362 | 64 | 31.09 | 4.50E-09 |
| | GO:0051704 | multi-organism process | 3460 | 37 | 12.87 | 5.70E-09 |
| | GO:0002215 | defense response to nematode | 21 | 5 | 0.08 | 1.30E-08 |
| | GO:0050896 | response to stimulus | 14686 | 90 | 54.61 | 5.20E-08 |
| | GO:0006952 | defense response | 3448 | 35 | 12.82 | 5.70E-08 |
| | GO:0009642 | response to light intensity | 344 | 11 | 1.28 | 8.20E-08 |
| | GO:0009266 | response to temperature stimulus | 1540 | 22 | 5.73 | 8.40E-08 |

| | | | | | |
|---|---|---|---|---|---|
| GO:0009817 | defense response to fungus, incompatible... | 154 | 8 | 0.57 | 1.30E-07 |
| GO:0009611 | response to wounding | 674 | 14 | 2.51 | 2.90E-07 |
| GO:0045087 | innate immune response | 1077 | 17 | 4 | 7.00E-07 |
| GO:0006955 | immune response | 1112 | 17 | 4.13 | 1.10E-06 |
| GO:1901141 | regulation of lignin biosynthetic proces... | 50 | 5 | 0.19 | 1.20E-06 |
| GO:0009408 | response to heat | 554 | 12 | 2.06 | 1.30E-06 |
| GO:0010200 | response to chitin | 371 | 10 | 1.38 | 1.50E-06 |
| GO:0002376 | immune system process | 1209 | 17 | 4.5 | 3.30E-06 |
| GO:0015690 | aluminum cation transport | 9 | 3 | 0.03 | 4.20E-06 |
| GO:0072512 | trivalent inorganic cation transport | 9 | 3 | 0.03 | 4.20E-06 |
| GO:0010243 | response to organonitrogen compound | 439 | 10 | 1.63 | 6.70E-06 |
| GO:0043455 | regulation of secondary metabolic proces... | 133 | 6 | 0.49 | 1.10E-05 |
| GO:0009751 | response to salicylic acid | 469 | 10 | 1.74 | 1.20E-05 |
| GO:0009644 | response to high light intensity | 204 | 7 | 0.76 | 1.30E-05 |
| GO:0035264 | multicellular organism growth | 13 | 3 | 0.05 | 1.40E-05 |
| GO:1900376 | regulation of secondary metabolite biosy... | 83 | 5 | 0.31 | 1.50E-05 |
| GO:1901698 | response to nitrogen compound | 711 | 12 | 2.64 | 1.70E-05 |
| GO:2000762 | regulation of phenylpropanoid metabolic ... | 86 | 5 | 0.32 | 1.80E-05 |
| GO:0009617 | response to bacterium | 1115 | 15 | 4.15 | 2.10E-05 |
| GO:1901700 | response to oxygen-containing compound | 3985 | 32 | 14.82 | 3.10E-05 |
| GO:0012502 | induction of programmed cell death | 3 | 2 | 0.01 | 4.10E-05 |
| GO:0010117 | photoprotection | 20 | 3 | 0.07 | 5.50E-05 |
| GO:0051091 | positive regulation of sequence-specific... | 4 | 2 | 0.01 | 8.20E-05 |
| GO:0009628 | response to abiotic stimulus | 5496 | 38 | 20.44 | 1.30E-04 |
| GO:0009816 | defense response to bacterium, incompati... | 129 | 5 | 0.48 | 1.30E-04 |
| GO:0080169 | cellular response to boron-containing su... | 5 | 2 | 0.02 | 1.40E-04 |
| GO:0000302 | response to reactive oxygen species | 421 | 8 | 1.57 | 2.00E-04 |
| GO:0042542 | response to hydrogen peroxide | 174 | 5 | 0.65 | 5.10E-04 |
| GO:0009416 | response to light stimulus | 2025 | 18 | 7.53 | 6.10E-04 |
| GO:0006915 | apoptotic process | 192 | 5 | 0.71 | 7.90E-04 |
| GO:0009314 | response to radiation | 2089 | 18 | 7.77 | 8.60E-04 |
| GO:0009723 | response to ethylene | 798 | 10 | 2.97 | 8.80E-04 |
| GO:0030001 | metal ion transport | 664 | 9 | 2.47 | 9.20E-04 |
| GO:0019748 | secondary metabolic process | 825 | 10 | 3.07 | 1.13E-03 |

| | | | | | |
|---|---|---|---|---|---|
| GO:0080029 | cellular response to boron-containing su... | 15 | 2 | 0.06 | 1.40E-03 |
| GO:0010036 | response to boron-containing substance | 16 | 2 | 0.06 | 1.59E-03 |
| GO:0009061 | anaerobic respiration | 17 | 2 | 0.06 | 1.80E-03 |
| GO:0010440 | stomatal lineage progression | 17 | 2 | 0.06 | 1.80E-03 |
| GO:0009409 | response to cold | 1052 | 11 | 3.91 | 2.08E-03 |
| GO:0046864 | isoprenoid transport | 20 | 2 | 0.07 | 2.50E-03 |
| GO:0046865 | terpenoid transport | 20 | 2 | 0.07 | 2.50E-03 |
| GO:0009809 | lignin biosynthetic process | 261 | 5 | 0.97 | 3.05E-03 |
| GO:0015692 | lead ion transport | 23 | 2 | 0.09 | 3.31E-03 |
| GO:0010941 | regulation of cell death | 267 | 5 | 0.99 | 3.36E-03 |
| GO:0009646 | response to absence of light | 80 | 3 | 0.3 | 3.37E-03 |
| GO:0010324 | membrane invagination | 1 | 1 | 0 | 3.72E-03 |
| GO:0014070 | response to organic cyclic compound | 976 | 10 | 3.63 | 3.79E-03 |
| GO:0006812 | cation transport | 1316 | 12 | 4.89 | 4.02E-03 |
| GO:0080027 | response to herbivore | 87 | 3 | 0.32 | 4.27E-03 |
| GO:0048446 | petal morphogenesis | 27 | 2 | 0.1 | 4.54E-03 |
| GO:0010035 | response to inorganic substance | 2439 | 18 | 9.07 | 4.57E-03 |
| GO:0044093 | positive regulation of molecular functio... | 291 | 5 | 1.08 | 4.82E-03 |
| GO:0010193 | response to ozone | 94 | 3 | 0.35 | 5.29E-03 |
| GO:0009407 | toxin catabolic process | 95 | 3 | 0.35 | 5.45E-03 |
| GO:0090487 | secondary metabolite catabolic process | 95 | 3 | 0.35 | 5.45E-03 |
| GO:0006869 | lipid transport | 300 | 5 | 1.12 | 5.47E-03 |
| GO:0080168 | abscisic acid transport | 31 | 2 | 0.12 | 5.96E-03 |
| GO:0015718 | monocarboxylic acid transport | 99 | 3 | 0.37 | 6.11E-03 |
| GO:0070417 | cellular response to cold | 102 | 3 | 0.38 | 6.64E-03 |
| GO:0042221 | response to chemical | 7052 | 39 | 26.22 | 6.89E-03 |
| GO:0009651 | response to salt stress | 1594 | 13 | 5.93 | 6.99E-03 |
| GO:1901064 | syringal lignin metabolic process | 34 | 2 | 0.13 | 7.14E-03 |
| GO:1901066 | syringal lignin biosynthetic process | 34 | 2 | 0.13 | 7.14E-03 |
| GO:1901428 | regulation of syringal lignin biosynthet... | 34 | 2 | 0.13 | 7.14E-03 |
| GO:1901430 | positive regulation of syringal lignin b... | 34 | 2 | 0.13 | 7.14E-03 |
| GO:0006457 | protein folding | 595 | 7 | 2.21 | 7.18E-03 |
| GO:0009753 | response to jasmonic acid | 597 | 7 | 2.22 | 7.31E-03 |
| GO:0009808 | lignin metabolic process | 322 | 5 | 1.2 | 7.32E-03 |

| | | | | | | |
|---|---|---|---|---|---|---|
| | GO:0051973 | positive regulation of telomerase activi... | 2 | 1 | 0.01 | 7.42E-03 |
| | GO:0071918 | urea transmembrane transport | 2 | 1 | 0.01 | 7.42E-03 |
| | GO:0006811 | ion transport | 2016 | 15 | 7.5 | 8.72E-03 |
| | GO:0051090 | regulation of sequence-specific DNA bind... | 39 | 2 | 0.15 | 9.31E-03 |
| | GO:0043068 | positive regulation of programmed cell d... | 40 | 2 | 0.15 | 9.78E-03 |
| | GO:1900378 | positive regulation of secondary metabol... | 40 | 2 | 0.15 | 9.78E-03 |
| | GO:0043067 | regulation of programmed cell death | 229 | 4 | 0.85 | 1.08E-02 |
| | GO:0010876 | lipid localization | 355 | 5 | 1.32 | 1.09E-02 |
| | GO:0035435 | phosphate ion transmembrane transport | 3 | 1 | 0.01 | 1.11E-02 |
| | GO:2000573 | positive regulation of DNA biosynthetic ... | 3 | 1 | 0.01 | 1.11E-02 |
| | GO:0006749 | glutathione metabolic process | 127 | 3 | 0.47 | 0.01205 |
| | GO:0042742 | defense response to bacterium | 834 | 8 | 3.1 | 0.01331 |
| | GO:0006970 | response to osmotic stress | 1738 | 13 | 6.46 | 0.01364 |
| | GO:0015698 | inorganic anion transport | 249 | 4 | 0.93 | 0.01432 |
| | GO:0001101 | response to acid chemical | 2953 | 19 | 10.98 | 0.01447 |
| | GO:0009404 | toxin metabolic process | 137 | 3 | 0.51 | 0.01475 |
| A2_Upper_down-regulated genes | GO:0071554 | cell wall organization or biogenesis | 1682 | 16 | 4.47 | 1.10E-05 |
| | GO:0071555 | cell wall organization | 1210 | 12 | 3.21 | 1.00E-04 |
| | GO:0006949 | syncytium formation | 41 | 3 | 0.11 | 1.80E-04 |
| | GO:0045229 | external encapsulating structure organiz... | 1302 | 12 | 3.46 | 2.00E-04 |
| | GO:0005975 | carbohydrate metabolic process | 2386 | 17 | 6.34 | 2.10E-04 |
| | GO:0015979 | photosynthesis | 483 | 7 | 1.28 | 3.30E-04 |
| | GO:0010114 | response to red light | 142 | 4 | 0.38 | 5.80E-04 |
| | GO:0042547 | cell wall modification involved in multi... | 78 | 3 | 0.21 | 1.21E-03 |
| | GO:0010088 | phloem development | 22 | 2 | 0.06 | 1.56E-03 |
| | GO:0009828 | plant-type cell wall loosening | 99 | 3 | 0.26 | 2.39E-03 |
| | GO:0010221 | negative regulation of vernalization res... | 1 | 1 | 0 | 2.66E-03 |
| | GO:0009767 | photosynthetic electron transport chain | 109 | 3 | 0.29 | 3.13E-03 |
| | GO:0010205 | photoinhibition | 33 | 2 | 0.09 | 3.50E-03 |
| | GO:0043155 | negative regulation of photosynthesis, l... | 33 | 2 | 0.09 | 3.50E-03 |
| | GO:1905156 | negative regulation of photosynthesis | 38 | 2 | 0.1 | 4.62E-03 |
| | GO:0006631 | fatty acid metabolic process | 601 | 6 | 1.6 | 5.56E-03 |
| | GO:0006636 | unsaturated fatty acid biosynthetic proc... | 46 | 2 | 0.12 | 6.71E-03 |
| | GO:0009827 | plant-type cell wall modification | 147 | 3 | 0.39 | 7.20E-03 |

| | | | | | | |
|---|---|---|---|---|---|---|
| | GO:0009825 | multidimensional cell growth | 148 | 3 | 0.39 | 7.33E-03 |
| | GO:0019253 | reductive pentose-phosphate cycle | 49 | 2 | 0.13 | 7.59E-03 |
| | GO:0010143 | cutin biosynthetic process | 51 | 2 | 0.14 | 8.20E-03 |
| | GO:0019685 | photosynthesis, dark reaction | 51 | 2 | 0.14 | 8.20E-03 |
| | GO:0015977 | carbon fixation | 55 | 2 | 0.15 | 9.49E-03 |
| | GO:0009628 | response to abiotic stimulus | 5496 | 24 | 14.6 | 1.01E-02 |
| | GO:0042548 | regulation of photosynthesis, light reac... | 57 | 2 | 0.15 | 1.02E-02 |
| | GO:0010226 | response to lithium ion | 4 | 1 | 0.01 | 1.06E-02 |
| | GO:0033559 | unsaturated fatty acid metabolic process | 61 | 2 | 0.16 | 1.16E-02 |
| | GO:0043467 | regulation of generation of precursor me... | 62 | 2 | 0.16 | 1.19E-02 |
| | GO:0009750 | response to fructose | 68 | 2 | 0.18 | 1.42E-02 |
| | GO:0006833 | water transport | 69 | 2 | 0.18 | 1.46E-02 |
| | GO:0042044 | fluid transport | 69 | 2 | 0.18 | 1.46E-02 |
| | GO:0006633 | fatty acid biosynthetic process | 360 | 4 | 0.96 | 1.59E-02 |
| | GO:0016998 | cell wall macromolecule catabolic proces... | 74 | 2 | 0.2 | 1.67E-02 |
| | GO:0080167 | response to karrikin | 367 | 4 | 0.97 | 1.69E-02 |
| | GO:0009831 | plant-type cell wall modification involv... | 76 | 2 | 0.2 | 1.76E-02 |
| | GO:0003156 | regulation of animal organ formation | 7 | 1 | 0.02 | 1.85E-02 |
| | GO:0009739 | response to gibberellin | 379 | 4 | 1.01 | 1.88E-02 |
| | GO:0010109 | regulation of photosynthesis | 80 | 2 | 0.21 | 1.93E-02 |
| | GO:0022900 | electron transport chain | 214 | 3 | 0.57 | 1.97E-02 |
| | GO:0009664 | plant-type cell wall organization | 385 | 4 | 1.02 | 1.98E-02 |
| | GO:0042545 | cell wall modification | 386 | 4 | 1.03 | 1.99E-02 |
| | GO:2000027 | regulation of organ morphogenesis | 9 | 1 | 0.02 | 2.37E-02 |
| | GO:0010087 | phloem or xylem histogenesis | 244 | 3 | 0.65 | 2.76E-02 |
| | GO:0031408 | oxylipin biosynthetic process | 101 | 2 | 0.27 | 2.98E-02 |
| | GO:0009639 | response to red or far red light | 656 | 5 | 1.74 | 3.12E-02 |
| | GO:0000272 | polysaccharide catabolic process | 448 | 4 | 1.19 | 3.20E-02 |
| | GO:0010583 | response to cyclopentenone | 108 | 2 | 0.29 | 3.37E-02 |
| | GO:0019684 | photosynthesis, light reaction | 266 | 3 | 0.71 | 3.43E-02 |
| | GO:0010219 | regulation of vernalization response | 14 | 1 | 0.04 | 3.66E-02 |
| | GO:0046470 | phosphatidylcholine metabolic process | 14 | 1 | 0.04 | 3.66E-02 |
| | GO:0071669 | plant-type cell wall organization or bio... | 689 | 5 | 1.83 | 3.73E-02 |
| | GO:0016052 | carbohydrate catabolic process | 690 | 5 | 1.83 | 3.75E-02 |

| | | | | | |
|---|---|---|---|---|---|
| GO:0031407 | oxylipin metabolic process | 117 | 2 | 0.31 | 3.90E-02 |
| GO:0010248 | establishment or maintenance of transmem... | 15 | 1 | 0.04 | 3.91E-02 |
| GO:0048646 | anatomical structure formation involved ... | 486 | 4 | 1.29 | 4.12E-02 |
| GO:0009749 | response to glucose | 121 | 2 | 0.32 | 4.14E-02 |
| GO:0032787 | monocarboxylic acid metabolic process | 1199 | 7 | 3.18 | 4.15E-02 |
| GO:0009416 | response to light stimulus | 2025 | 10 | 5.38 | 4.41E-02 |
| GO:0055076 | transition metal ion homeostasis | 296 | 3 | 0.79 | 4.47E-02 |
| GO:0006869 | lipid transport | 300 | 3 | 0.8 | 4.62E-02 |
| GO:0010067 | procambium histogenesis | 18 | 1 | 0.05 | 4.68E-02 |
| GO:0097164 | ammonium ion metabolic process | 18 | 1 | 0.05 | 4.68E-02 |
| GO:0044247 | cellular polysaccharide catabolic proces... | 131 | 2 | 0.35 | 4.78E-02 |
| GO:0010600 | regulation of auxin biosynthetic process | 19 | 1 | 0.05 | 4.93E-02 |
| GO:0009853 | photorespiration | 137 | 2 | 0.36 | 5.17E-02 |
| GO:0010065 | primary meristem tissue development | 20 | 1 | 0.05 | 5.18E-02 |
| GO:0009314 | response to radiation | 2089 | 10 | 5.55 | 5.24E-02 |
| GO:0006629 | lipid metabolic process | 2395 | 11 | 6.36 | 5.48E-02 |
| GO:0005976 | polysaccharide metabolic process | 1023 | 6 | 2.72 | 5.60E-02 |
| GO:0009746 | response to hexose | 145 | 2 | 0.39 | 5.72E-02 |
| GO:0044036 | cell wall macromolecule metabolic proces... | 333 | 3 | 0.88 | 5.95E-02 |
| GO:0009554 | megasporogenesis | 24 | 1 | 0.06 | 6.19E-02 |
| GO:0034284 | response to monosaccharide | 153 | 2 | 0.41 | 6.28E-02 |
| GO:0009642 | response to light intensity | 344 | 3 | 0.91 | 6.43E-02 |
| GO:0016042 | lipid catabolic process | 569 | 4 | 1.51 | 6.58E-02 |
| GO:0010876 | lipid localization | 355 | 3 | 0.94 | 6.92E-02 |
| GO:0007267 | cell-cell signaling | 28 | 1 | 0.07 | 7.18E-02 |
| GO:0010412 | mannan metabolic process | 28 | 1 | 0.07 | 7.18E-02 |
| GO:0046355 | mannan catabolic process | 28 | 1 | 0.07 | 7.18E-02 |
| GO:0090354 | regulation of auxin metabolic process | 28 | 1 | 0.07 | 7.18E-02 |
| GO:0009725 | response to hormone | 4064 | 16 | 10.79 | 7.35E-02 |
| GO:0009637 | response to blue light | 168 | 2 | 0.45 | 7.39E-02 |
| GO:0010033 | response to organic substance | 5028 | 19 | 13.35 | 7.42E-02 |
| GO:0010218 | response to far red light | 170 | 2 | 0.45 | 7.54E-02 |
| GO:0072330 | monocarboxylic acid biosynthetic process | 597 | 4 | 1.59 | 7.55E-02 |
| GO:0009871 | jasmonic acid and ethylene-dependent sys... | 30 | 1 | 0.08 | 7.67E-02 |

| | | | | | | |
|---|---|---|---|---|---|---|
| | GO:0046885 | regulation of hormone biosynthetic proce... | 31 | 1 | 0.08 | 7.92E-02 |
| | GO:0070828 | heterochromatin organization | 31 | 1 | 0.08 | 7.92E-02 |
| | GO:0042221 | response to chemical | 7052 | 25 | 18.73 | 7.96E-02 |
| | GO:0010090 | trichome morphogenesis | 186 | 2 | 0.49 | 8.79E-02 |
| | GO:0033356 | UDP-L-arabinose metabolic process | 35 | 1 | 0.09 | 8.89E-02 |
| | GO:0044275 | cellular carbohydrate catabolic process | 188 | 2 | 0.5 | 8.95E-02 |
| | GO:0000162 | tryptophan biosynthetic process | 36 | 1 | 0.1 | 9.13E-02 |
| | GO:0046219 | indolalkylamine biosynthetic process | 36 | 1 | 0.1 | 9.13E-02 |
| | GO:0048645 | animal organ formation | 36 | 1 | 0.1 | 9.13E-02 |
| | GO:0001101 | response to acid chemical | 2953 | 12 | 7.84 | 9.45E-02 |
| | GO:0009414 | response to water deprivation | 913 | 5 | 2.42 | 9.70E-02 |
| | GO:0009644 | response to high light intensity | 204 | 2 | 0.54 | 0.10266 |
| | GO:0009415 | response to water | 932 | 5 | 2.48 | 0.10349 |
| | GO:0009740 | gibberellic acid mediated signaling path... | 206 | 2 | 0.55 | 0.10434 |
| A2_Top_up-regulated genes | GO:0005976 | polysaccharide metabolic process | 1023 | 24 | 5.28 | 1.00E-09 |
| | GO:0000272 | polysaccharide catabolic process | 448 | 16 | 2.31 | 2.10E-09 |
| | GO:0006073 | cellular glucan metabolic process | 438 | 15 | 2.26 | 1.20E-08 |
| | GO:0044042 | glucan metabolic process | 457 | 15 | 2.36 | 2.10E-08 |
| | GO:0009768 | photosynthesis, light harvesting in phot... | 72 | 7 | 0.37 | 1.00E-07 |
| | GO:0071555 | cell wall organization | 1210 | 22 | 6.24 | 4.30E-07 |
| | GO:0044264 | cellular polysaccharide metabolic proces... | 581 | 15 | 3 | 4.60E-07 |
| | GO:0051273 | beta-glucan metabolic process | 188 | 9 | 0.97 | 6.80E-07 |
| | GO:0051275 | beta-glucan catabolic process | 60 | 6 | 0.31 | 7.10E-07 |
| | GO:0009765 | photosynthesis, light harvesting | 96 | 7 | 0.5 | 7.30E-07 |
| | GO:0016052 | carbohydrate catabolic process | 690 | 16 | 3.56 | 7.80E-07 |
| | GO:0005975 | carbohydrate metabolic process | 2386 | 32 | 12.31 | 9.20E-07 |
| | GO:0045229 | external encapsulating structure organiz... | 1302 | 22 | 6.72 | 1.40E-06 |
| | GO:0010088 | phloem development | 22 | 4 | 0.11 | 4.70E-06 |
| | GO:0071554 | cell wall organization or biogenesis | 1682 | 24 | 8.68 | 8.40E-06 |
| | GO:0030245 | cellulose catabolic process | 56 | 5 | 0.29 | 1.10E-05 |
| | GO:0045490 | pectin catabolic process | 218 | 8 | 1.12 | 2.00E-05 |
| | GO:0030243 | cellulose metabolic process | 161 | 7 | 0.83 | 2.20E-05 |
| | GO:0044262 | cellular carbohydrate metabolic process | 914 | 16 | 4.72 | 2.60E-05 |
| | GO:1901575 | organic substance catabolic process | 3081 | 34 | 15.9 | 2.60E-05 |

| | | | | | | |
|---|---|---|---|---|---|---|
| | GO:0009813 | flavonoid biosynthetic process | 296 | 9 | 1.53 | 2.70E-05 |
| | GO:0009057 | macromolecule catabolic process | 1850 | 24 | 9.55 | 3.90E-05 |
| | GO:0009251 | glucan catabolic process | 122 | 6 | 0.63 | 4.40E-05 |
| | GO:0045488 | pectin metabolic process | 320 | 9 | 1.65 | 4.90E-05 |
| | GO:0009628 | response to abiotic stimulus | 5496 | 50 | 28.36 | 5.30E-05 |
| | GO:0009056 | catabolic process | 3337 | 35 | 17.22 | 5.60E-05 |
| | GO:0046741 | transport of virus in host, tissue to ti... | 15 | 3 | 0.08 | 5.90E-05 |
| | GO:0010393 | galacturonan metabolic process | 329 | 9 | 1.7 | 6.00E-05 |
| | GO:0044247 | cellular polysaccharide catabolic proces... | 131 | 6 | 0.68 | 6.50E-05 |
| | GO:0009812 | flavonoid metabolic process | 341 | 9 | 1.76 | 7.90E-05 |
| | GO:0009416 | response to light stimulus | 2025 | 24 | 10.45 | 1.50E-04 |
| | GO:0015979 | photosynthesis | 483 | 10 | 2.49 | 2.30E-04 |
| | GO:0009314 | response to radiation | 2089 | 24 | 10.78 | 2.40E-04 |
| | GO:0044710 | single-organism metabolic process | 8779 | 68 | 45.3 | 2.90E-04 |
| | GO:1902446 | regulation of shade avoidance | 6 | 2 | 0.03 | 3.90E-04 |
| | GO:1902448 | positive regulation of shade avoidance | 6 | 2 | 0.03 | 3.90E-04 |
| | GO:0044275 | cellular carbohydrate catabolic process | 188 | 6 | 0.97 | 4.60E-04 |
| | GO:0019684 | photosynthesis, light reaction | 266 | 7 | 1.37 | 5.00E-04 |
| | GO:0080165 | callose deposition in phloem sieve plate | 7 | 2 | 0.04 | 5.50E-04 |
| | GO:0006421 | asparaginyl-tRNA aminoacylation | 8 | 2 | 0.04 | 7.30E-04 |
| | GO:0042545 | cell wall modification | 386 | 8 | 1.99 | 9.70E-04 |
| | GO:0009250 | glucan biosynthetic process | 220 | 6 | 1.14 | 1.05E-03 |
| | GO:0044000 | movement in host | 39 | 3 | 0.2 | 1.08E-03 |
| | GO:0046739 | transport of virus in multicellular host | 39 | 3 | 0.2 | 1.08E-03 |
| | GO:0051814 | movement in other organism involved in s... | 39 | 3 | 0.2 | 1.08E-03 |
| | GO:0052126 | movement in host environment | 39 | 3 | 0.2 | 1.08E-03 |
| | GO:0052192 | movement in environment of other organis... | 39 | 3 | 0.2 | 1.08E-03 |
| | GO:0006949 | syncytium formation | 41 | 3 | 0.21 | 1.25E-03 |
| | GO:0050896 | response to stimulus | 14686 | 99 | 75.78 | 1.31E-03 |
| | GO:0055114 | oxidation-reduction process | 3234 | 30 | 16.69 | 1.43E-03 |
| | GO:0046794 | transport of virus | 43 | 3 | 0.22 | 1.44E-03 |
| | GO:0097502 | mannosylation | 46 | 3 | 0.24 | 1.75E-03 |
| | GO:0008152 | metabolic process | 25748 | 157 | 132.86 | 2.00E-03 |
| | GO:0044766 | multi-organism transport | 50 | 3 | 0.26 | 2.23E-03 |

| | | | | | | |
|---|---|---|---|---|---|---|
| | GO:1902579 | multi-organism localization | 50 | 3 | 0.26 | 2.23E-03 |
| | GO:0010021 | amylopectin biosynthetic process | 15 | 2 | 0.08 | 2.66E-03 |
| | GO:2000896 | amylopectin metabolic process | 15 | 2 | 0.08 | 2.66E-03 |
| | GO:0010411 | xyloglucan metabolic process | 111 | 4 | 0.57 | 2.70E-03 |
| | GO:0007167 | enzyme linked receptor protein signaling... | 456 | 8 | 2.35 | 2.73E-03 |
| | GO:0007169 | transmembrane receptor protein tyrosine ... | 456 | 8 | 2.35 | 2.73E-03 |
| | GO:0016042 | lipid catabolic process | 569 | 9 | 2.94 | 3.03E-03 |
| | GO:0080086 | stamen filament development | 17 | 2 | 0.09 | 3.43E-03 |
| | GO:0009725 | response to hormone | 4064 | 34 | 20.97 | 3.73E-03 |
| | GO:0051701 | interaction with host | 129 | 4 | 0.67 | 4.61E-03 |
| | GO:0007166 | cell surface receptor signaling pathway | 498 | 8 | 2.57 | 4.63E-03 |
| | GO:0015995 | chlorophyll biosynthetic process | 134 | 4 | 0.69 | 5.27E-03 |
| | GO:0010410 | hemicellulose metabolic process | 216 | 5 | 1.11 | 5.46E-03 |
| | GO:0046148 | pigment biosynthetic process | 309 | 6 | 1.59 | 5.62E-03 |
| | GO:0006508 | proteolysis | 2070 | 20 | 10.68 | 5.74E-03 |
| | GO:0009827 | plant-type cell wall modification | 147 | 4 | 0.76 | 7.28E-03 |
| | GO:0006779 | porphyrin-containing compound biosynthet... | 149 | 4 | 0.77 | 7.63E-03 |
| | GO:0045493 | xylan catabolic process | 26 | 2 | 0.13 | 7.95E-03 |
| | GO:0034637 | cellular carbohydrate biosynthetic proce... | 438 | 7 | 2.26 | 8.09E-03 |
| | GO:0006074 | (1->3)-beta-D-glucan metabolic process | 27 | 2 | 0.14 | 8.55E-03 |
| | GO:0033692 | cellular polysaccharide biosynthetic pro... | 339 | 6 | 1.75 | 8.67E-03 |
| | GO:0009719 | response to endogenous stimulus | 4305 | 34 | 22.21 | 8.80E-03 |
| | GO:0033014 | tetrapyrrole biosynthetic process | 156 | 4 | 0.8 | 8.93E-03 |
| | GO:0010087 | phloem or xylem histogenesis | 244 | 5 | 1.26 | 9.00E-03 |
| | GO:1900384 | regulation of flavonol biosynthetic proc... | 2 | 1 | 0.01 | 1.03E-02 |
| | GO:0010383 | cell wall polysaccharide metabolic proce... | 253 | 5 | 1.31 | 1.04E-02 |
| | GO:0051552 | flavone metabolic process | 31 | 2 | 0.16 | 1.12E-02 |
| | GO:0051553 | flavone biosynthetic process | 31 | 2 | 0.16 | 1.12E-02 |
| | GO:0051554 | flavonol metabolic process | 31 | 2 | 0.16 | 1.12E-02 |
| | GO:0051555 | flavonol biosynthetic process | 31 | 2 | 0.16 | 1.12E-02 |
| | GO:0002684 | positive regulation of immune system pro... | 175 | 4 | 0.9 | 1.32E-02 |
| | GO:0050778 | positive regulation of immune response | 175 | 4 | 0.9 | 1.32E-02 |
| | GO:0006629 | lipid metabolic process | 2395 | 21 | 12.36 | 1.32E-02 |
| | GO:0042440 | pigment metabolic process | 377 | 6 | 1.95 | 1.41E-02 |

| | | | | | | |
|---|---|---|---|---|---|---|
| | GO:0010192 | mucilage biosynthetic process | 35 | 2 | 0.18 | 1.41E-02 |
| | GO:0040011 | locomotion | 98 | 3 | 0.51 | 1.44E-02 |
| | GO:0015994 | chlorophyll metabolic process | 180 | 4 | 0.93 | 1.45E-02 |
| | GO:0009828 | plant-type cell wall loosening | 99 | 3 | 0.51 | 1.48E-02 |
| | GO:0033566 | gamma-tubulin complex localization | 3 | 1 | 0.02 | 1.54E-02 |
| | GO:0010033 | response to organic substance | 5028 | 37 | 25.95 | 1.80E-02 |
| | GO:0009638 | phototropism | 40 | 2 | 0.21 | 0.01819 |
| | GO:0009629 | response to gravity | 196 | 4 | 1.01 | 0.01914 |
| | GO:0048584 | positive regulation of response to stimu... | 408 | 6 | 2.11 | 0.01989 |
| | GO:0045491 | xylan metabolic process | 111 | 3 | 0.57 | 0.02003 |
| | GO:0009607 | response to biotic stimulus | 2657 | 22 | 13.71 | 0.02044 |
| | GO:0006076 | (1->3)-beta-D-glucan catabolic process | 4 | 1 | 0.02 | 0.02048 |
| A2_Top_down-regulated genes | GO:0010200 | response to chitin | 371 | 42 | 4.28 | 1.50E-28 |
| | GO:0050896 | response to stimulus | 14686 | 297 | 169.39 | 2.50E-28 |
| | GO:0006950 | response to stress | 8362 | 205 | 96.45 | 9.60E-28 |
| | GO:0010243 | response to organonitrogen compound | 439 | 42 | 5.06 | 1.30E-25 |
| | GO:0006952 | defense response | 3448 | 111 | 39.77 | 7.30E-23 |
| | GO:1901700 | response to oxygen-containing compound | 3985 | 121 | 45.96 | 7.70E-23 |
| | GO:0009605 | response to external stimulus | 3371 | 107 | 38.88 | 1.50E-21 |
| | GO:0043207 | response to external biotic stimulus | 2606 | 90 | 30.06 | 1.80E-20 |
| | GO:0051707 | response to other organism | 2606 | 90 | 30.06 | 1.80E-20 |
| | GO:0009607 | response to biotic stimulus | 2657 | 91 | 30.65 | 1.90E-20 |
| | GO:0042221 | response to chemical | 7052 | 166 | 81.34 | 5.50E-20 |
| | GO:0010033 | response to organic substance | 5028 | 132 | 57.99 | 1.80E-19 |
| | GO:1901698 | response to nitrogen compound | 711 | 42 | 8.2 | 9.90E-18 |
| | GO:0009719 | response to endogenous stimulus | 4305 | 110 | 49.66 | 2.40E-15 |
| | GO:0001101 | response to acid chemical | 2953 | 84 | 34.06 | 2.90E-14 |
| | GO:0051704 | multi-organism process | 3460 | 91 | 39.91 | 1.90E-13 |
| | GO:0009620 | response to fungus | 862 | 39 | 9.94 | 7.50E-13 |
| | GO:0009628 | response to abiotic stimulus | 5496 | 118 | 63.39 | 2.50E-11 |
| | GO:0006979 | response to oxidative stress | 941 | 38 | 10.85 | 4.20E-11 |
| | GO:0098542 | defense response to other organism | 1745 | 54 | 20.13 | 8.30E-11 |
| | GO:0002376 | immune system process | 1209 | 42 | 13.95 | 4.00E-10 |
| | GO:0009617 | response to bacterium | 1115 | 40 | 12.86 | 4.20E-10 |

| | | | | | | |
|---|---|---|---|---|---|---|
| | GO:0009723 | response to ethylene | 798 | 32 | 9.2 | 1.70E-09 |
| | GO:0009751 | response to salicylic acid | 469 | 24 | 5.41 | 1.90E-09 |
| | GO:0070887 | cellular response to chemical stimulus | 2616 | 65 | 30.17 | 7.00E-09 |
| | GO:0009725 | response to hormone | 4064 | 88 | 46.88 | 9.30E-09 |
| | GO:0009408 | response to heat | 554 | 25 | 6.39 | 1.10E-08 |
| | GO:0009873 | ethylene-activated signaling pathway | 486 | 23 | 5.61 | 1.80E-08 |
| | GO:0071229 | cellular response to acid chemical | 1033 | 35 | 11.92 | 2.20E-08 |
| | GO:0009416 | response to light stimulus | 2025 | 53 | 23.36 | 3.70E-08 |
| | GO:0071369 | cellular response to ethylene stimulus | 508 | 23 | 5.86 | 3.90E-08 |
| | GO:0071310 | cellular response to organic substance | 2264 | 57 | 26.11 | 4.10E-08 |
| | GO:0009266 | response to temperature stimulus | 1540 | 44 | 17.76 | 5.00E-08 |
| | GO:0002252 | immune effector process | 221 | 15 | 2.55 | 5.60E-08 |
| | GO:0042742 | defense response to bacterium | 834 | 30 | 9.62 | 6.20E-08 |
| | GO:0050832 | defense response to fungus | 662 | 26 | 7.64 | 8.80E-08 |
| | GO:0009314 | response to radiation | 2089 | 53 | 24.1 | 9.90E-08 |
| | GO:0007154 | cell communication | 4063 | 84 | 46.86 | 1.60E-07 |
| | GO:0006022 | aminoglycan metabolic process | 74 | 9 | 0.85 | 1.90E-07 |
| | GO:0000160 | phosphorelay signal transduction system | 578 | 23 | 6.67 | 3.90E-07 |
| | GO:0010035 | response to inorganic substance | 2439 | 57 | 28.13 | 4.70E-07 |
| | GO:0043455 | regulation of secondary metabolic proces... | 133 | 11 | 1.53 | 4.70E-07 |
| | GO:0045087 | innate immune response | 1077 | 33 | 12.42 | 5.40E-07 |
| | GO:0002237 | response to molecule of bacterial origin | 108 | 10 | 1.25 | 5.40E-07 |
| | GO:0071446 | cellular response to salicylic acid stim... | 140 | 11 | 1.61 | 7.80E-07 |
| | GO:0006955 | immune response | 1112 | 33 | 12.83 | 1.10E-06 |
| | GO:0009863 | salicylic acid mediated signaling pathwa... | 119 | 10 | 1.37 | 1.30E-06 |
| | GO:1901701 | cellular response to oxygen-containing c... | 1405 | 38 | 16.21 | 1.50E-06 |
| | GO:0019748 | secondary metabolic process | 825 | 27 | 9.52 | 1.70E-06 |
| | GO:0009642 | response to light intensity | 344 | 16 | 3.97 | 3.20E-06 |
| | GO:0010942 | positive regulation of cell death | 56 | 7 | 0.65 | 3.70E-06 |
| | GO:0031347 | regulation of defense response | 568 | 21 | 6.55 | 3.90E-06 |
| | GO:0006026 | aminoglycan catabolic process | 57 | 7 | 0.66 | 4.20E-06 |
| | GO:0006030 | chitin metabolic process | 57 | 7 | 0.66 | 4.20E-06 |
| | GO:0006032 | chitin catabolic process | 57 | 7 | 0.66 | 4.20E-06 |
| | GO:0046348 | amino sugar catabolic process | 57 | 7 | 0.66 | 4.20E-06 |

| | | | | | |
|---|---|---|---|---|---|
| GO:1901072 | glucosamine-containing compound cataboli... | 57 | 7 | 0.66 | 4.20E-06 |
| GO:0009651 | response to salt stress | 1594 | 40 | 18.39 | 5.00E-06 |
| GO:1901071 | glucosamine-containing compound metaboli... | 62 | 7 | 0.72 | 7.50E-06 |
| GO:0080134 | regulation of response to stress | 692 | 23 | 7.98 | 7.80E-06 |
| GO:0010371 | regulation of gibberellin biosynthetic p... | 12 | 4 | 0.14 | 8.10E-06 |
| GO:0060860 | regulation of floral organ abscission | 12 | 4 | 0.14 | 8.10E-06 |
| GO:0060862 | negative regulation of floral organ absc... | 12 | 4 | 0.14 | 8.10E-06 |
| GO:0051716 | cellular response to stimulus | 5913 | 104 | 68.2 | 8.60E-06 |
| GO:0032101 | regulation of response to external stimu... | 150 | 10 | 1.73 | 1.10E-05 |
| GO:0009414 | response to water deprivation | 913 | 27 | 10.53 | 1.10E-05 |
| GO:0035264 | multicellular organism growth | 13 | 4 | 0.15 | 1.20E-05 |
| GO:0032870 | cellular response to hormone stimulus | 1898 | 44 | 21.89 | 1.20E-05 |
| GO:0009755 | hormone-mediated signaling pathway | 1777 | 42 | 20.5 | 1.20E-05 |
| GO:0010193 | response to ozone | 94 | 8 | 1.08 | 1.40E-05 |
| GO:0007165 | signal transduction | 3667 | 71 | 42.3 | 1.50E-05 |
| GO:0080169 | cellular response to boron-containing su... | 5 | 3 | 0.06 | 1.50E-05 |
| GO:0009415 | response to water | 932 | 27 | 10.75 | 1.60E-05 |
| GO:0006970 | response to osmotic stress | 1738 | 41 | 20.05 | 1.60E-05 |
| GO:0044700 | single organism signaling | 3680 | 71 | 42.45 | 1.60E-05 |
| GO:0023052 | signaling | 3685 | 71 | 42.5 | 1.70E-05 |
| GO:0071495 | cellular response to endogenous stimulus | 1926 | 44 | 22.22 | 1.70E-05 |
| GO:0031668 | cellular response to extracellular stimu... | 394 | 16 | 4.54 | 1.80E-05 |
| GO:0009409 | response to cold | 1052 | 29 | 12.13 | 1.90E-05 |
| GO:0033993 | response to lipid | 1877 | 43 | 21.65 | 2.00E-05 |
| GO:0018108 | peptidyl-tyrosine phosphorylation | 15 | 4 | 0.17 | 2.20E-05 |
| GO:0018212 | peptidyl-tyrosine modification | 15 | 4 | 0.17 | 2.20E-05 |
| GO:0009411 | response to UV | 369 | 15 | 4.26 | 3.10E-05 |
| GO:0031349 | positive regulation of defense response | 206 | 11 | 2.38 | 3.10E-05 |
| GO:0009404 | toxin metabolic process | 137 | 9 | 1.58 | 3.30E-05 |
| GO:0014070 | response to organic cyclic compound | 976 | 27 | 11.26 | 3.40E-05 |
| GO:0071496 | cellular response to external stimulus | 417 | 16 | 4.81 | 3.50E-05 |
| GO:0000302 | response to reactive oxygen species | 421 | 16 | 4.86 | 3.90E-05 |
| GO:0009611 | response to wounding | 674 | 21 | 7.77 | 4.90E-05 |
| GO:0050691 | regulation of defense response to virus ... | 19 | 4 | 0.22 | 5.90E-05 |

| | | | | | | |
|---|---|---|---|---|---|---|
| | GO:0009615 | response to virus | 263 | 12 | 3.03 | 6.40E-05 |
| | GO:0043090 | amino acid import | 37 | 5 | 0.43 | 6.50E-05 |
| | GO:0006040 | amino sugar metabolic process | 87 | 7 | 1 | 6.90E-05 |
| | GO:0080027 | response to herbivore | 87 | 7 | 1 | 6.90E-05 |
| | GO:0010117 | photoprotection | 20 | 4 | 0.23 | 7.30E-05 |
| | GO:0009737 | response to abscisic acid | 1378 | 33 | 15.89 | 8.10E-05 |
| | GO:0051607 | defense response to virus | 154 | 9 | 1.78 | 8.20E-05 |
| | GO:0009814 | defense response, incompatible interacti... | 609 | 19 | 7.02 | 0.00011 |
| | GO:0097305 | response to alcohol | 1399 | 33 | 16.14 | 0.00011 |
| | GO:0015690 | aluminum cation transport | 9 | 3 | 0.1 | 0.00012 |

## Supplementary Table 6 Overview of module and traits corresponding through WGCNA

| | sample_location | nitrogen_level | leaves_retained | total_nitrogen | soluble_protein | total_protein | aspartic_acid | glutamate | asparagine | serine | glutamine | histidine | glycine | threonine | arginine | ϒ_aminobutyric_acid | alanine |
|---|---|---|---|---|---|---|---|---|---|---|---|---|---|---|---|---|---|
| magenta | 0.03 | -0.19 | -0.26 | 0.026 | 0.12 | 0.063 | −0.19 | 0.058 | −0.3 | −0.075 | −0.097 | −0.36 | −0.11 | −0.0063 | 0.15 | 0.28 | −0.063 |
| pink | 0.13 | -0.073 | 0.013 | -0.025 | -0.24 | −0.038 | −0.31 | 0.26 | −0.048 | 0.024 | −0.096 | −0.064 | 0.027 | −0.15 | 0.099 | 0.044 | −0.18 |
| red | 0.3 | -0.073 | -0.13 | -0.26 | -0.24 | −0.26 | −0.32 | 0.024 | −0.35 | −0.24 | −0.27 | −0.095 | −0.23 | −0.21 | −0.056 | −0.06 | −0.36 |
| turquoise | 0.41 | -0.16 | -0.072 | -0.32 | -0.24 | −0.27 | −0.45 | −0.38 | −0.54 | −0.51 | −0.5 | −0.2 | −0.5 | −0.5 | −0.31 | −0.2 | −0.38 |
| yellow | 0.4 | -0.073 | -0.034 | -0.35 | -0.23 | −0.33 | −0.26 | −0.52 | −0.56 | −0.56 | −0.42 | −0.021 | −0.53 | −0.47 | −0.33 | −0.28 | −0.38 |
| black | 0.39 | -0.046 | -0.11 | -0.43 | -0.25 | −0.46 | −0.24 | −0.083 | −0.34 | −0.28 | −0.32 | −0.0024 | −0.25 | −0.14 | −0.12 | −0.18 | −0.38 |
| blue | 0.51 | -0.26 | -0.38 | -0.24 | -0.33 | −0.26 | −0.31 | −0.16 | −0.46 | −0.28 | −0.28 | −0.32 | −0.34 | −0.33 | 0.15 | 0.044 | −0.2 |
| purple | 0.35 | -0.28 | -0.42 | -0.16 | -0.21 | −0.21 | −0.14 | −0.1 | −0.3 | −0.16 | −0.19 | −0.11 | −0.23 | −0.13 | 0.25 | 0.074 | −0.097 |
| green | -0.72 | 0.15 | 0.067 | 0.38 | 0.25 | 0.4 | 0.33 | 0.42 | 0.65 | 0.52 | 0.48 | 0.078 | 0.52 | 0.45 | 0.13 | 0.11 | 0.39 |
| greenyellow | -0.79 | -0.054 | 0.024 | 0.25 | 0.21 | 0.36 | −0.037 | 0.18 | 0.38 | 0.21 | 0.12 | −0.13 | 0.23 | 0.18 | −0.23 | −0.06 | 0.17 |
| brown | -0.019 | 0.061 | 0.15 | -0.13 | 0.0037 | −0.12 | 0.11 | −0.33 | −0.086 | −0.24 | −0.074 | 0.25 | −0.18 | −0.094 | −0.28 | −0.23 | −0.1 |
| tan | 0.19 | -0.23 | 0.51 | -0.21 | -0.28 | −0.17 | −0.14 | −0.33 | −0.27 | −0.39 | −0.21 | −0.067 | −0.27 | −0.43 | −0.47 | −0.21 | −0.27 |
| grey | -0.058 | -0.085 | 0.22 | -0.16 | -0.0054 | 0.045 | −0.16 | −0.46 | −0.22 | −0.43 | −0.32 | 0.11 | −0.35 | −0.33 | −0.55 | −0.34 | −0.23 |

| | proline | tyrosine | ammonium_ion | agmatine | valine | methionine | norleucine | tryptophan | isoleucine | leucine | phenylalanine | ornithine | lysine | tyramine | putrescine | phenethylamine | isoamylamine | spermidine |
|---|---|---|---|---|---|---|---|---|---|---|---|---|---|---|---|---|---|---|
| magenta | −0.11 | 0.089 | 0.049 | 0.026 | 0.026 | 0.21 | −0.27 | −0.23 | 0.037 | 0.1 | −0.046 | 0.17 | 0.14 | −0.11 | −0.34 | −0.12 | −0.31 | 0.064 |
| pink | 0.0022 | −0.09 | 0.028 | 0.2 | −0.089 | 0.068 | −0.28 | 0.079 | 0.12 | −0.14 | −0.085 | 0.032 | −0.089 | 0.13 | −0.13 | 0.054 | −0.048 | 0.036 |
| red | −0.14 | −0.11 | −0.13 | 0.25 | −0.15 | 0.19 | −0.49 | 0.11 | 0.27 | −0.14 | −0.35 | −0.17 | −0.14 | 0.057 | −0.49 | −0.3 | −0.4 | −0.28 |
| turquoise | −0.35 | −0.2 | −0.43 | −0.14 | −0.26 | −0.13 | −0.089 | 0.17 | 0.1 | −0.17 | −0.47 | −0.28 | −0.18 | 0.22 | −0.52 | −0.095 | −0.38 | −0.45 |
| yellow | −0.37 | −0.075 | −0.35 | −0.16 | −0.13 | 0.041 | −0.1 | 0.39 | 0.34 | −0.075 | −0.47 | −0.43 | −0.11 | 0.12 | −0.44 | −0.38 | −0.42 | −0.49 |
| black | −0.21 | −0.1 | −0.16 | 0.28 | −0.18 | 0.16 | −0.54 | 0.15 | 0.32 | −0.14 | −0.43 | −0.38 | −0.18 | −0.063 | −0.48 | −0.44 | −0.43 | −0.45 |
| blue | −0.35 | 0.17 | −0.039 | −0.066 | 0.054 | 0.23 | −0.27 | 0.033 | 0.39 | 0.18 | −0.23 | −0.09 | 0.19 | 0.06 | −0.52 | −0.15 | −0.45 | −0.27 |
| purple | −0.29 | 0.33 | 0.11 | −0.0026 | 0.18 | 0.42 | −0.23 | 0.089 | 0.52 | 0.34 | −0.15 | −0.18 | 0.3 | −0.065 | −0.42 | −0.23 | −0.39 | −0.29 |
| green | 0.47 | −0.028 | 0.33 | 0.22 | 0.086 | −0.076 | 0.23 | −0.27 | −0.39 | −0.026 | 0.4 | 0.38 | −0.0065 | −0.15 | 0.64 | 0.31 | 0.63 | 0.56 |
| greenyellow | 0.32 | −0.32 | −0.012 | 0.16 | −0.22 | −0.29 | 0.28 | −0.23 | −0.58 | −0.27 | 0.15 | 0.27 | −0.27 | −0.1 | 0.33 | 0.3 | 0.5 | 0.31 |
| brown | −0.072 | −0.028 | −0.15 | −0.059 | −0.032 | 0.00033 | 0.15 | 0.32 | 0.12 | −0.038 | −0.17 | −0.32 | −0.088 | −0.011 | 0.037 | −0.23 | 0.014 | −0.23 |
| tan | −0.36 | −0.36 | −0.35 | 0.0012 | −0.31 | −0.3 | 0.12 | −0.052 | 0.063 | −0.42 | −0.45 | −0.38 | −0.36 | 0.0057 | −0.067 | −0.13 | 0.075 | −0.32 |
| grey | −0.15 | −0.3 | −0.44 | −0.18 | −0.29 | −0.3 | 0.34 | 0.31 | −0.15 | −0.27 | −0.35 | −0.33 | −0.31 | 0.15 | −0.11 | −0.0076 | 0.035 | −0.34 |

# Supplementary Table 7  Overview of module trait *p*-value through WGCNA

| | sample_location | nitrogen_level | leaves_retained | total_nitrogen | soluble_protein | total_protein | aspartic_acid | glutamate | asparagine | serine | glutamine | histidine | glycine | threonine | arginine | ϒ_aminobutyric_acid | alanine |
|---|---|---|---|---|---|---|---|---|---|---|---|---|---|---|---|---|---|
| magenta | 0.9 | 0.3 | 0.2 | 0.9 | 0.5 | 0.7 | 0.3 | 0.8 | 0.1 | 0.7 | 0.6 | 0.04 | 0.6 | 1 | 0.4 | 0.1 | 0.7 |
| pink | 0.5 | 0.7 | 0.9 | 0.9 | 0.2 | 0.8 | 0.09 | 0.1 | 0.8 | 0.9 | 0.6 | 0.7 | 0.9 | 0.4 | 0.6 | 0.8 | 0.3 |
| red | 0.09 | 0.7 | 0.5 | 0.2 | 0.2 | 0.1 | 0.07 | 0.9 | 0.05 | 0.2 | 0.1 | 0.6 | 0.2 | 0.2 | 0.8 | 0.7 | 0.04 |
| turquoise | 0.02 | 0.4 | 0.7 | 0.07 | 0.2 | 0.1 | 0.009 | 0.03 | 0.002 | 0.003 | 0.003 | 0.3 | 0.003 | 0.004 | 0.08 | 0.3 | 0.03 |
| yellow | 0.02 | 0.7 | 0.9 | 0.05 | 0.2 | 0.07 | 0.1 | 0.002 | 8e−04 | 9e−04 | 0.02 | 0.9 | 0.002 | 0.007 | 0.07 | 0.1 | 0.03 |
| black | 0.03 | 0.8 | 0.5 | 0.01 | 0.2 | 0.008 | 0.2 | 0.6 | 0.05 | 0.1 | 0.07 | 1 | 0.2 | 0.4 | 0.5 | 0.3 | 0.03 |
| blue | 0.003 | 0.2 | 0.03 | 0.2 | 0.06 | 0.1 | 0.09 | 0.4 | 0.008 | 0.1 | 0.1 | 0.08 | 0.06 | 0.07 | 0.4 | 0.8 | 0.3 |
| purple | 0.05 | 0.1 | 0.02 | 0.4 | 0.2 | 0.3 | 0.4 | 0.6 | 0.09 | 0.4 | 0.3 | 0.5 | 0.2 | 0.5 | 0.2 | 0.7 | 0.6 |
| green | 3e−06 | 0.4 | 0.7 | 0.03 | 0.2 | 0.02 | 0.06 | 0.02 | 5e−05 | 0.002 | 0.005 | 0.7 | 0.002 | 0.009 | 0.5 | 0.6 | 0.03 |
| greenyellow | 6e−08 | 0.8 | 0.9 | 0.2 | 0.3 | 0.05 | 0.8 | 0.3 | 0.03 | 0.2 | 0.5 | 0.5 | 0.2 | 0.3 | 0.2 | 0.7 | 0.3 |
| brown | 0.9 | 0.7 | 0.4 | 0.5 | 1 | 0.5 | 0.6 | 0.06 | 0.6 | 0.2 | 0.7 | 0.2 | 0.3 | 0.6 | 0.1 | 0.2 | 0.6 |
| tan | 0.3 | 0.2 | 0.003 | 0.2 | 0.1 | 0.3 | 0.4 | 0.06 | -0.1 | 0.03 | 0.2 | 0.7 | 0.1 | 0.01 | 0.007 | 0.3 | 0.1 |
| grey | 0.8 | 0.6 | 0.2 | 0.4 | 1 | 0.8 | 0.4 | 0.008 | 0.2 | 0.01 | 0.08 | 0.5 | 0.05 | 0.07 | 0.001 | 0.06 | 0.2 |

| | proline | tyrosine | ammonium_ion | agmatine | valine | methionine | norleucine | tryptophan | isoleucine | leucine | phenylalanine | ornithine | lysine | tyramine | putrescine | phenethylamine | isoamylamine | spermidine |
|---|---|---|---|---|---|---|---|---|---|---|---|---|---|---|---|---|---|---|
| magenta | 0.6 | 0.6 | 0.8 | 0.9 | 0.9 | 0.3 | 0.1 | 0.2 | 0.8 | 0.6 | 0.8 | 0.4 | 0.5 | 0.5 | 0.06 | 0.5 | 0.08 | 0.7 |
| pink | 1 | 0.6 | 0.9 | 0.3 | 0.6 | 0.7 | 0.1 | 0.7 | 0.5 | 0.5 | 0.6 | 0.9 | 0.6 | 0.5 | 0.5 | 0.8 | 0.8 | 0.8 |
| red | 0.5 | 0.5 | 0.5 | 0.2 | 0.4 | 0.3 | 0.005 | 0.5 | 0.1 | 0.4 | 0.05 | 0.4 | 0.5 | 0.8 | 0.005 | 0.1 | 0.02 | 0.1 |
| turquoise | 0.05 | 0.3 | 0.02 | 0.4 | 0.2 | 0.5 | 0.6 | 0.3 | 0.6 | 0.3 | 0.007 | 0.1 | 0.3 | 0.2 | 0.003 | 0.6 | 0.03 | 0.009 |
| yellow | 0.04 | 0.7 | 0.05 | 0.4 | 0.5 | 0.8 | 0.6 | 0.03 | 0.06 | 0.7 | 0.007 | 0.01 | 0.5 | 0.5 | 0.01 | 0.03 | 0.02 | 0.004 |
| black | 0.2 | 0.6 | 0.4 | 0.1 | 0.3 | 0.4 | 0.002 | 0.4 | 0.07 | 0.4 | 0.01 | 0.03 | 0.3 | 0.7 | 0.006 | 0.001 | 0.01 | 0.009 |
| blue | 0.05 | 0.4 | 0.8 | 0.7 | 0.8 | 0.2 | 0.1 | 0.9 | 0.03 | 0.3 | 0.2 | −0.6 | 0.3 | 0.7 | 0.002 | 0.4 | 0.01 | 0.1 |
| purple | 0.1 | 0.06 | 0.6 | 1 | 0.3 | 0.02 | 0.2 | 0.6 | 0.002 | 0.06 | 0.4 | 0.3 | 0.09 | 0.7 | 0.02 | 0.2 | 0.03 | 0.1 |
| green | 0.006 | 0.9 | 0.06 | 0.2 | 0.6 | 0.7 | 0.2 | 0.1 | 0.03 | 0.9 | 0.007 | 0.03 | 1 | 0.4 | 7e−05 | 0.08 | 1e−04 | 8e−04 |
| greenyellow | 0.07 | 0.07 | 0.9 | 0.4 | 0.2 | 0.1 | 0.1 | 0.2 | 5e−04 | 0.1 | 0.4 | 0.1 | 0.1 | 0.6 | 0.07 | 0.1 | 0.003 | 0.08 |
| brown | 0.7 | 0.9 | 0.4 | 0.7 | 0.9 | 1 | 0.4 | 0.07 | 0.5 | 0.8 | 0.3 | 0.07 | 0.6 | 1 | 0.8 | 0.2 | 0.9 | 0.2 |
| tan | 0.04 | 0.04 | 0.05 | 1 | 0.08 | 0.1 | 0.5 | 0.8 | 0.7 | 0.02 | 0.01 | 0.03 | 0.04 | 1 | 0.7 | 0.5 | 0.7 | 0.07 |
| grey | 0.4 | 0.1 | 0.01 | 0.3 | 0.1 | 0.09 | 0.06 | 0.09 | 0.4 | 0.1 | 0.05 | 0.07 | 0.09 | 0.4 | 0.6 | 1 | 0.8 | 0.05 |

**Supplementary Table 8 GO enrichment analysis of WGCNA module genes**

| Moudle | GO-ID | *P*-value | Corrected *P*-value | Number of DEGs with GO terms | Number of genes with GO terms | Description of GO annotation |
|---|---|---|---|---|---|---|
| Green | GO:1901566 | 1.50E-24 | 1.50E-24 | 258 | 3534 | organonitrogen compound biosynthetic process |
| | GO:0017014 | 3.80E-12 | 3.80E-12 | 16 | 45 | protein nitrosylation |
| | GO:0018119 | 3.80E-12 | 3.80E-12 | 16 | 45 | peptidyl-cysteine S-nitrosylation |
| | GO:0006520 | 4.10E-09 | 4.10E-09 | 78 | 1015 | cellular amino acid metabolic process |
| | GO:1901564 | 3.10E-08 | 3.10E-08 | 493 | 10444 | organonitrogen compound metabolic process |
| | GO:1901605 | 6.00E-08 | 6.00E-08 | 57 | 696 | alpha-amino acid metabolic process |
| | GO:0008652 | 1.40E-06 | 1.40E-06 | 40 | 462 | cellular amino acid biosynthetic process |
| | GO:1901607 | 3.90E-05 | 3.90E-05 | 33 | 406 | alpha-amino acid biosynthetic process |
| | GO:0000413 | 9.20E-05 | 9.20E-05 | 11 | 73 | protein peptidyl-prolyl isomerization |
| | GO:0006091 | 3.35E-41 | 3.53E-39 | 122 | 744 | generation of precursor metabolites and energy |
| | GO:0018193 | 0.0006 | 0.0006 | 43 | 668 | peptidyl-amino acid modification |
| | GO:0009066 | 0.00065 | 0.00065 | 15 | 151 | aspartate family amino acid metabolic process |
| | GO:1901606 | 0.00187 | 0.00187 | 14 | 151 | alpha-amino acid catabolic process |
| | GO:0015977 | 6.2212E-16 | 3.2228E-14 | 21 | 55 | carbon fixation |
| | GO:0009082 | 0.00254 | 0.00254 | 8 | 63 | branched-chain amino acid biosynthetic process |
| | GO:0006575 | 0.00281 | 0.00281 | 19 | 246 | cellular modified amino acid metabolic process |
| | GO:0009064 | 0.00376 | 0.00376 | 16 | 198 | glutamine family amino acid metabolic process |
| | GO:0009063 | 0.00399 | 0.00399 | 14 | 164 | cellular amino acid catabolic process |
| | GO:0003333 | 0.00489 | 0.00489 | 9 | 85 | amino acid transmembrane transport |
| | GO:0009071 | 0.00665 | 0.00665 | 5 | 32 | serine family amino acid catabolic process |
| | GO:0009067 | 0.00749 | 0.00749 | 10 | 107 | aspartate family amino acid biosynthetic process |
| | GO:0006434 | 0.00817 | 0.00817 | 2 | 4 | seryl-tRNA aminoacylation |
| | GO:0009072 | 0.00915 | 0.00915 | 15 | 199 | aromatic amino acid family metabolic process |
| | GO:0019740 | 0.01018 | 0.01018 | 6 | 49 | nitrogen utilization |
| | GO:0015807 | 0.0112 | 0.0112 | 6 | 50 | L-amino acid transport |
| | GO:1902475 | 0.0112 | 0.0112 | 6 | 50 | L-alpha-amino acid transmembrane transport |
| | GO:0051952 | 0.01166 | 0.01166 | 3 | 13 | regulation of amine transport |
| | GO:0051955 | 0.01166 | 0.01166 | 3 | 13 | regulation of amino acid transport |
| | GO:0044270 | 0.0395 | 0.0395 | 28 | 518 | cellular nitrogen compound catabolic process |

| | | | | | |
|---|---|---|---|---|---|
| GO:0016168 | 5.10E-26 | 5.10E-26 | 34 | 87 | chlorophyll binding |
| GO:0031409 | 1.50E-24 | 1.50E-24 | 30 | 70 | pigment binding |
| GO:0015179 | 0.01148 | 0.01148 | 6 | 51 | L-amino acid transmembrane transporter activity |
| GO:0045309 | 0.01297 | 0.01297 | 5 | 38 | protein phosphorylated amino acid binding |
| GO:0016597 | 0.01589 | 0.01589 | 9 | 104 | amino acid binding |
| GO:0008836 | 0.02578 | 0.02578 | 2 | 7 | diaminopimelate decarboxylase activity |
| GO:0015185 | 0.0513 | 0.0513 | 2 | 10 | gamma-aminobutyric acid transmembrane transporter activity |
| GO:0044435 | 2.40E-235 | 0.00E+00 | 592 | 3297 | plastid part |
| GO:0009579 | 1.27E-186 | 0.00E+00 | 353 | 1378 | thylakoid |
| GO:0031976 | 1.33E-154 | 0.00E+00 | 294 | 1144 | plastid thylakoid |
| GO:0009534 | 7.63E-154 | 0.00E+00 | 293 | 1142 | chloroplast thylakoid |
| GO:0009507 | 1.40E-151 | 0.00E+00 | 841 | 8906 | chloroplast |
| GO:0009536 | 2.74E-151 | 0.00E+00 | 850 | 9080 | plastid |
| GO:0031984 | 4.57E-147 | 0.00E+00 | 296 | 1231 | organelle subcompartment |
| GO:0009532 | 2.08E-142 | 0.00E+00 | 334 | 1651 | plastid stroma |
| GO:0009570 | 1.23E-141 | 0.00E+00 | 330 | 1619 | chloroplast stroma |
| GO:0009526 | 1.95E-131 | 0.00E+00 | 325 | 1693 | plastid envelope |
| GO:0009941 | 1.78E-129 | 0.00E+00 | 317 | 1633 | chloroplast envelope |
| GO:0044436 | 9.49E-125 | 0.00E+00 | 253 | 1055 | thylakoid part |
| GO:0055035 | 1.84E-120 | 0.00E+00 | 229 | 883 | plastid thylakoid membrane |
| GO:0009535 | 2.77E-120 | 0.00E+00 | 228 | 876 | chloroplast thylakoid membrane |
| GO:0034357 | 6.81E-117 | 0.00E+00 | 236 | 977 | photosynthetic membrane |
| GO:0042651 | 2.99E-113 | 0.00E+00 | 231 | 967 | thylakoid membrane |
| GO:0015979 | 6.25E-111 | 0.00E+00 | 161 | 436 | photosynthesis |
| GO:0031975 | 1.15E-85 | 1.92E-83 | 347 | 2762 | envelope |
| GO:0031967 | 4.05E-83 | 6.40E-81 | 342 | 2751 | organelle envelope |
| GO:0009521 | 1.18E-73 | 1.78E-71 | 81 | 147 | photosystem |
| GO:0044446 | 4.54E-69 | 6.51E-67 | 788 | 11341 | intracellular organelle part |
| GO:0044422 | 1.07E-68 | 1.47E-66 | 788 | 11362 | organelle part |
| GO:0019684 | 3.09E-57 | 4.07E-55 | 83 | 226 | photosynthesis, light reaction |
| GO:0044444 | 9.26E-57 | 1.17E-54 | 1217 | 22100 | cytoplasmic part |
| GO:0009543 | 1.22E-49 | 1.43E-47 | 66 | 160 | chloroplast thylakoid lumen |

| | | | | | |
|---|---|---|---|---|---|
| GO:0031978 | 1.22E-49 | 1.43E-47 | 66 | 160 | plastid thylakoid lumen |
| GO:0009523 | 1.53E-48 | 1.73E-46 | 54 | 101 | photosystem II |
| GO:0031977 | 3.97E-48 | 4.32E-46 | 69 | 186 | thylakoid lumen |
| GO:0005737 | 3.97E-36 | 4.04E-34 | 1337 | 27075 | cytoplasm |
| GO:0009522 | 4.77E-30 | 4.71E-28 | 34 | 67 | photosystem I |
| GO:0010287 | 1.53E-28 | 1.47E-26 | 54 | 212 | plastoglobule |
| GO:0009765 | 2.30E-28 | 2.14E-26 | 38 | 96 | photosynthesis, light harvesting |
| GO:0030095 | 4.30E-26 | 3.88E-24 | 24 | 35 | chloroplast photosystem II |
| GO:0009768 | 9.54E-25 | 8.15E-23 | 31 | 72 | photosynthesis, light harvesting in photosystem I |
| GO:0005576 | 9.99E-25 | 8.31E-23 | 348 | 5129 | extracellular region |
| GO:0048046 | 5.19E-24 | 4.21E-22 | 127 | 1198 | apoplast |
| GO:0009654 | 6.08E-23 | 4.69E-21 | 24 | 43 | oxygen evolving complex |
| GO:0015995 | 7.35E-23 | 5.53E-21 | 33 | 94 | chlorophyll biosynthetic process |
| GO:0016020 | 5.79E-22 | 4.25E-20 | 982 | 19707 | membrane |
| GO:0032544 | 4.87E-21 | 3.50E-19 | 18 | 24 | plastid translation |
| GO:0015994 | 1.03E-20 | 7.22E-19 | 36 | 131 | chlorophyll metabolic process |
| GO:0009657 | 1.21E-20 | 8.29E-19 | 67 | 453 | plastid organization |
| GO:0006779 | 1.48E-20 | 9.96E-19 | 33 | 109 | porphyrin biosynthetic process |
| GO:0019843 | 8.97E-20 | 5.90E-18 | 36 | 139 | rRNA binding |
| GO:0033014 | 1.27E-19 | 8.22E-18 | 33 | 116 | tetrapyrrole biosynthetic process |
| GO:0006778 | 2.17E-18 | 1.37E-16 | 36 | 152 | porphyrin metabolic process |
| GO:0033013 | 4.31E-18 | 2.67E-16 | 36 | 155 | tetrapyrrole metabolic process |
| GO:0042180 | 1.41E-17 | 8.54E-16 | 188 | 2507 | cellular ketone metabolic process |
| GO:0009547 | 5.19E-17 | 3.10E-15 | 14 | 18 | plastid ribosome |
| GO:0043436 | 1.36E-16 | 7.81E-15 | 182 | 2453 | oxoacid metabolic process |
| GO:0019752 | 1.36E-16 | 7.81E-15 | 182 | 2453 | carboxylic acid metabolic process |
| GO:0006082 | 1.65E-16 | 9.33E-15 | 182 | 2458 | organic acid metabolic process |
| GO:0044281 | 2.49E-16 | 1.38E-14 | 321 | 5227 | small molecule metabolic process |
| GO:0018130 | 4.22E-16 | 2.30E-14 | 51 | 344 | heterocycle biosynthetic process |
| GO:0044271 | 6.19E-16 | 3.22E-14 | 106 | 1150 | cellular nitrogen compound biosynthetic process |
| GO:0071704 | 6.22E-16 | 3.22E-14 | 21 | 55 | organic substance metabolic process |

| | | | | | |
|---|---|---|---|---|---|
| GO:0019253 | 6.51E-16 | 3.32E-14 | 20 | 49 | reductive pentose-phosphate cycle |
| GO:0046501 | 7.18E-16 | 3.55E-14 | 15 | 24 | protoporphyrinogen IX metabolic process |
| GO:0006782 | 7.18E-16 | 3.55E-14 | 15 | 24 | protoporphyrinogen IX biosynthetic process |
| GO:0051186 | 1.25E-15 | 6.08E-14 | 90 | 906 | cofactor metabolic process |
| GO:0019685 | 1.65E-15 | 7.92E-14 | 20 | 51 | photosynthesis, dark reaction |
| GO:0046148 | 2.56E-15 | 1.21E-13 | 42 | 252 | pigment biosynthetic process |
| GO:0009538 | 4.68E-15 | 2.18E-13 | 14 | 22 | photosystem I reaction center |
| GO:0051188 | 5.32E-15 | 2.44E-13 | 58 | 457 | cofactor biosynthetic process |
| GO:0008152 | 1.79E-14 | 8.08E-13 | 1108 | 23950 | metabolic process |
| GO:0042170 | 2.71E-14 | 1.21E-12 | 59 | 488 | plastid membrane |
| GO:0031969 | 2.91E-14 | 1.28E-12 | 58 | 475 | chloroplast membrane |
| GO:0042440 | 4.99E-14 | 2.16E-12 | 44 | 298 | pigment metabolic process |
| GO:0009767 | 9.79E-14 | 4.18E-12 | 22 | 76 | photosynthetic electron transport chain |
| GO:0008266 | 1.85E-13 | 7.71E-12 | 17 | 43 | poly(U) RNA binding |
| GO:0006783 | 1.85E-13 | 7.71E-12 | 17 | 43 | heme biosynthetic process |
| GO:0044283 | 2.47E-13 | 1.01E-11 | 174 | 2507 | small molecule biosynthetic process |
| GO:0009735 | 2.78E-13 | 1.13E-11 | 73 | 723 | response to cytokinin stimulus |
| GO:0010207 | 7.68E-13 | 3.07E-11 | 16 | 40 | photosystem II assembly |
| GO:0042168 | 1.04E-12 | 4.11E-11 | 17 | 47 | heme metabolic process |
| GO:0055114 | 1.26E-12 | 4.90E-11 | 192 | 2909 | oxidation reduction |
| GO:0005840 | 1.28E-12 | 4.93E-11 | 95 | 1106 | ribosome |
| GO:0000311 | 7.35E-12 | 2.73E-10 | 10 | 14 | plastid large ribosomal subunit |
| GO:0009658 | 1.97E-11 | 7.23E-10 | 42 | 327 | chloroplast organization |
| GO:0010319 | 2.56E-11 | 9.31E-10 | 25 | 127 | stromule |
| GO:0046394 | 3.18E-11 | 1.13E-09 | 100 | 1260 | carboxylic acid biosynthetic process |
| GO:0016053 | 3.18E-11 | 1.13E-09 | 100 | 1260 | organic acid biosynthetic process |
| GO:0008187 | 3.65E-11 | 1.28E-09 | 17 | 57 | poly-pyrimidine tract binding |
| GO:0030076 | 1.67E-10 | 5.81E-09 | 25 | 138 | light-harvesting complex |
| GO:0006631 | 2.16E-10 | 7.42E-09 | 71 | 800 | fatty acid metabolic process |
| GO:0042549 | 3.63E-10 | 1.23E-08 | 9 | 14 | photosystem II stabilization |
| GO:0006412 | 4.00E-10 | 1.34E-08 | 107 | 1448 | translation |

| | | | | | |
|---|---|---|---|---|---|
| GO:0005198 | 4.69E-10 | 1.56E-08 | 95 | 1233 | structural molecule activity |
| GO:0003735 | 5.13E-10 | 1.69E-08 | 77 | 918 | structural constituent of ribosome |
| GO:0051920 | 5.66E-10 | 1.84E-08 | 10 | 19 | peroxiredoxin activity |
| GO:0022900 | 6.26E-10 | 2.02E-08 | 28 | 181 | electron transport chain |
| GO:0031225 | 7.63E-10 | 2.44E-08 | 52 | 516 | anchored to membrane |
| GO:0046906 | 1.26E-09 | 3.98E-08 | 51 | 508 | tetrapyrrole binding |
| GO:0044425 | 2.22E-09 | 6.93E-08 | 620 | 12867 | membrane part |
| GO:0006633 | 3.17E-09 | 9.74E-08 | 51 | 522 | fatty acid biosynthetic process |
| GO:0006949 | 3.17E-09 | 9.74E-08 | 13 | 41 | syncytium formation |
| GO:0010007 | 3.63E-09 | 1.09E-07 | 6 | 6 | magnesium chelatase complex |
| GO:0003727 | 7.70E-09 | 2.30E-07 | 21 | 119 | single-stranded RNA binding |
| GO:0032787 | 2.09E-08 | 6.13E-07 | 92 | 1275 | monocarboxylic acid metabolic process |
| GO:0044106 | 2.56E-08 | 7.41E-07 | 84 | 1132 | cellular amine metabolic process |
| GO:0042548 | 3.09E-08 | 8.89E-07 | 14 | 57 | regulation of photosynthesis, light reaction |
| GO:0018198 | 4.08E-08 | 1.16E-06 | 20 | 119 | peptidyl-cysteine modification |
| GO:0006519 | 4.53E-08 | 1.28E-06 | 126 | 1957 | cellular amino acid and derivative metabolic process |
| GO:0005507 | 8.55E-08 | 2.39E-06 | 54 | 628 | copper ion binding |
| GO:0005975 | 8.95E-08 | 2.48E-06 | 161 | 2697 | carbohydrate metabolic process |
| GO:0004853 | 9.26E-08 | 2.52E-06 | 5 | 5 | uroporphyrinogen decarboxylase activity |
| GO:0047100 | 9.26E-08 | 2.52E-06 | 5 | 5 | glyceraldehyde-3-phosphate dehydrogenase (NADP+) (phosphorylating) activity |
| GO:0043467 | 9.73E-08 | 2.63E-06 | 14 | 62 | regulation of generation of precursor metabolites and energy |
| GO:0046483 | 1.04E-07 | 2.80E-06 | 75 | 1003 | heterocycle metabolic process |
| GO:0016491 | 1.11E-07 | 2.96E-06 | 189 | 3296 | oxidoreductase activity |
| GO:0004312 | 1.49E-07 | 3.92E-06 | 14 | 64 | fatty acid synthase activity |
| GO:0000313 | 2.24E-07 | 5.84E-06 | 14 | 66 | organellar ribosome |
| GO:0031224 | 2.52E-07 | 6.52E-06 | 473 | 9769 | intrinsic to membrane |
| GO:0016851 | 2.75E-07 | 6.95E-06 | 6 | 9 | magnesium chelatase activity |
| GO:0051002 | 2.75E-07 | 6.95E-06 | 6 | 9 | ligase activity, forming nitrogen-metal bonds |
| GO:0051003 | 2.75E-07 | 6.95E-06 | 6 | 9 | ligase activity, forming nitrogen-metal bonds, forming coordination complexes |
| GO:0010109 | 4.59E-07 | 1.15E-05 | 15 | 80 | regulation of photosynthesis |
| GO:0009773 | 4.69E-07 | 1.17E-05 | 10 | 34 | photosynthetic electron transport in photosystem I |
| GO:0006007 | 4.95E-07 | 1.22E-05 | 27 | 231 | glucose catabolic process |

| | | | | | |
|---|---|---|---|---|---|
| GO:0009308 | 5.09E-07 | 1.25E-05 | 91 | 1350 | amine metabolic process |
| GO:0009279 | 5.38E-07 | 1.29E-05 | 5 | 6 | cell outer membrane |
| GO:0009782 | 5.38E-07 | 1.29E-05 | 5 | 6 | photosystem I antenna complex |
| GO:0010242 | 5.38E-07 | 1.29E-05 | 5 | 6 | oxygen evolving activity |
| GO:0010114 | 7.90E-07 | 1.88E-05 | 20 | 142 | response to red light |
| GO:0030312 | 1.03E-06 | 2.44E-05 | 136 | 2283 | external encapsulating structure |
| GO:0019320 | 1.05E-06 | 2.45E-05 | 27 | 240 | hexose catabolic process |
| GO:0046365 | 1.05E-06 | 2.45E-05 | 27 | 240 | monosaccharide catabolic process |
| GO:0000315 | 1.12E-06 | 2.58E-05 | 10 | 37 | organellar large ribosomal subunit |
| GO:0016853 | 1.22E-06 | 2.76E-05 | 49 | 596 | isomerase activity |
| GO:0005996 | 1.25E-06 | 2.82E-05 | 45 | 528 | monosaccharide metabolic process |
| GO:0006629 | 1.33E-06 | 2.97E-05 | 145 | 2482 | lipid metabolic process |
| GO:0009828 | 1.55E-06 | 3.45E-05 | 16 | 99 | plant-type cell wall loosening |
| GO:0009309 | 1.60E-06 | 3.54E-05 | 46 | 550 | amine biosynthetic process |
| GO:0000312 | 2.37E-06 | 5.08E-05 | 4 | 4 | plastid small ribosomal subunit |
| GO:0016712 | 2.37E-06 | 5.08E-05 | 4 | 4 | oxidoreductase activity, acting on paired donors, with incorporation or reduction of molecular oxygen, reduced flavin or flavoprotein as one donor, and incorporation of one atom of oxygen |
| GO:0070330 | 2.37E-06 | 5.08E-05 | 4 | 4 | aromatase activity |
| GO:0006096 | 2.38E-06 | 5.08E-05 | 22 | 179 | glycolysis |
| GO:0048564 | 2.73E-06 | 5.79E-05 | 6 | 12 | photosystem I assembly |
| GO:0009533 | 3.08E-06 | 6.49E-05 | 7 | 18 | chloroplast stromal thylakoid |
| GO:0032991 | 3.20E-06 | 6.70E-05 | 292 | 5772 | macromolecular complex |
| GO:0009295 | 3.84E-06 | 7.98E-05 | 15 | 94 | nucleoid |
| GO:0009528 | 4.04E-06 | 8.35E-05 | 21 | 171 | plastid inner membrane |
| GO:0046164 | 4.86E-06 | 9.97E-05 | 27 | 260 | alcohol catabolic process |
| GO:0019318 | 5.79E-06 | 1.18E-04 | 36 | 406 | hexose metabolic process |
| GO:0043094 | 5.93E-06 | 1.20E-04 | 24 | 218 | cellular metabolic compound salvage |
| GO:0046658 | 6.08E-06 | 1.22E-04 | 29 | 294 | anchored to plasma membrane |
| GO:0006732 | 6.41E-06 | 1.28E-04 | 51 | 669 | coenzyme metabolic process |
| GO:0005618 | 8.18E-06 | 1.62E-04 | 131 | 2272 | cell wall |
| GO:0016984 | 8.30E-06 | 1.62E-04 | 6 | 14 | ribulose-bisphosphate carboxylase activity |
| GO:0009573 | 8.30E-06 | 1.62E-04 | 6 | 14 | chloroplast ribulose bisphosphate carboxylase complex |
| GO:0048492 | 8.30E-06 | 1.62E-04 | 6 | 14 | ribulose bisphosphate carboxylase complex |
| GO:0044237 | 9.35E-06 | 1.81E-04 | 875 | 19921 | cellular metabolic process |

| | | | | | |
|---|---|---|---|---|---|
| GO:0004550 | 1.02E-05 | 1.96E-04 | 7 | 21 | nucleoside diphosphate kinase activity |
| GO:0080065 | 1.15E-05 | 2.19E-04 | 4 | 5 | 4-alpha-methyl-delta7-sterol-4alpha-methyl oxidase activity |
| GO:0003755 | 1.42E-05 | 2.71E-04 | 16 | 117 | peptidyl-prolyl cis-trans isomerase activity |
| GO:0006006 | 1.55E-05 | 2.93E-04 | 27 | 277 | glucose metabolic process |
| GO:0047259 | 2.07E-05 | 3.89E-04 | 6 | 16 | glucomannan 4-beta-mannosyltransferase activity |
| GO:0006066 | 2.10E-05 | 3.93E-04 | 66 | 984 | alcohol metabolic process |
| GO:0009706 | 2.41E-05 | 4.47E-04 | 19 | 163 | chloroplast inner membrane |
| GO:0019205 | 2.60E-05 | 4.80E-04 | 14 | 97 | nucleobase, nucleoside, nucleotide kinase activity |
| GO:0030529 | 2.76E-05 | 5.07E-04 | 102 | 1720 | ribonucleoprotein complex |
| GO:0010205 | 3.20E-05 | 5.81E-04 | 8 | 33 | photoinhibition |
| GO:0043155 | 3.20E-05 | 5.81E-04 | 8 | 33 | negative regulation of photosynthesis, light reaction |
| GO:0016859 | 3.28E-05 | 5.91E-04 | 16 | 125 | cis-trans isomerase activity |
| GO:0005509 | 3.29E-05 | 5.91E-04 | 38 | 475 | calcium ion binding |
| GO:0009783 | 3.33E-05 | 5.94E-04 | 4 | 6 | photosystem II antenna complex |
| GO:0018065 | 3.51E-05 | 6.20E-04 | 5 | 11 | protein-cofactor linkage |
| GO:0030313 | 3.51E-05 | 6.20E-04 | 5 | 11 | cell envelope |
| GO:0009637 | 3.67E-05 | 6.44E-04 | 19 | 168 | response to blue light |
| GO:0004650 | 4.13E-05 | 7.21E-04 | 15 | 114 | polygalacturonase activity |
| GO:0051287 | 4.92E-05 | 8.54E-04 | 17 | 143 | NAD or NADH binding |
| GO:0042547 | 5.08E-05 | 8.77E-04 | 12 | 78 | cell wall modification involved in multidimensional cell growth |
| GO:0016418 | 5.82E-05 | 9.94E-04 | 5 | 12 | S-acetyltransferase activity |
| GO:0004742 | 5.82E-05 | 9.94E-04 | 5 | 12 | dihydrolipoyllysine-residue acetyltransferase activity |
| GO:0016052 | 5.89E-05 | 1.00E-03 | 55 | 804 | carbohydrate catabolic process |
| GO:0016869 | 6.04E-05 | 1.01E-03 | 3 | 3 | intramolecular transferase activity, transferring amino groups |
| GO:0042286 | 6.04E-05 | 1.01E-03 | 3 | 3 | glutamate-1-semialdehyde 2,1-aminomutase activity |
| GO:0004462 | 6.28E-05 | 1.05E-03 | 8 | 36 | lactoylglutathione lyase activity |
| GO:0045298 | 6.31E-05 | 1.05E-03 | 7 | 27 | tubulin complex |
| GO:0009628 | 7.25E-05 | 1.20E-03 | 246 | 4956 | response to abiotic stimulus |
| GO:0016209 | 7.99E-05 | 1.32E-03 | 31 | 372 | antioxidant activity |
| GO:0006725 | 8.25E-05 | 1.35E-03 | 78 | 1272 | cellular aromatic compound metabolic process |
| GO:0035304 | 9.15E-05 | 1.48E-03 | 5 | 13 | regulation of protein amino acid dephosphorylation |
| GO:0019676 | 9.15E-05 | 1.48E-03 | 5 | 13 | ammonia assimilation cycle |
| GO:0005527 | 1.08E-04 | 1.73E-03 | 9 | 49 | macrolide binding |
| GO:0005528 | 1.08E-04 | 1.73E-03 | 9 | 49 | FK506 binding |
| GO:0010206 | 1.18E-04 | 1.89E-03 | 6 | 21 | photosystem II repair |
| GO:0009058 | 1.21E-04 | 1.93E-03 | 474 | 10382 | biosynthetic process |

| | | | | | | |
|---|---|---|---|---|---|---|
| | GO:1901700 | 1E-30 | 1E-30 | 442 | 3988 | response to oxygen-containing compound |
| | GO:0010243 | 1E-30 | 1E-30 | 121 | 505 | response to organonitrogen compound |
| | GO:1901698 | 1E-30 | 1E-30 | 123 | 643 | response to nitrogen compound |
| | GO:0001101 | 8.5E-27 | 8.5E-27 | 314 | 3008 | response to acid chemical |
| | GO:0071310 | 4.5E-23 | 4.5E-23 | 235 | 2136 | cellular response to organic substance |
| | GO:0009725 | 5.3E-23 | 5.3E-23 | 391 | 4237 | response to hormone |
| | GO:0035556 | 2.5E-19 | 2.5E-19 | 130 | 988 | intracellular signal transduction |
| | GO:0009723 | 1.4E-17 | 1.4E-17 | 109 | 798 | response to ethylene |
| | GO:0071395 | 6.3E-17 | 6.3E-17 | 52 | 243 | cellular response to jasmonic acid stimulus |
| | GO:0009867 | 2E-16 | 2E-16 | 51 | 241 | jasmonic acid mediated signaling pathway |
| | GO:0071229 | 1.2E-15 | 1.2E-15 | 126 | 1049 | cellular response to acid chemical |
| | GO:0014070 | 1.6E-15 | 1.6E-15 | 108 | 842 | response to organic cyclic compound |
| | GO:0009753 | 1.3E-14 | 1.3E-14 | 84 | 597 | response to jasmonic acid |
| | GO:0009751 | 1.2E-13 | 1.2E-13 | 70 | 469 | response to salicylic acid |
| | GO:0009863 | 4.4E-13 | 4.4E-13 | 31 | 119 | salicylic acid mediated signaling pathway |
| | GO:1903506 | 2.6E-11 | 2.6E-11 | 430 | 5639 | regulation of nucleic acid-templated transcription |
| | GO:0009889 | 3.7E-10 | 3.7E-10 | 456 | 6161 | regulation of biosynthetic process |
| Blue | GO:0008150 | 9.3E-10 | 9.3E-10 | 2733 | 46836 | biological process |
| | GO:0097659 | 2.8E-09 | 2.8E-09 | 434 | 5907 | nucleic acid-templated transcription |
| | GO:0051171 | 0.000000034 | 0.000000034 | 471 | 6623 | regulation of nitrogen compound metabolic process |
| | GO:0016126 | 0.00011 | 0.00011 | 15 | 88 | sterol biosynthetic process |
| | GO:0009095 | 0.00975 | 0.00975 | 5 | 24 | aromatic amino acid family biosynthetic process, prephenate pathway |
| | GO:0051172 | 0.01267 | 0.01267 | 64 | 853 | negative regulation of nitrogen compound metabolic process |
| | GO:0018401 | 0.00062 | 0.00062 | 6 | 20 | peptidyl-proline hydroxylation to 4-hydroxy-L-proline |
| | GO:0019471 | 0.00062 | 0.00062 | 6 | 20 | 4-hydroxyproline metabolic process |
| | GO:0019511 | 0.00062 | 0.00062 | 6 | 20 | peptidyl-proline hydroxylation |
| | GO:0043565 | 3.4E-17 | 3.4E-17 | 130 | 1005 | sequence-specific DNA binding |
| | GO:0140110 | 6.20E-17 | 6.20E-17 | 366 | 4055 | transcription regulator activity |
| | GO:0016301 | 7.40E-17 | 7.40E-17 | 307 | 3250 | kinase activity |
| | GO:0001067 | 3.4E-12 | 3.4E-12 | 70 | 482 | regulatory region nucleic acid binding |
| | GO:0044212 | 3.4E-12 | 3.4E-12 | 70 | 482 | transcription regulatory region DNA binding |
| | GO:0016772 | 2.3E-10 | 2.3E-10 | 317 | 3820 | transferase activity, transferring phosphorus-containing groups |
| | GO:0003674 | 0.00000034 | 0.00000034 | 2844 | 47020 | molecular function |
| | GO:0140096 | 0.0000078 | 0.0000078 | 377 | 5185 | catalytic activity, acting on a protein |

| | | | | | |
|---|---|---|---|---|---|
| GO:0008481 | 0.000012 | 0.000012 | 4 | 4 | sphinganine kinase activity |
| GO:0004656 | 0.00136 | 0.00136 | 6 | 22 | procollagen-proline 4-dioxygenase activity |
| GO:0019798 | 0.00136 | 0.00136 | 6 | 22 | procollagen-proline dioxygenase activity |
| GO:0031543 | 0.00136 | 0.00136 | 6 | 22 | peptidyl-proline dioxygenase activity |
| GO:0031545 | 0.00136 | 0.00136 | 6 | 22 | peptidyl-proline 4-dioxygenase activity |
| GO:1990714 | 0.00966 | 0.00966 | 4 | 15 | hydroxyproline O-galactosyltransferase activity |
| GO:0010200 | 2.12E-52 | 6.89E-49 | 117 | 371 | response to chitin |
| GO:0009743 | 1.55E-30 | 2.52E-27 | 135 | 756 | response to carbohydrate stimulus |
| GO:0010033 | 2.33E-29 | 2.52E-26 | 474 | 4794 | response to organic substance |
| GO:0042221 | 2.47E-28 | 2.01E-25 | 645 | 7198 | response to chemical stimulus |
| GO:0006952 | 6.03E-22 | 3.92E-19 | 328 | 3232 | defense response |
| GO:0051707 | 3.04E-20 | 1.65E-17 | 265 | 2502 | response to other organism |
| GO:0009607 | 9.48E-20 | 4.41E-17 | 270 | 2587 | response to biotic stimulus |
| GO:0005886 | 1.20E-19 | 4.87E-17 | 834 | 10612 | plasma membrane |
| GO:0009620 | 2.22E-19 | 8.02E-17 | 120 | 829 | response to fungus |
| GO:0006950 | 2.47E-19 | 8.02E-17 | 662 | 8066 | response to stress |
| GO:0016020 | 4.79E-19 | 1.42E-16 | 1407 | 19707 | membrane |
| GO:0030528 | 1.45E-17 | 3.94E-15 | 374 | 4080 | transcription regulator activity |
| GO:0050832 | 6.47E-17 | 1.62E-14 | 95 | 627 | defense response to fungus |
| GO:0050896 | 9.73E-17 | 2.11E-14 | 981 | 13159 | response to stimulus |
| GO:0031224 | 1.36E-16 | 2.76E-14 | 760 | 9769 | intrinsic to membrane |
| GO:0051704 | 1.68E-16 | 3.22E-14 | 291 | 3019 | multi-organism process |
| GO:0003700 | 2.68E-16 | 4.85E-14 | 353 | 3869 | transcription factor activity |
| GO:0031347 | 5.53E-16 | 8.65E-14 | 78 | 477 | regulation of defense response |
| GO:0009617 | 5.58E-16 | 8.65E-14 | 134 | 1075 | response to bacterium |
| GO:0080134 | 6.12E-16 | 9.04E-14 | 86 | 557 | regulation of response to stress |
| GO:0009873 | 1.14E-15 | 1.62E-13 | 75 | 454 | ethylene mediated signaling pathway |
| GO:0005509 | 1.38E-15 | 1.87E-13 | 77 | 475 | calcium ion binding |
| GO:0000160 | 2.48E-15 | 3.23E-13 | 85 | 560 | two-component signal transduction system (phosphorelay) |
| GO:0023033 | 2.93E-15 | 3.67E-13 | 277 | 2900 | signaling pathway |
| GO:0023052 | 8.25E-15 | 9.94E-13 | 324 | 3563 | signaling |

| | | | | | |
|---|---|---|---|---|---|
| GO:0023060 | 1.08E-14 | 1.26E-12 | 185 | 1735 | signal transmission |
| GO:0023046 | 1.27E-14 | 1.43E-12 | 185 | 1738 | signaling process |
| GO:0071369 | 1.48E-14 | 1.61E-12 | 75 | 476 | cellular response to ethylene stimulus |
| GO:0010941 | 3.49E-14 | 3.44E-12 | 52 | 267 | regulation of cell death |
| GO:0048583 | 1.62E-13 | 1.55E-11 | 119 | 981 | regulation of response to stimulus |
| GO:0006464 | 1.77E-13 | 1.65E-11 | 394 | 4635 | protein modification process |
| GO:0042742 | 2.32E-13 | 2.09E-11 | 102 | 792 | defense response to bacterium |
| GO:0071446 | 2.42E-13 | 2.13E-11 | 33 | 125 | cellular response to salicylic acid stimulus |
| GO:0071495 | 2.66E-13 | 2.27E-11 | 181 | 1746 | cellular response to endogenous stimulus |
| GO:0016021 | 3.54E-13 | 2.88E-11 | 709 | 9333 | integral to membrane |
| GO:0060548 | 1.19E-12 | 9.25E-11 | 23 | 65 | negative regulation of cell death |
| GO:0009719 | 2.08E-12 | 1.58E-10 | 341 | 3962 | response to endogenous stimulus |
| GO:0043687 | 3.25E-12 | 2.40E-10 | 351 | 4118 | post-translational protein modification |
| GO:0070887 | 4.14E-12 | 2.97E-10 | 235 | 2516 | cellular response to chemical stimulus |
| GO:0002237 | 4.20E-12 | 2.97E-10 | 29 | 108 | response to molecule of bacterial origin |
| GO:0044425 | 1.97E-11 | 1.31E-09 | 923 | 12867 | membrane part |
| GO:0006796 | 9.08E-11 | 5.79E-09 | 240 | 2667 | phosphate metabolic process |
| GO:0009816 | 9.79E-11 | 6.12E-09 | 28 | 114 | defense response to bacterium, incompatible interaction |
| GO:0006793 | 1.01E-10 | 6.19E-09 | 240 | 2670 | phosphorus metabolic process |
| GO:0043412 | 1.69E-10 | 1.02E-08 | 400 | 4967 | macromolecule modification |
| GO:0009611 | 2.23E-10 | 1.32E-08 | 84 | 679 | response to wounding |
| GO:0009409 | 1.10E-09 | 6.30E-08 | 112 | 1039 | response to cold |
| GO:0006350 | 4.10E-09 | 2.26E-07 | 325 | 3997 | transcription |
| GO:0006351 | 4.10E-09 | 2.26E-07 | 325 | 3997 | transcription, DNA-dependent |
| GO:0050794 | 4.70E-09 | 2.47E-07 | 648 | 8873 | regulation of cellular process |
| GO:0050776 | 4.86E-09 | 2.47E-07 | 41 | 252 | regulation of immune response |
| GO:0002682 | 4.86E-09 | 2.47E-07 | 41 | 252 | regulation of immune system process |
| GO:0032774 | 5.32E-09 | 2.66E-07 | 325 | 4007 | RNA biosynthetic process |
| GO:0005794 | 1.20E-08 | 5.88E-07 | 243 | 2859 | Golgi apparatus |
| GO:0032870 | 1.21E-08 | 5.88E-07 | 153 | 1619 | cellular response to hormone stimulus |

| | | | | | |
|---|---|---|---|---|---|
| GO:0018108 | 2.19E-08 | 1.03E-06 | 8 | 11 | peptidyl-tyrosine phosphorylation |
| GO:0018212 | 2.19E-08 | 1.03E-06 | 8 | 11 | peptidyl-tyrosine modification |
| GO:0045087 | 2.26E-08 | 1.05E-06 | 102 | 971 | innate immune response |
| GO:0006468 | 2.53E-08 | 1.16E-06 | 166 | 1813 | protein amino acid phosphorylation |
| GO:0009755 | 2.64E-08 | 1.19E-06 | 142 | 1492 | hormone-mediated signaling pathway |
| GO:0005789 | 3.01E-08 | 1.34E-06 | 95 | 889 | endoplasmic reticulum membrane |
| GO:0002376 | 3.22E-08 | 1.42E-06 | 112 | 1105 | immune system process |
| GO:0042175 | 6.31E-08 | 2.74E-06 | 95 | 903 | nuclear membrane-endoplasmic reticulum network |
| GO:0051245 | 8.50E-08 | 3.59E-06 | 7 | 9 | negative regulation of cellular defense response |
| GO:0015690 | 8.50E-08 | 3.59E-06 | 7 | 9 | aluminum ion transport |
| GO:0043069 | 1.03E-07 | 4.29E-06 | 14 | 43 | negative regulation of programmed cell death |
| GO:0006955 | 1.39E-07 | 5.73E-06 | 107 | 1072 | immune response |
| GO:0010185 | 1.53E-07 | 6.16E-06 | 8 | 13 | regulation of cellular defense response |
| GO:0045088 | 1.53E-07 | 6.16E-06 | 34 | 213 | regulation of innate immune response |
| GO:0016310 | 1.73E-07 | 6.87E-06 | 196 | 2285 | phosphorylation |
| GO:0045449 | 2.12E-07 | 8.31E-06 | 420 | 5584 | regulation of transcription |
| GO:0006470 | 2.51E-07 | 9.73E-06 | 38 | 258 | protein amino acid dephosphorylation |
| GO:0006665 | 2.63E-07 | 1.01E-05 | 20 | 90 | sphingolipid metabolic process |
| GO:0050789 | 3.03E-07 | 1.14E-05 | 703 | 9988 | regulation of biological process |
| GO:0005783 | 3.08E-07 | 1.15E-05 | 171 | 1954 | endoplasmic reticulum |
| GO:0016567 | 3.78E-07 | 1.40E-05 | 132 | 1426 | protein ubiquitination |
| GO:0010120 | 4.44E-07 | 1.61E-05 | 12 | 35 | camalexin biosynthetic process |
| GO:0052317 | 4.44E-07 | 1.61E-05 | 12 | 35 | camalexin metabolic process |
| GO:0031349 | 6.19E-07 | 2.21E-05 | 29 | 176 | positive regulation of defense response |
| GO:0006355 | 6.68E-07 | 2.36E-05 | 413 | 5538 | regulation of transcription, DNA-dependent |
| GO:0002831 | 7.73E-07 | 2.70E-05 | 13 | 43 | regulation of response to biotic stimulus |
| GO:0043067 | 8.68E-07 | 2.90E-05 | 34 | 229 | regulation of programmed cell death |
| GO:0042431 | 8.82E-07 | 2.90E-05 | 12 | 37 | indole metabolic process |
| GO:0009700 | 8.82E-07 | 2.90E-05 | 12 | 37 | indole phytoalexin biosynthetic process |
| GO:0052314 | 8.82E-07 | 2.90E-05 | 12 | 37 | phytoalexin metabolic process |

| | | | | | |
|---|---|---|---|---|---|
| GO:0052315 | 8.82E-07 | 2.90E-05 | 12 | 37 | phytoalexin biosynthetic process |
| GO:0046217 | 8.82E-07 | 2.90E-05 | 12 | 37 | indole phytoalexin metabolic process |
| GO:0032446 | 9.25E-07 | 3.01E-05 | 135 | 1491 | protein modification by small protein conjugation |
| GO:0044432 | 1.19E-06 | 3.80E-05 | 95 | 964 | endoplasmic reticulum part |
| GO:0071212 | 1.19E-06 | 3.80E-05 | 95 | 964 | subsynaptic reticulum |
| GO:0010371 | 1.59E-06 | 5.03E-05 | 7 | 12 | regulation of gibberellin biosynthetic process |
| GO:0009266 | 2.01E-06 | 6.30E-05 | 136 | 1527 | response to temperature stimulus |
| GO:0051252 | 2.22E-06 | 6.87E-05 | 413 | 5604 | regulation of RNA metabolic process |
| GO:0051716 | 2.31E-06 | 7.10E-05 | 332 | 4378 | cellular response to stimulus |
| GO:0023034 | 2.46E-06 | 7.47E-05 | 57 | 501 | intracellular signaling pathway |
| GO:0004672 | 2.62E-06 | 7.90E-05 | 172 | 2038 | protein kinase activity |
| GO:0016773 | 2.89E-06 | 8.60E-05 | 194 | 2355 | phosphotransferase activity, alcohol group as acceptor |
| GO:0030148 | 2.91E-06 | 8.60E-05 | 14 | 55 | sphingolipid biosynthetic process |
| GO:0006979 | 2.95E-06 | 8.63E-05 | 81 | 803 | response to oxidative stress |
| GO:0009415 | 3.77E-06 | 1.09E-04 | 89 | 912 | response to water |
| GO:0048585 | 5.25E-06 | 1.51E-04 | 48 | 406 | negative regulation of response to stimulus |
| GO:0009814 | 6.02E-06 | 1.72E-04 | 61 | 565 | defense response, incompatible interaction |
| GO:0005768 | 6.53E-06 | 1.85E-04 | 90 | 938 | endosome |
| GO:0003677 | 7.42E-06 | 2.07E-04 | 432 | 5967 | DNA binding |
| GO:0065007 | 7.46E-06 | 2.07E-04 | 798 | 11737 | biological regulation |
| GO:0003830 | 7.54E-06 | 2.08E-04 | 6 | 10 | beta-1,4-mannosylglycoprotein 4-beta-N-acetylglucosaminyltransferase activity |
| GO:0070647 | 8.22E-06 | 2.25E-04 | 137 | 1584 | protein modification by small protein conjugation or removal |
| GO:0019219 | 9.81E-06 | 2.64E-04 | 422 | 5830 | regulation of nucleobase, nucleoside, nucleotide and nucleic acid metabolic process |
| GO:0005484 | 1.09E-05 | 2.91E-04 | 17 | 86 | SNAP receptor activity |
| GO:0009737 | 1.13E-05 | 2.99E-04 | 116 | 1303 | response to abscisic acid stimulus |
| GO:0009862 | 1.15E-05 | 3.02E-04 | 11 | 39 | systemic acquired resistance, salicylic acid mediated signaling pathway |
| GO:0031326 | 1.24E-05 | 3.16E-04 | 434 | 6030 | regulation of cellular biosynthetic process |
| GO:0051091 | 1.25E-05 | 3.16E-04 | 4 | 4 | positive regulation of transcription factor activity |

| | | | | | |
|---|---|---|---|---|---|
| GO:0090047 | 1.25E-05 | 3.16E-04 | 4 | 4 | positive regulation of transcription regulator activity |
| GO:0031348 | 1.30E-05 | 3.26E-04 | 25 | 163 | negative regulation of defense response |
| GO:0019787 | 1.50E-05 | 3.68E-04 | 75 | 761 | small conjugating protein ligase activity |
| GO:0016564 | 1.50E-05 | 3.68E-04 | 11 | 40 | transcription repressor activity |
| GO:0003714 | 1.50E-05 | 3.68E-04 | 11 | 40 | transcription corepressor activity |
| GO:0009414 | 1.56E-05 | 3.78E-04 | 85 | 893 | response to water deprivation |
| GO:0033612 | 1.59E-05 | 3.80E-04 | 14 | 63 | receptor serine/threonine kinase binding |
| GO:0002218 | 1.60E-05 | 3.80E-04 | 22 | 135 | activation of innate immune response |
| GO:0002253 | 1.60E-05 | 3.80E-04 | 22 | 135 | activation of immune response |
| GO:0004721 | 1.72E-05 | 4.05E-04 | 52 | 473 | phosphoprotein phosphatase activity |
| GO:0004842 | 1.96E-05 | 4.58E-04 | 72 | 728 | ubiquitin-protein ligase activity |
| GO:0031323 | 2.02E-05 | 4.69E-04 | 479 | 6761 | regulation of cellular metabolic process |
| GO:0010565 | 2.03E-05 | 4.69E-04 | 24 | 157 | regulation of cellular ketone metabolic process |
| GO:0010556 | 2.27E-05 | 5.20E-04 | 422 | 5883 | regulation of macromolecule biosynthetic process |
| GO:0004713 | 2.34E-05 | 5.33E-04 | 8 | 22 | protein tyrosine kinase activity |
| GO:0042578 | 2.99E-05 | 6.66E-04 | 81 | 856 | phosphoric ester hydrolase activity |
| GO:0060860 | 2.99E-05 | 6.66E-04 | 6 | 12 | regulation of floral organ abscission |
| GO:0060862 | 2.99E-05 | 6.66E-04 | 6 | 12 | negative regulation of floral organ abscission |
| GO:0016050 | 3.09E-05 | 6.83E-04 | 18 | 102 | vesicle organization |
| GO:0060089 | 3.57E-05 | 7.80E-04 | 75 | 781 | molecular transducer activity |
| GO:0004871 | 3.57E-05 | 7.80E-04 | 75 | 781 | signal transducer activity |
| GO:0005802 | 3.64E-05 | 7.90E-04 | 66 | 664 | trans-Golgi network |
| GO:0048584 | 4.10E-05 | 8.84E-04 | 39 | 331 | positive regulation of response to stimulus |
| GO:0005102 | 4.22E-05 | 9.03E-04 | 17 | 95 | receptor binding |
| GO:0010337 | 4.33E-05 | 9.20E-04 | 10 | 37 | regulation of salicylic acid metabolic process |
| GO:0046520 | 4.66E-05 | 9.84E-04 | 7 | 18 | sphingoid biosynthetic process |
| GO:0004674 | 4.73E-05 | 9.86E-04 | 140 | 1686 | protein serine/threonine kinase activity |
| GO:0016311 | 4.77E-05 | 9.89E-04 | 41 | 357 | dephosphorylation |
| GO:0004722 | 5.39E-05 | 1.11E-03 | 40 | 347 | protein serine/threonine phosphatase activity |
| GO:0000186 | 5.96E-05 | 1.21E-03 | 4 | 5 | activation of MAPKK activity |

| | | | | | |
|---|---|---|---|---|---|
| GO:0043388 | 5.96E-05 | 1.21E-03 | 4 | 5 | positive regulation of DNA binding |
| GO:0008514 | 6.56E-05 | 1.32E-03 | 14 | 71 | organic anion transmembrane transporter activity |
| GO:0010008 | 6.98E-05 | 1.39E-03 | 31 | 246 | endosome membrane |
| GO:0044440 | 6.98E-05 | 1.39E-03 | 31 | 246 | endosomal part |
| GO:0050691 | 7.00E-05 | 1.39E-03 | 7 | 19 | regulation of defense response to virus by host |
| GO:0016740 | 7.59E-05 | 1.48E-03 | 546 | 7911 | transferase activity |
| GO:0034051 | 7.66E-05 | 1.48E-03 | 5 | 9 | negative regulation of plant-type hypersensitive response |
| GO:0010483 | 7.66E-05 | 1.48E-03 | 5 | 9 | pollen tube reception |
| GO:0051703 | 7.66E-05 | 1.48E-03 | 5 | 9 | intraspecies interaction between organisms |
| GO:0051592 | 7.71E-05 | 1.48E-03 | 11 | 47 | response to calcium ion |
| GO:0030246 | 8.25E-05 | 1.58E-03 | 52 | 502 | carbohydrate binding |
| GO:0010942 | 9.25E-05 | 1.76E-03 | 12 | 56 | positive regulation of cell death |
| GO:0019747 | 9.46E-05 | 1.78E-03 | 11 | 48 | regulation of isoprenoid metabolic process |
| GO:0005217 | 9.46E-05 | 1.78E-03 | 11 | 48 | intracellular ligand-gated ion channel activity |
| GO:0070417 | 1.06E-04 | 1.99E-03 | 17 | 102 | cellular response to cold |
| GO:0006643 | 1.21E-04 | 2.25E-03 | 25 | 186 | membrane lipid metabolic process |
| GO:0009804 | 1.24E-04 | 2.29E-03 | 8 | 27 | coumarin metabolic process |
| GO:0009805 | 1.24E-04 | 2.29E-03 | 8 | 27 | coumarin biosynthetic process |
| GO:0004708 | 1.39E-04 | 2.53E-03 | 6 | 15 | MAP kinase kinase activity |
| GO:0010363 | 1.39E-04 | 2.53E-03 | 10 | 42 | regulation of plant-type hypersensitive response |
| GO:0042546 | 1.46E-04 | 2.60E-03 | 45 | 425 | cell wall biogenesis |
| GO:0046834 | 1.46E-04 | 2.60E-03 | 5 | 10 | lipid phosphorylation |
| GO:0080142 | 1.46E-04 | 2.60E-03 | 5 | 10 | regulation of salicylic acid biosynthetic process |
| GO:0010411 | 1.48E-04 | 2.63E-03 | 16 | 95 | xyloglucan metabolic process |
| GO:0009628 | 1.53E-04 | 2.71E-03 | 354 | 4956 | response to abiotic stimulus |
| GO:0016762 | 1.64E-04 | 2.88E-03 | 14 | 77 | xyloglucan:xyloglucosyl transferase activity |
| GO:0010382 | 1.69E-04 | 2.94E-03 | 29 | 235 | cellular cell wall macromolecule metabolic process |
| GO:0035264 | 1.70E-04 | 2.94E-03 | 4 | 6 | multicellular organism growth |
| GO:0045140 | 1.70E-04 | 2.94E-03 | 4 | 6 | inositol phosphoceramide synthase activity |
| GO:0070882 | 1.71E-04 | 2.94E-03 | 51 | 504 | cellular cell wall organization or biogenesis |

| | | | | | |
|---|---|---|---|---|---|
| GO:0048523 | 1.75E-04 | 3.00E-03 | 103 | 1208 | negative regulation of cellular process |
| GO:0050778 | 1.80E-04 | 3.04E-03 | 22 | 158 | positive regulation of immune response |
| GO:0002684 | 1.80E-04 | 3.04E-03 | 22 | 158 | positive regulation of immune system process |
| GO:0045089 | 1.80E-04 | 3.04E-03 | 22 | 158 | positive regulation of innate immune response |
| GO:0000165 | 1.93E-04 | 3.24E-03 | 18 | 117 | MAPKKK cascade |
| GO:0051098 | 2.10E-04 | 3.48E-03 | 10 | 44 | regulation of binding |
| GO:0010112 | 2.11E-04 | 3.48E-03 | 6 | 16 | regulation of systemic acquired resistance |
| GO:0016881 | 2.15E-04 | 3.53E-03 | 75 | 827 | acid-amino acid ligase activity |
| GO:0019932 | 2.33E-04 | 3.79E-03 | 15 | 89 | second-messenger-mediated signaling |
| GO:0004683 | 2.33E-04 | 3.79E-03 | 15 | 89 | calmodulin-dependent protein kinase activity |

# Supplementary Table 9 FPKM of blue and green network genes

| Module | Gene_id | Gene_Name | A1_B1_Bo | A1_B1_M | A1_B1_U | A1_B1_T | A1_B2_Bo | A1_B2_M | A1_B2_U | A1_B2_T | A2_B1_Bo | A2_B1_M | A2_B1_U | A2_B1_T | A2_B2_Bo | A2_B2_M | A2_B2_U | A2_B2_T |
|---|---|---|---|---|---|---|---|---|---|---|---|---|---|---|---|---|---|---|
| Blue | Nitab4.5_0000008g0320.1 | *NtIPCS2-1* | 57.09705 | 59.0286 | 26.89085 | 28.89675 | 28.1891 | 26.4522 | 42.4342 | 15.30733 | 102.8811 | 21.7627 | 24.01475 | 7.741575 | 16.60815 | 14.39535 | 7.322295 | 26.2803 |
| | Nitab4.5_0000022g0300.1 | *NtMPK3-2* | 109.7475 | 91.4907 | 25.23565 | 29.1391 | 21.49914 | 13.35655 | 38.4677 | 8.65828 | 183.2555 | 12.96962 | 10.15661 | 2.617125 | 12.8566 | 5.53375 | 3.77995 | 38.27105 |
| | Nitab4.5_0000172g0510.1 | *NtPI4Kgamma3-1* | 50.85155 | 50.1562 | 24.8515 | 26.69515 | 23.5426 | 24.24155 | 32.90125 | 14.75064 | 71.33425 | 23.5224 | 18.7672 | 9.89178 | 18.85925 | 14.4662 | 11.46089 | 27.4204 |
| | Nitab4.5_0000338g0250.1 | *NtCRK2* | 6.000145 | 5.68086 | 2.106175 | 2.264135 | 2.16353 | 2.336395 | 3.445135 | 1.423958 | 8.09156 | 2.378675 | 2.485935 | 0.826529 | 2.02479 | 1.278184 | 0.789581 | 2.576885 |
| | Nitab4.5_0000391g0120.1 | *Nitab4.5_0000391g0120.1* | 24.2244 | 21.3647 | 7.830645 | 7.860915 | 6.557505 | 4.90299 | 9.91871 | 1.977214 | 46.89045 | 3.70926 | 6.033355 | 0.498968 | 6.92032 | 2.114315 | 0.853336 | 5.649265 |
| | Nitab4.5_0000441g0240.1 | *NtARF-1* | 39.2191 | 27.81775 | 9.84525 | 13.41335 | 5.44764 | 3.284545 | 18.4932 | 1.524628 | 84.46625 | 4.59775 | 5.19937 | 0.281315 | 3.999755 | 1.40446 | 0.538237 | 10.11765 |
| | Nitab4.5_0000444g0190.1 | *NtAMT2* | 48.0251 | 39.20975 | 17.63525 | 21.1937 | 13.61171 | 14.66365 | 33.04245 | 4.2851 | 92.5397 | 11.136 | 12.19041 | 2.36899 | 13.76542 | 6.73144 | 2.87951 | 22.72519 |
| | Nitab4.5_0000560g0040.1 | *NtLRR-1* | 18.24475 | 17.4587 | 6.517885 | 8.30441 | 6.45209 | 6.1016 | 10.18579 | 3.022665 | 27.99655 | 7.43052 | 5.86133 | 2.35828 | 6.265875 | 3.65777 | 1.97142 | 9.35009 |
| | Nitab4.5_0000736g0050.1 | *NtDUF1645-1* | 26.50705 | 24.9497 | 8.28371 | 9.374625 | 8.768665 | 9.486905 | 14.8569 | 3.34631 | 39.07745 | 7.22198 | 7.49423 | 1.160561 | 7.650495 | 3.702945 | 1.588182 | 9.24617 |
| | Nitab4.5_0000916g0100.1 | *NtPCR2-1* | 117.152 | 94.3206 | 34.0522 | 36.96505 | 36.4743 | 27.1606 | 53.97965 | 14.91172 | 168.188 | 26.68505 | 29.54255 | 5.13709 | 30.93735 | 11.53091 | 7.26014 | 46.00345 |
| | Nitab4.5_0001413g0010.1 | *NtDUF1645-2* | 37.6207 | 44.3152 | 12.33815 | 14.0076 | 15.84768 | 12.43892 | 18.91765 | 3.760835 | 61.7073 | 10.44608 | 11.54007 | 1.525517 | 13.03729 | 4.889965 | 2.582375 | 14.84465 |
| | Nitab4.5_0001781g0200.1 | *NtVPS60.1* | 28.5729 | 26.82195 | 13.31665 | 15.559 | 11.17309 | 11.84275 | 22.0241 | 7.783025 | 47.86265 | 11.46455 | 10.23148 | 4.849425 | 10.43397 | 7.67912 | 4.62802 | 12.75519 |
| | Nitab4.5_0002052g0010.1 | *NtACL* | 43.1616 | 52.5881 | 10.11311 | 10.5992 | 10.49004 | 6.57568 | 19.96525 | 3.93325 | 89.76695 | 6.36179 | 4.565825 | 0.899713 | 5.363825 | 2.79397 | 1.605726 | 9.06842 |
| | Nitab4.5_0002152g0030.1 | *NtIPCS2-2* | 58.24345 | 48.24705 | 22.4218 | 25.77235 | 21.14095 | 20.6314 | 45.0967 | 9.537865 | 111.6789 | 15.79635 | 17.21545 | 4.937205 | 13.75085 | 9.377605 | 5.4405 | 28.0351 |
| | Nitab4.5_0002543g0110.1 | *NtCaLB* | 69.5968 | 57.0112 | 16.5911 | 17.1642 | 18.49584 | 16.9145 | 28.3078 | 9.27771 | 104.4658 | 15.4243 | 11.55676 | 6.04814 | 14.67995 | 9.588275 | 6.38192 | 23.88788 |
| | Nitab4.5_0002716g0070.1 | *NtRAP2.12* | 161.6725 | 132.727 | 92.39175 | 87.1942 | 75.21255 | 75.9179 | 113.9255 | 47.8923 | 243.6365 | 68.88855 | 67.20185 | 27.7969 | 75.3085 | 47.545 | 32.45015 | 94.3954 |
| | Nitab4.5_0003233g0040.1 | *NtFBX* | 23.488 | 18.0097 | 10.59775 | 13.0011 | 9.39395 | 8.739885 | 16.4918 | 3.25654 | 46.1178 | 9.11496 | 8.6743 | 3.184025 | 9.70734 | 4.85357 | 2.907895 | 14.20031 |
| | Nitab4.5_0003289g0030.1 | *NtTET8* | 422.4645 | 396.3415 | 140.9433 | 145.9565 | 140.0378 | 127.9535 | 196.2945 | 79.27145 | 646.515 | 109.7672 | 93.75965 | 37.09545 | 95.06685 | 59.8117 | 42.09155 | 134.9987 |
| | Nitab4.5_0004471g0060.1 | *Nitab4.5_0004471g0060.1* | 51.5558 | 37.0464 | 13.54595 | 15.0759 | 11.10283 | 9.4141 | 22.1176 | 3.356163 | 83.91145 | 6.418785 | 11.30983 | 0.648474 | 11.98437 | 2.246807 | 0.755906 | 10.69028 |
| | Nitab4.5_0004654g0040.1 | *NtNAC062* | 70.98905 | 59.38665 | 25.20925 | 28.15705 | 24.0788 | 28.4977 | 42.25595 | 19.293 | 112.3211 | 23.8563 | 21.69875 | 11.10334 | 19.4163 | 14.61355 | 9.685565 | 26.8115 |
| | Nitab4.5_0006899g0030.1 | *NtAP-1* | 10.7726 | 7.786205 | 4.68957 | 4.42626 | 4.96736 | 4.45477 | 6.487925 | 2.68229 | 20.5618 | 3.43284 | 3.980565 | 1.590785 | 3.19542 | 2.187935 | 1.46247 | 5.042225 |
| | Nitab4.5_0007460g0010.1 | *NtMES13* | 20.16295 | 20.478 | 2.638745 | 3.929255 | 5.398445 | 2.60377 | 7.070245 | 0.719898 | 34.3937 | 2.1979 | 2.005855 | 0.076472 | 2.08361 | 0.92565 | 0.156558 | 4.34015 |
| | Nitab4.5_0009389g0010.1 | *NtVPS28-2* | 23.99335 | 26.8106 | 14.655 | 12.4633 | 12.5148 | 12.72885 | 19.23965 | 7.328195 | 37.7332 | 10.23413 | 11.02549 | 6.009175 | 12.11611 | 7.817325 | 5.061095 | 13.47284 |
| | Nitab4.5_0010801g0010.1 | *NtAP-2* | 15.91925 | 12.11235 | 3.95057 | 4.94103 | 4.437385 | 4.85885 | 8.671795 | 2.39468 | 21.51685 | 4.680845 | 3.98236 | 1.704425 | 4.365425 | 3.59375 | 1.71592 | 6.334555 |
| | Nitab4.5_0010911g0020.1 | *NtLRR-2* | 17.14375 | 13.5859 | 6.740085 | 8.191105 | 5.516125 | 4.631705 | 10.31623 | 2.629345 | 25.54665 | 5.524965 | 4.94665 | 1.703655 | 5.01365 | 3.111775 | 1.649595 | 9.17802 |
| | Nitab4.5_0011081g0020.1 | *NtPCR2-2* | 91.0402 | 59.41555 | 20.03605 | 18.6773 | 28.60466 | 10.87343 | 34.0032 | 5.801505 | 141.728 | 10.82137 | 13.19647 | 1.77317 | 17.30787 | 5.746825 | 2.67143 | 35.10777 |
| | Nitab4.5_0011106g0010.1 | *NtARF-2* | 133.752 | 106.4449 | 26.00725 | 26.9241 | 38.65746 | 13.45445 | 43.95565 | 8.44092 | 218.332 | 14.30729 | 14.07924 | 1.833535 | 17.99103 | 6.944895 | 3.001525 | 37.78926 |
| | Nitab4.5_0013692g0010.1 | *NtMPK3-1* | 50.1592 | 49.64495 | 12.29795 | 13.7107 | 14.82165 | 8.416575 | 19.4564 | 4.758655 | 94.57375 | 7.416355 | 6.644345 | 1.612005 | 8.802605 | 3.59551 | 1.966055 | 18.30933 |
| | Nitab4.5_0013740g0020.1 | *NtPI4Kgamma3-2* | 49.34355 | 51.24535 | 29.57805 | 30.42455 | 29.31285 | 29.25065 | 35.65575 | 19.78945 | 70.62205 | 27.8447 | 22.3855 | 14.8615 | 24.5256 | 20.46885 | 14.9858 | 29.96215 |
| | Nitab4.5_0000019g0400.1 | *NtARF-3* | 61.85685 | 25.97355 | 7.836 | 8.394725 | 11.68435 | 5.23215 | 17.46785 | 2.497843 | 90.21685 | 5.220555 | 6.067405 | 0.534529 | 6.67234 | 1.791971 | 0.845989 | 16.44885 |
| Green | Nitab4.5_0000078g0290.1 | *NtGDSL* | 0.905262 | 0.810977 | 0.555957 | 0.735018 | 0.652695 | 0.735001 | 0.692283 | 3.266475 | 0.156684 | 0.971061 | 1.252087 | 3.617415 | 0.432976 | 0.959136 | 2.99938 | 1.345929 |
| | Nitab4.5_0000255g0150.1 | *NtBGAL1* | 1.984775 | 1.99213 | 2.38093 | 3.27681 | 1.116886 | 1.865105 | 2.214927 | 8.455985 | 1.025341 | 2.87237 | 3.702145 | 9.43473 | 1.396285 | 2.481925 | 6.56097 | 4.672895 |

| | | | | | | | | | | | | | | | | | |
|---|---|---|---|---|---|---|---|---|---|---|---|---|---|---|---|---|---|
| Nitab4.5_0000305g0210.1 | *Nitab4.5_000 0305g0210.1* | 356.26 | 254.7665 | 330.973 | 333.9075 | 269.833 | 267.4965 | 284.155 | 522.3 | 156.209 | 292.9 | 354.8885 | 611.8435 | 255.592 | 326.6905 | 622.6855 | 425.0755 |
| Nitab4.5_0000335g0140.1 | *NtCLC-1* | 22.3099 | 24.5764 | 22.0152 | 19.8493 | 32.8985 | 35.61335 | 40.25415 | 4.993505 | 59.78995 | 25.6332 | 21.02025 | 4.81537 | 37.68735 | 20.98595 | 6.00247 | 15.9046 |
| Nitab4.5_0000343g0260.1 | *NtSBT1.8* | 2.30619 | 1.858185 | 3.088595 | 3.31647 | 2.642765 | 3.486415 | 2.725795 | 10.8582 | 1.217379 | 3.38444 | 4.266405 | 11.53514 | 2.302915 | 2.82233 | 8.70073 | 4.407855 |
| Nitab4.5_0000348g0200.1 | *NtKASI* | 15.68175 | 7.95191 | 17.7666 | 20.2276 | 8.300825 | 7.436855 | 12.88287 | 32.7468 | 6.725385 | 12.58887 | 15.09915 | 45.56515 | 9.323845 | 13.53615 | 31.40365 | 19.78395 |
| Nitab4.5_0000461g0190.1 | *NtZIP11* | 13.6914 | 17.3895 | 13.79285 | 14.6935 | 19.35245 | 19.60705 | 21.8 | 5.162365 | 31.682 | 17 | 12.89665 | 5.572995 | 22.2128 | 13.4342 | 5.762755 | 11.20935 |
| Nitab4.5_0000482g0200.1 | *NtGH9B8* | 0.367456 | 0.574742 | 1.031176 | 1.129054 | 0.256653 | 0.295666 | 0.662106 | 6.15679 | 0.200342 | 0.574518 | 2.131042 | 7.560105 | 0.300761 | 0.631818 | 4.93843 | 1.769245 |
| Nitab4.5_0000482g0280.1 | *NtO-GlyHy-1* | 2.142155 | 2.055015 | 2.602375 | 2.394475 | 1.432805 | 1.892395 | 2.311693 | 13.3393 | 0.597658 | 1.62865 | 3.50043 | 15.88913 | 1.563 | 1.91122 | 12.60425 | 4.60619 |
| Nitab4.5_0000672g0140.1 | *NtPMCT* | 6.68987 | 6.246825 | 5.402155 | 4.92473 | 6.739405 | 7.116605 | 6.55114 | 2.66987 | 11.18435 | 5.72016 | 5.55308 | 2.44952 | 8.586765 | 4.898815 | 2.735115 | 5.23143 |
| Nitab4.5_0001881g0020.1 | *NtFAD3* | 0.609715 | 0.868106 | 1.065003 | 1.160558 | 0.547555 | 1.507803 | 0.571822 | 7.88082 | 0.320165 | 1.094507 | 1.489136 | 8.332285 | 0.772378 | 1.617415 | 5.26957 | 2.663495 |
| Nitab4.5_0002011g0010.1 | *NtEMP1-1* | 2.412105 | 2.24586 | 2.219125 | 2.46132 | 1.54343 | 2.452525 | 1.748955 | 7.333425 | 1.242067 | 2.94203 | 3.19785 | 7.00111 | 1.856875 | 2.80068 | 6.48063 | 3.12611 |
| Nitab4.5_0002467g0010.1 | *NtRGP3* | 6.142135 | 6.819395 | 6.469035 | 7.160025 | 4.67729 | 6.69812 | 5.19682 | 31.47475 | 4.816535 | 7.84187 | 9.02782 | 31.6655 | 4.67316 | 8.38353 | 19.48475 | 10.82308 |
| Nitab4.5_0002827g0090.1 | *NtADT1* | 1.208595 | 0.53088 | 1.288258 | 1.555655 | 0.701011 | 0.805509 | 1.189616 | 3.297255 | 0.544612 | 1.243098 | 1.801575 | 4.260845 | 0.523084 | 1.260835 | 2.85693 | 2.03081 |
| Nitab4.5_0002947g0040.1 | *NtEMP1-2* | 1.77325 | 1.838315 | 2.23233 | 2.44015 | 1.43281 | 2.04807 | 1.464701 | 8.026635 | 0.726456 | 2.97635 | 2.97391 | 8.439695 | 1.270574 | 2.881855 | 7.52161 | 2.83359 |
| Nitab4.5_0003434g0070.1 | *NtXYP1* | 3.21038 | 5.387265 | 5.388355 | 4.85609 | 3.306765 | 4.380085 | 4.616565 | 24.727 | 1.759579 | 7.341775 | 8.916725 | 28.4054 | 3.30757 | 6.174125 | 20.54785 | 10.09267 |
| Nitab4.5_0003481g0010.1 | *NtAP-3* | 1.183395 | 0.98997 | 1.84951 | 2.285175 | 1.138055 | 1.326685 | 1.547923 | 23.06975 | 0.637419 | 1.62199 | 3.54 | 46.46541 | 0.990541 | 1.350938 | 18.16711 | 6.398115 |
| Nitab4.5_0003815g0060.1 | *NtPKP-ALPHA* | 1.80224 | 1.21821 | 2.83893 | 2.702505 | 0.962832 | 1.4779 | 2.12252 | 7.699875 | 0.936432 | 1.35278 | 2.346045 | 9.656105 | 0.895104 | 1.688895 | 6.142725 | 4.347475 |
| Nitab4.5_0003987g0050.1 | *Nitab4.5_000 3987g0050.1* | 0.920621 | 1.159985 | 1.382305 | 1.77439 | 1.22633 | 1.307835 | 1.123426 | 4.46131 | 0.524616 | 1.686855 | 1.773997 | 5.80431 | 1.135967 | 2.19166 | 4.516065 | 2.0779 |
| Nitab4.5_0004215g0040.1 | *NtFLA7* | 1.029694 | 1.79866 | 1.844835 | 1.579105 | 1.358175 | 1.57364 | 1.396248 | 11.75275 | 0.722868 | 2.415505 | 4.24461 | 11.22855 | 1.458055 | 2.70241 | 8.11562 | 3.582555 |
| Nitab4.5_0005008g0010.1 | *NtO-GlyHy-2* | 0.500728 | 0.917371 | 1.259395 | 1.415921 | 0.72705 | 0.590028 | 1.05871 | 14.42094 | 0.201936 | 1.089674 | 1.730815 | 15.68066 | 0.379598 | 1.28298 | 10.50744 | 3.2455 |
| Nitab4.5_0005776g0010.1 | *NtCLC2-2* | 22.903 | 24.70105 | 23.61255 | 25.6485 | 29.23785 | 30.89605 | 37.79205 | 10.3261 | 49.2042 | 26.19145 | 25.18285 | 8.426355 | 31.5213 | 22.48425 | 9.125815 | 19.6879 |
| Nitab4.5_0005808g0080.1 | *NtAP-4* | 0.983593 | 1.49448 | 3.39899 | 4.15387 | 1.09608 | 1.210885 | 2.072224 | 44.25625 | 0.150144 | 3.45233 | 7.2651 | 55.2802 | 1.149511 | 2.89762 | 33.551 | 9.021615 |
| Nitab4.5_0006221g0010.1 | *Nitab4.5_000 6221g0010.1* | 86.6831 | 73.50635 | 75.29325 | 67.5313 | 84.554 | 78.4808 | 105.5704 | 28.7263 | 127.823 | 58.71635 | 61.4226 | 28.87475 | 76.80655 | 57.06705 | 27.26725 | 64.36735 |
| Nitab4.5_0007255g0060.1 | *NtGER3* | 7.376435 | 16.9945 | 17.34484 | 17.89508 | 11.64098 | 9.08369 | 12.06353 | 143.0479 | 1.639858 | 28.05502 | 32.48859 | 232.0993 | 6.34283 | 20.25105 | 145.8595 | 61.14085 |
| Nitab4.5_0007773g0010.1 | *NtPMEI* | 0.298946 | 0.378301 | 0.68961 | 0.788621 | 0.361844 | 0.280507 | 0.221274 | 10.00488 | 0.080004 | 0.689356 | 1.380729 | 10.88985 | 0.092212 | 0.710575 | 9.218805 | 2.569545 |
| Nitab4.5_0010144g0030.1 | *NtPL-1* | 6.712775 | 6.79139 | 8.63999 | 9.25758 | 4.95553 | 6.02826 | 6.591665 | 17.3378 | 3.943345 | 8.711845 | 9.752235 | 20.57305 | 5.78101 | 8.320705 | 19.47345 | 10.29299 |
| Nitab4.5_0010728g0020.1 | *NtPL-2* | 2.131455 | 3.09575 | 2.851955 | 4.06706 | 1.847705 | 3.007465 | 2.523316 | 17.48845 | 1.042288 | 5.124975 | 7.288155 | 19.66755 | 2.76977 | 5.239675 | 15.60265 | 5.576065 |
| Nitab4.5_0022736g0010.1 | *NtAGP18* | 0.153028 | 0.461608 | 0.756494 | 0.830694 | 0.200943 | 0.163401 | 0.532783 | 2.67056 | 0.067094 | 0.527552 | 1.101065 | 4.836925 | 0.102458 | 0.516857 | 3.129895 | 1.246574 |
| Nitab4.5_0000163g0200.1 | *NtFLA1* | 0.966953 | 1.004893 | 2.068745 | 1.4509 | 0.66657 | 0.954111 | 1.619783 | 10.65529 | 0.372572 | 1.972555 | 3.623549 | 10.64605 | 0.730011 | 1.774215 | 7.8354 | 2.17846 |
